# New Synthesis and Pharmacological Evaluation of Enantiomerically Pure (*R*)- and (*S*)-Methadone Metabolites as *N*-Methyl-d-aspartate Receptor Antagonists

**DOI:** 10.1021/acs.jmedchem.4c02605

**Published:** 2025-02-25

**Authors:** Marco Banzato, Alberto Furlan, Patrizia Locatelli, Jacopo Sgrignani, Alberto Ongaro, Alessandro Dolmella, Sara De Martin, Stefano Comai, Andrea Cavalli, Charles Inturrisi, Ezio Bettini, Paolo L. Manfredi, Andrea Mattarei

**Affiliations:** † Department of Pharmaceutical and Pharmacological Sciences, University of Padova, Via Francesco Marzolo 5, 35131 Padua, Italy; ‡ Institute for Research in Biomedicine, Via Chiesa 5, 6500 Bellinzona, Switzerland; § Department of Biomedical Sciences, University of Padova, Via Ugo Bassi 58/B, 35131 Padua, Italy; ∥ Department of Psychiatry, McGill University, 1033 Pine Avenue West, Montreal, Quebec H3A 1A1, Canada; ⊥ IRCSS San Raffaele Scientific Institute, via Olgettina 58, 20132 Milan, Italy; # Relmada Therapeutics, Coral Gables, Florida 33134, United States; ∇ In Vitro Pharmacology Department, Aptuit, An Evotec Company, Via Alessandro Fleming, 4, 37135 Verona, Italy

## Abstract

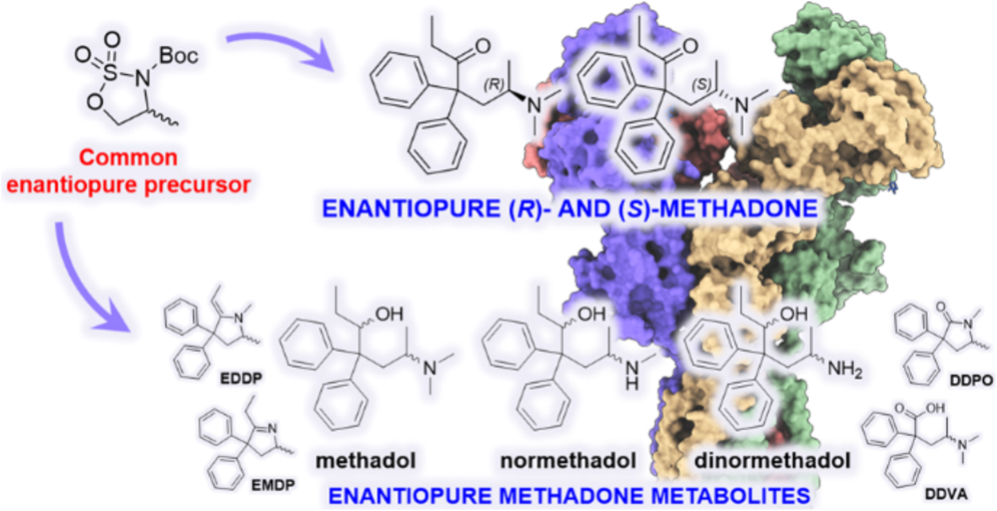

*N*-Methyl-d-aspartate receptor (NMDAR) is gaining increasing interest as a pharmacological target for the development of fast-acting antidepressants. (*S*)-Methadone (esmethadone), has recently shown promising efficacy for the treatment of major depressive disorder. However, methods for its enantiopure preparation still rely on complex and expensive resolution procedures. In addition, enantiopure methadone metabolites have never been evaluated for their NMDAR activity. Here, we report the development of a novel chiral pool approach, based on cyclic sulfamidate ring-opening reaction, for the asymmetric synthesis of (*R*)- and (*S*)-methadone, and the application of this methodology to the stereodivergent synthesis of 20 enantiopure methadone metabolites. The compounds were evaluated for their NMDAR antagonism and for their affinity toward a series of relevant CNS receptors. Strikingly, *N*-demethylated (6*R*)-methadol metabolites retain the higher NMDAR uncompetitive antagonism of (*R*)-methadone, while presenting lower opioid receptor affinity compared to (*S*)-methadone. These compounds could represent novel candidates for drug development in CNS disorders.

## Introduction

*N*-Methyl-d-aspartate receptors (NMDARs) are cation-selective glutamate-gated ion channels with high Ca^2+^ permeability belonging to the family of ionotropic glutamate receptors (iGluRs).^1^ NMDAR channel opening requires binding of the coagonists glutamate and glycine as well as membrane depolarization to disengage Mg^2+^ from the selectivity filter.^2^ Since their discovery in the 1960s, they have been the subject of intense investigation due to their key role as mediators of brain plasticity.^3^ Indeed, NMDARs are endowed with the unique ability to convert specific patterns of neuronal activity into long-term changes in synapse structure and function that are thought to correlate with cognitive functions, memory, and learning.^4^ Importantly, deregulated NMDARs are involved in various psychiatric and neurological disorders, including neuropathic pain, stroke, epilepsy, neurodegenerative diseases, schizophrenia, and depression.^5−11^

**Figure 1:**
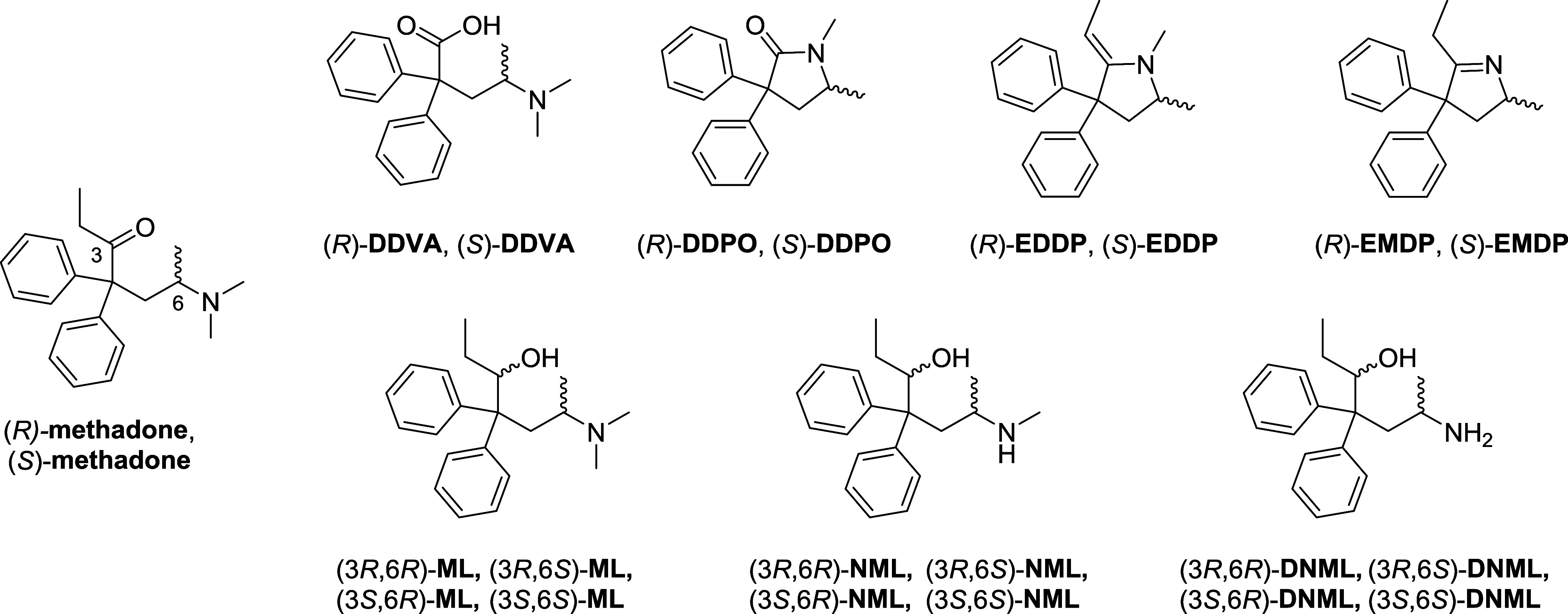
Molecular structures of methadone and its main human metabolites.

NMDARs are heteromeric tetramers derived from three related families: glycine-binding GluN1 subunits (encoded by the *GRIN1* gene), glutamate-binding GluN2 subunits (GluN2A-2D, encoded by *GRIN2A-2D*), and glycine-binding GluN3 subunits (GluN3A-3B, encoded by *GRIN3A-3B*). A functional NMDAR requires the coexpression of at least one GluN1 and one GluN2 subunit, and the classic NMDAR is composed of two GluN1 and two GluN2 subunits, where the two GluN2 subunits can be either identical or different, allowing assembly of diheteromeric or triheteromeric complexes, respectively.^1,4^ Subunit composition affects NMDAR biophysical, gating, and pharmacological properties, and their density and localization across CNS regions and during stages of development have been correlated with disease states, stimulating growing interest in the development of drugs able to interact and modulate NMDARs containing specific GluN2 subunits.^3,12−14^ In the last decades, renewed enthusiasm in the pharmacology of NMDARs has been spurred by the finding that certain low affinity uncompetitive antagonists of these ion channels produce rapid, robust, and sustained antidepressant activity, which outlasts the detectability of the drug in the body.^15−18^ Research in this field has led to the approval of esketamine, as the first fast-acting antidepressant in the new drug class of uncompetitive NMDAR antagonist antidepressants.^19^ Soon after, the uncompetitive NMDAR antagonist dextromethorphan in combination with bupropion showed efficacy in clinical trials and was then approved by the FDA, representing the first and only rapid-acting oral medication for major depressive disorder (MDD).^20^

(*R*,*S*)-Methadone (Figure 1) is a long-acting opioid that has been used since the 1940s for opioid use disorder and pain management. (*R*)-Methadone (levomethadone) is a μ-opioid receptor (MOP) agonist 8 to 50 times more potent than (*S*)-methadone (esmethadone), thus contributing almost exclusively to the opioid agonist effects of the racemic mixture.^21,22^ Furthermore, as recently demonstrated by Levinstein et al.,^23^ esmethadone exhibits unique pharmacodynamic properties by acting as a MOP antagonist when MOP is complexed with the galanin 1 receptor (Gal_1_R), which explain its lack of opioid-like agonistic effects, including dopamine mediated reinforcing effects in animals,^24^ and in humans.^25^ While both methadone enantiomers are uncompetitive NMDAR antagonists with lower potency compared to ketamine,^26^ the lack of meaningful opioid and psychotomimetic effects of esmethadone, along with promising preclinical results,^27^ has led to its clinical investigation as a new fast-acting antidepressant drug candidate.^28^ Esmethadone showed safety and increased circulating brain-derived neurotrophic factor (BDNF) levels in healthy adults in a phase 1 clinical study,^29^ showed rapid and sustained antidepressant effects in a phase 2 clinical study,^30^ and encouraging results in a phase 3 clinical study.^31^ Importantly, despite the activity of esmethadone as an uncompetitive NMDAR antagonist is well established in vitro,^26^ its in vivo mechanism of action (MOA) has been under debate.^23,32^ The understanding of the precise MOA by which esmethadone exerts antidepressant-like effects in animal models^27,33^ and in humans^30,31^ remains a work in progress. In the meantime, blocking NMDARs at resting membrane potential is a well-grounded hypothesis for explaining the antidepressant effects of uncompetitive NMDAR antagonists like esmethadone^32,34,35^ and ketamine.^36,37^

Notably, findings on the molecular mechanisms underlying the antidepressant actions of (*R*,*S*)-ketamine identified one of its metabolites, (2*S*,6*S*;2*R*,6*R*)-hydroxynorketamine (HNK), particularly the (2*R*,6*R*)-HNK enantiomer, as essential for its antidepressant effects.^38^ Interestingly, methadone shares metabolic pathways with ketamine, leading to metabolites with similar molecular features (Figure 1). The two most abundant metabolites of methadone are 1,5-dimethyl-3,3-diphenyl-2-ethylidenepyrrolidine (**EDDP**) and 2-ethyl-5-methyl-3,3-diphenylpyrroline (**EMDP**). These metabolites result from two consequential enzymatic demethylations of methadone (by CYP3A4 and CYP2B6), with the first demethylation causing a spontaneous reaction between the ketone and the secondary amine, forming a cyclic enamine. In addition, methadone metabolism includes a pool of less abundant metabolites: 4-dimethylamino-2,2-diphenylvaleric acid (**DDVA**) can be obtained from the enzymatic oxidation of the ketone side chain, which can then cyclize into 1,5-dimethyl-3,3-diphenyl-2-pyrrolidone (**DDPO**). Furthermore, reduction of the ketone group (by alcohol dehydrogenase), followed by one or two CYP-mediated *N*-demethylations, generates a set of compounds known as methadols (**ML**), normethadols (**NML**) and dinormethadols (**DNML**), each existing as pairs of diastereomers.^39^

To date, the activity toward NMDARs of methadone metabolites and their potential role in its antidepressant activity have never been investigated. A thorough review of the literature revealed that only a few inefficient procedures have been reported for the synthesis of methadone metabolites, primarily as racemic mixtures.^40−42^ Notably, also the previously reported procedures for the synthesis of enantiopure (*R*)- or (*S*)-methadone have significant drawbacks affecting efficiency,^43^ or require laborious enantiomeric resolution techniques to obtain pure isolated stereoisomers.^44^

The aim of the present study was to develop a new efficient synthesis strategy for the preparation of optically pure (*R*)- and (*S*)-methadone, and their main enantiopure metabolites, and to assess, for the first time, their activity toward diheteromeric NMDARs containing specific GluN2 subunits. Additionally, the pharmacological profile of methadone metabolites with the highest antagonistic effect toward NMDAR was outlined by evaluating their ability to displace known radiolabeled ligands of selected relevant receptors in the CNS, with the final aim of selecting the most promising compounds as second-generation methadone derivative clinical candidates.

## Results and Discussion

**Scheme 1:**
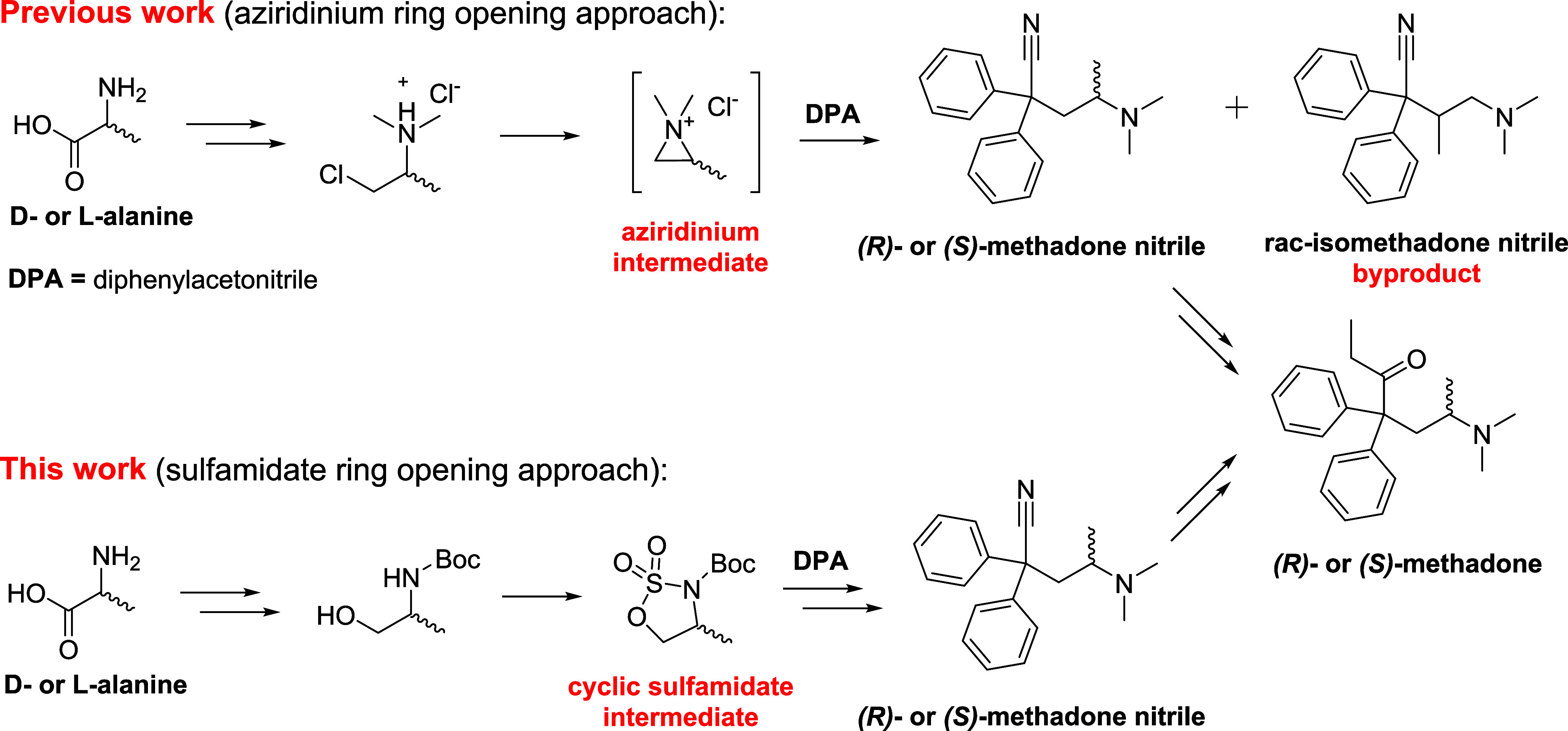
Chiral Pool Synthesis of (*R*)- or (*S*)-Methadone Based on Aziridinium Ring-Opening Reaction Strategy and Novel Cyclic Sulfamidate Ring-Opening Reaction Approach, Allowing for Improved Regiochemical Selectivity, Process Simplification, and Increased Overall Yield

Although almost a century has passed since the discovery of methadone, the 2023 aggregate production quota in the US established by the Drug Enforcement Administration (DEA) for this API amounts to 25,620 kg, underlining the clinical relevance of this active ingredient, dispensed mainly in opioid treatment programs (OTPs), colloquially known as methadone maintenance programs.^45^ Methadone is generally marketed as a racemic mixture, so it is not surprising that methodologies for its production as a single enantiomer are scarcely reported in the literature. However, the recent interest sparked by the development of the (*S*)-methadone enantiomer for the treatment of MDD, combined with the prospect of using half the dose of the (*R*)-methadone enantiomer in the OTPs with lower risk of QT interval prolongation compared to (*R*,*S*)-methadone,^46^ highlighted the need for developing efficient procedures for the preparation of pure methadone stereoisomers. The chiral pool synthesis (Scheme 1, *Previous work*) developed by Mkrtchyan et al.^47^ in 2017, represented an advance in the state of the art for the asymmetric synthesis of either (*R*)- and (*S*)-methadone, starting from d- or l-alanine respectively with about 20% overall yield, and an enantiomeric excess greater than 99%. However, the key step in this process still relies on the classic synthesis used to produce the racemic mixture of methadone, namely the attack of 2,2-diphenylacetonitrile anion on the 1,1,2-trimethylaziridin-1-ium generated *in situ* by intramolecular S_N_2-reaction of 1-dimethylamino-2-chloropropane under basic conditions. In this setup, the diphenylacetonitrile anion can attack from two sides of the aziridinium salt resulting in competitive ring-opening reactions, with retention of configuration for the desired intermediate, i.e., 4-(dimethylamino)-2,2-diphenylpentanenitrile, methadone nitrile, but low levels of regiochemical discrimination, i.e., formation of the byproduct isomethadone nitrile. Importantly, under the optimal mixture of base, solvent, and reaction conditions, the outcome of this reaction is at best a 1:3 (isomethadone nitrile:methadone nitrile) mixture of the two regioisomeric products. The resulting mixture is then purified by adopting a complex crystallization process that exploit the higher solubility of isomethadone-nitrile, leading to nonefficient recovery and low purified yield of the desired methadone nitrile intermediate, which is then converted to optically pure methadone through the classic ethyl Grignard addition to the nitrile group, and acid-mediated hydrolysis of the resulting imine intermediate.^47^

**Scheme 2:**
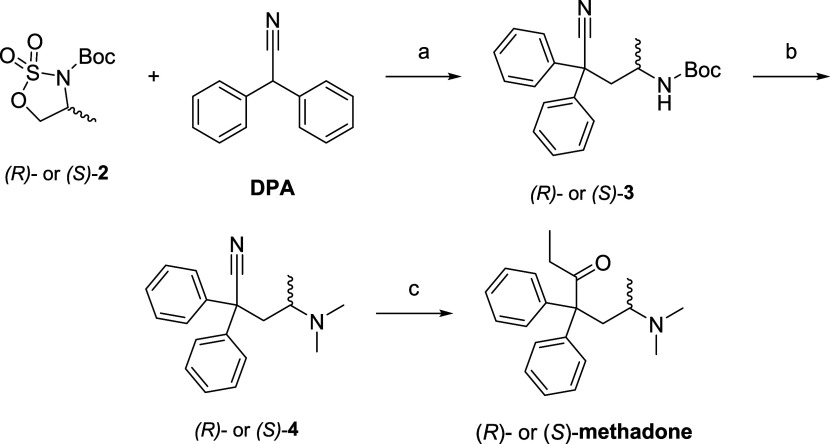
Chiral Pool Synthesis of Enantiomerically Pure (*R*)- or (*S*)-Methadone via a Novel Cyclic Sulfamidate Ring-Opening Reaction Approach Reagents and conditions: (a) NaHMDS, THF, −20 °C, 1 h, then aq. citric acid, 90%; (b) HCl in MeOH, MeOH 0 °C, 4 h, then CH_2_O aq., ACN/H_2_O, NaHB(OAc)_3_, rt, 1 h, 96%; and (c) EtMgBr, toluene, 100 °C, 4 h, then 6 M aq. HCl, 50 °C, 16 h, 93%.

We have now developed a new synthesis strategy for (*R*)- or (*S*)-methadone (Scheme 1, *This work*) that envisions an enantio-retentive process, based on the strategic exploitation of the chiral pool employing enantiopure d- or l-alanine-derived *N-Boc*-protected cyclic 4-methyl-sulfamidate as key starting material. In the novel synthesis (Scheme 2) cyclic sulfamidate ring-opening reaction with diphenylacetonitrile, in the presence of NaHMDS, allows the obtainment of the *N-Boc*-protected intermediate **3** in excellent yield with retention of configuration, completely precluding the formation of the regioisomeric isomethadone byproduct. The following reaction in the new process is a two-step/one-pot *N-Boc*-deprotection/reductive amination of **3**, leading to the synthesis of the common intermediate (*R*)- or (*S*)-methadone nitrile **4**, in almost quantitative yield. Finally, treatment of **4** with a slight excess of EtMgBr results in the generation of the corresponding ethyl-imine, which is readily hydrolyzed under acidic conditions to provide (*R*)- or (*S*)-methadone, as already reported in previous syntheses.^47^ The new process results in an overall yield of 63%, and an enantiomeric excess (See Supporting Information) greater than 99%, clearly outperforming all the previously reported procedures, greatly simplifying the operations required to obtain enantiomerically pure methadones.

**Scheme 3:**
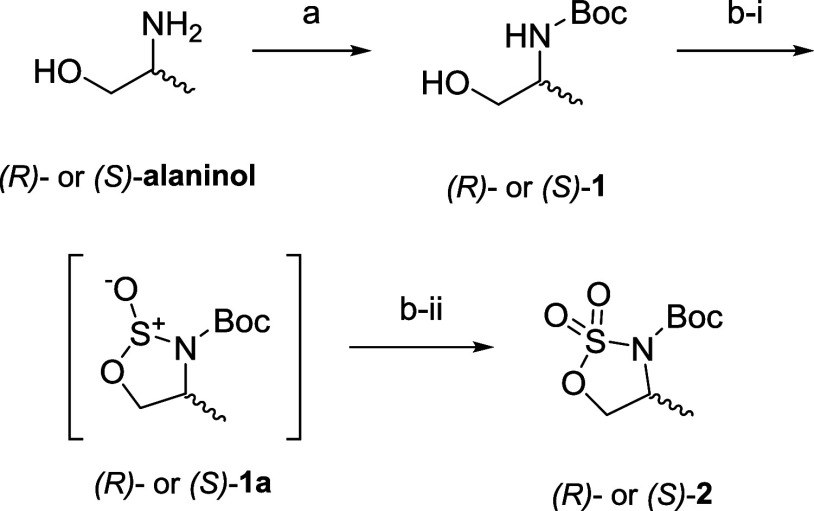
Synthesis of the Alanine-Derived Enantiomerically Pure Cyclic Sulfamidates ((*R*)-**2** or (*S*)-**2**) Reagents and conditions: (a) Boc_2_O, TEA, DCM, rt, 2.5 h, 88%; (b-i) SOCl_2_, TEA, imidazole, DCM, −40 °C, then −60 °C to rt, 16 h; and (b-ii) NaIO_4_, RuCl_3_, ACN/H_2_O, 0 °C, 3 h, 86% over two steps.

The key reagent, *N-Boc*-protected cyclic 4-methyl-sulfamidate (**2**) was produced by optimizing an already reported protocol^48^ through a two-stage synthesis starting from commercially available enantiopure (*R*)- or (*S*)-alaninol (Scheme 3). The first stage is the *N-Boc*-protection of the amino group (**1**). The second stage is a two-steps/one-pot process to obtain first the *N-Boc*-protected cyclic sulfamidite intermediate (**1a**, reaction b-i) by cyclization reaction with thionyl chloride in the presence of imidazole and TEA followed by the oxidation of the sulfur atom in the presence of sodium periodate as an oxidizing agent and ruthenium(III) chloride as a catalyst (reaction b-ii) to obtain the desired (*R*)- or (*S*)-**2** in 86% overall yield.

**Scheme 4:**
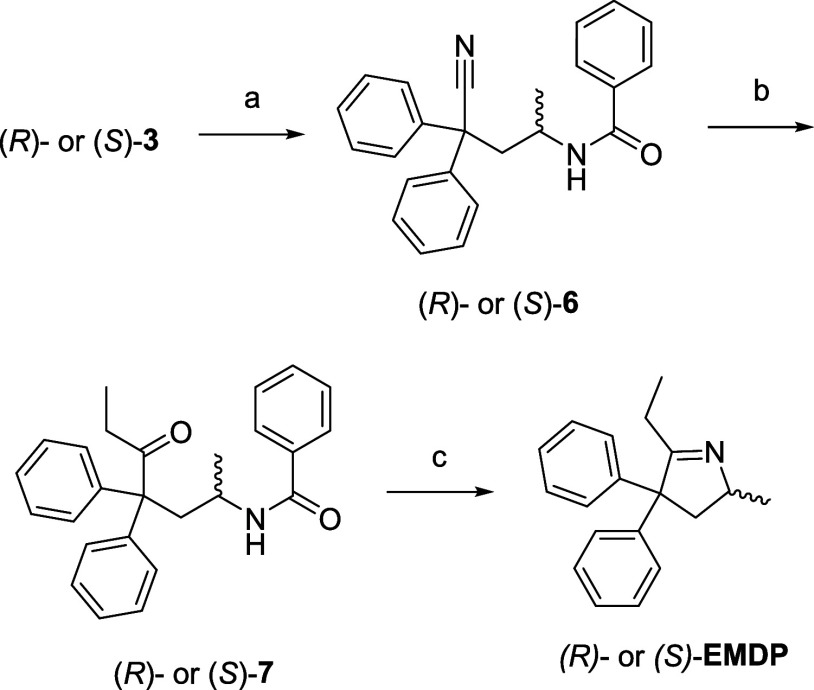
Synthesis of (*R*)-**EMDP** or (*S*)-**EMDP**, Enantiomerically Pure Methadone Metabolites Reagents and conditions. (a) HCl in dioxane, DCM, 0 °C, 3 h, then BzCl, TEA, DCM, 0 °C, 1 h, 98%; (b) EtMgBr, toluene, 100 °C, 4 h, then 10% aq. CH_3_COOH, 75 °C, 20 h, 76%; and (c) 6 M aq. HCl, dioxane, 100 °C, 2 h, 85%.

The efficient production of an enantiopure *N-Boc*-protected analogue of the methadone nitrile intermediate, i.e., (*R*)-**3** or (*S*)-**3**, prompted us to evaluate the exploitation of this novel synthesis approach in the preparation of the main known optically pure methadone human metabolites (Figure 1). In particular, while *N,N*-dimethylmethadols (**ML**s) in their OH-acetylated form have been extensively investigated in the past, and clinically administered as analgesics or in OTPs as an alternative to methadone,^49^ their *N*-demethylated analogues (**NMLs**, and **DNML**s) have been only poorly pharmacologically characterized,^50^ probably because of the complexity of their preparation. To the best of our knowledge, the synthesis of *N*-demethylated methadols has only been approached in one previous report by Booher and Pohland,^41^ through an extremely complex, low-yield process, starting from a racemic mixture of α-(±)-acetylmethadol, and without obtaining the isolated stereoisomers. Here, starting from enantiopure (*R*)- or (*S*)-*N-Boc*-protected methadone nitrile intermediates ((*R*)-**3** or (*S*)-**3**), we first replaced the *N-Boc*-protecting group with a more stable benzamide group ((*R*)-**6** or (*S*)-**6**), to allow the conversion to the corresponding ethyl ketone ((*R*)-**7** or (*S*)-**7**) via Grignard addition. Notably, intermediate **7** can be easily converted in high yield to enantiopure (*R*)-**EMDP** or (*S*)-**EMDP** by a simple hydrochloric acid-mediated amide hydrolysis (Scheme 4), since the free amino group spontaneously reacts by intramolecular cyclization with the ketone, providing an easy approach for the asymmetric synthesis of this major methadone metabolite.

**Scheme 5:**
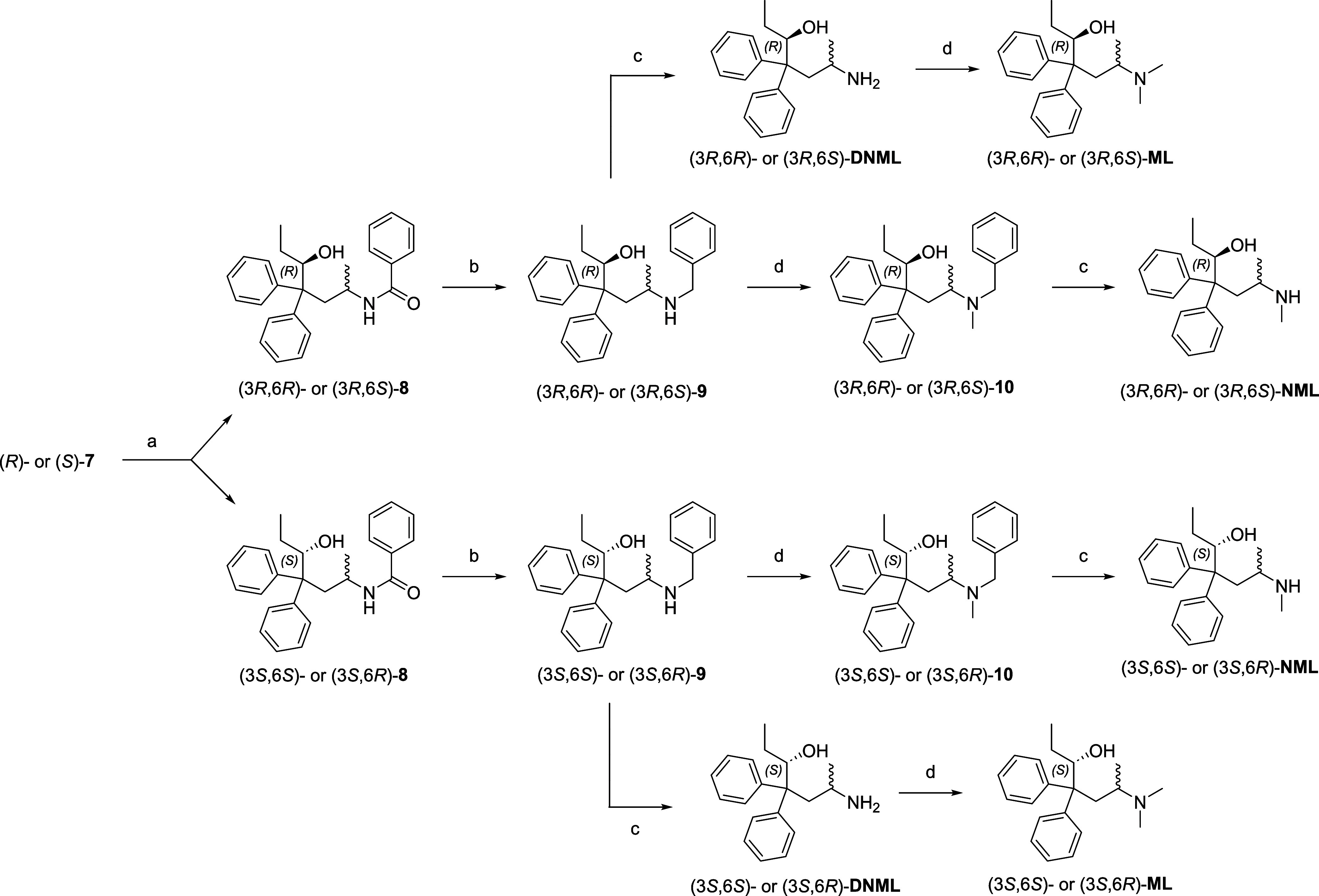
Synthesis of (3*R*,6*R*)-, (3*R*,6*S*)-, (3*S*,6*S*)-, or (3*S*, 6*R*)-Methadols (**MLs**, **NMLs**, **DNMLs**), Enantiomerically Pure Methadone Metabolites Reagents and conditions. (a) LAH, THF, 0 °C, 1 h, 96%, chromatographic separation of the stereoisomers; (b) LAH, THF, reflux, 24–48 h, quant.; (c) Pd/C, NH_4_HCO_2_, EtOH, 60 °C, 3 h, 88–94%; (d) aq. CH_2_O, NaHB(OAc)_3_, ACN, rt, 1 h, quant.′

On the other hand, the reduction of the ketone group of (*R*)-**7** or (*S*)-**7** to alcohol (Scheme 5), using LiAlH_4_ as reducing agent under mild conditions (0 °C), generates *N*-benzoyl-protected dinormethadols (**DNML**s), as two diastereoisomers for each starting material (**8**) that were easily isolated as the corresponding pure stereoisomers by column chromatography on silica gel. The reduction reaction resulted in about 18% diastereomeric excess of the α-stereoisomer, namely (3*R*,6*R*)-**8** or (3*S*,6*S*)-**8**. Chirality of the newly generated stereocenter was unambiguously assigned by controlled crystallization followed by single-crystal XRD for each purified stereoisomer, and optical purity was assessed by chiral chromatography analysis (See Supporting Information). The four isolated, enantiomerically pure stereoisomers of **8**, were then subjected to a second treatment with LiAlH_4_ in harsher conditions, to achieve the reduction of the benzamide moiety to benzyl group (**9**). Notably, complete reduction of **7** to **9** was not performed in a single step to allow efficient chromatographic separation of **8**-diasteroisomers, which was much more complex in the presence of a basic benzylamine group. *N*-benzyl protected dinormethadols (**9**) were employed as starting materials to obtain enantiopure dinormethadols (**DNML**s) by Pd/C catalyzed debenzylation reaction in the presence of ammonium formate, or enantiopure normethadols (**NML**s), by reductive amination in the presence of NaBH(OAc)_3_ and formaldehyde (to intermediate **10**) followed by debenzylation reaction. Finally, enantiopure methadols were obtained from the reductive amination of **DNML** under the aforementioned reductive amination conditions in quantitative yield (Scheme 5).

**Scheme 6:**
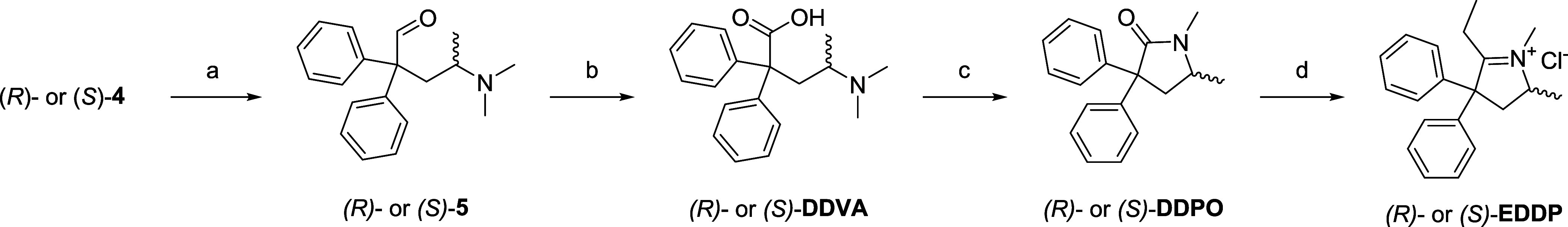
Synthesis of (R)- or (S)-**DDVA**, **DDPO**, **EDDP**, Enantiomerically Pure Methadone Metabolites Reagents and conditions: (a) 3-pentyl MgBr, toluene, 100 °C, 4 h, then 6 M aq. HCl, rt, 16 h, 98%; (b) H2O2, NaH_2_PO_4_, NaClO_2_, ACN/H_2_O, 0 °C to rt, 4 h, 83%; (c) SOCl_2_, CHCl_3_, DMF, 70 °C, 4 h, 92%; and (d) EtLi, Et_2_O, rt, 3 h, then 4 M HCl in dioxane, 92%.

To complete the preparation of the remaining enantiopure methadone metabolites (**DVVA**, **DDPO**, **EDDP**, Scheme 6) planned in this study, mainly previously reported procedures were envisioned with minor modifications, starting from the enantiopure methadone nitrile intermediates (*R*)-**4** or (*S*)-**4**. In particular, as previously reported,^51^ methadone nitrile could have been directly converted to **DDVA** by a sulfuric acid-mediated nitrile hydrolysis, which, in our hands, resulted in a complex mixture of byproducts, and poor isolated yield. Serendipitously, we found a way to bypass this issue by converting the nitrile group of **4** to the corresponding aldehyde (**5**) in 98% yield by reaction with 3-pentylmagnesium bromide followed by hydrochloric acid-mediated hydrolysis. **5** was then easily converted in quantitative yield to the carboxy analogue (**DDVA**) under Pinnick oxidation conditions using NaClO_2_ as oxidant at buffered pH, and H_2_O_2_ as hypochlorite scavenger. **DDVA** was then treated with SOCl_2_, promoting a simultaneous cyclization-demethylation, to obtain **DDPO**,^51^ which was finally treated with ethyllithium to generate the cyclic enamine **EDDP** as previously reported.^42^

All the 20 enantiomerically pure main methadone metabolites synthesized were accurately characterized by means of ^1^H, ^13^C NMR spectroscopy, HRMS, polarimetry, UPLC-UV, and chiral HPLC (Spectra and chromatograms are shown in the Supporting Information). To further confirm the absolute configuration of methadols and normethadols, final products were crystallized and analyzed by XRD (Supporting Information, CCDC deposition numbers 2369660–2369671). Unfortunately, we were unable to obtain suitable crystals of **DNML**s for XRD, but their absolute configuration was assigned by XRD of a previous intermediate in the synthetic path, i.e., **8**, for which retention of configuration in the following steps was demonstrated with the other synthesized analogues.

### In Vitro Fluorometric Imaging Plate Reader (FLIPR)/Ca^2+^ Assay

To evaluate the activity of synthesized methadone metabolites on NMDAR, a series of in vitro experiments using the fluorometric imaging plate reader (FLIPR) Ca^2+^ assay were conducted at Aptuit, an Evotec Company (Italy). The FLIPR assay allows for high-throughput quantification of Ca^2+^ influx, providing a robust platform for comparing the relative efficacy of each compound synthesized. We tested the different metabolites alongside known uncompetitive NMDAR channel blockers, i.e., dextromethorphan, (±)-ketamine, memantine, and (+)-MK-801. These compounds were assessed for their effects on specific heterodimeric NMDAR subtypes stably overexpressed in Chinese Hamster Ovary (CHO) cells. The subtypes tested included GluN1-GluN2A, GluN1-GluN2B, GluN1-GluN2C, and GluN1-GluN2D. CHO cells expressing each of the four NMDAR subtypes were incubated with 10 μM l-glutamate, a concentration known to elicit maximal Ca^2+^ influx, serving as the stimulus. Intracellular Ca^2+^ levels were subsequently measured to determine the extent of NMDAR activation. As indicated in Table 1, the potency of the different metabolites varied significantly across different NMDAR subtypes, potentially indicating subtype-specific pharmacological targeting. As expected,^26^ the reference compound (+)-MK-801 showed the highest pIC50 values, confirming its high potency in antagonizing all the tested NMDAR subtypes. Overall, the pIC50 of the active compounds are of the same magnitude as those of (*S*)-**methadone**, indicating that they can be considered low potency NMDAR antagonists, as the first-in class candidate drug. At variance with (*S*)-methadone, two metabolites ((3*R*,6*R*)-**DNML** and (3*S*,6*S*)-**DNML**) showed a preferential activity on the GluN1-GluN2A subtype. Notably, (3*R*,6*S*)-**DNML** has a preferential effect on the GluN1-GluN2C and GluN1-GluN2D subtypes. Similar to ketamine, (3*R*,6*R*)-**NML** and (3*R*,6*R*)-**DNML** demonstrate relatively high potency across multiple subtypes, suggesting potential as effective NMDAR antagonists. (*R*)-**EDDP** displays selectivity and moderate potency against GluN1-GluN2C subtype, whereas (*R*)-**EMDP** shows high selectivity and potency against GluN1-GluN2A subtype. (*S*)-**DDVA**, (*S*)-**DDPO**, (*S*)-**EDDP**, and (*S*)-**EMDP** have pIC50 values below 4 for all four NMDAR subtypes suggesting that these compounds do not effectively inhibit the activity of these NMDAR subtypes.

**Table 1:** Mean pIC50 Values (with Standard Error of the Mean) of Methadone Metabolites on Specific NMDAR GluN1-GluN2 Subtypes Using FLIPR/Ca^2+^ Assay^a^

	**pIC50** (mean ± SEM)			
	**GluN1-GluN2A**	**GluN1-GluN2B**	**GluN1-GluN2C**	**GluN1-GluN2D**
(+)-**MK-801**	6.6 ± 0.035	7.2 ± 0.067	6.2 ± 0.064	6.1 ± 0.58
(±)-**ketamine**	4.6 ± 0.028	5.2 ± 0.037	5.4 ± 0.069	5.0 ± 0.087
(*R*)-**methadone**	4.7 ± 0.082	5.0 ± 0.037	5.1 ± 0.040	4.9 ± 0.040
(*S*)-**methadone**	4.4 ± 0.062	4.9 ± 0.071	5.2 ± 0.051	4.6 ± 0.041
**memantine**	4.5 ± 0.019	5.0 ± 0.032	5.5 ± 0.063	5.0 ± 0.14
**dextromethorphan**	4.3 ± 0.029	4.8 ± 0.037	5.2 ± 0.057	4.6 ± 0.12
(3*S*,6*R*)-**ML**	4.5 ± 0.18	n.d.	4.2 ± 0.074	4.2 ± 0.23
(3*R*,6*R*)-**ML**	4.5 ± 0.085	4.0 ± 0.12	4.1 ± 0.062	4.3 ± 0.12
(3*S*,6*S*)-**ML**	4.1 ± 0.034	4.0 ± 0.077	4.1 ± 0.056	4.2 ± 0.16
(3*R*,6*S*)-**ML**	4.0 ± 0.23	n.d.	n.d.	n.d.
(3*S*,6*R*)-**NML**	4.8 ± 0.050	4.6 ± 0.047	5.0 ± 0.049	5.0 ± 0.058
(3*R*,6*R*)-**NML**	5.0 ± 0.081	4.8 ± 0.055	5.0 ± 0.048	5.0 ± 0.052
(3*R*,6*S*)-**NML**	4.4 ± 0.037	4.5 ± 0.053	4.6 ± 0.046	4.7 ± 0.057
(3*S*,6*S*)-**NML**	4.6 ± 0.062	4.7 ± 0.080	4.7 ± 0.042	4.6 ± 0.065
(3*S*,6*R*)-**DNML**	4.5 ± 0.031	4.4 ± 0.042	4.6 ± 0.038	4.7 ± 0.061
(3*R*,6*R*)-**DNML**	4.8 ± 0.060	4.6 ± 0.054	4.7 ± 0.050	4.7 ± 0.058
(3*R*,6*S*)-**DNML**	4.6 ± 0.077	4.6 ± 0.062	5.1 ± 0.062	4.9 ± 0.093
(3*S*,6*S*)-**DNML**	4.9 ± 0.081	4.4 ± 0.13	4.7 ± 0.056	4.7 ± 0.079
(*R*)-**DDVA**	n.d.	n.d.	n.d.	n.d.
(*R*)-**DDPO**	n.d.	n.d.	n.d.	n.d.
(*R*)-**EDDP**	n.d.	n.d.	4.3 ± 0.056	4.3 ± 0.060
(*R*)-**EMDP**	4.8 ± 0.088	n.d.	n.d.	n.d.
(*S*)-**DDVA**	n.d.	n.d.	n.d.	n.d.
(*S*)-**DDPO**	n.d.	n.d.	n.d.	n.d.
(*S*)-**EDDP**	n.d.	n.d.	n.d.	n.d.
(*S*)-**EMDP**	4.1 ± 0.14	n.d.	n.d.	n.d.

a pIC50 values represent the inhibitory potency of the compounds on the different GluN1-GluN2 channel subtypes in CHO cells upon stimulation with 10 μM L-glutamate. n.d. indicate that 50% of inhibition could not be reached, nor at the highest concentration used.

### Radioligand Displacement Binding Assays

**Figure 2:**
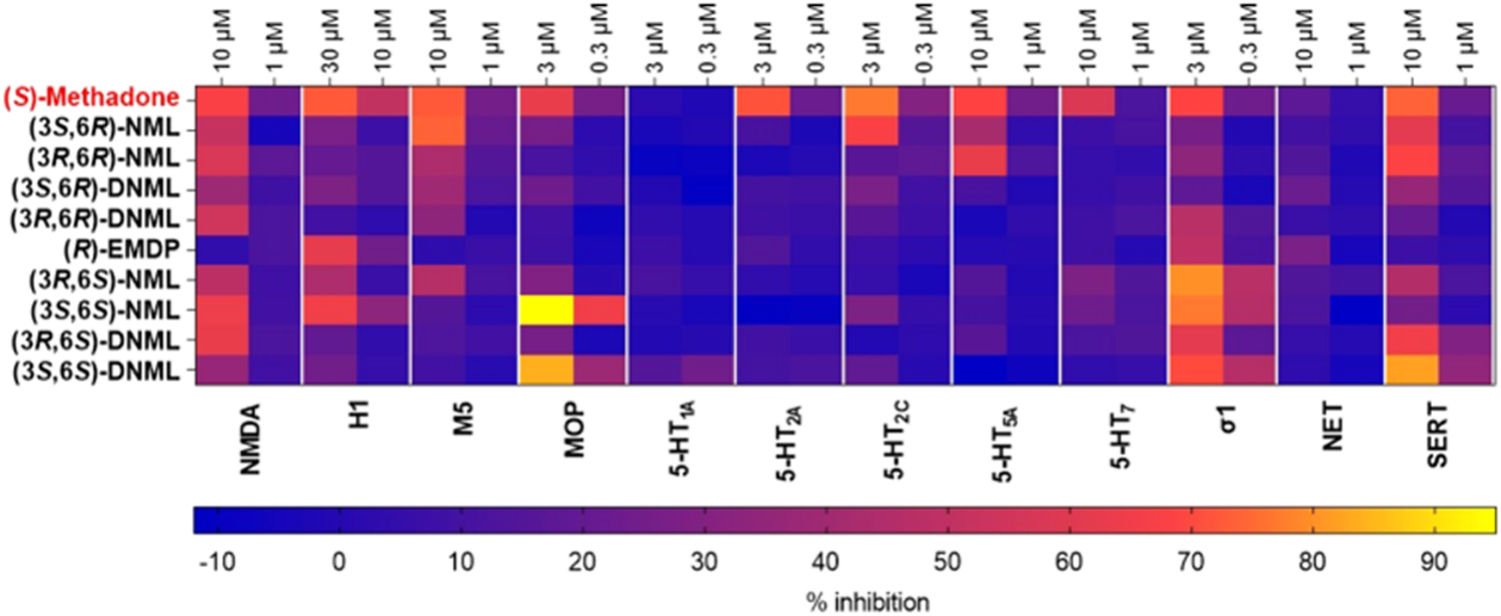
Heatmap of radioligand displacement binding assays screen at two concentrations of (S)-methadone and nine selected methadone metabolites on a panel of CNS receptors implicated in the psychopharmacology of MDD.

Among the synthesized compounds, 9 methadone metabolites, chosen based on their high potency as uncompetitive NMDAR antagonists in the FLIPR assay (Table 1), underwent evaluation, and comparison with (*S*)-methadone, at Eurofins Panlabs Discovery Services (Taiwan) through radioligand displacement binding assays targeting a range of CNS receptors and targets known to be potentially implicated in the neurobiology and psychopharmacology of depression, including NMDA, H1, M5, MOP, 5-HT1A, 5-HT2A, 5-HT2C, 5-HT5a, 5-HT7, sigma1, norepinephrine transporter (NET), and serotonin transporter (SERT). The heatmap in Figure 2 displays the % of inhibition of (*S*)-methadone and the tested metabolites against the different targets. A higher percentage of inhibition indicates stronger displacement ability of the tested compound for the pharmacological target. As expected, all the tested compounds, except for (*R*)-**EMDP**, caused displacement at the NMDAR at the highest concentration. In particular, (3*S*,6*S*)-**NML**, (3*R*,6*S*)-**DNML** have comparable displacement activity at the NMDAR as (*S*)-methadone. None of the tested molecules showed activity on the 5-HT1A receptor, whereas only (*S*)-methadone showed appreciable activity on 5-HT2A. Among all, (3*S*,6*R*)-**NML** is the one with the affinity trend most similar to (*S*)-methadone on the tested receptors. Notably, (3*S*,6*S*)-**NML** showed high displacement percentages at the MOP and Sigma-1 (σ1) receptors, and (3*S*,6*S*)-**NML** displayed significant displacement percentages at the histamine H1 receptor, like (*S*)-methadone and (*R*)-**EMDP**. The serotonin transporter (SERT) was displaced by (3*S*,6*S*)-**DNML**, (3*R*,6*S*)-**DNML**, and by (3*S*,6*R*)-**NML** and (3*R*,6*R*)-**NML** at 10 μM, suggesting the potential interaction at these pharmacological targets at a concentration where activity on NMDAR is present. (3*R*,6*S*)-**NML** may have interesting selective affinity for the σ1 receptor, whereas (*R*)-**EMDP** generally show moderate to low displacement across various receptors. Overall, it is worth noticing that *N*-demethylated-(6*S*)-methadols displayed a higher affinity trend for the MOP than the corresponding *N*-demethylated-(6*R*)-methadols, showing an opposite trend than (*R*)- and (*S*)-methadone, confirming what was previously reported for methadol acetylated analogs.^50^

To confirm the affinity trend and evaluate the relative affinity for NMDAR vs MOP, the binding affinities (p*K*_i_) of *N*-demethylated-(6*R*)-methadol metabolites were obtained and compared with those of (*S*)-methadone. The relative affinity was expressed by the NMDAR/MOP index calculated as the antilogarithm of the difference between p*K*_i_ values for NMDAR and p*K*_i_ values for MOP (see Table 2). Of note, (3*S*,6*R*)-**NML** and (3*R*,6*R*)-**NML** showed a greatly enhanced relative affinity toward NMDAR, with a NMDAR/MOP ratio of 0.98 and 0.83, respectively, compared to (*S*)-methadone NMDAR/MOP ratio of 0.17. This finding confirms that *N*-demethylated-(6*R*)-methadols have lower affinity for the MOP when compared to (*S*)-methadone, underlying the need for further studies to unravel the relationship between the molecular MOA of (*S*)-methadone and its derivatives and the antidepressant effects.

**Table 2:** MOP and NMDAR Binding Affinities (p*K*_i_) of (3*S*,6*R*)-**NML** and (3*R*,6*R*)-**NML** and (*S*)-Methadone

	**p***K*_**i**_**(mean ± SEM)**		
	**[3H]TCP, NMDA**	**[3H]diprenorphine, MOP**	**selectivity NMDAR/MOP**
(3*S*,6*R*)-**NML**	5.54 ± 0.09	5.55 ± 0.17	0.98
(3*R*,6*R*)-**NML**	5.56 ± 0.07	5.64 ± 0.09	0.83
(*S*)-**Methadone**	5.58 ± 0.07	6.35 ± 0.07	0.12

### Computational Chemistry Studies

**Figure 3:**
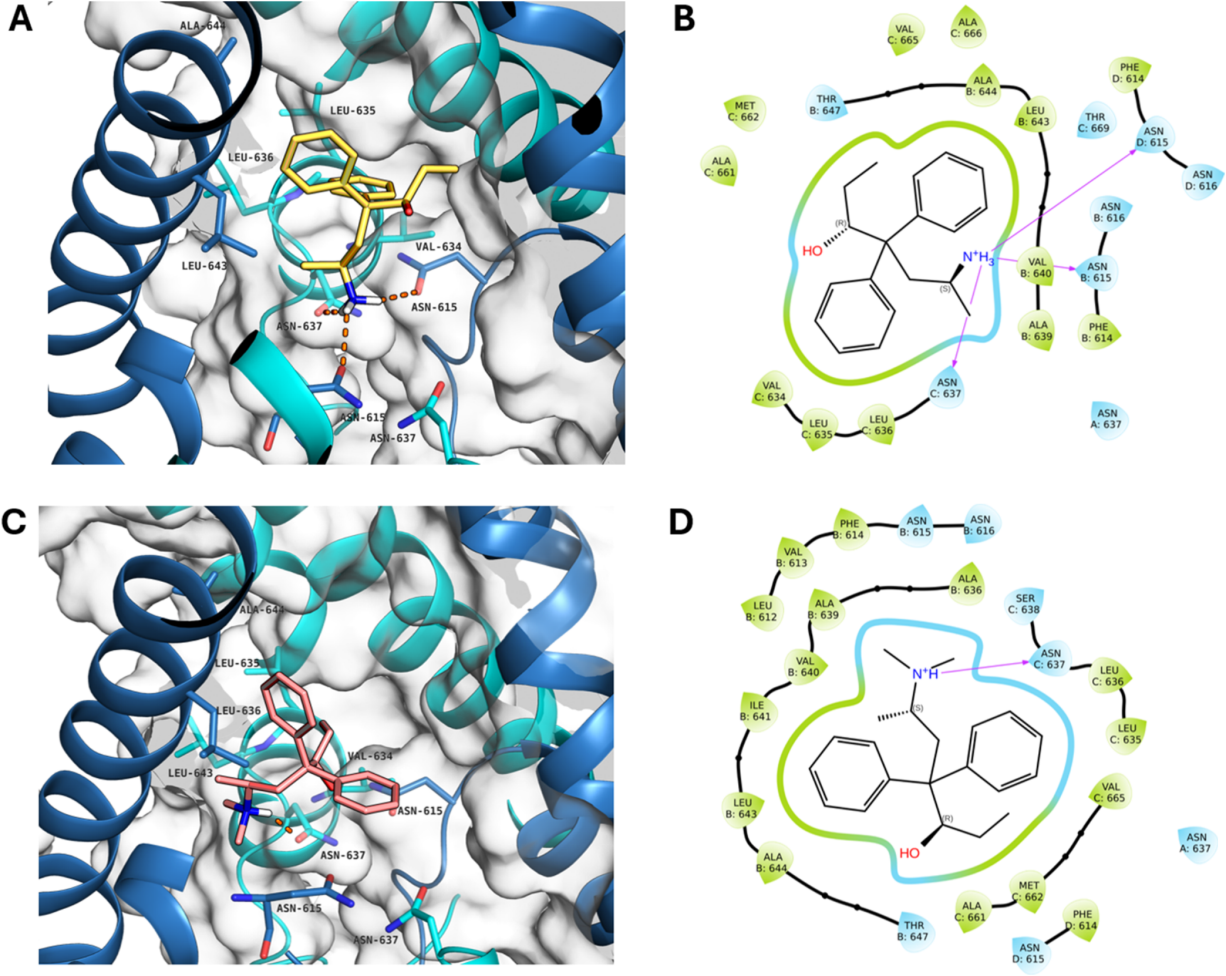
Best (3*R*,6*S*)-DNML (A, yellow) and (3*R*,6*S*)-ML (C, salmon pink) docking pose in GluN1-GluN2C homology model centering the grid on the asparagines 615 of the GluN2C subunits, hydrogen bonds are shown in orange. Best (3*R*,6*S*)-DNML (B) and (3*R*,6*S*)-ML (D) Schrödinger 2D ligand interactions visualization, hydrogen bonds are shown in purple, hydrophobic regions are shown in light green and hydrophilic regions are shown in light blue. The structure of the receptor was generated using homology modeling and the structure identified by the code 6WHT as template.

Aiming to rationalize the results of the inhibition assays, we performed docking calculations for all the synthesized compounds in all the tested NMDAR subtypes by adopting the models and the protocol already described in our previous work.^26^ Briefly, ligand docking calculations were carried out using Glide,^52,53^ centering the grid on the QRN site (specifically, the asparagine residues at position-615 in the template structures) and employing the extra precision (XP) mode. Multiple docking poses in the predicted binding site revealed the importance of the amine moiety for having efficient hydrogen bond interactions with the four asparagine residues (the two ASN 637 of the GluN1 chains A and C, two ASN 615 of the GluN2C chains B and D) on the putative binding site (**A** and **C**, Figure 3). Furthermore, it was observed that the substitution (methylation) level of the amine moiety may play a pivotal role in the number of possible hydrogen bonds. *In silico* data, together with *in vitro* binding assays results support the hypothesis that the higher number of available hydrogen bond donor of the primary amine (3 hydrogens in the protonated (*3R*,*6S*)-DNML, Figure 3) compared to the secondary and tertiary amines (1 hydrogen in (*3R*,*6S*)-**ML**, Figure 3) may indeed increase the binding efficiency.^54^ Furthermore, by directly comparing (*3R,6S*)-**DNML** and (*3R,6S*)-**ML** best poses neither shows explicit hydrophobic interactions (π–π stacking or π-cation) between the protein and the phenyl moieties of the ligands, however all the methadone metabolites tend to orient the phenyl groups toward the hydrophobic regions (light green in **B** and **D**, Figure 3) of the binding site formed by branched amino acid residues of GluN1 C subunit (VAL 634, LEU 635, 636) and the GluN2C B subunit (LEU 643, and ALA 644). It is also noticeable that other than the significant difference in number of hydrogen bonds, one of the phenyl groups of (*3R,6S*)-**ML** is clearly located in a less favorable hydrophilic environment (light blue in **D**, Figure 3) compared to both aromatic groups of (*3R,6S*)-**DNML** (light green in **B**, Figure 3).

## Conclusions

In the present study, we developed a novel chiral pool synthetic strategy, based on l-alanine or d-alanine-derived cyclic sulfamidates, to obtain enantiomerically pure (*R*)-methadone, (*S*)-methadone, and applied this approach for the stereodivergent synthesis of 20 enantiopure methadone human metabolites.

The pharmacodynamic evaluation of the synthesized compounds revealed that *N*-demethylated-(6*R*)-methadol metabolites, specifically (3*S*,6*R*)-**NML** and (3*R*,6*R*)-**NML**, maintain or even increase NMDAR antagonism compared with (*R*)-methadone, which is greater than that of (*S*)-methadone, while losing the undesired affinity for the MOP receptor. Therefore, this study provides the first in vitro evidence that *N*-demethylated-(6*R*)-methadols are novel NMDAR antagonists, exhibiting higher potency than (*S*)-methadone for this target and potentially devoid of opioid off-target effects. Future in vivo studies should validate the therapeutic potential of these compounds and fully elucidate their pharmacokinetic and pharmacodynamic profiles.

## Experimental Section

Reagents and solvents were high-purity commercial products and were used without additional purification. Reactions were monitored by thin-layer chromatography (TLC) using 40 × 80 mm plastic plates coated with a 200 μm layer of silica gel (60 Å pore size, F_254_, Merck). Plates were revealed by UV irradiation (λ = 254 nm) or KMnO_4_ staining. Flash column chromatography was performed using 220–440 mesh silica gel (60 Å pore size, Merck), aided by a positive nitrogen pressure. ^1^H and ^13^C nuclear magnetic resonance (NMR) spectra were acquired on a Bruker Avance III HD 400 spectrometer operating at 400 MHz for ^1^H and 101 MHz for ^13^C. Chemical shifts (δ) are given in ppm and coupling constants (*J*) in Hz. Spectra were calibrated with the residual solvent signal. MestReNova (v14.2.0–26256) software was used to process NMR spectra. To avoid redundancy, only the NMR data of the (*R*)-configured compounds at the nitrogen-bearing carbon are reported in the experimental section, spectra of all isomers are reported in the Supporting Information. High resolution mass spectrometry (HRMS) was performed in positive mode (ESI) on an Agilent 6550 IFunnel Q-TOF MS system, via flow-injection technique of a methanolic solution in a 1:1 water/ACN + 0.1% formic acid as eluent system. [α]_*D*_ was determined with a JASCO P2000 polarimeter equipped with sodium lamp (λ = 589 nm), measuring a solution in CHCl_3_ or EtOH at a concentration specified for each compound. The purity of final compounds was assessed via UPLC analysis on an Agilent 1290 Infinity system equipped with a DAD detector (λ = 190–400 nm) under the following conditions: column ZORBAX Eclipse XDB-C18 (2.1 × 50 mm, 1.8 μm) at 25 °C, mobile phase A water +0.1% TFA, mobile phase B ACN + 0.1% TFA, gradient starting from 5% B and reaching 100% B in 10 min, detection λ = 220 and 254 nm, injection volume 5 μL, compound solution 0.1–0.5 mM in DMSO or water/ACN, all tested compounds are >95% pure by UPLC analysis. Optical purity of the final compounds was determined by chiral HPLC using a Shimadzu SPD-10A system equipped with a REFLECT C-amylose A column (4.6 × 250 mm, 5 μM). Elution was performed in isocratic conditions using phase A hexane +0.1% DEA or acetic acid, phase B isopropanol or ethanol as specified for each compound, detection λ = 254 nm, all tested compounds are >95% optically pure by chiral HPLC analysis.

### Chemistry. (*R*)- or (*S*)-*tert*-Butyl (1-hydroxypropan-2-yl)carbamate (**1**)

To an ice-cooled solution of (*R*)- or (*S*)-**alaninol** (4.553 g, 60.63 mmol) in DCM (45 mL) were added TEA (9.245 mL, 66.69 mmol, 1.1 equiv) and ditertbutyl dicarbonate (14.558 g, 66.69 mmol, 1.1 equiv). The solution was stirred at room temperature for 2.5 h. The reaction was checked via TLC and, upon completion, the DCM was evaporated under reduced pressure. The residue was diluted with ethyl acetate (200 mL) and washed with aq. HCl 0.5 M/brine (200 mL + 50 mL). The aqueous phase was further extracted with ethyl acetate (5 × 50 mL). The organic fractions were collected, dried over anhydrous Na_2_SO_4_, and evaporated under reduced pressure. The crude product was purified via flash column chromatography using petroleum ether/acetone 75:25 to 5:5 as eluent system, obtaining (*R*)- or (*S*)-**1** as a white solid (9.349 g, 53.35 mmol, 88% yield). HRMS (ESI): *m*/*z* calculated for C_8_H_17_NO_3_ + Na^+^ [M + Na^+^]: 198.1101. Found: 198.1103. ^1^H NMR (400 MHz, CDCl_3_) δ 5.04 (d, *J* = 7.9 Hz, 1H), 3.82 (s, 1H), 3.62 (s, 1H), 3.48 (dd, *J* = 11.0, 4.2 Hz, 1H), 3.39 (dd, *J* = 11.0, 5.6 Hz, 1H), 1.34 (s, 9H), 1.04 (d, *J* = 6.8 Hz, 3H). ^13^C NMR (101 MHz, CDCl_3_) δ 156.35, 79.48, 66.49, 48.40, 28.47, 17.39. (*R*)-**1** [α]_*D*_^25^ + 8.94 (*c* = 1.5, CHCl_3_), (*S*)-**1** [α]_*D*_^25^ −8.56 (*c* = 1.5, CHCl_3_).

### (*R*)- or (*S*)-*tert*-Butyl 4-methyl-1,2,3-oxathiazolidine-3-carboxylate 2,2-dioxide (**2**)

To a stirred solution of imidazole (7.770 g, 114.14 mmol, 4 equiv) and triethylamine (9.493 mL, 68.48 mmol, 2.4 equiv) in DCM (85 mL), cooled at −40 °C under nitrogen atmosphere, was added dropwise a solution of SOCl_2_ (2.482 mL, 34.24 mmol, 1.2 equiv) in DCM (19 mL). The mixture was cooled to −60 °C, then a solution of (*R*)- or (*S*)-**1** (5.000 g, 28.53 mmol, 1 equiv) in DCM (104 mL) was added dropwise over 30 min and the mixture was left coming up to room temperature over 16 h. Upon completion, as confirmed by TLC analysis, the mixture was poured into a separatory funnel and washed 3 times with water/brine 1:1 (50 mL each). The organic fraction was collected, dehydrated over anhydrous Na_2_SO_4_ and evaporated under reduced pressure. The residual oil was reconstituted in ACN (55 mL) and water (30 mL). The solution was cooled to 0 °C and RuCl_3_ (30.0 mg, 0.14 mmol, 0.005 equiv) was added. Then, NaIO_4_ (6.713 g, 31.39 mmol, 1.1 equiv) was added portionwise. The biphasic mixture was vigorously stirred for 2.5 h at room temperature under nitrogen atmosphere. The mixture was diluted in DCM (100 mL) and washed with water (100 mL). The aqueous phase was further extracted with DCM (3 × 50 mL). The organic fractions were collected, dehydrated over anhydrous Na_2_SO_4_ and evaporated under reduced pressure. The crude product was dissolved in IPAc at 45 °C (30 mL), then *n*-heptane (150 mL) was added with gentle stirring over 1 h. The obtained slurry was stirred for 2 h at room temperature. Finally, it was cooled to −18 °C for 16 h. The solids were filtered, washed with cold *n*-heptane (50 mL) and dried *in vacuo*, obtaining (*R*)- or (*S*)-**2** as a white crystalline solid (5.824 g, 24.55 mmol, 86% yield). HRMS (ESI): *m*/*z* calculated for C_8_H_15_NO_5_S + Na^+^ [M + Na^+^]: 260.0563. Found: 260.0570. ^1^H NMR (400 MHz, CDCl_3_) δ 4.65 (dd, *J* = 9.1, 5.9 Hz, 1H), 4.40 (m, 1H), 4.19 (dd, *J* = 9.1, 2.9 Hz, 1H), 1.54 (s, 9H), 1.49 (d, *J* = 6.4 Hz, 3H). ^13^C NMR (101 MHz, CDCl_3_) δ 148.57, 85.46, 71.46, 53.94, 28.04, 18.41. (*R*)-**2** [α]_*D*_^25^ −5.29 (*c* = 1.5, CHCl_3_), (*S*)-**2** [α]_*D*_^25^ + 5.40 (*c* = 1.5, CHCl_3_).

### (*R*)- or (*S*)-*tert*-Butyl (4-cyano-4,4-diphenylbutan-2-yl)carbamate (**3**)

To a stirred solution of (*R*)- or (*S*)-**2** (5.000 g, 21.07 mmol, 1 equiv) and Ph_2_CHCN (4.479 g, 23.18 mmol, 1.1 equiv) in anhydrous THF (57 mL), cooled at −20 °C, was added dropwise under nitrogen atmosphere NaHMDS (1 M in THF, 25.29 mL, 25.29 mmol, 1.2 equiv). The mixture was stirred at −20 °C for 1 h. At completion, the reaction mixture was warmed to 0 °C and quenched with 5% citric acid (w/v) (aq.) (25 mL). After stirring for 10 min, most of THF was removed under reduced pressure, then the residue was transferred to a separatory funnel with other 5% citric acid solution (25 mL) and extracted with DCM (100 mL). The aqueous phase was further extracted with DCM (3 × 50 mL). The organic fractions were collected, dehydrated over anhydrous Na_2_SO_4_ and evaporated under reduced pressure. The crude product was redissolved in THF (7 mL), then *n*-heptane (100 mL) was added dropwise over 1 h, with gentle stirring. The solution was cooled to 0 °C, seeded with the target compound, and the resulting slurry was stirred for other 2 h at 0 °C. After aging for 16 h at −18 °C, the solids were filtered, washed with cold *n*-heptane (50 mL) and dried *in vacuo*, obtaining (*R*)- or (*S*)-**3** as a white crystalline solid (6.576 g, 18.79 mmol, 90% yield). HRMS (ESI): *m*/*z* calculated for C_22_H_26_N_2_O_2_ + H^+^ [M + H^+^]: 351.2067. Found: 351.2081. ^1^H NMR (400 MHz, CDCl_3_) δ 7.47–7.25 (m, 10H), 4.37 (s, 1H), 3.76–3.63 (m, 1H), 2.80–2.66 (m, 1H), 2.44 (dd, *J* = 14.2, 5.3 Hz, 1H), 1.40 (s, 9H), 1.19 (d, *J* = 6.6 Hz, 3H). ^13^C NMR (101 MHz, CDCl_3_) δ 155.91, 139.66, 138.82, 129.27, 129.09, 128.01, 127.80, 126.79, 126.60, 120.73, 79.45, 57.09, 43.80, 39.23, 28.53, 14.70. (*R*)-**3** [α]_*D*_^25^ −2.21 (*c* = 1.5, CHCl_3_), (*S*)-**3** [α]_*D*_^25^ + 2.11 (*c* = 1.5, CHCl_3_).

### (*R*)- or (*S*)-4-(Dimethylamino)-2,2-diphenylpentanenitrile (**4**)

To an ice-cooled solution of (*R*)- or (*S*)-**3** (1.250 g, 3.57 mmol, 1 equiv) in MeOH (12 mL) was added HCl (3 M in MeOH, 12.5 mL, 37.5 mmol, 10.5 equiv) dropwise under nitrogen atmosphere and the mixture was stirred at 0 °C for 4 h. At reaction completion, solvents and residual HCl were removed by evaporation under reduced pressure to obtain the primary amine hydrochloride as light-yellow foam. The residue was redissolved in ACN (16 mL) and water (3 mL), cooled in ice bath, and to this solution formaldehyde (37% w/v in water, 2.66 mL, 35.70 mmol, 10 equiv) was added. Then, NaBH(OAc)_3_ (2.421 g, 11.42 mmol, 3.2 equiv) was added portionwise. The reaction mixture was stirred at room temperature for 1 h. Then, the solution was poured into sat. aq. NaHCO_3_ (80 mL) and extracted with DCM (3 × 100 mL). The collected organic fractions were dried over anhydrous Na_2_SO_4_ and evaporated under reduced pressure. The crude product was purified by flash column chromatography using DCM/EA 8:2 + 1% NH_3_ 7 M in MeOH as eluent system to obtain (*R*)- or (*S*)-**4**, as a white solid (0.954 g, 3.43 mmol, 96% yield). HRMS (ESI): *m*/*z* calculated for C_19_H_22_N_2_ + H^+^ [M + H^+^]: 279.1856. Found: 279.1862. ^1^H NMR (400 MHz, CDCl_3_) δ 7.48–7.24 (m, 10H), 2.68 (dd, *J* = 13.8, 6.5 Hz, 1H), 2.53 (h, *J* = 6.5 Hz, 1H), 2.23 (dd, *J* = 13.9, 6.0 Hz, 1H), 2.13 (s, 6H), 0.91 (d, *J* = 6.6 Hz, 3H). ^13^C NMR (101 MHz, CDCl_3_) δ 141.34, 140.75, 128.85, 128.77, 127.89, 127.74, 127.46, 127.28, 122.93, 55.56, 49.70, 43.33, 40.02, 13.16. (*R*)-**4** [α]_*D*_^25^ −49.83 (*c* = 0.6, EtOH), (*S*)-**4** [α]_*D*_^25^ + 48.10 (*c* = 0.6, EtOH).

### (*R*)- or (*S*)-4-(Dimethylamino)-2,2-diphenylpentanal (**5**)

To a suspension of (*R*)- or (*S*)-**4** (1.000 g, 3.59 mmol, 1 equiv) in anhydrous toluene (10 mL), stirred under nitrogen atmosphere in an oven-dried vial, 3-pentylmagnesium bromide (2.0 M in diethyl ether, 5 mL, 10.00 mmol, 2.8 equiv) was added dropwise. A gentle nitrogen flow was applied with cautious heating (40–50 °C) to distill off the ether. After that, the reaction was sealed and heated to 100 °C for 3.5 h. When the starting material was consumed, as seen by TLC analysis, the reaction mixture was cooled to 0 °C and quenched by carefully adding aq. HCl (6 M, 10 mL). The reaction was then vigorously stirred at room temperature for 16 h. The reaction mixture was poured into aq. 10% Na_2_CO_3_ (200 mL) and extracted with ethyl acetate (3 × 100 mL). The combined organic fractions were dried over anhydrous Na_2_SO_4_. After removal of the solvents by reduced pressure evaporation, the crude oil was purified by silica gel chromatography using CHCl_3_/MeOH 9:1 as mobile phase to obtain (*R*)- or (*S*)-**5** as a colorless oil (0.990 g, 3.52 mmol, 98% yield). HRMS (ESI): *m*/*z* calculated for C_19_H_23_N_2_O + H^+^ [M + H^+^]: 282.1852. Found: 282.1858. ^1^H NMR (400 MHz, CDCl_3_) δ 9.29 (s, 1H), 7.39–7.16 (m, 10H), 2.79 (dd, *J* = 13.8, 11.2 Hz, 1H), 2.48–2.38 (m, 1H), 2.18 (s, 6H), 2.05 (dd, *J* = 13.8, 3.4 Hz, 1H), 0.85 (d, *J* = 6.6 Hz, 3H). ^13^C NMR (101 MHz, CDCl_3_) δ 191.60, 142.54, 141.48, 129.64, 129.22, 128.58, 127.99, 127.29, 126.56, 60.44, 55.21, 42.95, 40.37, 11.59. (*R*)-**5** [α]_*D*_^25^ −20.36 (*c* = 1.5, CHCl_3_), (*S*)-**5** [α]_*D*_^25^ + 19.73 (*c* = 1.5, CHCl_3_).

### (*R*)- or (*S*)-4-(Dimethylamino)-2,2-diphenylpentanoic acid (DDVA)

To an ice-cooled solution of (*R*)- or (*S*)-**5** (1.200 g, 4.26 mmol, 1 equiv) in 1:1 MeCN/H_2_O (43 mL) were added in order NaH_2_PO_4_·2H_2_O (1.329 g, 8.52 mmol, 2.0 equiv), H_2_O_2_ (30% w/v, 0.870 mL, 8.52 mmol, 2.0 equiv), and NaClO_2_·3H_2_O (0.923 g, 6.39 mmol, 1.5 equiv). The reaction mixture was stirred at room temperature until completion as determined by TLC (4 h). The residual oxidants were quenched with excess solid Na_2_SO_3_. Then, the mixture was diluted with brine (50 mL) and extracted with ethyl acetate (3 × 50 mL). The combined organic fractions were dried over Na_2_SO_4_ and evaporated under reduced pressure. The crude product was purified by silica gel flash chromatography using CHCl_3_/MeOH 9:1 as eluent system, obtaining (*R*)- or (*S*)-**DDVA** as a white solid (1.051 g, 3.54 mmol, 83% yield). HRMS (ESI): *m*/*z* calculated for C_19_H_23_NO_2_ + H^+^ [M + H^+^]: 298.1802 Found: 298.1812. ^1^H NMR (400 MHz, CDCl_3_) δ 7.40–7.10 (m, 10H), 3.01 (dd, *J* = 15.4, 9.7 Hz, 1H), 2.71–2.61 (m, 1H), 2.39 (s, 6H), 2.07 (dd, *J* = 15.4, 1.4 Hz, 1H), 0.99 (d, *J* = 6.9 Hz, 3H). ^13^C NMR (101 MHz, CDCl_3_) δ 177.07, 147.51, 143.04, 129.06, 128.63, 128.30, 127.52, 126.85, 126.02, 63.11, 57.44, 41.68, 34.44, 13.56. (*R*)-**DDVA** [α]_*D*_^25^ −102.05 (*c* = 0.6, EtOH). UPLC purity >99%, *t*_R_ = 3.19 min. Chiral HPLC (hexane +0.1% AcOH/IPA 4:6) *t*_R_ = 7.1 min, e.e. > 99%. (*S*)-**DDVA** [α]_*D*_^25^ + 112.6 (*c* = 0.6, EtOH). UPLC purity >99%. Chiral HPLC *t*_R_ = 8.4 min, e.e. > 99%.

### (*R*)- or (*S*)-6-(Dimethylamino)-4,4-diphenylheptan-3-one ((*R*)- or (*S*)-**methadone**)

(*R*)- or (*S*)-**4** (0.300 g, 1.08 mmol, 1.0 equiv) was dissolved in anhydrous toluene (1.5 mL) and ethyl magnesium bromide (3.0 M in diethyl ether, 0.75 mL, 2.2 mmol, 2.1 equiv) was added dropwise under nitrogen atmosphere. After the Grignard reagent was added, the reaction mixture was slowly heated to 40–50 °C until all the ether was distilled off, aided by a gentle nitrogen flow. The reaction was then sealed and heated to 100 °C for 4 h. At completion, the reaction was cooled in ice bath, carefully quenched by dropwise addition of 6 M aq. HCl (3 mL), and vigorously stirred at 50 °C for 16 h. The reaction mixture was then cooled to room temperature and partitioned between 10% aq. Na_2_CO_3_ (300 mL) and ethyl acetate (100 mL). The aqueous phase was further extracted with ethyl acetate (3 × 50 mL). The organic fractions were collected, dehydrated with Na_2_SO_4_ and evaporated under reduced pressure. The crude product was purified by silica gel chromatography using CHCl_3_/MeOH 95:5 to 8:2 as eluent system, obtaining (*R*)- or (*S*)-**methadone** as a white solid (0.312 g, 1.01 mmol, 93% yield). HRMS (ESI): *m*/*z* calculated for C_21_H_27_NO + H^+^ [M + H^+^]: 310.2165 Found: 310.2171. ^1^H NMR (400 MHz, CDCl_3_) δ 7.42–7.20 (m, 10H), 2.86 (dd, *J* = 13.9, 5.4 Hz, 1H), 2.45–2.23 (m, 3H), 2.12 (s, 6H), 2.00 (dd, *J* = 13.9, 5.7 Hz, 1H), 0.83 (t, *J* = 7.2 Hz, 3H), 0.47 (d, *J* = 6.6 Hz, 3H). ^13^C NMR (101 MHz, CDCl_3_) δ 210.35, 142.93, 142.39, 130.20, 129.31, 128.27, 127.96, 126.98, 126.67, 65.21, 55.25, 43.24, 40.30, 32.20, 12.53, 9.48. (*R*)-**methadone** [α]_*D*_^25^ −27.47 (*c* = 1.0, EtOH). UPLC purity >99%, *t*_R_ = 4.0 min. Chiral HPLC (hexane +0.1% DEA/IPA 99:1) *t*_R_ = 4.1 min, e.e. > 99%. (*S*)-**methadone** [α]_*D*_^25^ + 28.56 (*c* = 1.0, EtOH). UPLC purity >99%. Chiral HPLC *t*_R_ = 4.3 min, e.e. > 99%.

### (*R*)- or (*S*)-1,5-Dimethyl-3,3-diphenylpyrrolidin-2-one (**DDPO**)

To an ice-cooled suspension of (*R*)- or (*S*)-**DDVA** (0.872 g, 2.93 mmol, 1 equiv) in CHCl_3_ (5 mL), stirred under nitrogen atmosphere, was added DMF (50 μL, catalytic). To that mixture was added dropwise a solution of SOCl_2_ (0.523 mL, 7.03 mmol, 2.4 equiv) in CHCl_3_ (2 mL). The reaction mixture was stirred at 70 °C for 4 h. Then, the solvent and excess SOCl_2_ were removed under reduced pressure. The residue was dissolved in DCM (50 mL) and washed with sat. aq. NaHCO_3_ (50 mL). The aqueous phase was further extracted with DCM (4 × 20 mL). The organic fractions were collected, dehydrated over Na_2_SO_4_, and evaporated under reduced pressure. The crude product was purified by flash chromatography using DCM/Acetone 95:5 as eluent system, obtaining (*R*)- or (*S*)-**DDPO** as an off-white crystalline solid (0.715 g, 2.70 mmol, 92% yield). HRMS (ESI): *m*/*z* calculated for C_18_H_19_NO + H^+^ [M + H^+^]: 266.1539 Found: 266.1545. ^1^H NMR (400 MHz, CDCl_3_) δ 7.39–7.17 (m, 10H), 3.54 (m, 1H), 3.00 (dd, *J* = 12.9, 5.9 Hz, 1H), 2.90 (s, 3H), 2.24 (dd, *J* = 13.0, 8.7 Hz, 1H), 1.28 (d, *J* = 6.2 Hz, 3H). ^13^C NMR (101 MHz, CDCl_3_) δ 175.39, 144.76, 142.34, 128.51, 128.26, 128.10, 127.87, 127.03, 126.62, 57.83, 52.03, 43.85, 27.87, 19.73. (*R*)-**DDPO** [α]_*D*_^25^ −10.55 (*c* = 0.6, EtOH). UPLC purity >99%, *t*_R_ = 5.33 min. Chiral HPLC (hexane +0.1% DEA/IPA 95:5) *t*_R_ = 11.7 min, e.e. > 99%. (*S*)-**DDPO** [α]_*D*_^25^ + 10.23 (*c* = 0.6, EtOH). UPLC purity >99%. Chiral HPLC *t*_R_ = 10.6 min, e.e. > 99%.

### (*R*)- or (*S*)-2-Ethyl-1,5-dimethyl-3,3-diphenyl-3,4-dihydro-5H-pyrrolium chloride (**EDDP**)

To a stirred suspension of (*R*)- or (*S*)-**DDPO** (0.300 g, 1.13 mmol, 1 equiv) in anhydrous Et_2_O (11 mL) was added EtLi (0.5 M in benzene/cyclohexane, 6.800 mL, 3.40 mmol, 3.0 equiv) under nitrogen atmosphere. The reaction mixture was stirred at rt for 3 h. The reaction was checked by TLC analysis, and, when complete, 1 mL of water was added to quench the residual EtLi. The organic phase was diluted with EA (40 mL) and washed once with sat. NaHCO_3_ (40 mL), dried over Na_2_SO_4_, and evaporated under reduced pressure. The crude was purified by column chromatography using CHCl_3_/MeOH 9:1 + 1% NH_4_OH as eluent system, obtaining (*R*)- or (*S*)-**EDDP**, as the 2-ethylidenepyrrolidine form (reddish oil). For stability reasons, the product was converted into the hydrochloride salt (2-ethyl-3,4-dihydro-5H-pyrrolium) by dissolving it in dioxane (2 mL) and treating the resulting solution with 4 M HCl in dioxane (0.420 mL, 1.5 equiv). After evaporating the dioxane and the excess HCl, (*R*)- or (*S*)-**EDDP** hydrochloride was obtained as a reddish gum (0.326 g, 1.04 mmol, 92% yield). HRMS (ESI): *m*/*z* calculated for C_19_H_24_N + H^+^ [M + H^+^]: 278.1903 Found: 278.1908. ^1^H NMR (400 MHz, CDCl_3_) δ 7.43–7.27 (m, 8H), 7.21–7.13 (m, 2H), 4.99 (h, *J* = 6.8 Hz, 1H), 3.96 (s, 3H), 3.37 (dd, *J* = 13.9, 8.1 Hz, 1H), 3.07–2.95 (m, 1H), 2.97–2.84 (m, 1H), 2.54 (dd, *J* = 13.9, 6.4 Hz, 1H), 1.53 (d, *J* = 6.6 Hz, 3H), 0.63 (t, *J* = 7.6 Hz, 3H). ^13^C NMR (101 MHz, CDCl_3_) δ 194.43, 141.53, 138.95, 129.48, 129.47, 128.90, 128.70, 128.49, 128.18, 68.32, 67.17, 45.43, 37.86, 24.85, 18.68, 11.11. (*R*)-EDDP*HCl [α]_*D*_^25^ + 63.20 (*c* = 1.0, EtOH). UPLC purity 98.9%, *t*_R_ = 3.58 min. (*S*)-EDDP*HCl [α]_*D*_^25^ −62.43 (*c* = 1.0, EtOH). UPLC purity 96.4%. Chiral HPLC separation of the two enantiomers was not achieved for this metabolite.

### (*R*)- or (*S*)-N-(4-Cyano-4,4-diphenylbutan-2-yl)benzamide (**6**)

To an ice-cooled solution of (*R*)- or (*S*)-**3** (0.600 g, 1.71 mmol, 1.0 equiv) in DCM (6 mL) was added dropwise HCl in dioxane (4 M, 24.0 mmol, 6.00 mL, 14.0 equiv). The reaction mixture was stirred at 0 °C for 3 h and checked by TLC analysis. At completion, the solvents and residual HCl were removed under reduced pressure. The crude product was redissolved in DCM (10 mL) and cooled to 0 °C. Then, a solution of TEA (0.950 mL, 6.85 mmol, 4 equiv) in DCM (3 mL) and a solution of benzoyl chloride (0.260 mL, 2.23 mmol, 1.3 equiv) in DCM (4 mL) were added dropwise in order. The mixture was stirred at 0 °C for 1 h. At completion, as seen by TLC, the mixture was diluted with DCM (100 mL) and washed with aq. 0.5 M HCl (100 mL). The aqueous phase was further extracted with DCM (70 mL × 3). The collected organic fractions were dehydrated over Na_2_SO_4_, dried under reduced pressure and the crude product was purified by silica gel chromatography using PE/EA 7:3 as eluent system, obtaining (*R*)- or (*S*)-**6** as a white vitreous solid (0.596 g, 1.68 mmol, 98% yield). HRMS (ESI): *m*/*z* calculated for C_24_H_22_N_2_O + H^+^ [M + H^+^]: 355.1805 Found: 355.1818. ^1^H NMR (400 MHz, CDCl_3_) δ 7.69–7.62 (m, 2H), 7.51–7.41 (m, 3H), 7.41–7.20 (m, 10H), 6.04 (d, *J* = 8.1 Hz, 1H), 4.28–4.13 (m, 1H), 2.93 (dd, *J* = 14.4, 8.8 Hz, 1H), 2.60 (dd, *J* = 14.4, 4.7 Hz, 1H), 1.34 (d, *J* = 6.6 Hz, 3H). ^13^C NMR (101 MHz, CDCl_3_) δ 166.72, 140.06, 139.79, 134.54, 131.48, 129.19, 129.18, 128.53, 128.25, 128.23, 127.06, 127.02, 126.93, 122.96, 49.38, 45.17, 44.16, 21.99. (*R*)-**6** [α]_*D*_^25^ −44.27 (*c* = 1.5, CHCl_3_), (*S*)-**6** [α]_*D*_^25^ + 41.90 (*c* = 1.5, CHCl_3_).

### (*R*)- or (*S*)-N-(5-Oxo-4,4-diphenylheptan-2-yl)benzamide (**7**)

(*R*)- or (*S*)-**6** (0.500 g, 1.41 mmol, 1 equiv) was put in an oven-dried vial with anhydrous toluene (2 mL). Ethyl magnesium bromide (3.0 M in diethyl ether, 1.41 mL, 4.23 mmol, 3.0 equiv) was added dropwise under nitrogen. After that, the reaction mixture was carefully heated to (40–50 °C) until all the diethyl ether was all distilled off, aided by a slow nitrogen flow. The reaction was then sealed and heated to 100 °C for 4 h. When all the starting material was converted to the corresponding ketimine, the reaction mixture was cooled to 0 °C and charged with 10% aq. AcOH (4 mL). The biphasic mixture was stirred vigorously at 70 °C for 16 h. Upon completion, the reaction mixture was cooled to rt and neutralized by addition into a solution of 100 mL of saturated aq. NaHCO_3_, which was extracted with DCM (100 mL × 3). The organic fractions were collected, dehydrated over Na_2_SO_4_, evaporated under reduced pressure and the crude product was purified by column chromatography using PE/EA 75:25 as eluent system obtaining (*R*)- or (*S*)-**7** as a white solid (0.412 g, 1.07 mmol, 76% yield). HRMS (ESI): *m*/*z* calculated for C_26_H_27_NO_2_ + H^+^ [M + H^+^]: 386.2115 Found: 386.2126. ^1^H NMR (400 MHz, CDCl_3_) δ 7.68–7.60 (m, 2H), 7.51–7.23 (m, 11H), 7.13–7.04 (m, 2H), 6.87 (d, *J* = 6.8 Hz, 1H), 3.34–3.19 (m, 1H), 3.10 (dd, *J* = 14.5, 11.2 Hz, 1H), 2.56–2.42 (m, 1H), 2.22–2.08 (m, 2H), 1.19 (d, *J* = 6.2 Hz, 3H), 0.84 (t, *J* = 7.3 Hz, 3H). ^13^C NMR (101 MHz, CDCl_3_) δ 214.82, 165.63, 142.32, 140.09, 134.76, 131.12, 129.14, 129.02, 128.83, 128.75, 128.46, 127.70, 127.58, 126.89, 66.56, 44.09, 43.70, 33.64, 22.58, 9.62. (*R*)-**7** [α]_*D*_^25^ −171.68 (*c* = 1.5, CHCl_3_), (*S*)-**7** [α]_*D*_^25^ + 173.24 (*c* = 1.5, CHCl_3_).

### (*R*)- or (*S*)-5-Ethyl-2-methyl-4,4-diphenyl-3,4-dihydro-2H-pyrrole (**EMDP**)

To a solution of (*R*)- or (*S*)-**7** (0.050 g, 0.13 mmol, 1.0 equiv) in dioxane (0.360 mL) was added aq. HCl (6 M, 0.360 mL). The reaction mixture was stirred at 100 °C for 2 h. At completion, the reaction mixture was poured into 10% aq. Na_2_CO_3_ solution (15 mL) which was extracted with DCM (3 × 10 mL). The organic fractions were collected, dehydrated over Na_2_SO_4_, and evaporated under reduced pressure. The crude product was purified by column chromatography using 2% acetone in DCM as eluent system, obtaining (*R*)- or (*S*)-**EMDP** as a white solid (0.029 g, 0.11 mmol, 85% yield). HRMS (ESI): *m*/*z* calculated for C_19_H_21_N + H^+^ [M + H^+^]: 264.1747 Found: 264.1755. ^1^H NMR (400 MHz, CDCl_3_) δ 7.38–7.12 (m, 10H), 4.00–3.87 (m, 1H), 2.71 (dd, *J* = 13.0, 6.3 Hz, 1H), 2.25 (dd, *J* = 13.0, 8.7 Hz, 1H), 2.22–2.11 (m, 1H), 2.11–1.99 (m, 1H), 1.41 (d, *J* = 6.7 Hz, 3H), 1.08 (t, *J* = 7.3 Hz, 3H). ^13^C NMR (101 MHz, CDCl_3_) δ 180.15, 145.00, 143.75, 128.48, 128.35, 128.19, 126.78, 126.59, 69.37, 64.50, 50.71, 25.02, 21.82, 11.18. (*R*)-**EMDP** [α]_*D*_^25^ −33.61 (*c* = 0.9, EtOH). UPLC purity 98.4%, *t*_R_ = 3.66 min. Chiral HPLC (hexane +0.1% DEA/IPA 97:3) *t*_R_ = 4.3 min, e.e. > 99%. (*S*)-**EMDP** [α]_*D*_^25^ + 31.49 (*c* = 1.5, EtOH). UPLC purity 98.4%. Chiral HPLC *t*_R_ = 3.6 min, e.e. > 99%.

### 6-(Benzamido)-4,4-diphenylheptan-3-ol (**8**)

(*R*)- or (*S*)-**7** (1.080 g, 2.80 mmol, 1 equiv) was dissolved in anhydrous THF (1 mL) and cooled to 0 °C under inert atmosphere. Lithium aluminum hydride (1 M in THF, 5.600 mL, 5.60 mmol, 2 equiv) was added dropwise and the mixture was stirred at 0 °C for 1 h. At completion, as seen by TLC analysis, the reaction was quenched with half-saturated aq. Rochelle’s salt (60 mL), and the mixture vigorously stirred for 15 min. After that, it was poured into a separatory funnel with DCM (100 mL) and extracted. The aqueous phase was further extracted with DCM (3 × 50 mL), then the combined organics were dried over Na_2_SO_4_, and evaporated under reduced pressure. The two diastereomers were isolated by silica gel column chromatography using DCM/EA 9:1 to 6:4 as eluent system, obtaining the α-stereoisomer ((3*R*,6*R*)-**8** or (3*S*,6*S*)-**8** when the starting material was (*R*)- or (*S*)-**7**, respectively) as a colorless foam, and the β-stereoisomer ((3*S*,6*R*)-**8** or (3*R*,6*S*)-**8** when the starting material was (*R*)- or (*S*)-**7**, respectively) as a white solid (1.043 g, 2.69 mmol, 96% yield, 18% α-isomer diastereomeric excess). *α-Stereoisomers* ((3*R*,6*R*)-**8** or (3*S*,6*S*)-**8**): HRMS (ESI): *m*/*z* calculated for C_26_H_29_NO_2_ + H^+^ [M + H^+^]: 388.2271 Found: 388.2282. ^1^H NMR (400 MHz, CDCl_3_) δ 7.46–7.37 (m, 1H), 7.37–7.18 (m, 12H), 7.18–7.12 (m, 1H), 7.11–7.03 (m, 1H), 5.35 (d, *J* = 7.4 Hz, 1H), 4.43–4.33 (m, 1H), 4.26–4.12 (m, 1H), 2.66 (dd, *J* = 14.7, 3.0 Hz, 1H), 2.32 (dd, *J* = 14.6, 8.6 Hz, 1H), 1.79–1.59 (m, 2H), 1.12 (d, *J* = 6.5 Hz, 3H), 0.97 (t, *J* = 7.3 Hz, 3H), 0.73–0.57 (m, 1H). ^13^C NMR (101 MHz, CDCl_3_) δ 166.27, 143.98, 134.62, 131.20, 129.65, 129.29, 128.22, 128.07, 128.06, 126.81, 126.67, 126.57, 76.45, 55.56, 45.72, 43.63, 26.68, 23.47, 11.44. ((3*R*,6*R*)-**8**) [α]_*D*_^25^ −38.10 (*c* = 1.5, CHCl_3_). Chiral HPLC (hexane +0.1% DEA/IPA 8:2) *t*_R_ = 8.1 min, e.e. > 99%. ((3*S*,6*S*)-**8**) [α]_*D*_^25^ + 37.03 (*c* = 1.5, CHCl_3_). Chiral HPLC (hexane +0.1% DEA/IPA 9:1) *t*_R_ = 7.5 min, e.e. > 99%. *β-stereoisomers* ((3*S*,6*R*)-**8** or (3*R*,6*S*)-**8**): HRMS (ESI): *m*/*z* calculated for C_26_H_29_NO_2_ + H^+^ [M + H^+^]: 388.2271 Found: 388.2278. ^1^H NMR (400 MHz, CDCl_3_) δ 7.78–7.71 (m, 2H), 7.53–7.45 (m, 1H), 7.44–7.36 (m, 2H), 7.36–7.11 (m, 10H), 6.42 (d, *J* = 6.5 Hz, 1H), 4.62–4.52 (m, 1H), 3.98–3.85 (m, 1H), 3.11–3.00 (m, 2H), 2.07–1.95 (m, 1H), 1.75–1.62 (m, 1H), 1.02 (t, *J* = 7.3 Hz, 3H), 0.78 (d, *J* = 6.4 Hz, 3H), 0.69–0.53 (m, 1H). ^13^C NMR (101 MHz, CDCl_3_) δ 167.05, 144.99, 144.34, 134.62, 131.53, 129.72, 129.40, 128.59, 128.19, 127.54, 127.04, 126.55, 126.47, 74.26, 55.45, 45.97, 43.71, 27.17, 22.41, 11.67. ((3*S*,6*R*)-**8**) [α]_*D*_^25^ + 1.47 (*c* = 1.5, CHCl_3_). Chiral HPLC (hexane +0.1% DEA/IPA 8:2) *t*_R_ = 3.2 min, e.e. >99%. ((3*R*,6*S*)-**8**) [α]_*D*_^25^ −1.77 (*c* = 1.5, CHCl_3_). Chiral HPLC (hexane +0.1% DEA/IPA 9:1) *t*_R_ = 6.7 min, e.e. > 99%.

#### General Procedure for the Synthesis of N-Benzyl-Protected DNMs (**9**)

To an ice-cooled solution of **8** (1.0 equiv) in anhydrous THF (1.25 mL/mmol) was added lithium aluminum hydride (1 M in THF, 8.0 equiv) dropwise under inert atmosphere, and the mixture was refluxed for 70 h. After that, the reaction was quenched by pouring it into a separatory funnel containing half-saturated aq. Rochelle’s salt, which was extracted with EA (3 times). The organic fractions were collected, dehydrated over Na_2_SO_4_ and evaporated under reduced pressure, obtaining the desired *N*-benzyl-protected DNM (**9**). *α-Stereoisomers* ((3*R*,6*R*)-**9** and (3*S*,6*S*)-**9**). HRMS (ESI): *m*/*z* calculated for C_26_H_31_NO + H^+^ [M + H^+^]: 374.2478 Found: 374.2487. ^1^H NMR (400 MHz, CDCl_3_) δ 7.56–7.49 (m, 2H), 7.37–7.14 (m, 13H), 4.04 (dd, *J* = 10.0, 2.6 Hz, 1H), 3.64 (d, *J* = 12.3 Hz, 1H), 3.42 (d, *J* = 12.4 Hz, 1H), 2.60–2.49 (m, 1H), 2.47–2.32 (m, 2H), 1.55–1.41 (m, 1H), 1.36–1.24 (m, 1H), 1.09 (d, *J* = 6.4 Hz, 3H), 0.88 (t, *J* = 7.4 Hz, 3H). ^13^C NMR (101 MHz, CDCl_3_) δ 148.61, 144.41, 139.22, 130.35, 128.93, 128.57, 128.45, 127.88, 127.72, 127.27, 125.99, 125.91, 81.41, 56.66, 51.32, 50.13, 26.47, 21.99, 12.17. **(3*****R*****,6*****R*****)-6-(benzylamino)-4,4-diphenylheptan-3-ol** ((3*R*,6*R*)-**9**). Obtained from (3*R*,6*R*)-**8**, white solid, quantitative yield. [α]_*D*_^25^ −39.80 (*c* = 1.5, CHCl_3_). **(3*****S*****,6*****S*****)-6-(benzylamino)-4,4-diphenylheptan-3-ol** ((3*S*,6*S*)-**9**). Obtained from (3*S*,6*S*)-**8**, white solid, quantitative yield. [α]_*D*_^25^ + 34.23 (*c* = 1.5, CHCl_3_). *β-stereoisomers* ((3*S*,6*R*)-**9** and (3*R*,6*S*)-**9**). HRMS (ESI): *m*/*z* calculated for C_26_H_31_NO + H^+^ [M + H^+^]: 374.2478 Found: 374.2471. ^1^H NMR (400 MHz, CDCl_3_) δ 7.42–7.37 (m, 2H), 7.36–7.25 (m, 7H), 7.24–7.17 (m, 3H), 7.16–7.10 (m, 3H), 4.17–4.09 (m, 1H), 3.77 (d, *J* = 12.2 Hz, 1H), 3.54 (d, *J* = 12.2 Hz, 1H), 2.65 (dd, *J* = 14.6, 8.8 Hz, 1H), 2.56–2.44 (m, 1H), 2.20 (d, *J* = 14.5 Hz, 1H), 1.23–1.12 (m, 1H), 1.11 (d, *J* = 6.4 Hz, 3H), 1.04–0.87 (m, 4H). ^13^C NMR (101 MHz, CDCl_3_) δ 147.72, 146.96, 139.05, 129.41, 128.82, 128.67, 128.61, 127.96, 127.60, 127.44, 125.72, 125.70, 75.91, 55.72, 51.11, 49.25, 45.34, 26.43, 22.16, 11.68. **(3*****S*****,6*****R*****)-6-(benzylamino)-4,4-diphenylheptan-3-ol** ((3*S*,6*R*)-**9**). Obtained from (3*S*,6*R*)-**8**, colorless gum, quantitative yield. [α]_*D*_^25^ −145.50 (*c* = 1.5, CHCl_3_). **(3*****R*****,6*****S*****)-6-(benzylamino)-4,4-diphenylheptan-3-ol** ((3*R*,6*S*)-**9**). Obtained from (3*R*,6*S*)-**8**, colorless gum, 98% yield. [α]_*D*_^25^ + 144.16 (*c* = 1.5, CHCl_3_).

#### General Procedure for the Synthesis of 10 and MLs

To an ice-cooled solution of amine **9** or **DNML** (1.0 equiv) in ACN/water 1:1 (8.75 mL/mmol) was added glacial acetic acid (0.5 equiv) and aq. formaldehyde (37% m/v, 3.5 equiv). Then, NaBH(OAc)_3_ (3.2 equiv) was added portionwise. The reaction mixture was warmed to room temperature and stirred for 1 h. At completion, the mixture was poured into saturated aq. NaHCO_3_ and extracted with DCM (3 times). The collected organic fractions were dried over anhydrous Na_2_SO_4_ and evaporated under reduced pressure. The crude product was purified by silica gel column chromatography to obtain the *N*-methylated amine (**10** or **ML**) using DCM/EA 97:3 to 9:1 as eluent (for compounds **10**), and CHCl_3_/MeOH 100:0 to 95:5 + 1% NH_3_ 7 M in MeOH (for β-stereoisomers (3*S*,6*R* and 3*R*,6*S*) of **ML**), or CHCl_3_/MeOH 100:0 to 98:1 + 1% NH_3_ 7 M in MeOH (for α-stereoisomers (3*R*,6*R* and 3*S*,6*S*) of **ML**). *α-stereoisomers* of **10** ((3*R*,6*R*)-**10** and (3*S*,6*S*)-**10**). HRMS (ESI): *m*/*z* calculated for C_27_H_33_NO + H^+^ [M + H^+^]: 388.2635 Found: 388.2639. ^1^H NMR (400 MHz, CDCl_3_) δ 7.61 (d, *J* = 7.0 Hz, 2H), 7.39–7.11 (m, 13H), 3.94 (dd, *J* = 9.8, 2.9 Hz, 1H), 3.67 (d, *J* = 12.8 Hz, 1H), 3.27 (d, *J* = 12.8 Hz, 1H), 2.83 (dd, *J* = 15.0, 8.8 Hz, 1H), 2.61–2.49 (m, 1H), 2.08 (s, 3H), 2.06 (dd, *J* = 14.9, 1.5 Hz, 1H), 1.75–1.59 (m, 1H), 1.30–1.16 (m, 1H), 0.87–0.82 (m, 6H). ^13^C NMR (101 MHz, CDCl_3_) δ 149.13, 143.85, 137.99, 130.98, 129.44, 128.95, 128.54, 127.76, 127.66, 127.38, 126.00, 125.79, 82.79, 57.18, 55.45, 49.20, 35.98, 26.40, 13.63, 12.26. **(3*****R*****,6*****R*****)-6-(benzyl(methyl)amino)-4,4-diphenylheptan-3-ol** ((3*R*,6*R*)-**10**). Obtained from (3*R*,6*R*)-**9**, colorless gum (72% yield). [α]_*D*_^25^ −58.4 (*c* = 1.5, CHCl_3_). **(3*****S*****,6*****S*****)-6-(benzyl(methyl)amino)-4,4-diphenylheptan-3-ol** ((3*S*,6*S*)-**10**). Obtained from (3*S*,6*S*)-**9**, colorless gum (73% yield). [α]_*D*_^25^ + 54.1 (*c* = 1.5, CHCl_3_). *β-stereoisomers* of **10** ((3*S*,6*R*)-**10** and (3*R*,6*S*)-**10**). HRMS (ESI): *m*/*z* calculated for C_27_H_33_NO + H^+^ [M + H^+^]: 388.2635 Found: 388.2647. ^1^H NMR (400 MHz, CDCl_3_) δ 7.39–7.09 (m, 15H), 4.15–4.07 (m, 1H), 3.65 (d, *J* = 12.6 Hz, 1H), 3.40 (d, *J* = 13.0 Hz, 1H), 2.97 (dd, *J* = 14.9, 8.6 Hz, 1H), 2.64 (p, *J* = 6.9 Hz, 1H), 2.13 (s, 3H), 1.95 (d, *J* = 14.9 Hz, 1H), 1.21–1.08 (m, 1H), 0.98 (t, 3H), 0.96–0.90 (m, 1H), 0.88 (d, *J* = 6.8 Hz, 3H). ^13^C NMR (101 MHz, CDCl_3_) δ 147.81, 146.87, 137.74, 129.62, 129.41, 128.60, 127.95, 127.62, 127.43, 125.72, 75.94, 58.51, 55.94, 54.46, 41.65, 35.50, 26.60, 13.62, 11.64. **(3*****S*****,6*****R*****)-6-(benzyl(methyl)amino)-4,4-diphenylheptan-3-ol** ((3*S*,6*R*)-**10**). Obtained from (3*S*,6*R*)-**9**, white solid (74% yield). [α]_*D*_^25^ −147.2 (*c* = 1.5, CHCl_3_). **(3*****R*****,6*****S*****)-6-(benzyl(methyl)amino)-4,4-diphenylheptan-3-ol** ((3*R*,6*S*)-**10**). Obtained from (3*R*,6*S*)-**9**, white solid (80% yield). [α]_*D*_^25^ + 148.5 (*c* = 1.5, CHCl_3_).

*α-stereoisomers* of **ML**s ((3*R*,6*R*)-**ML** and (3*S*,6*S*)-**ML**). HRMS (ESI): *m*/*z* calculated for C_21_H_29_NO + H^+^ [M + H^+^]: 312.2322 Found: 312.2325. ^1^H NMR (400 MHz, CDCl_3_) δ 7.65–7.58 (m, 2H), 7.33–7.11 (m, 8H), 3.80 (dd, *J* = 10.1, 3.0 Hz, 1H), 2.69 (dd, *J* = 14.9, 9.6 Hz, 1H), 2.30–2.21 (m, 1H), 2.16 (s, 6H), 2.00 (dd, *J* = 14.9, 1.4 Hz, 1H), 1.78–1.62 (m, 1H), 1.15–1.01 (m, 1H), 0.80 (t, *J* = 7.4 Hz, 3H), 0.76 (d, *J* = 6.8 Hz, 3H). ^13^C NMR (101 MHz, CDCl_3_) δ 149.62, 143.74, 131.11, 128.93, 127.73, 127.61, 125.95, 125.74, 83.47, 57.31, 55.18, 49.70, 39.31, 26.38, 12.51, 12.22. **(3*****R*****,6*****R*****)-6-(dimethylamino)-4,4-diphenylheptan-3-ol** ((3*R*,6*R*)-**ML**). Obtained from (3*R*,6*R*)-**DNML**, white solid (quantitative yield). [α]_*D*_^25^ + 7.52 (*c* = 1.5, CHCl_3_). UPLC purity 97.5%, *t*_R_ = 3.65 min. Chiral HPLC *t*_R_ = 5.8 min, e.e. > 99% (hexane +0.1% DEA/IPA 99:1, 1 mL/min). **(3*****S*****,6*****S*****)-6-(dimethylamino)-4,4-diphenylheptan-3-ol** ((3*S*,6*S*)-**ML**). Obtained from (3*S*,6*S*)-**DNML**, white solid (91% yield). [α]_*D*_^25^ −7.43 (*c* = 1.5, CHCl_3_). UPLC purity 97.6%, *t*_R_ = 3.66 min. Chiral HPLC *t*_R_ = 2.9 min, e.e. > 99% (hexane +0.1% DEA/EtOH 98:2). *β-stereoisomers* of **ML**s ((3*S*,6*R*)-**ML** and (3*R*,6*S*)-**ML**). HRMS (ESI): *m*/*z* calculated for C_21_H_29_NO + H^+^ [M + H^+^]: 312.2322 Found: 312.2328. ^1^H NMR (400 MHz, CDCl_3_) δ 7.53–7.47 (m, 2H), 7.31–7.24 (m, 2H), 7.22–7.07 (m, 6H), 4.08 (dd, *J* = 9.5, 2.5 Hz, 1H), 2.75 (dd, *J* = 14.9, 9.4 Hz, 1H), 2.44–2.32 (m, 1H), 2.16 (s, 6H), 2.00 (d, *J* = 14.9 Hz, 1H), 1.15–0.97 (m, 2H), 0.91 (t, *J* = 7.2 Hz, 3H), 0.82 (d, *J* = 6.8 Hz, 3H). ^13^C NMR (101 MHz, CDCl_3_) δ 148.91, 147.67, 129.52, 128.31, 127.96, 127.75, 125.61, 125.57, 77.24, 55.84, 55.11, 40.63, 39.23, 26.59, 12.73, 11.78. **(3*****S*****,6*****R*****)-6-(dimethylamino)-4,4-diphenylheptan-3-ol** ((3*S*,6*R*)-**ML**). Obtained from (3*S*,6*R*)-**DNML**, white solid (quantitative yield). [α]_*D*_^25^ −133.61 (*c* = 1.5, CHCl_3_). UPLC purity 98.1%, *t*_R_ = 3.88 min. Chiral HPLC *t*_R_ = 5.4 min, e.e. > 99% (hexane +0.1% DEA/IPA 99:1, 1 mL/min). **(3*****R*****,6*****S*****)-6-(dimethylamino)-4,4-diphenylheptan-3-ol** ((3*R*,6*S*)-**ML**). Obtained from (3*R*,6*S*)-**DNML**, white solid (87% yield). [α]_*D*_^25^ + 131.86 (*c* = 1.5, CHCl_3_). UPLC purity >99%, *t*_R_ = 3.82 min. Chiral HPLC *t*_R_ = 2.7 min, e.e. > 99% (hexane +0.1% DEA/EtOH 98:2).

#### General Procedure for the Synthesis of DNMLs and NMLs

To a stirred solution of **9** or **10** (1.0 equiv) in absolute ethanol (3.8 mL/mmol) was added Pd/C (10% loading, 20% w/w) and ammonium formate (5.0 equiv). The reaction was refluxed under nitrogen atmosphere for 4 h. At completion, the reaction was filtered over a short pad of Celite, which was washed with MeOH. The solvents were evaporated under reduced pressure, the residue was redissolved in DCM and washed with saturated aq. NaHCO_3_. The aqueous phase was further extracted with DCM (3 times). The organic fractions were collected, dehydrated over Na_2_SO_4_ and evaporated under reduced pressure. The crude product was purified by silica gel column chromatography using DCM/MeOH 100:0 to 9:1 + 1% NH_3_ (7 M in MeOH), obtaining **DNML** or **NML**. *α-stereoisomers* of **DNML**s ((3*R*,6*R*)-**DNML** and (3*S*,6*S*)-**DNML**). HRMS (ESI): *m*/*z* calculated for C_19_H_25_NO + H^+^ [M + H^+^]: 284.2009 Found: 284.2011. ^1^H NMR (400 MHz, CDCl_3_) δ 7.55–7.47 (m, 2H), 7.35–7.13 (m, 8H), 4.03 (dd, *J* = 10.2, 2.7 Hz, 1H), 2.74–2.62 (m, 1H), 2.41 (dd, *J* = 14.6, 1.8 Hz, 1H), 2.23 (dd, *J* = 14.6, 8.7 Hz, 1H), 1.50–1.34 (m, 1H), 1.29–1.15 (m, 1H), 1.05 (d, *J* = 6.4 Hz, 3H), 0.87 (t, *J* = 7.3 Hz, 3H). ^13^C NMR (101 MHz, CDCl_3_) δ 148.08, 144.16, 130.47, 128.94, 127.88, 127.75, 126.05, 125.95, 81.14, 56.56, 52.18, 44.04, 27.87, 26.42, 12.05. **(3*****R*****,6*****R*****)-6-amino-4,4-diphenylheptan-3-ol** ((3*R*,6*R*)-**DNML**). Obtained from (3*R*,6*R*)-**9**, white solid (quantitative yield). [α]_*D*_^25^ −5.40 (*c* = 1.5, CHCl_3_). UPLC purity 99%, *t*_R_ = 3.39 min. Chiral HPLC *t*_R_ = 8.6 min, e.e. > 98% (hexane +0.1% DEA/IPA 95:5). **(3*****S*****,6*****S*****)-6-amino-4,4-diphenylheptan-3-ol** ((3*S*,6*S*)-**DNML**). Obtained from (3*S*,6*S*)-**9**, white solid (88% yield). [α]_*D*_^25^ + 4.44 (*c* = 1.5, CHCl_3_). UPLC purity 96.8%, *t*_R_ = 3.34 min. Chiral HPLC *t*_R_ = 8.0 min, e.e. > 99% (hexane +0.1% DEA/IPA 95:5). *β-stereoisomers* of **DNML**s ((3*S*,6*R*)-**DNML** and (3*R*,6*S*)-**DNML**). HRMS (ESI): *m*/*z* calculated for C_19_H_25_NO + H^+^ [M + H^+^]: 284.2009 Found: 284.2015. ^1^H NMR (400 MHz, CDCl_3_) δ 7.53–7.44 (m, 2H), 7.33–7.25 (m, 2H), 7.24–7.14 (m, 3H), 7.16–7.07 (m, 3H), 4.20 (dd, *J* = 10.0, 2.1 Hz, 1H), 2.71–2.60 (m, 1H), 2.56–2.48 (m, 1H), 2.28 (d, *J* = 14.5 Hz, 1H), 1.28–1.04 (m, 2H), 1.11 (d, *J* = 6.4 Hz, 3H), 0.95 (t, *J* = 7.2 Hz, 3H). ^13^C NMR (101 MHz, CDCl_3_) δ 148.26, 147.14, 129.43, 128.37, 128.00, 127.75, 125.69, 125.67, 76.94, 55.45, 45.14, 43.94, 28.94, 26.29, 11.82. **(3***S***,6***R***)-6-amino-4,4-diphenylheptan-3-ol** ((3*S*,6*R*)-**DNML**). Obtained from (3*S*,6*R*)-**9**, white solid (98% yield). [α]_*D*_^25^ −155.41 (*c* = 1.5, CHCl_3_). UPLC purity 98.6%, *t*_R_ = 3.64 min. Chiral HPLC *t*_R_ = 7.2 min, e.e. > 99% (hexane +0.1% DEA/IPA 95:5). **(3*****R*****,6*****S*****)-6-amino-4,4-diphenylheptan-3-ol** ((3*R*,6*S*)-**DNML**). Obtained from (3*R*,6*S*)-**9**, white solid (94% yield). [α]_*D*_^25^ + 158.69 (*c* = 1.5, CHCl_3_). UPLC purity >99%, *t*_R_ = 3.64 min. Chiral HPLC *t*_R_ = 7.5 min, e.e. > 99% (hexane +0.1% DEA/IPA 95:5).

*α-stereoisomers* of **NML**s ((3*R*,6*R*)-**NML** and (3*S*,6*S*)-**NML**). HRMS (ESI): *m*/*z* calculated for C_20_H_27_NO + H^+^ [M + H^+^]: 298.2165 Found: 298.2160. ^1^H NMR (400 MHz, CDCl_3_) δ 7.59–7.52 (m, 2H), 7.34–7.10 (m, 8H), 3.90 (dd, *J* = 10.3, 2.8 Hz, 1H), 2.44–2.25 (m, 2H), 2.25–2.15 (m, 1H), 2.19 (s, 3H), 1.67–1.51 (m, 1H), 1.20–1.02 (m, 1H), 1.00 (d, *J* = 6.4 Hz, 3H), 0.84 (t, *J* = 7.3 Hz, 3H). ^13^C NMR (101 MHz, CDCl_3_) δ 148.98, 143.96, 130.71, 128.90, 127.82, 127.68, 126.00, 125.86, 82.37, 56.85, 52.31, 51.64, 32.79, 26.27, 20.91, 12.09. **(3*****R*****,6*****R*****)-6-(methylamino)-4,4-diphenylheptan-3-ol** ((3*R*,6*R*)-**NML**). Obtained from (3*R*,6*R*)-**10**, colorless gum (99% yield). [α]_*D*_^25^ −12.44 (*c* = 1.5, CHCl_3_). UPLC purity 98.6%, *t*_R_ = 3.54 min. Chiral HPLC *t*_R_ = 8.8 min, e.e. > 98% (hexane +0.1% DEA/IPA 98:2). **(3*****S*****,6*****S*****)-6-(methylamino)-4,4-diphenylheptan-3-ol** ((3*S*,6*S*)-**NML**). Obtained from (3*S*,6*S*)-**10**, colorless gum (91% yield). [α]_*D*_^25^ + 11.18 (*c* = 1.5, CHCl_3_). UPLC purity 96.7%, *t*_R_ = 3.54 min. Chiral HPLC *t*_R_ = 11.2 min, e.e. > 99% (hexane +0.1% DEA/IPA 98:2). *β-stereoisomers* of **NML**s ((3*S*,6*R*)-**NML** and (3*R*,6*S*)-**NML**). HRMS (ESI): *m*/*z* calculated for C_20_H_27_NO + H^+^ [M + H^+^]: 298.2165 Found: 298.2173. ^1^H NMR (400 MHz, CDCl_3_) δ 7.52–7.44 (m, 2H), 7.33–7.24 (m, 2H), 7.23–7.15 (m, 3H), 7.14–7.07 (m, 3H), 4.13 (dd, *J* = 9.6, 2.4 Hz, 1H), 2.54 (dd, *J* = 14.6, 9.0 Hz, 1H), 2.32–2.27 (m, 1H), 2.27 (s, 3H), 2.28–2.19 (m, 1H), 1.18–1.06 (m, 2H), 1.04 (d, *J* = 6.5 Hz, 3H), 0.93 (t, *J* = 7.2 Hz, 3H). ^13^C NMR (101 MHz, CDCl_3_) δ 148.55, 147.47, 129.43, 128.34, 127.94, 127.70, 125.59, 77.03, 55.61, 51.09, 44.29, 32.50, 26.44, 21.30, 11.77. **(3*****S*****,6*****R*****)-6-(methylamino)-4,4-diphenylheptan-3-ol** ((3*S*,6*R*)-**NML**). Obtained from (3*S*,6*R*)-**10**, white solid (94% yield). [α]_*D*_^25^ −166.62 (*c* = 1.5, CHCl_3_). UPLC purity 98.2%, *t*_R_ = 3.76 min. Chiral HPLC *t*_R_ = 6.7 min, e.e. > 99% (hexane +0.1% DEA/IPA 98:2). **(3*****R*****,6*****S*****)-6-(methylamino)-4,4-diphenylheptan-3-ol** ((3*R*,6*S*)-**NML**). Obtained from (3*R*,6*S*)-**10**, white solid (88% yield). [α]_*D*_^25^ + 158.22 (*c* = 1.5, CHCl_3_). UPLC purity 97.6%, *t*_R_ = 3.76 min. Chiral HPLC *t*_R_ = 10.3 min, e.e. > 99% (hexane +0.1% DEA/IPA 98:2).

### FLIPR Assays

Eleven concentrations were assessed in 5 replicates for each compound: 100–33–11–3.7–1.2 μM, then 412–137–46–15–5.1–1.7 nM. l-glutamate and glycine were both present at 10 μM concentration. 400 × compound plates were prepared by Echo Labcyte system, containing in every compound well: 10 nl/well of 12,000 × l-glutamate/glycine solution in H_2_O and 300 nl/well of 400 × test item solution in DMSO. 400 × compound plate was stored at −20 °C until FLIPR experimental day. On the experiment day, 4 × assay plates were prepared by adding 30 μL/well of assay buffer in the presence of 0.5% (v/v) poloxamer 188 solution. Assay buffer composition was 145 mM NaCl, 5 mM KCl, 2 mM CaCl_2_, 1 g/Liter D-(+)-glucose, and 20 mM HEPES (pH 7.3 adjusted with NaOH). Cells were plated at a density of 15.000/well in 384 black/clear bottom plates. Receptor expression was induced with 500 μM ketamine and 10 μg/mL tetracycline, except for NR1–2B-CHO cells, which showed NMDAR constitutive expression. Cells were kept at 30 °C in 5% CO_2_ incubator for 24 h. Cells were preloaded for 1 h with 2 μM Fluo-4 Ca^2+^-sensitive probe, simultaneously with 2.5 mM probenecid, an inhibitor of nonspecific anion transport, and 500 μM ketamine to avoid cytotoxicity. After that, cells were washed with a three-cycle wash in assay buffer to remove all the ketamine, as demonstrated by a consistent full response elicited in positive control wells by glutamate and glycine. Intracellular Ca^2+^ level was monitored by FLIPR (excitation wavelength at 470–495 nm, emission wavelength at 515–575 nm) for 10 s before and 5 min after the addition of test items. All test items, including l-glutamate and glycine, were added simultaneously. Fluorescence was measured as the area under the curve (AUC) during the 5 min immediately after the addition of the test item. FLIPR data were normalized as 100%, in the presence of 10 μM l-glutamate and 10 μM glycine, while as 0%, in the presence of assay buffer. AUC values of fluorescence were measured by ScreenWorks 4.1 (Molecular Devices) FLIPR software, to monitor calcium level during the 5 min after test item addition (AUC 10–310 s). Then, data were normalized by Excel 2013 (Microsoft Office) software, using wells added with 10 μM l-glutamate plus 10 μM glycine (column 23) as high control, and wells added with assay buffer only (column 24) as low control. To assess plate quality, *Z*′ calculations were performed in Excel. *Z*′ was calculated according to following equation: *Z*′ = 1–3(σh + σl)/|μh – μL| where μ and σ are the means and the standard deviations of the means of high (h) and low (l) controls, respectively. Compound concentration–response curves were plotted by Prism 8 GraphPad software, in the different experimental conditions (see Supporting Information). Data were analyzed using least-squares regression fitting, to fit data to a standard four parameters logistic equation using GraphPad Prism10. This regression fitting allowed the calculation of pIC50 and the corresponding SEM.

### Radioligand Displacement Binding Assays

Selected compounds were submitted to Eurofins Panlabs Discovery Services (Taiwan) for radioligand displacement binding assays over an array of CNS receptors. For NMDA receptor, cerebral cortices of Wistar rats weighing 150–200 g were dissected, homogenized, and washed. In the other cases, cell lines expressing the target receptor are used. The specific conditions (receptor source, radioligand, nonspecific ligand, incubation time and temperature, buffer composition, specific binding %, test items concentrations) are listed: **Glutamate, NMDA, Phencyclidine binding site**. Source: Wistar rat cerebral cortex. Ligand: 4.0 nM [^3^H] TCP. Nonspecific ligand: 1.0 μM (+)-MK-801. Test compound concentrations: 1 μM and 10 μM. Incubation time and temperature: 1.5 h, 25 °C. Specific binding: 94%. Incubation buffer: 10 mM Tris-HCl, pH 7.4. **Histamine H**_**1**_. Source: Human recombinant CHO-K1 cells. Ligand: 1.20 nM [^3^H] pyrilamine. Nonspecific ligand: 1.0 μM pyrilamine. Test compound concentrations: 30 μM and 10 μM. Incubation time and temperature: 3 h, 25 °C. Specific binding: 94%. Incubation buffer: 10 mM Tris-HCl, pH 7.4, 5 mM MgCl_2_. **Muscarinic M**_**5**_. Source: Human recombinant CHO-K1 cells. Ligand: 0.8 nM [^3^H] *N*-methylscopolamine. Nonspecific ligand: 1.0 μM atropine. Test compound concentrations: 1 and 10 μM. Incubation time and temperature: 2 h, 25 °C. Specific binding: 95%. Incubation buffer: 50 mM Tris-HCl, pH 7.4, 10 mM MgCl_2_, 1 mM EDTA. **μ opioid receptor (MOP)**. Source: Human recombinant CHO-K1 cells. Ligand: 0.6 nM [^3^H] diprenorphine. Nonspecific ligand: 10.0 μM naloxone. Test compound concentrations: 0.3 μM and 3 μM. Incubation time and temperature: 1 h, 25 °C. Specific binding: 90%. Incubation buffer: 50 mM Tris-HCl, pH 7.4. **Serotonin 5-HT**_**1A**_. Source: Human recombinant CHO-K1 cells. Ligand: 1.50 nM [^3^H] 8-OH-DPAT. Nonspecific ligand: 10.0 μM metergoline. Test compound concentrations: 0.3 μM and 3 μM. Incubation time and temperature: 1 h, 25 °C. Specific binding: 75%. Incubation buffer: 50 mM Tris-HCl, pH 7.4, 0.1% ascorbic acid, 10 mM MgSO_4_, 0.5 mM EDTA. **Serotonin 5-HT**_**2A**_. Source: Human recombinant CHO-K1 cells. Ligand: 1.0 nM [^3^H] ketanserin. Nonspecific ligand: 1.0 μM mianserin. Test compound concentrations: 0.3 μM and 3 μM. Incubation time and temperature: 1 h, 25 °C. Specific binding: 90%. Incubation buffer: 50 mM Tris-HCl, pH 7.4. **Serotonin 5-HT**_**2C**_. Source: Human recombinant CHO-K1 cells. Ligand: 1.0 nM [^3^H] mesulergine. Nonspecific ligand: 1.0 μM mianserin. Test compound concentrations: 0.3 μM and 3 μM. Incubation time and temperature: 1 h, 25 °C. Specific binding: 90%. Incubation buffer: 50 mM Tris-HCl, pH 7.4, 0.1% ascorbic acid, 10 μM pargyline. **Serotonin 5-HT**_**5A**_. Source: Human recombinant CHO-K1 cells. Ligand: 1.7 nM [^3^H] lysergic acid diethylamide (LSD). Nonspecific ligand: 100 μM serotonin. Test compound concentrations: 1 μM and 10 μM. Incubation time and temperature: 1 h, 37 °C. Specific binding: 80%. Incubation buffer: 50 mM Tris-HCl, pH 7.4, 10 mM MgCl_2_, 0.5 mM EDTA. **Serotonin 5-HT**_**7**_. Source: Human recombinant CHO-K1 cells. Ligand: 5.50 nM [^3^H] lysergic acid diethylamide. Nonspecific ligand: 10.0 μM serotonin. Test compound concentrations: 1 μM and 10 μM. Incubation time and temperature: 2 h, 25 °C. Specific binding: 90%. Incubation buffer: 50 mM Tris-HCl, pH 7.4, 10 mM MgCl_2_, 0.5 mM EDTA. **Sigma σ**_**1**_. Source: Human Jurkat cells. Ligand: 15.0 nM [^3^H] pentazocine. Nonspecific ligand: 10.0 μM haloperidol. Test compound concentrations: 0.3 μM and 3 μM. Incubation time and temperature: 2 h, 37 °C. Specific binding: 90%. Incubation buffer: 50 mM Tris-HCl, pH 8.0. **Norepinephrine transporter NET**. Source: Human recombinant MDCK cells. Ligand: 0.2 nM [^125^I] RTI-55. Nonspecific ligand: 10.0 μM desipramine. Test compound concentrations: 1 μM and 10 μM. Incubation time and temperature: 3 h, 4 °C. Specific binding: 75%. Incubation buffer: 50 mM Tris-HCl, pH 7.4, 100 mM NaCl, 1 μM leupeptin, 10 μM PMSF. **Serotonin transporter SERT**. Source: Human recombinant HEK-293 cells. Ligand: 0.4 nM [^3^H] paroxetine. Nonspecific ligand: 10.0 μM imipramine. Test compound concentrations: 1 μM and 10 μM. Incubation time and temperature: 1 h, 25 °C. Specific binding: 95%. Incubation buffer: 50 mM Tris-HCl, pH 7.4, 120 mM NaCl, 5 mM KCl. The values of Inhibition constants (*K*_i_), were calculated using the equation of Cheng and Prusoff^55^ using the observed IC_50_ of the tested compound, determined by a nonlinear, least-squares regression analysis using MathIQTM (IDBusiness Solutions Ltd., UK), the concentration of radioligand employed in the assay, and the historical values for the *K*_D_ of the ligand. pKi values were calculated as −log (*K*_i_). In the table, p*K*_i_ are reported as mean ± SEM of the independent experiments (see Supporting Information for concentration–response curves).

## Supporting Information Available

^1^H- and ^13^C NMR spectra, UPLC and chiral HPLC chromatograms, X-ray crystallography, FLIPR assay concentration–response curves, two concentrations and concentration–response curves for radioligand displacement assay results of the synthesized compounds (PDF)
SMILES structures with pharmacological data (CSV)
Coordinates of compound (3*R*,6*S*)-DNML docking pose in 6WHT (PDB)
Coordinates of compound (3R,6S)-ML docking pose in 6WHT (PDB)

## Supplementary Material

jm4c02605_si_002.csv

jm4c02605_si_003.pdb

jm4c02605_si_004.pdb
