# Supplementary material for: New Synthesis and Pharmacological Evaluation of Enantiomerically Pure (R)- and (S)-Methadone Metabolites as N-Methyl-d-aspartate Receptor Antagonists
Source: J Med Chem. 2025 Feb 25;68(5):5455–70. doi: 10.1021/acs.jmedchem.4c02605 (PMC11912475; doi:10.1021/acs.jmedchem.4c02605)
Supplement: Supplementary file 1 — jm4c02605_si_001.pdf [file jm4c02605_si_001.pdf]

SUPPORTING INFORMATION

New Synthesis and Pharmacological Evaluation of Enantiomerically Pure (R)- and (S)-Methadone Metabolites as N-methyl-D-aspartate Receptor Antagonists

Marco Banzato[1,#], Alberto Furlan[1,2,#], Patrizia Locatelli[2], Jacopo Sgrignani[2], Alberto Ongaro[1], Alessandro Dolmella[1], Sara De Martin[1], Stefano Comai[1,3,4,5], Andrea Cavalli[2], Charles Inturrisi[6], Ezio Bettini[7], Paolo L. Manfredi[6,*], Andrea Mattarei[1,*]

1. *Department of Pharmaceutical and Pharmacological Sciences, University of Padova, Via Francesco Marzolo 5, 35131, Padua, Italy*
2. *Institute for Research in Biomedicine, Via Chiesa 5, 6500, Bellinzona, Switzerland*
3. *Department of Biomedical Sciences, University of Padova, Via Ugo Bassi 58/B, 35131,. Padua, Italy*
4. *Department of Psychiatry, McGill University, 1033 Pine Avenue West, H3A 1A1 Montreal, QC, Canada*
5. *IRCSS San Raffaele Scientific Institute, via Olgettina 58, 20132, Milan, Italy*
6. *Relmada Therapeutics, Coral Gables, 33134, Florida, United States*
7. *In Vitro Pharmacology Department, Aptuit, An Evotec Company, Via Alessandro Fleming, 4, 37135, Verona, Italy*

Please address correspondence to Andrea Mattarei at andrea.mattarei@unipd.it or Paolo L. Manfredi at PManfredi@relmada.com

Table of Contents

NMR spectra, UPLC and chiral HPLC chromatograms of the synthesized compounds S2

X-ray crystallography S77

FLIPR assay concentration-response curves S90

Radioligand displacement assay results S94

Radioligand displacement assay concentration-response curves S95

NMR spectra, UPLC and chiral HPLC chromatograms of the synthesized compounds

^{1}H and ^{13}C NMR spectra of (*R*)-tert-butyl (1-hydroxypropan-2-yl)carbamate ((*R*)-**1**).

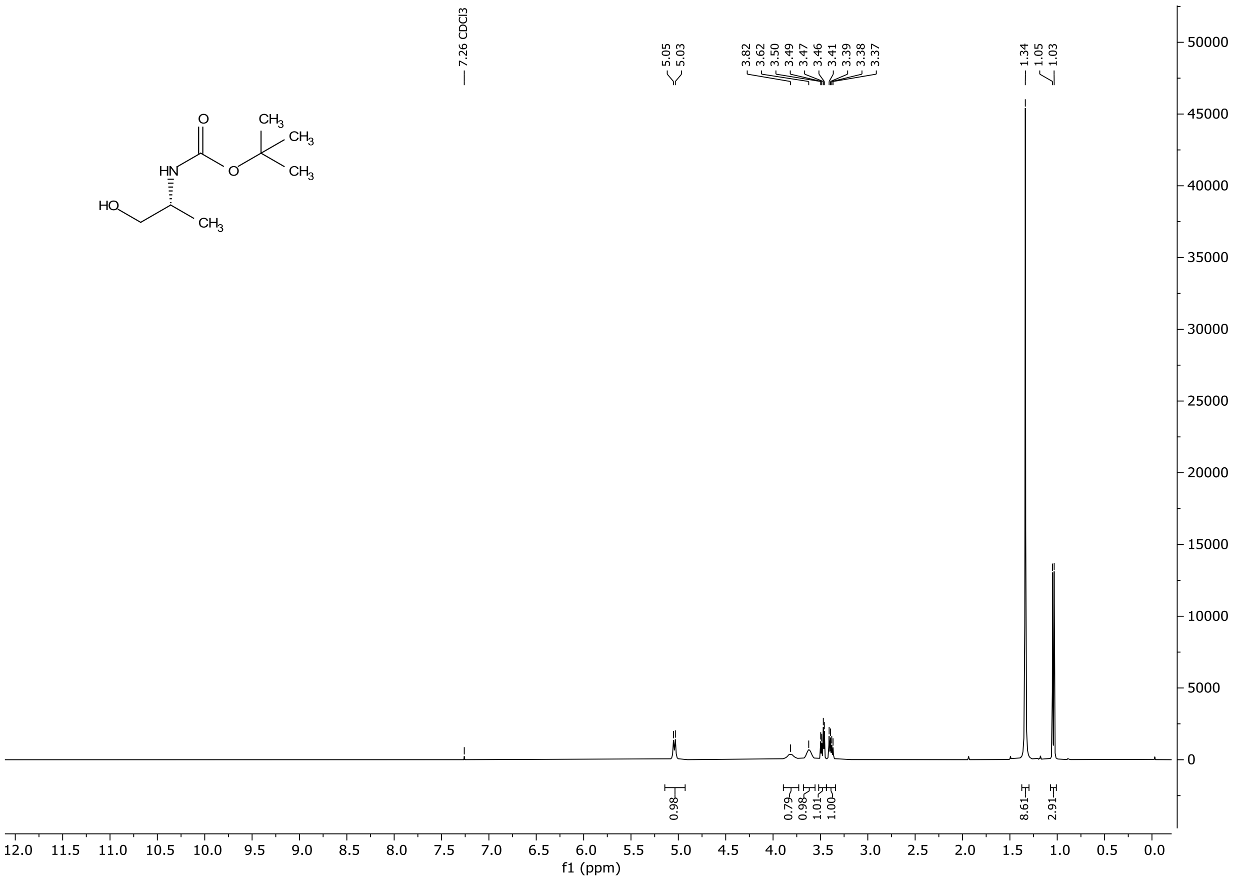

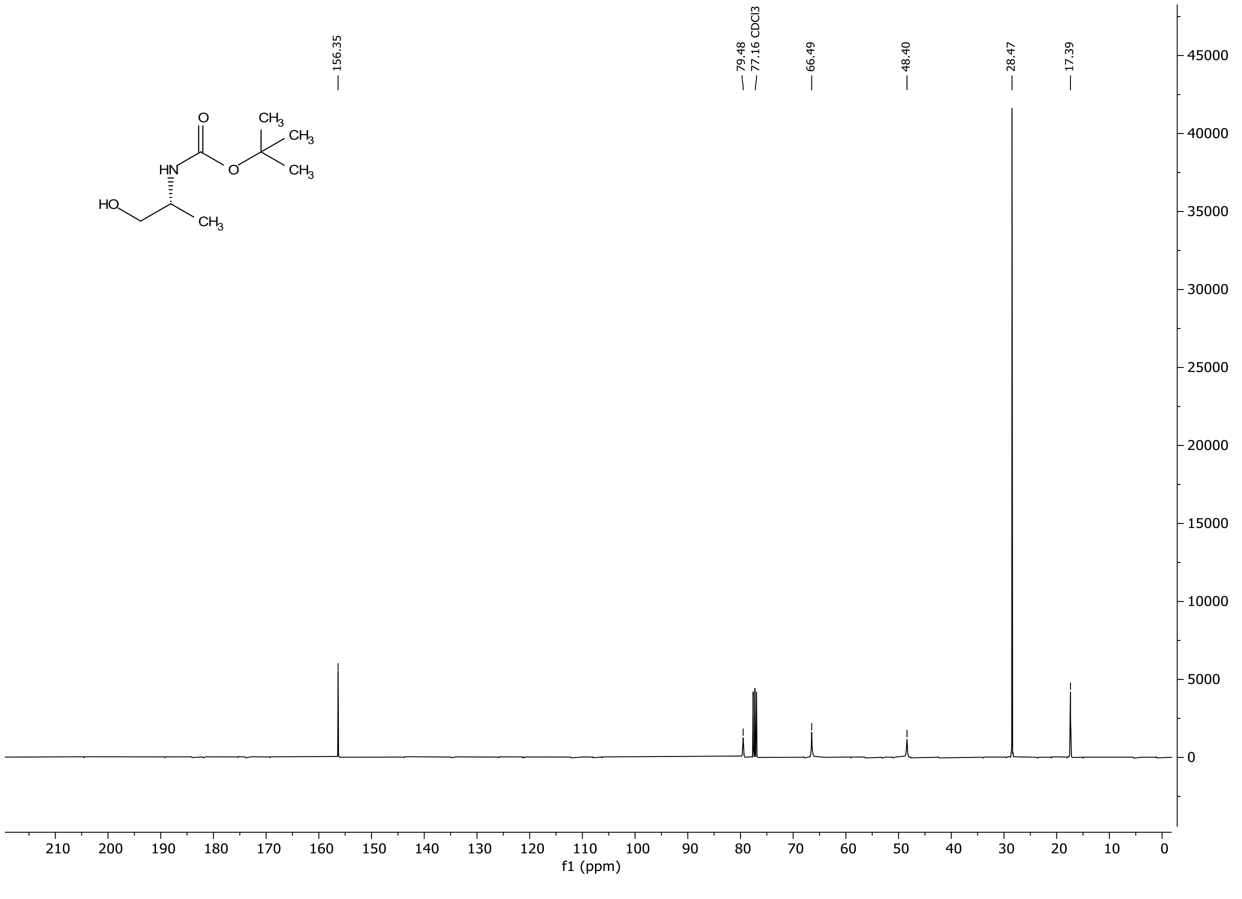

^{1}H and ^{13}C NMR spectra of (*S*)-tert-butyl (1-hydroxypropan-2-yl)carbamate ((*S*)-**1**).

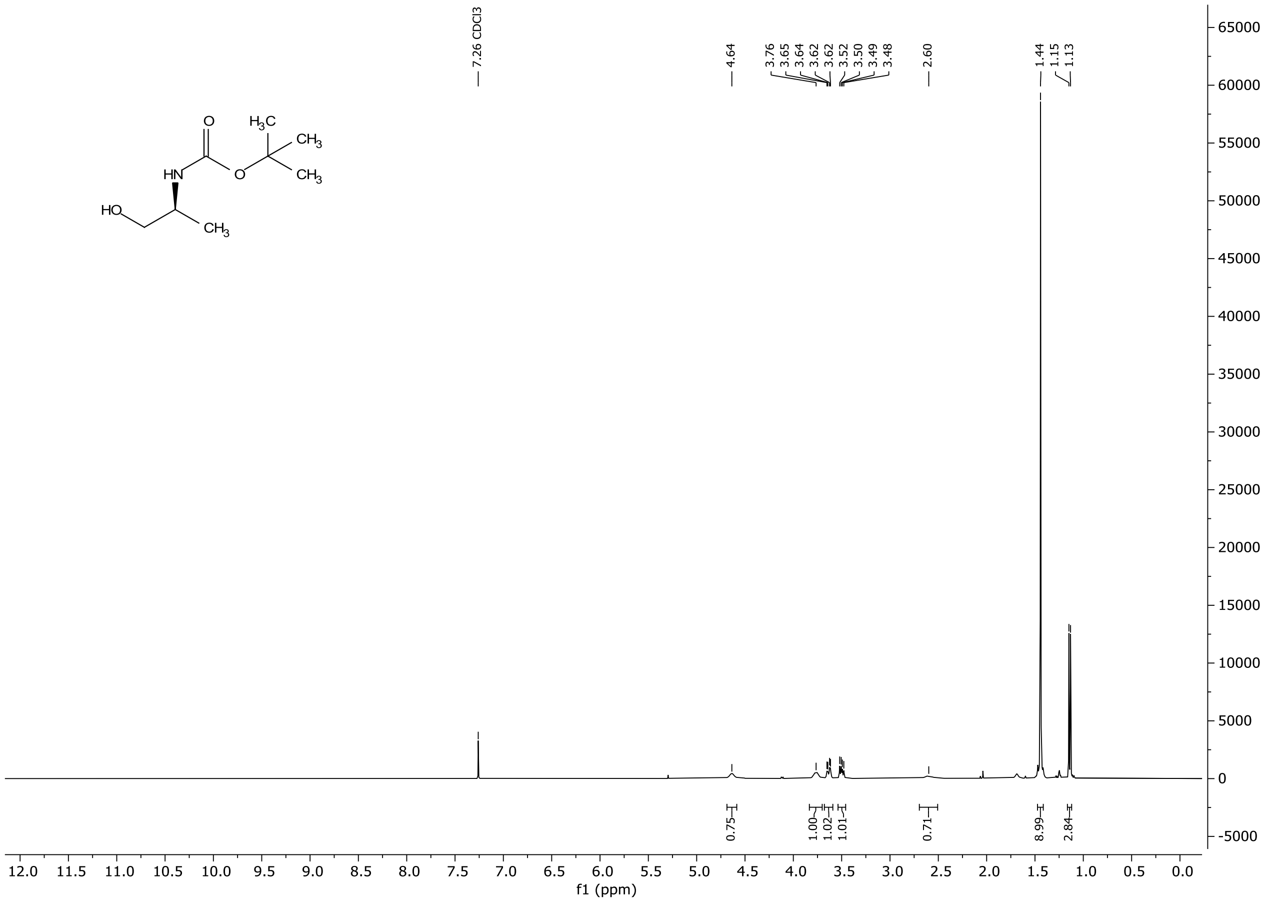

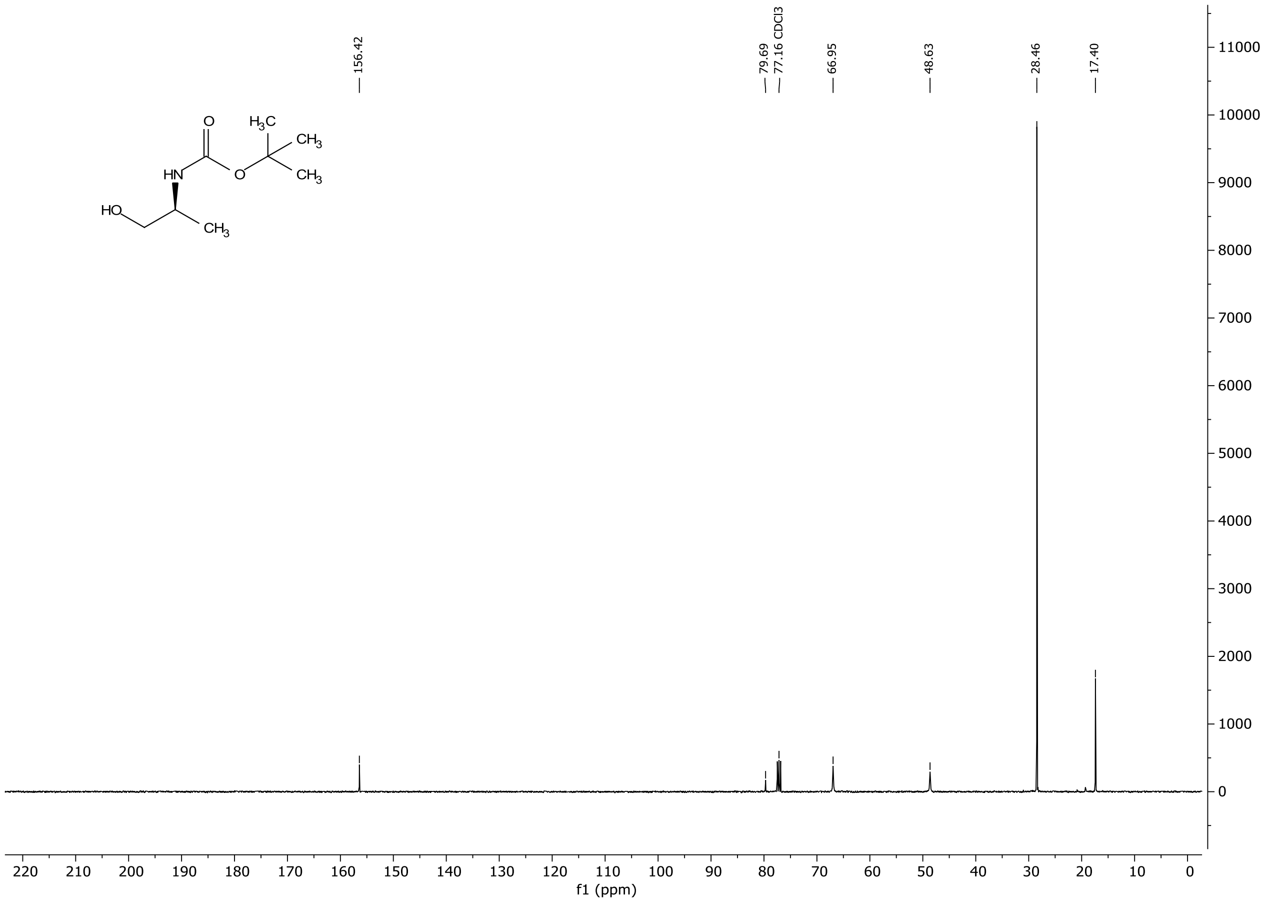

^{1}H and ^{13}C NMR spectra of (*R*)-tert-butyl 4-methyl-1,2,3-oxathiazolidine-3-carboxylate 2,2-dioxide ((*R*)- **2**).

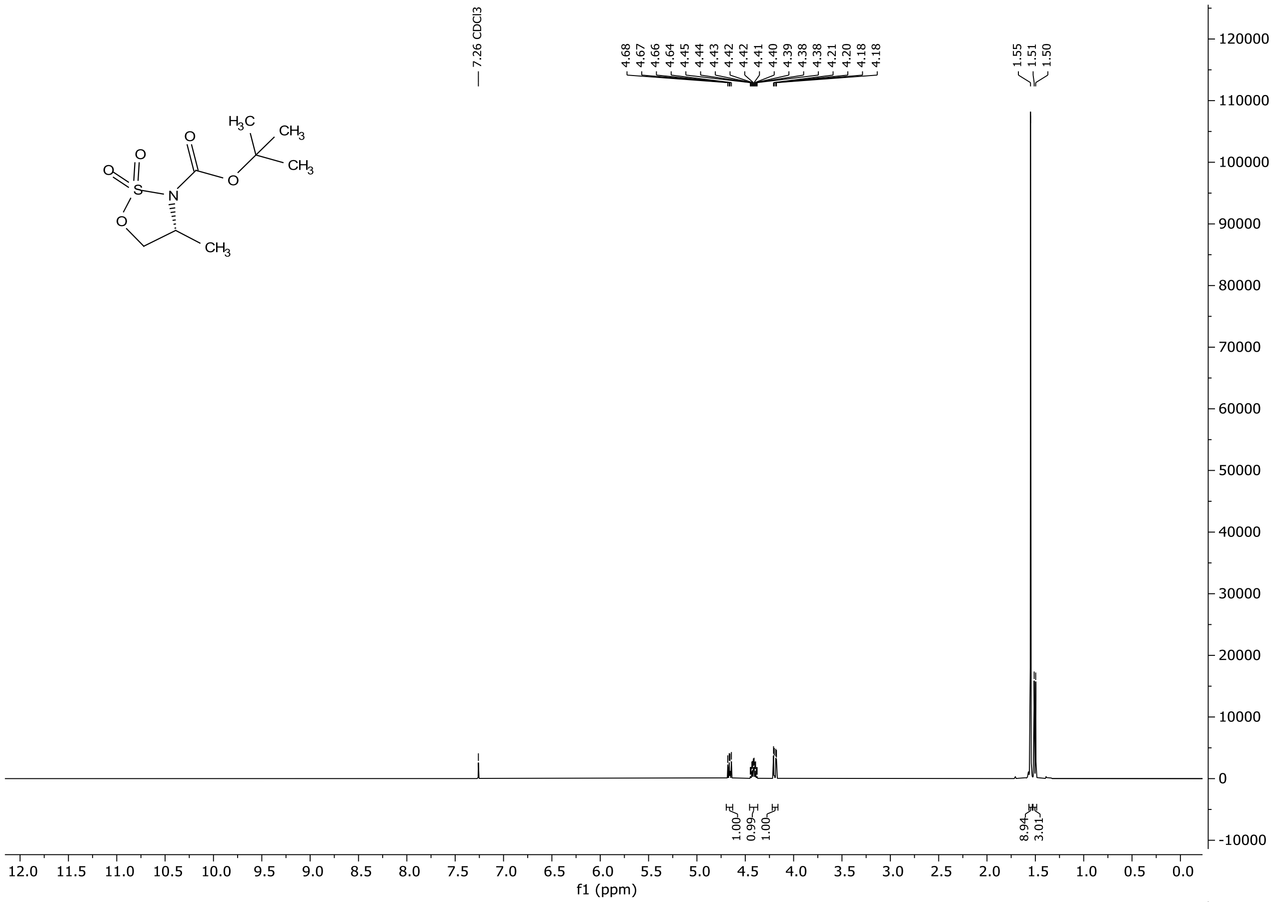

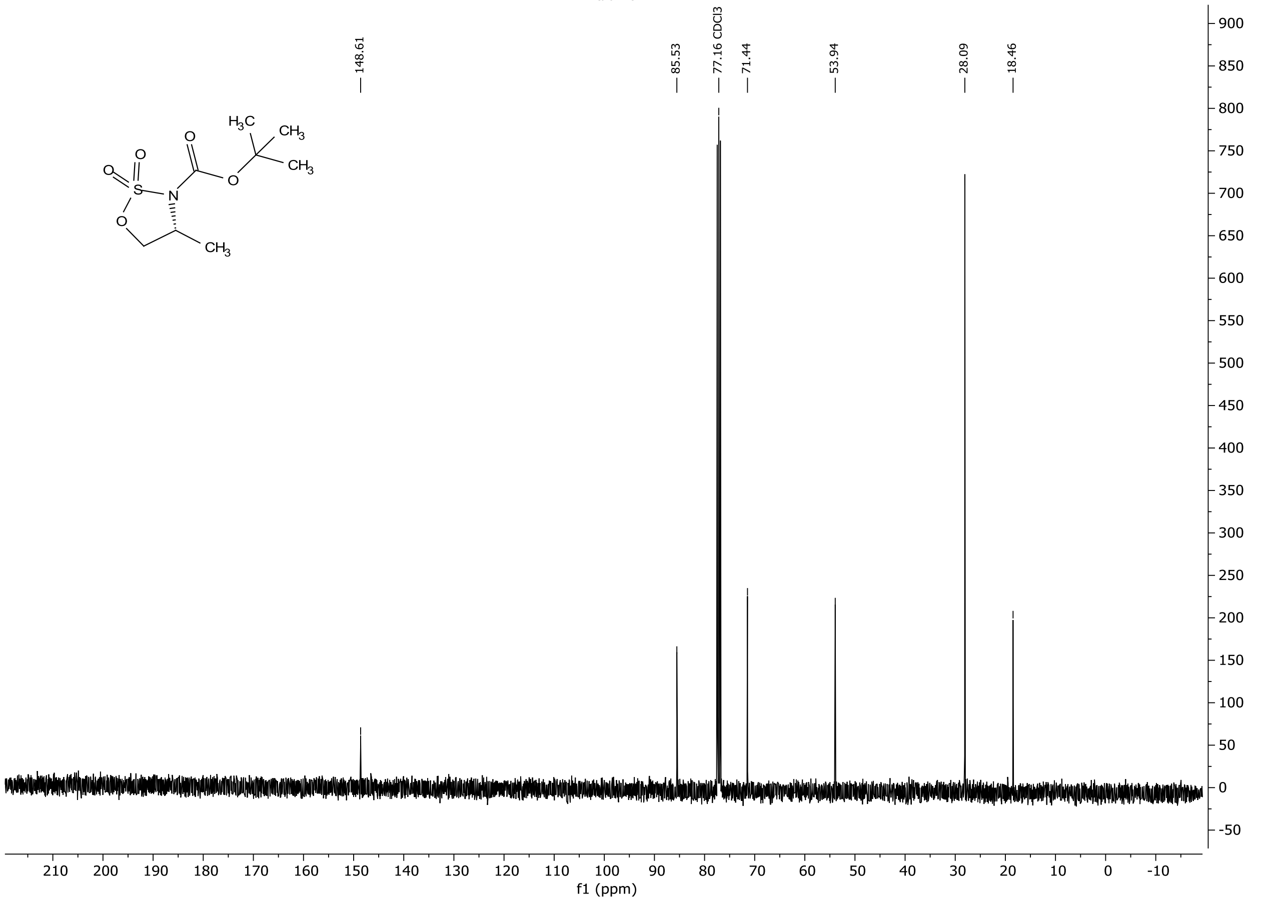

^{1}H and ^{13}C NMR spectra of (*S*)-tert-butyl 4-methyl-1,2,3-oxathiazolidine-3-carboxylate 2,2-dioxide ((*S*)-**2**).

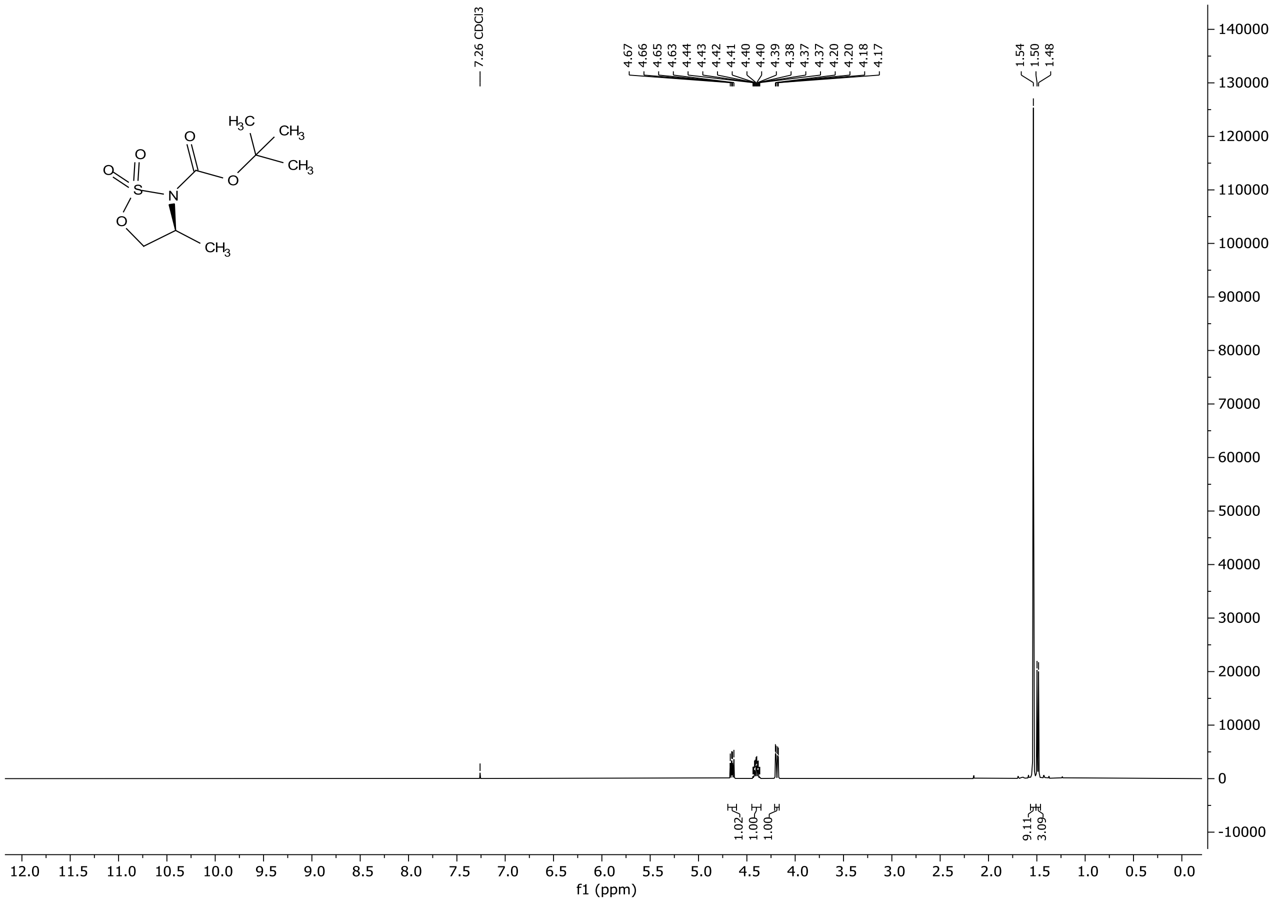

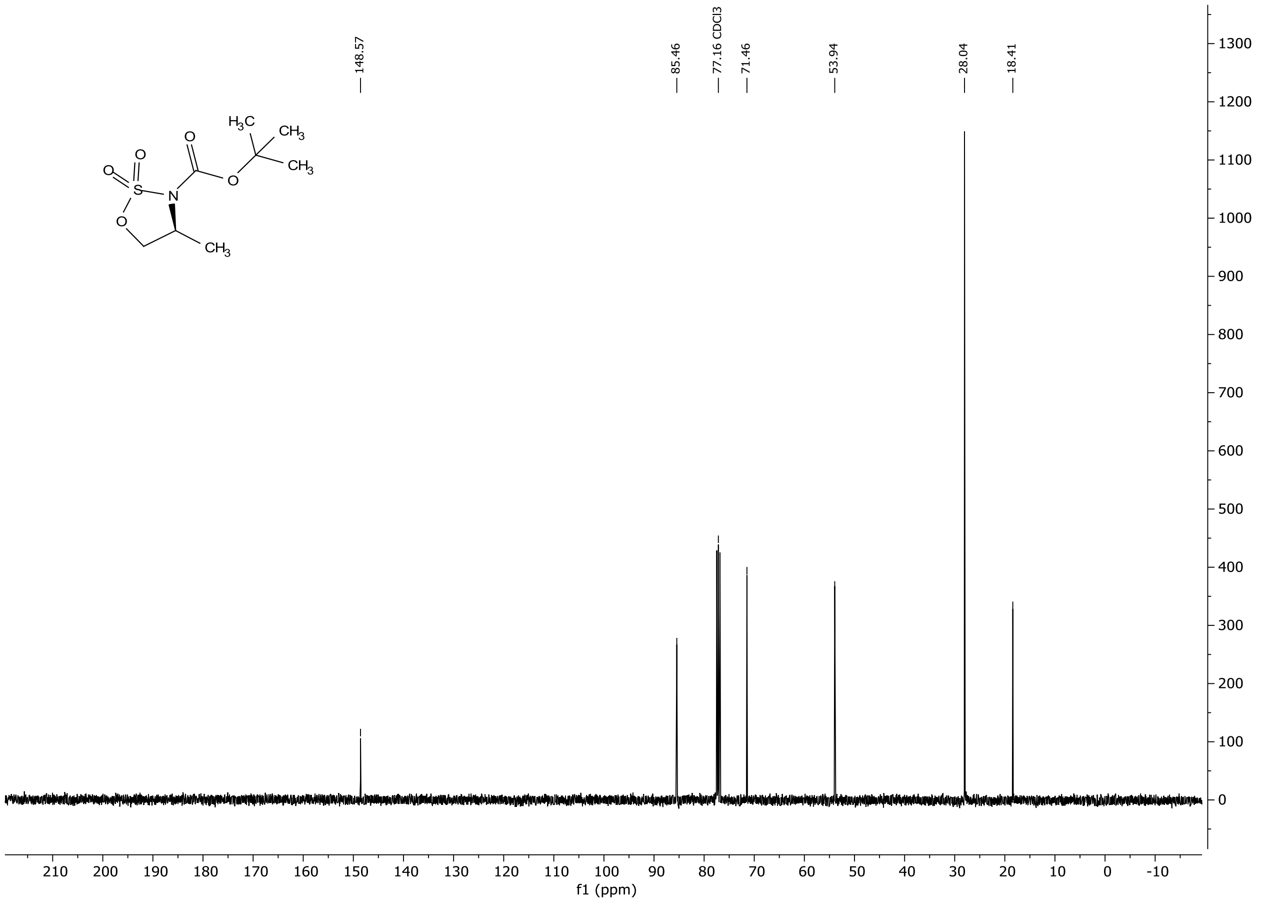

^{1}H and ^{13}C NMR spectra of (*R*)-tert-butyl (4-cyano-4,4-diphenylbutan-2-yl)carbamate ((*R*)-**3**).

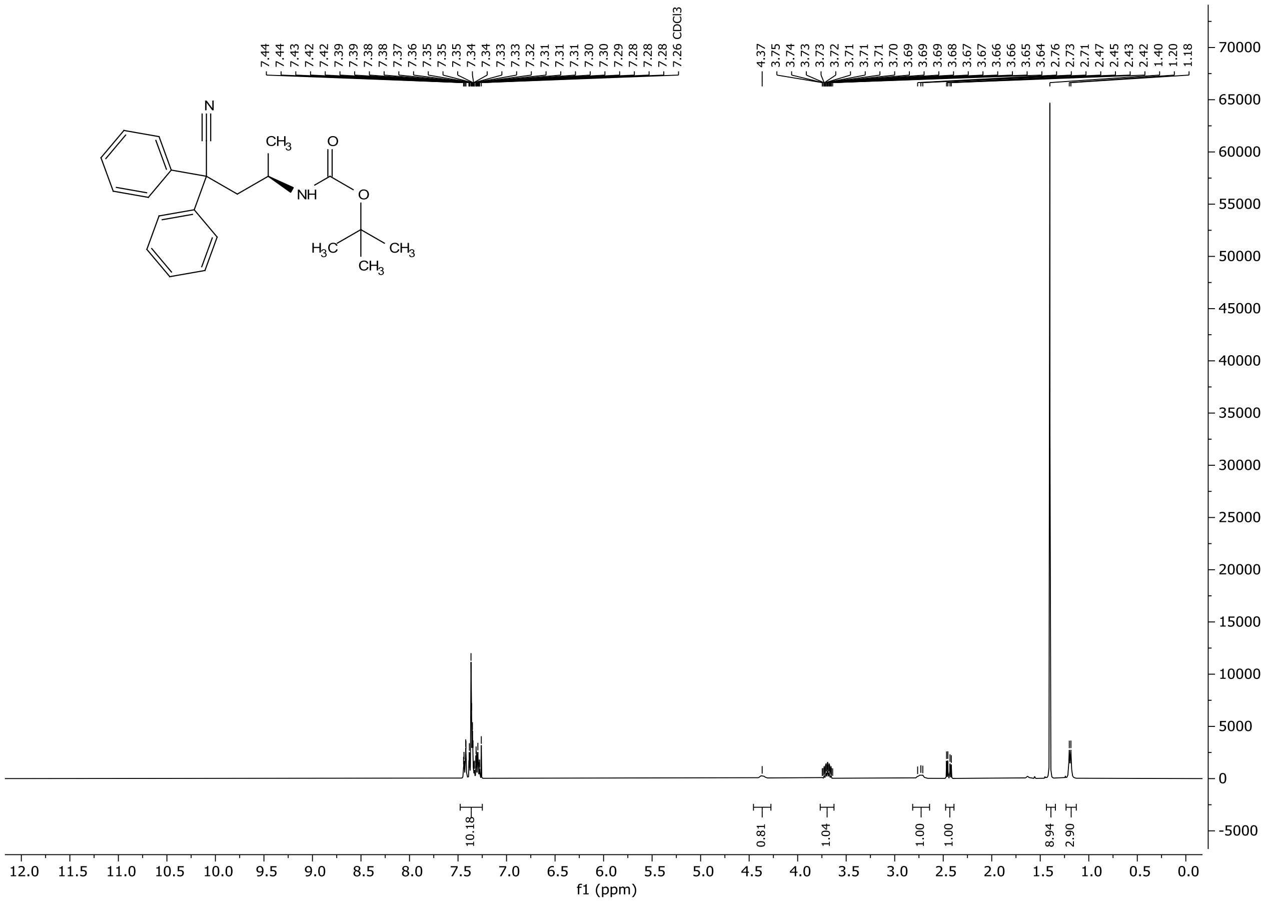

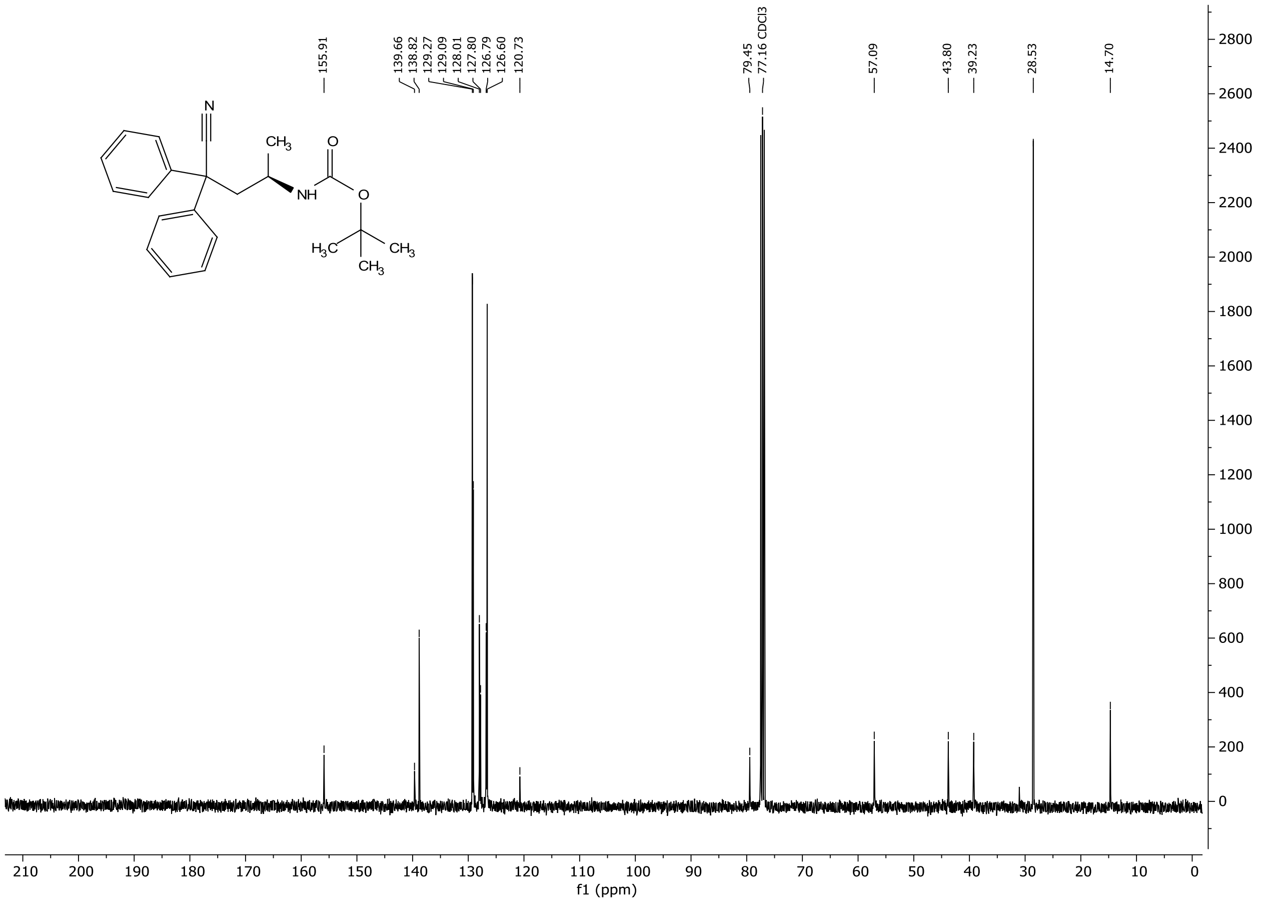

^{1}H and ^{13}C NMR spectra of (*S*)-tert-butyl (4-cyano-4,4-diphenylbutan-2-yl)carbamate ((*S*)-**3**).

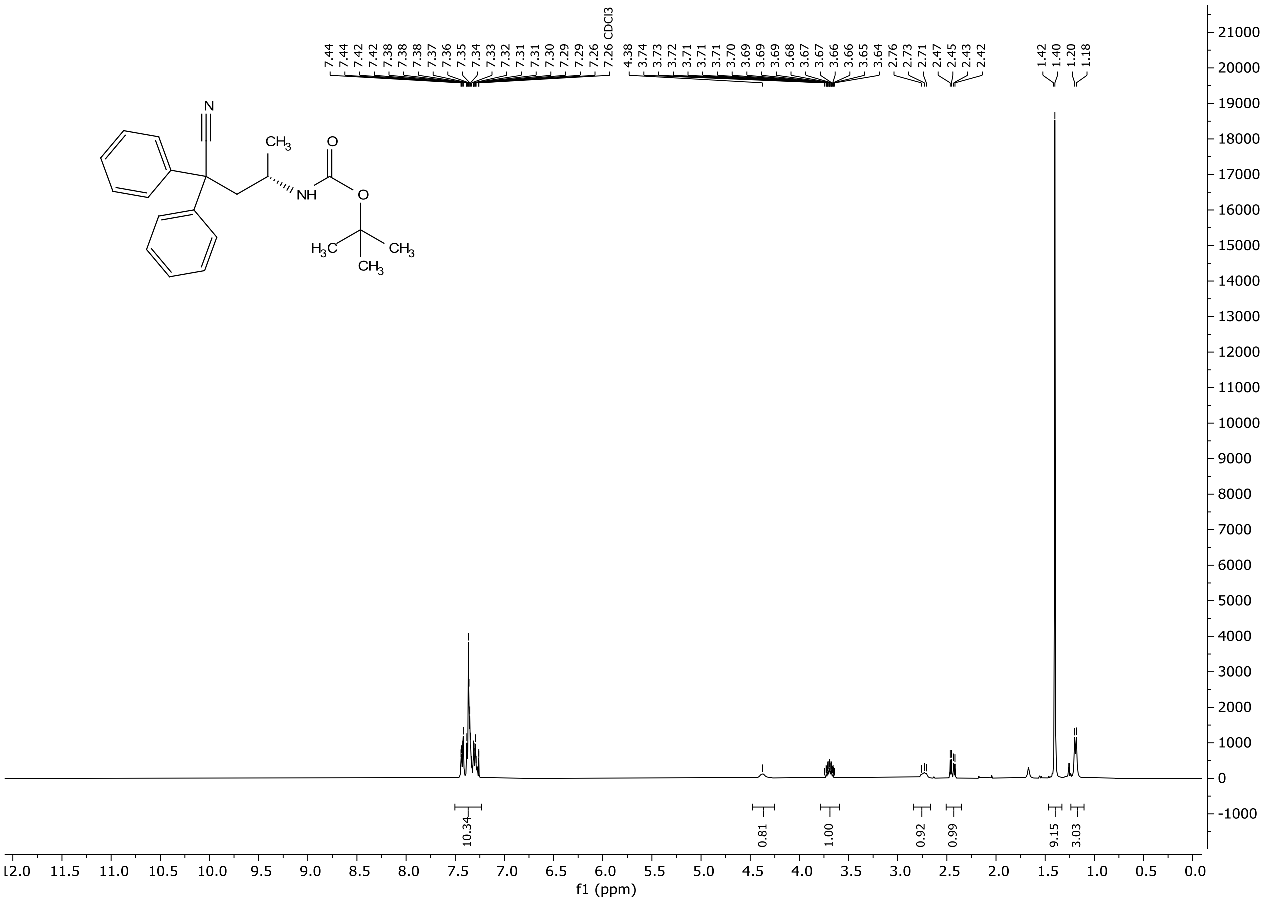

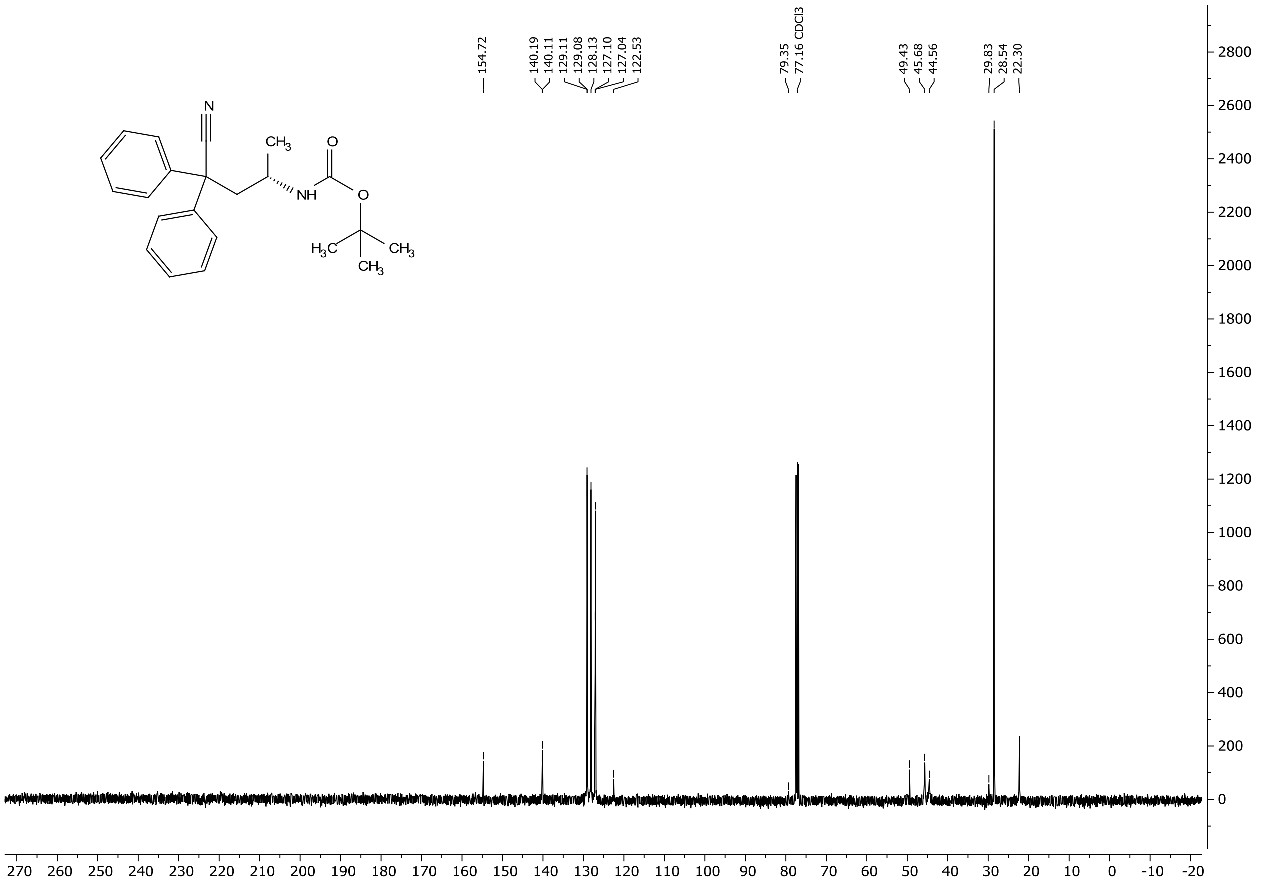

^1H and ^{13}C NMR spectra of (*R*)-4-(dimethylamino)-2,2-diphenylpentanenitrile ((*R*)-**4**).

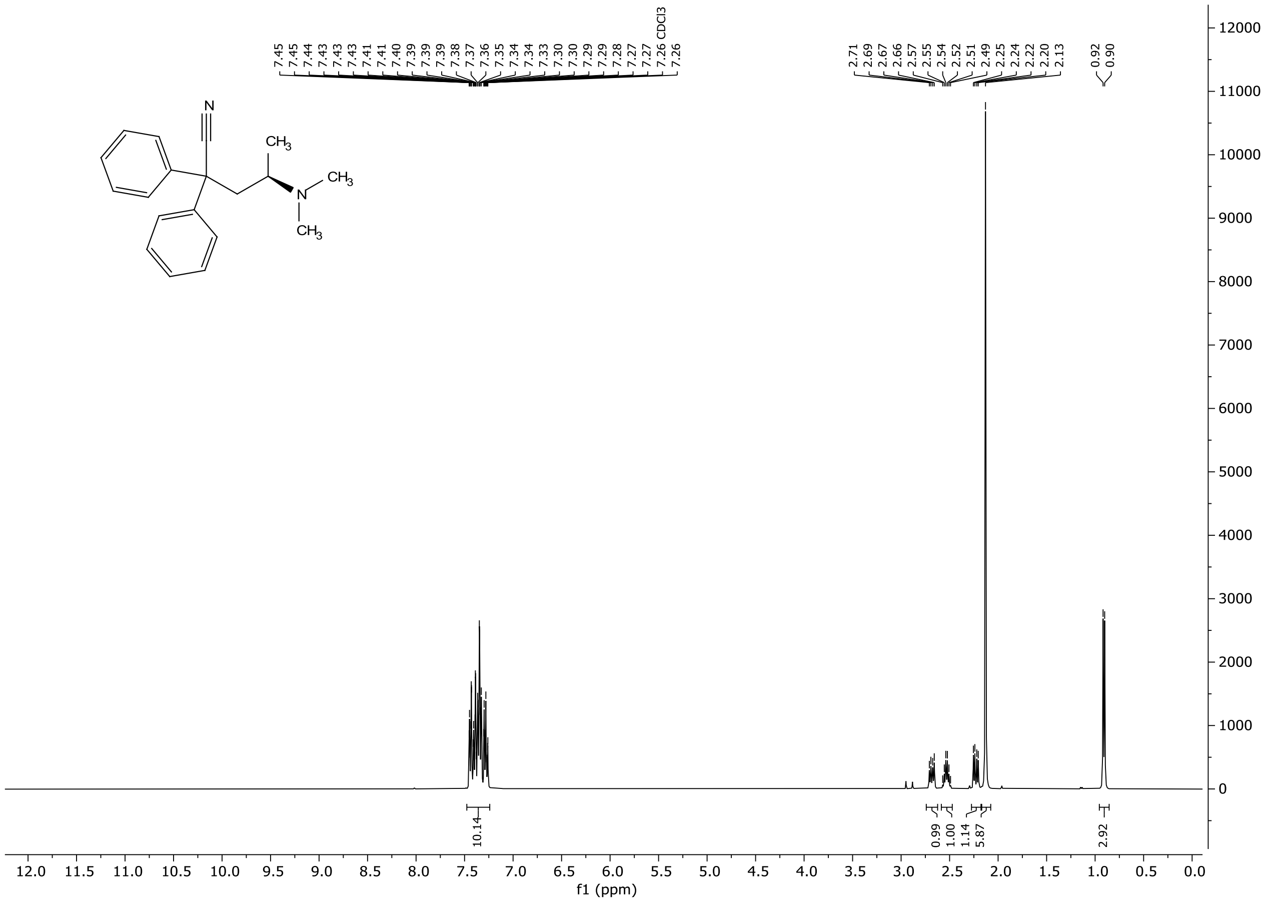

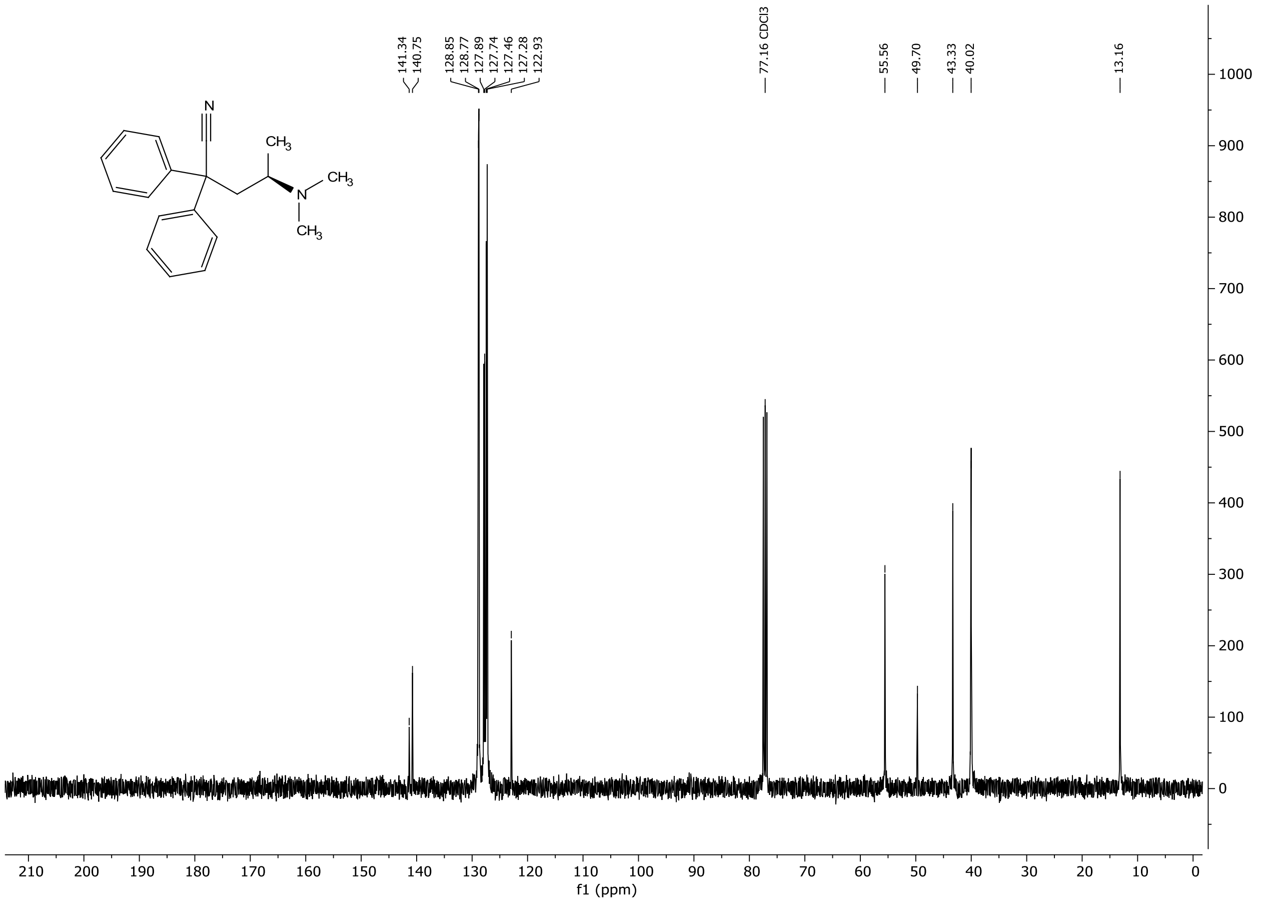

^{1}H and ^{13}C NMR spectra of (*S*)-4-(dimethylamino)-2,2-diphenylpentanenitrile ((*S*)-**4**).

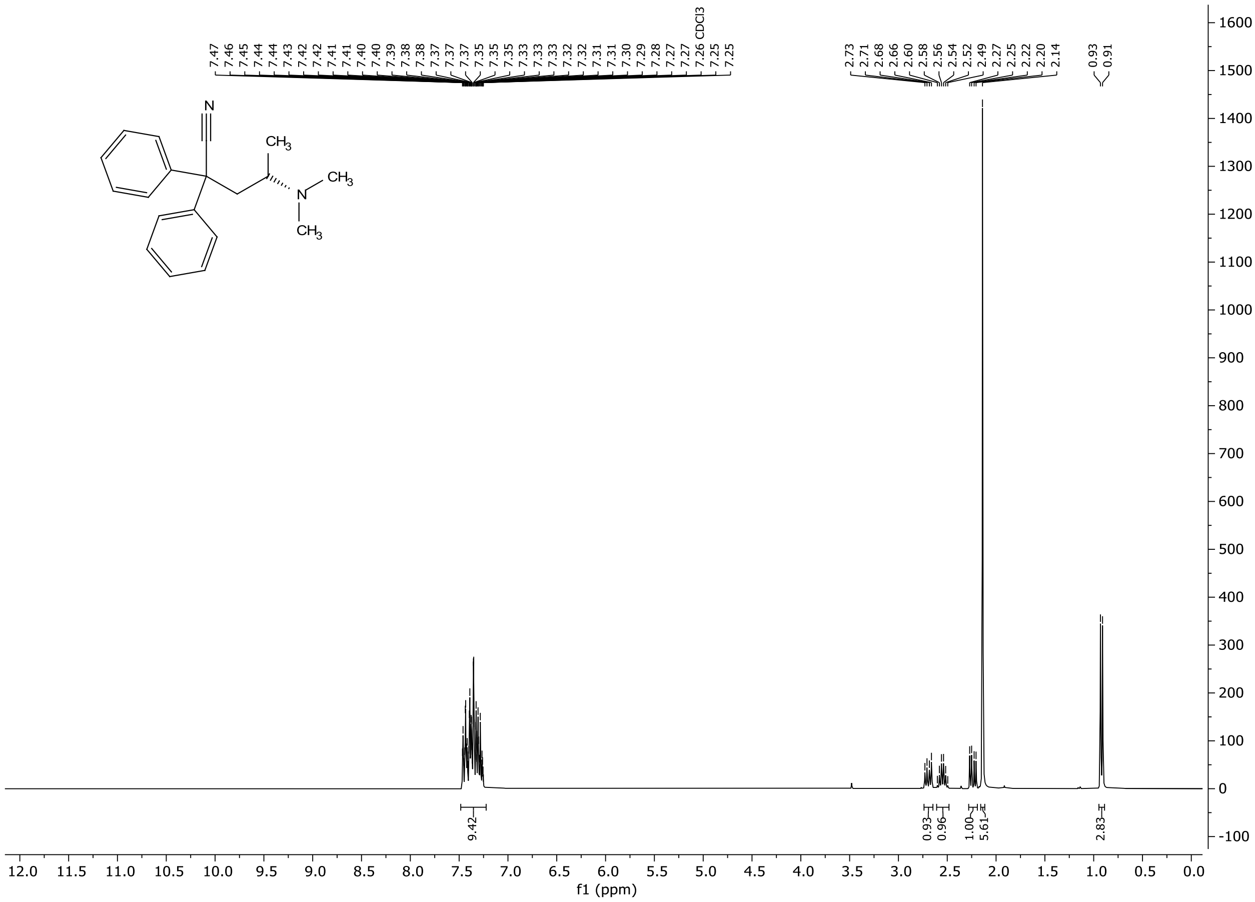

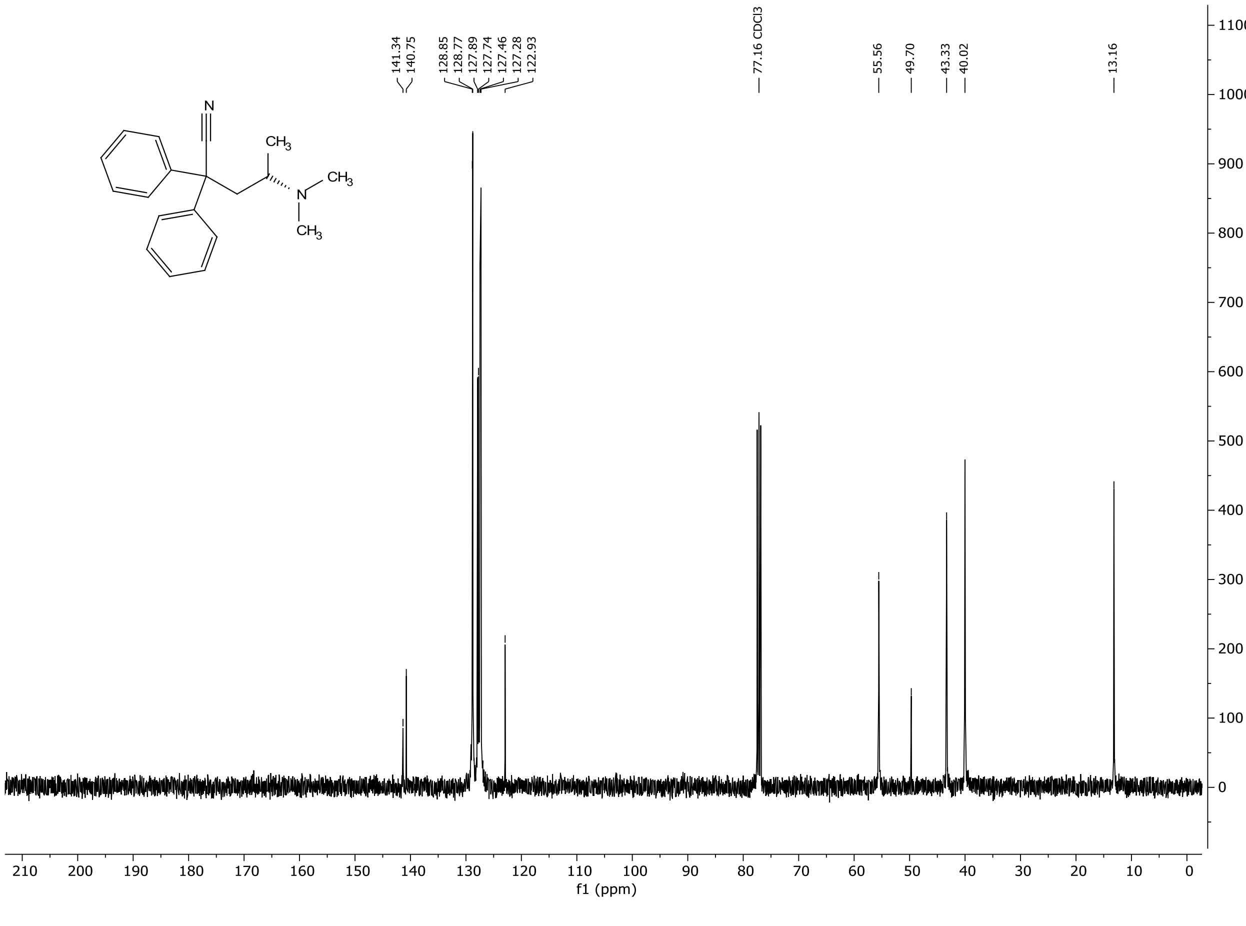

^{1}H and ^{13}C NMR spectra of (*R*)-6-(dimethylamino)-4,4-diphenylheptan-3-one ((*R*)-**methadone**).

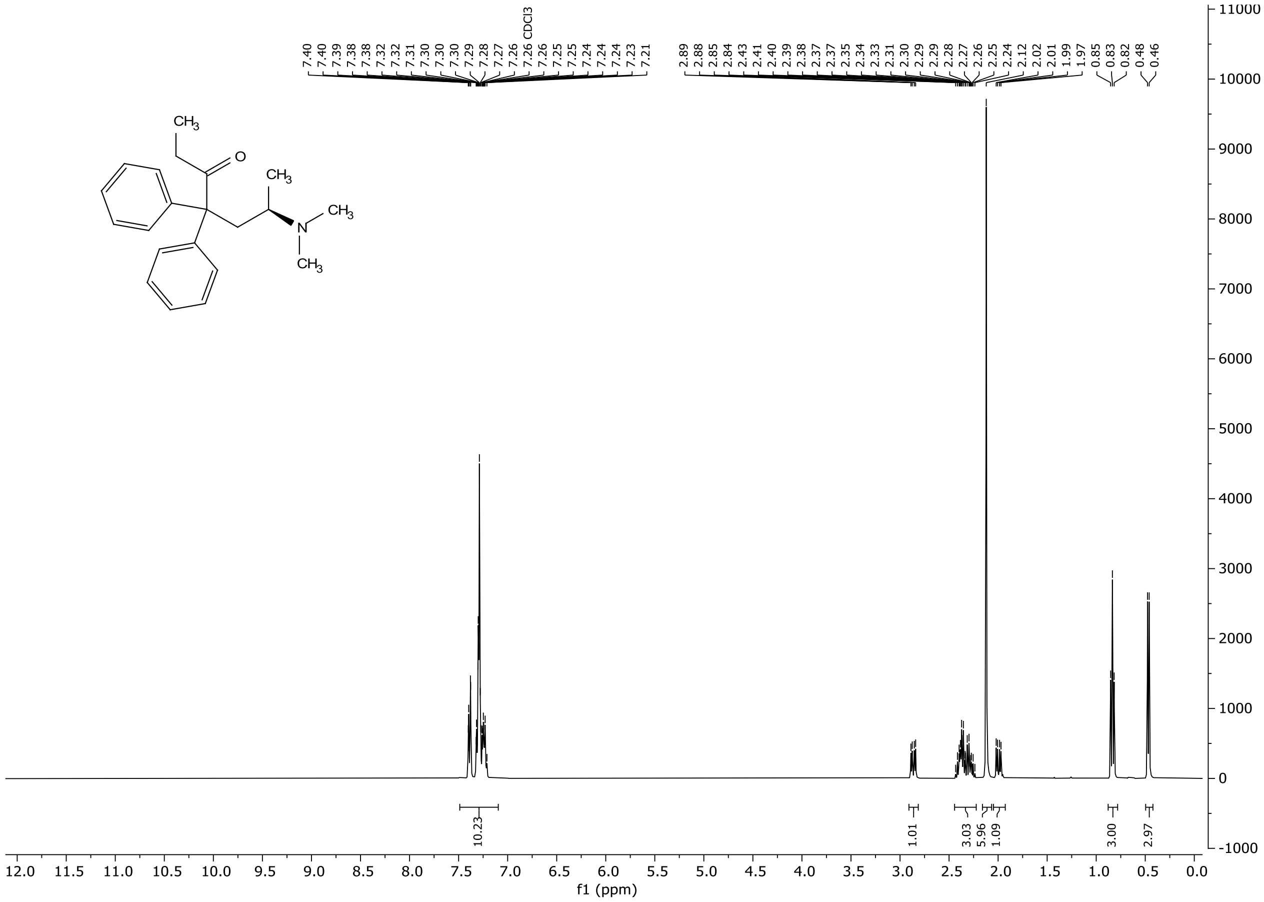

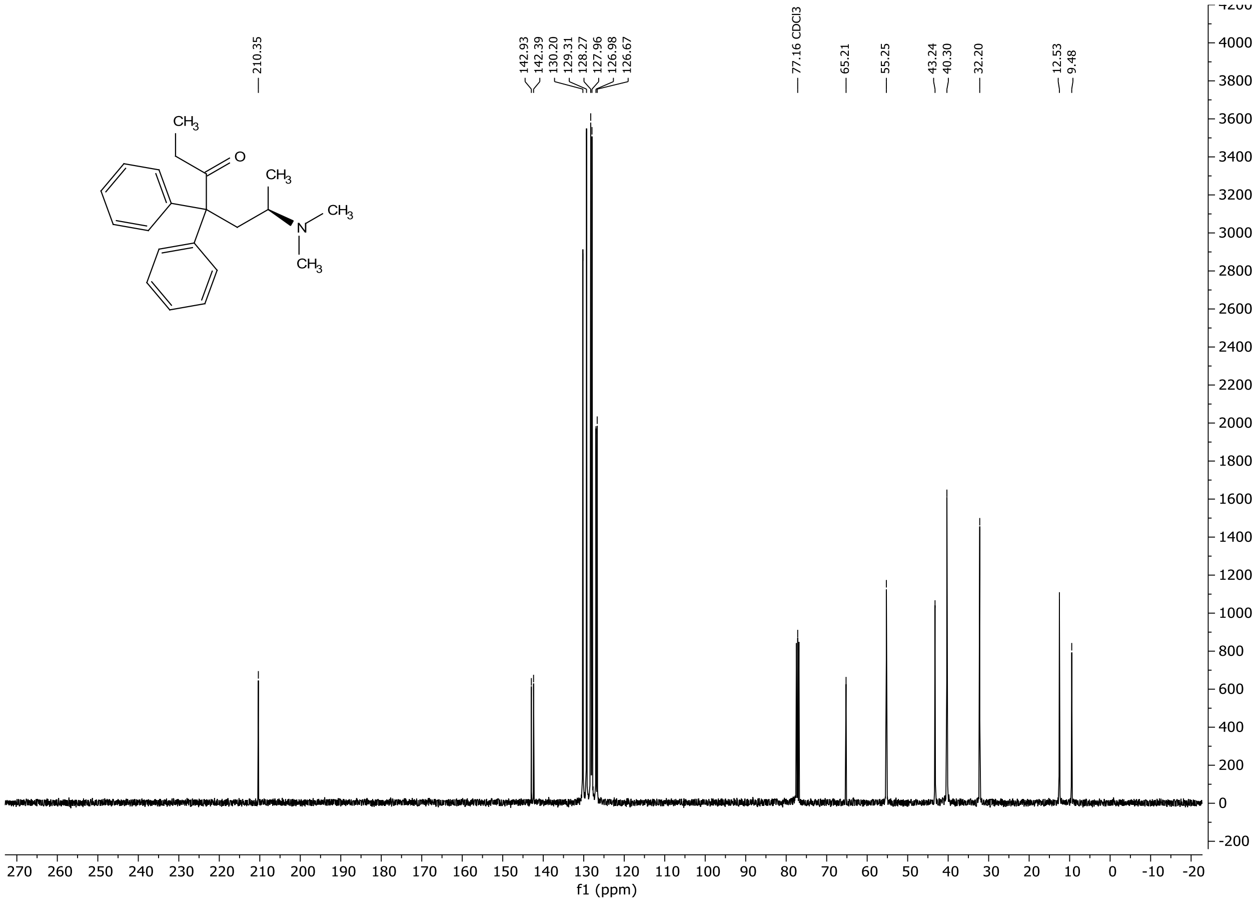

Blank run UPLC

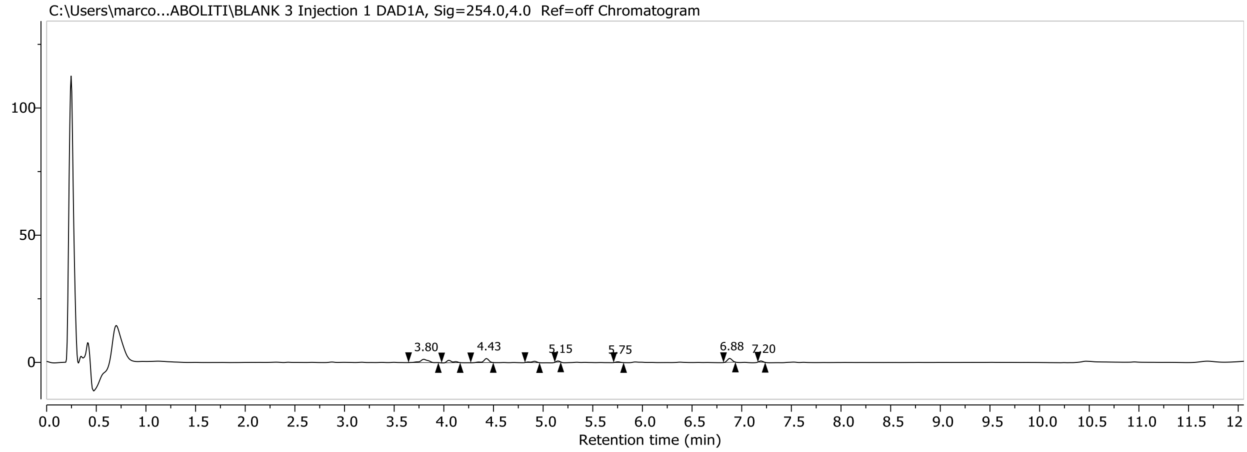

UPLC chromatogram of (*R*)-6-(dimethylamino)-4,4-diphenylheptan-3-one ((*R*)-**methadone**).

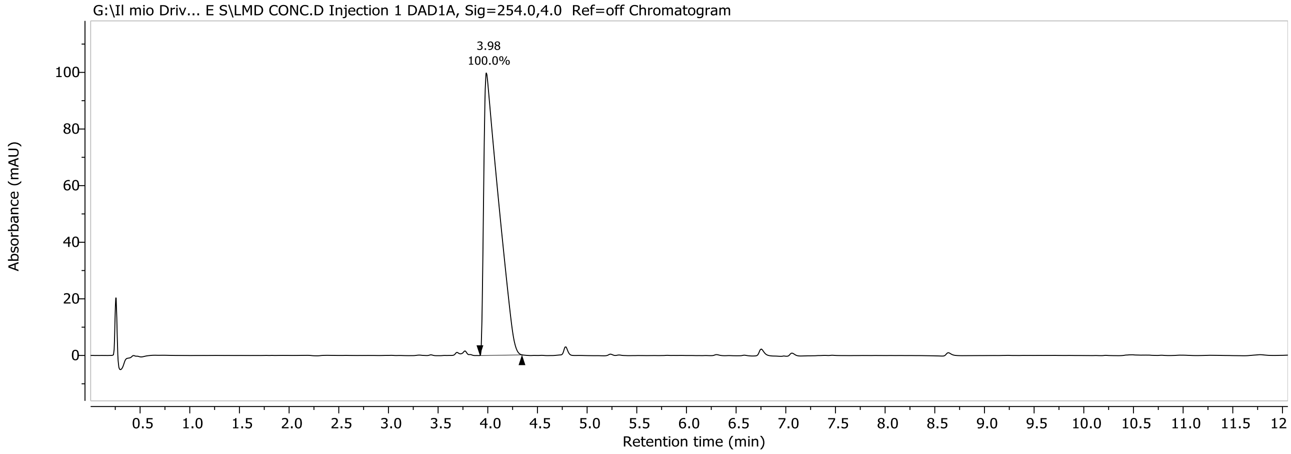

Chiral HPLC chromatogram of (*R*)-6-(dimethylamino)-4,4-diphenylheptan-3-one ((*R*)-**methadone**).

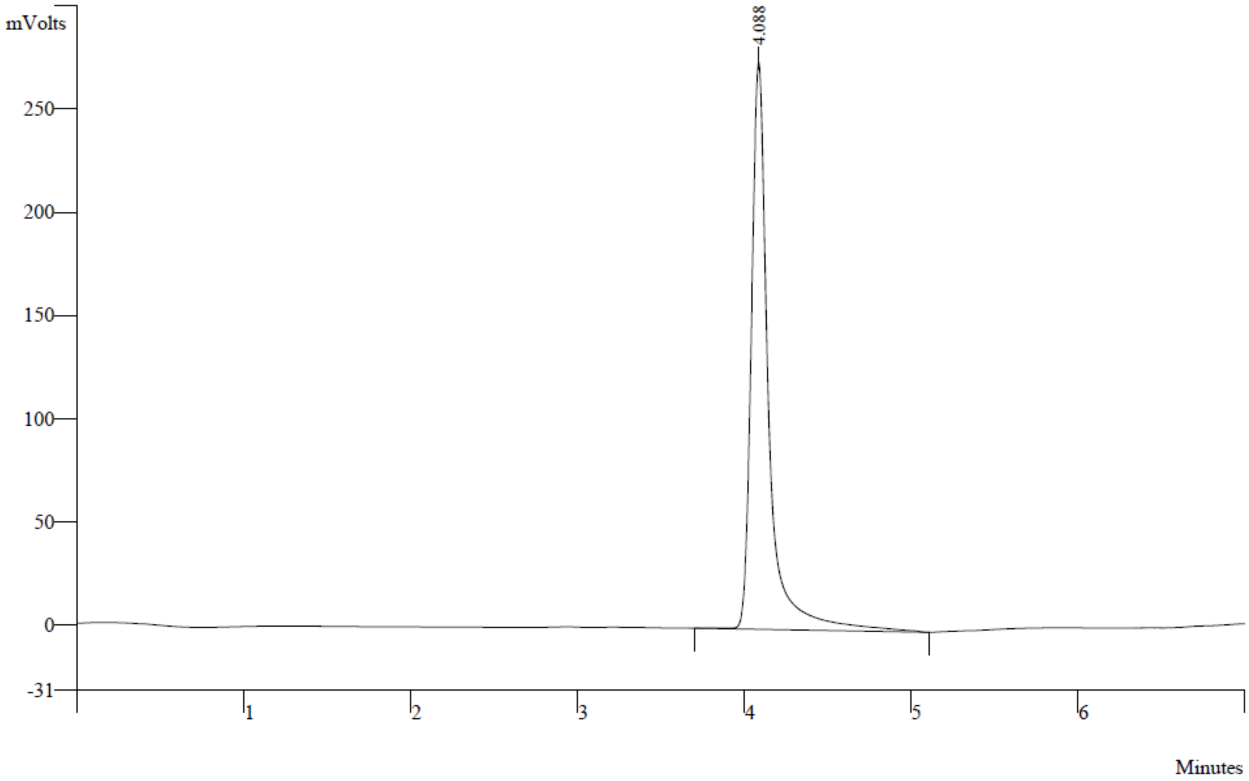

Peak No	Ret Time (min)	Peak Area (counts)	Result ()
1	4,088	1996720	100,00
		**1996720**	**100,00**

^{1}H and ^{13}C NMR spectra of (*S*)-6-(dimethylamino)-4,4-diphenylheptan-3-one ((*S*)-**methadone**).

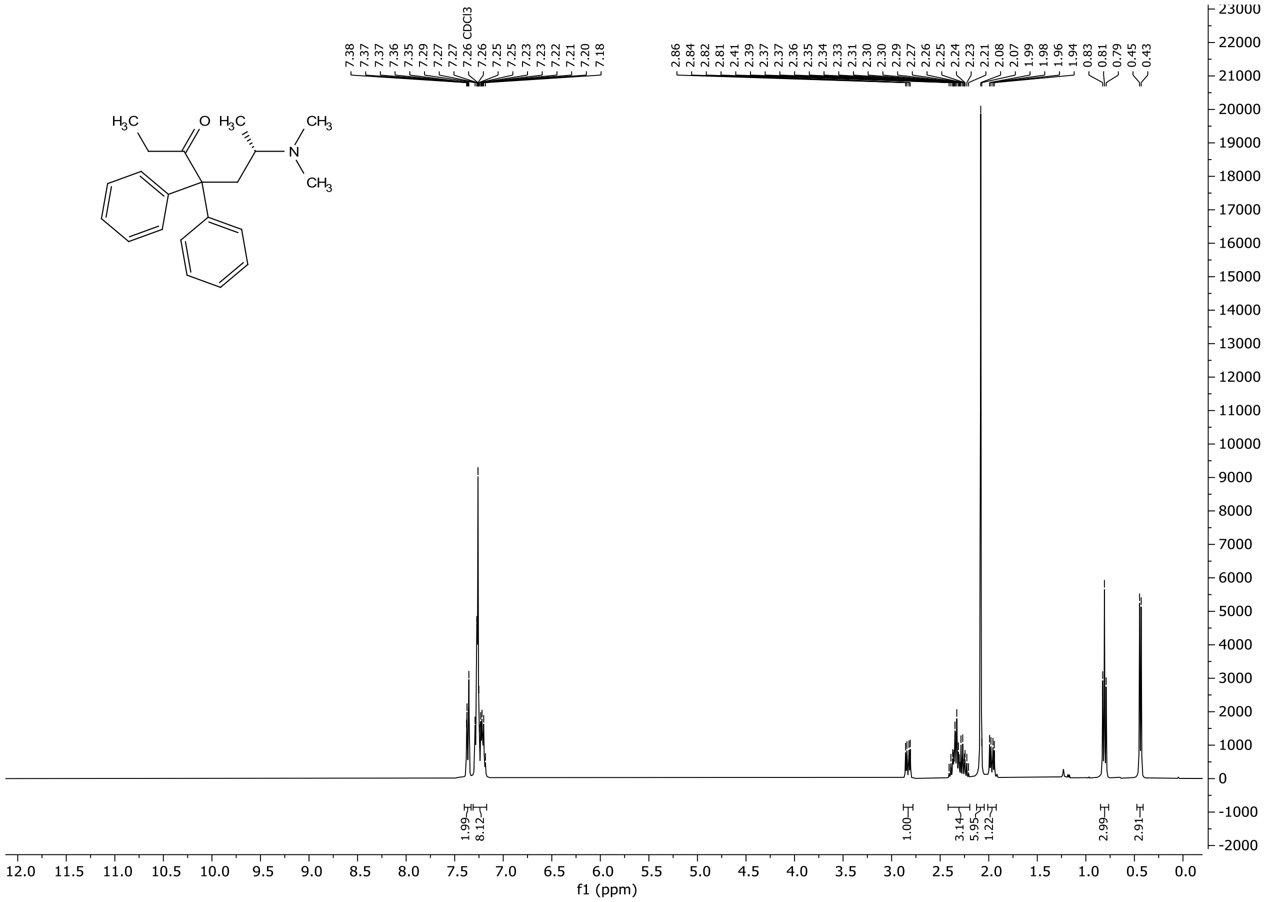

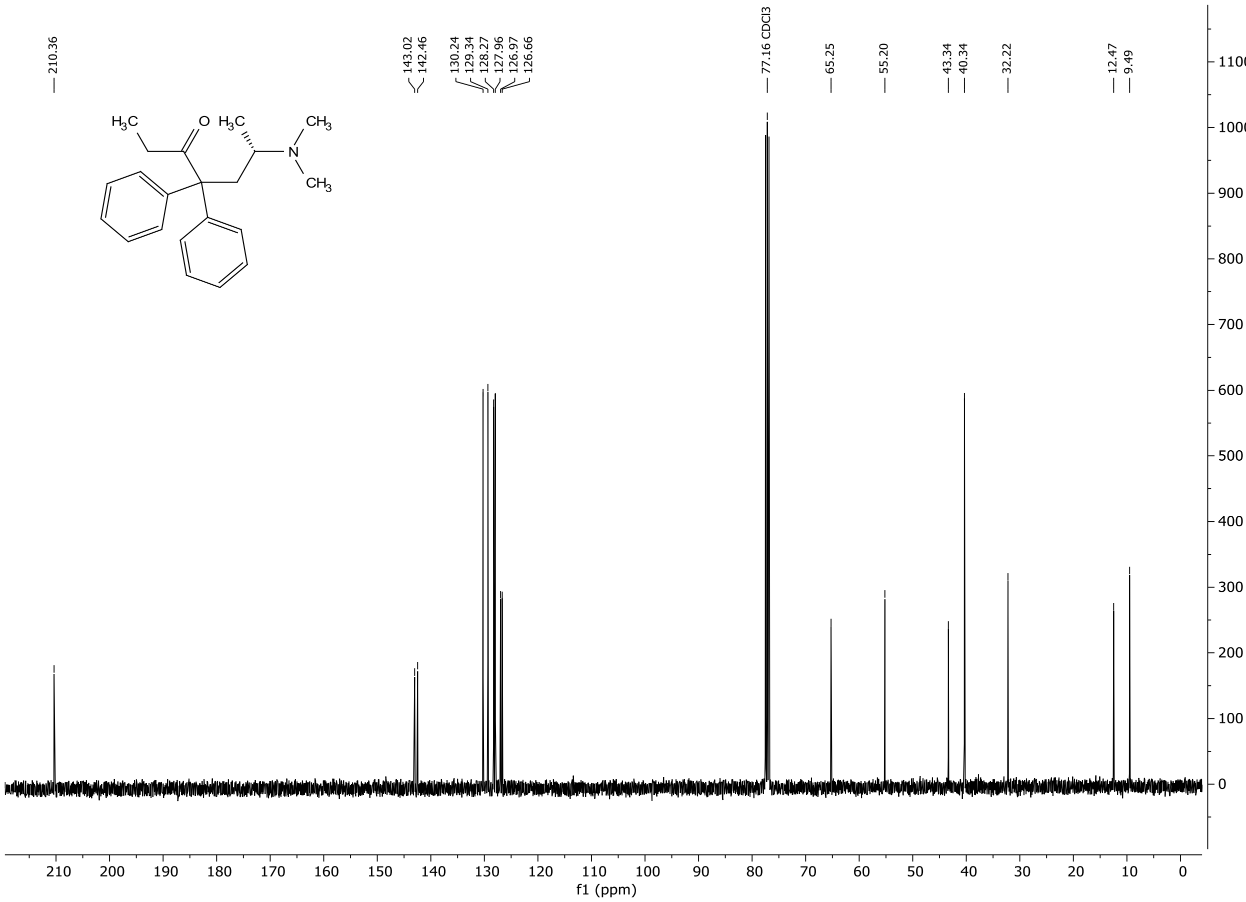

UPLC chromatogram of (*S*)-6-(dimethylamino)-4,4-diphenylheptan-3-one ((*S*)-**methadone**).

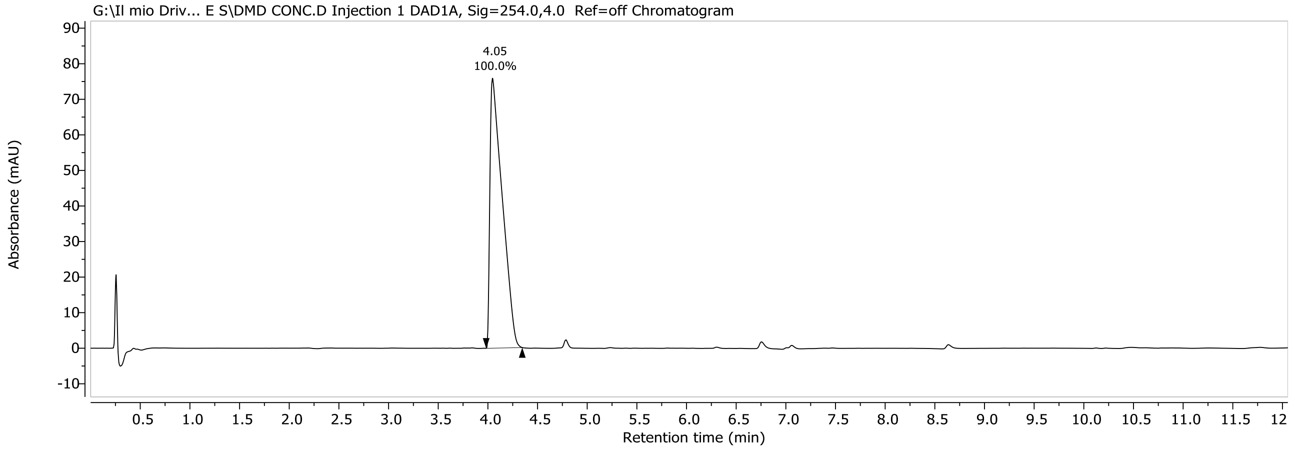

Chiral HPLC chromatogram of (*S*)-6-(dimethylamino)-4,4-diphenylheptan-3-one ((*S*)-**methadone**).

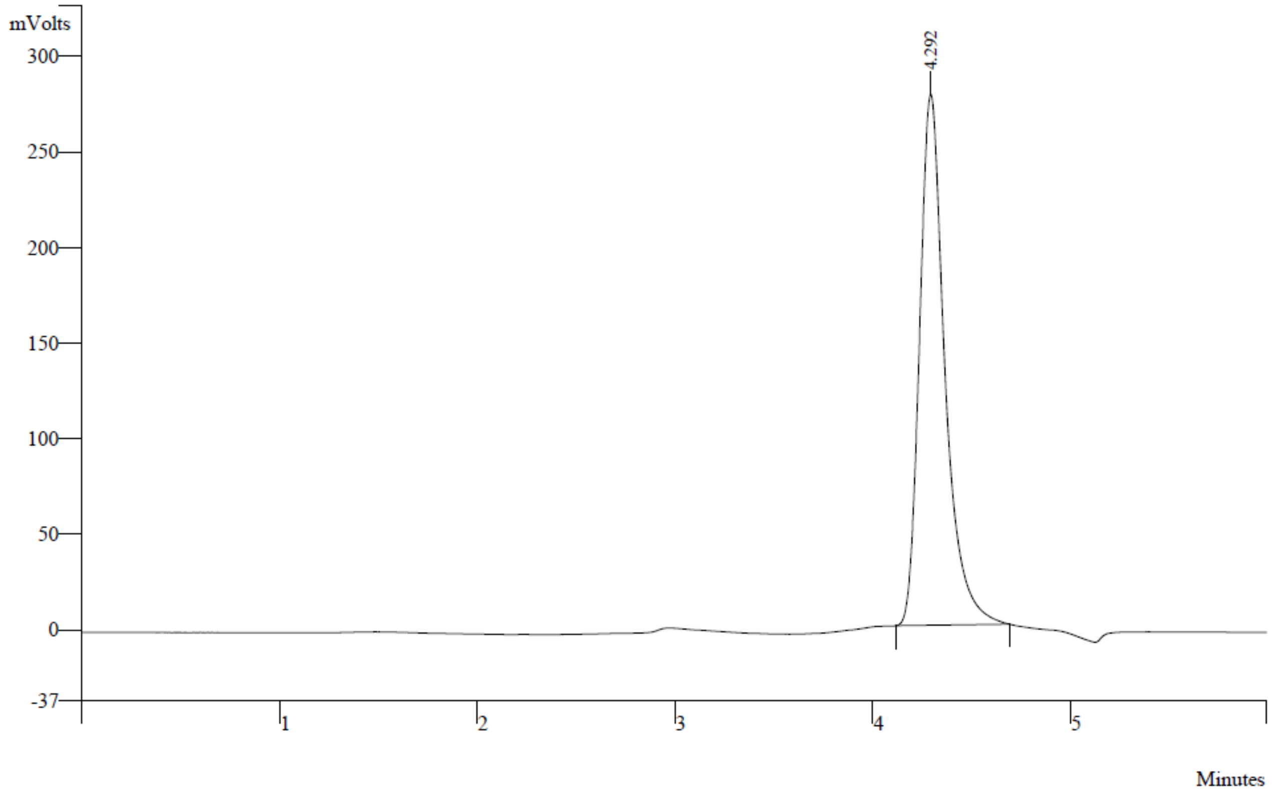

Peak No	Ret Time (min)	Peak Area (counts)	Result ()
1	4,292	2478807	100,00
		**2478807**	**100,00**

^{1}H and ^{13}C NMR spectra of (*R*)-4-(dimethylamino)-2,2-diphenylpentanal ((*R*)-**5**).

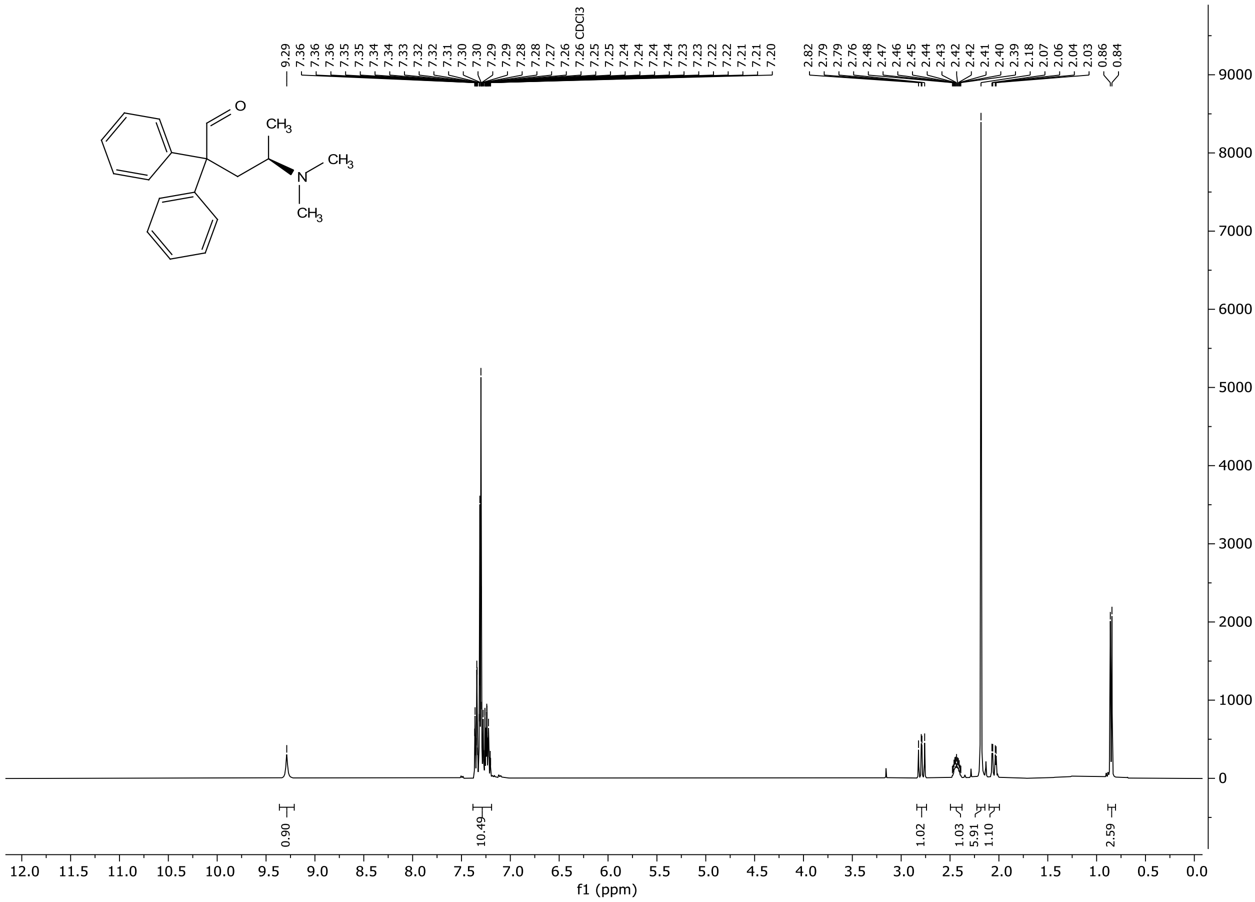

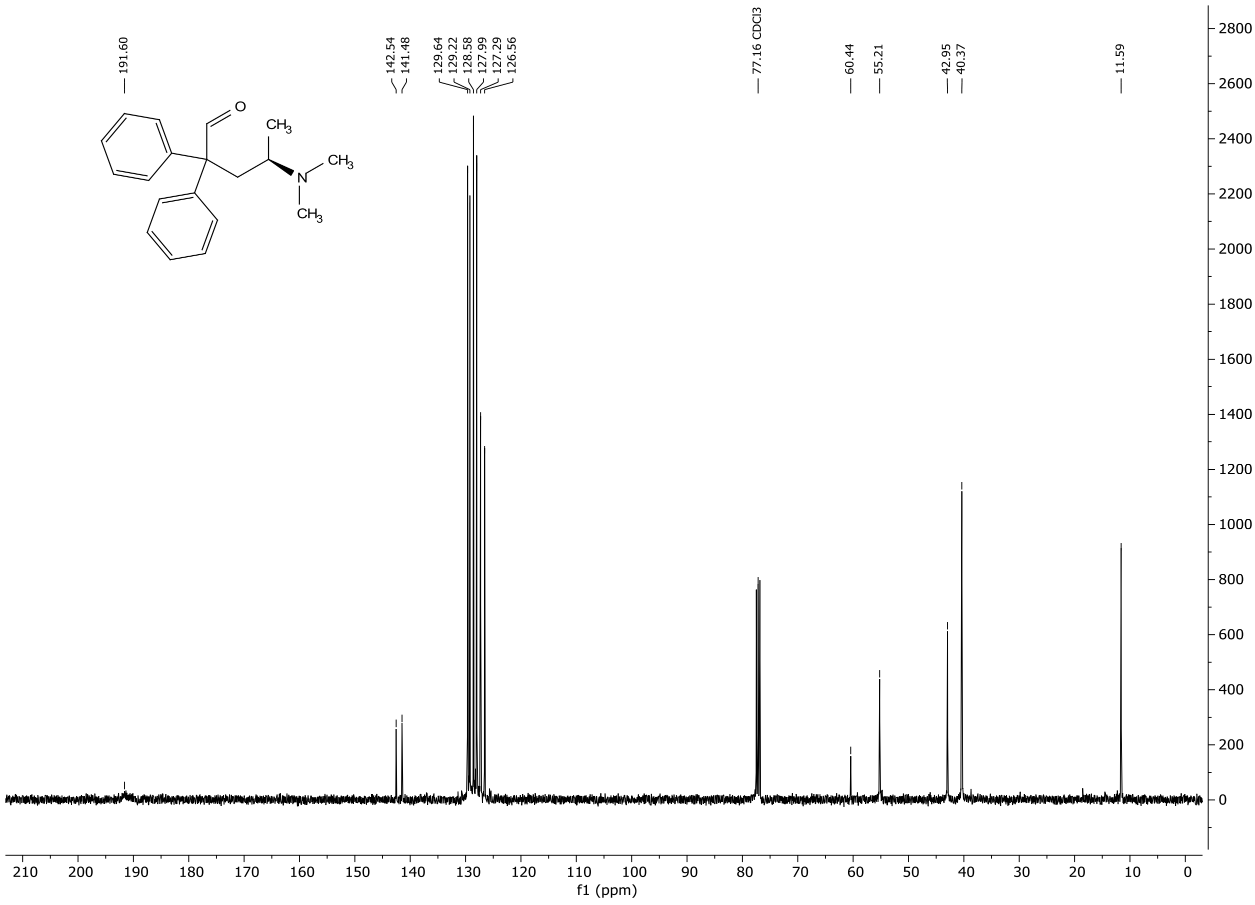

^{1}H and ^{13}C NMR spectra of (*S*)-4-(dimethylamino)-2,2-diphenylpentanal ((*S*)-**5**).

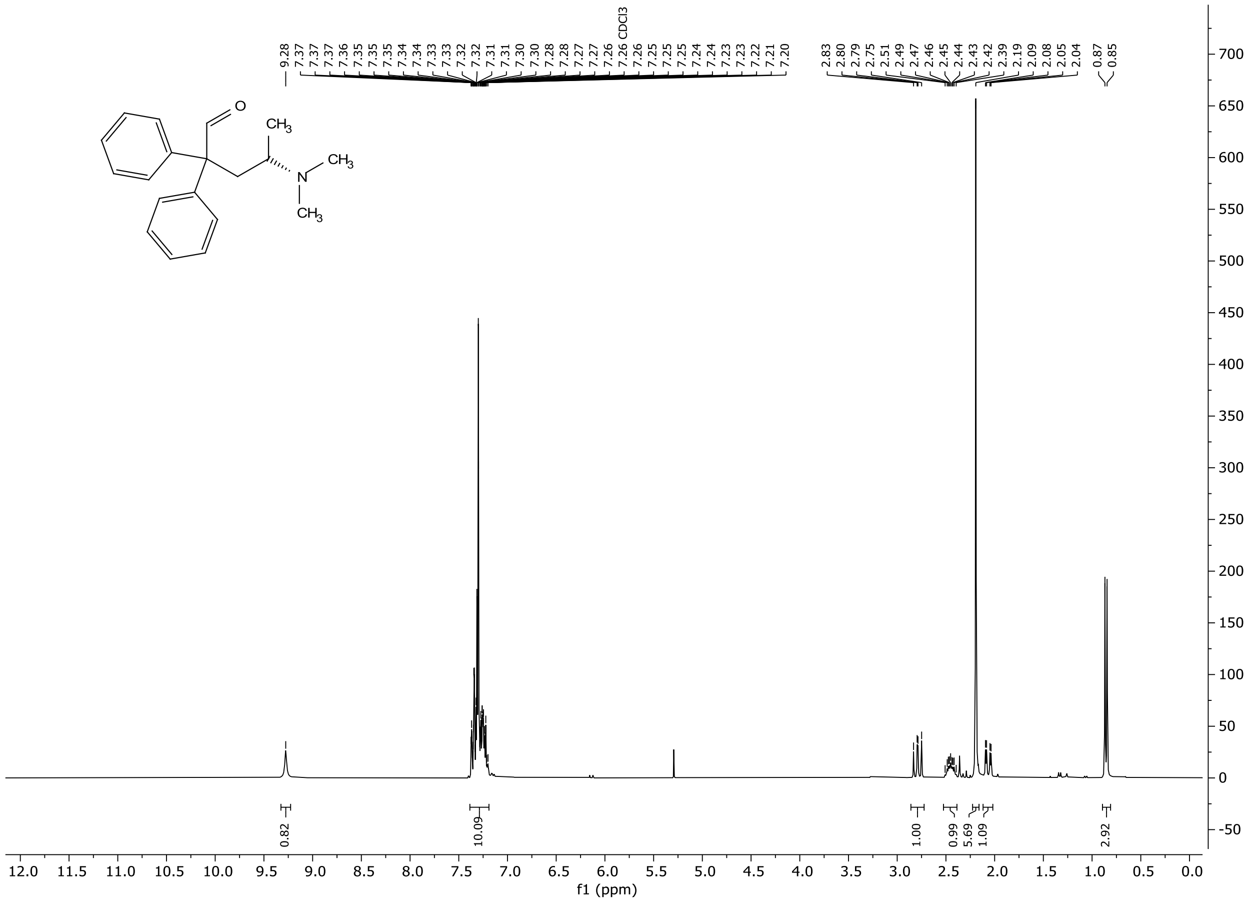

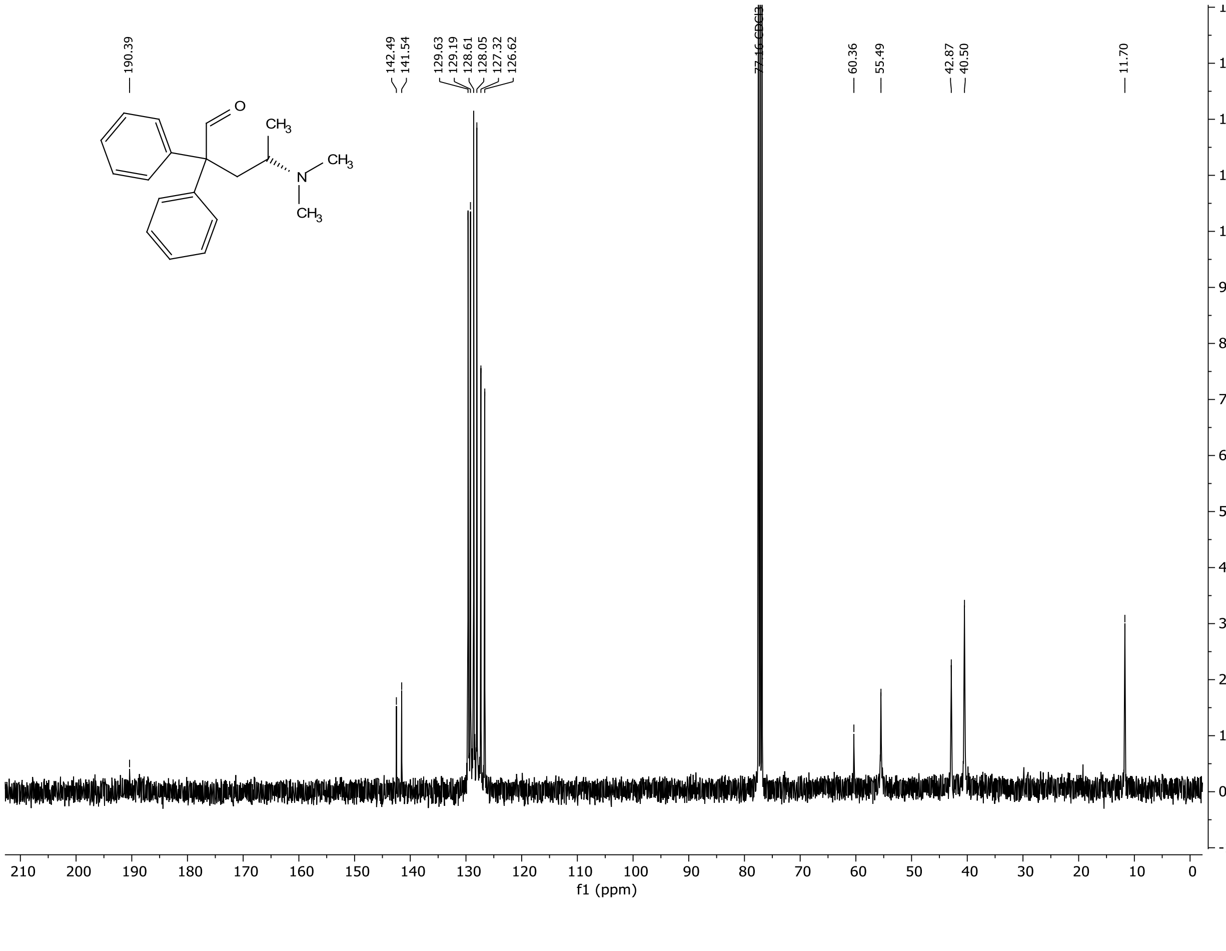

^{1}H and ^{13}C NMR spectra of (*R*)-4-(dimethylamino)-2,2-diphenylpentanoic acid ((*R*)-**DDVA**).

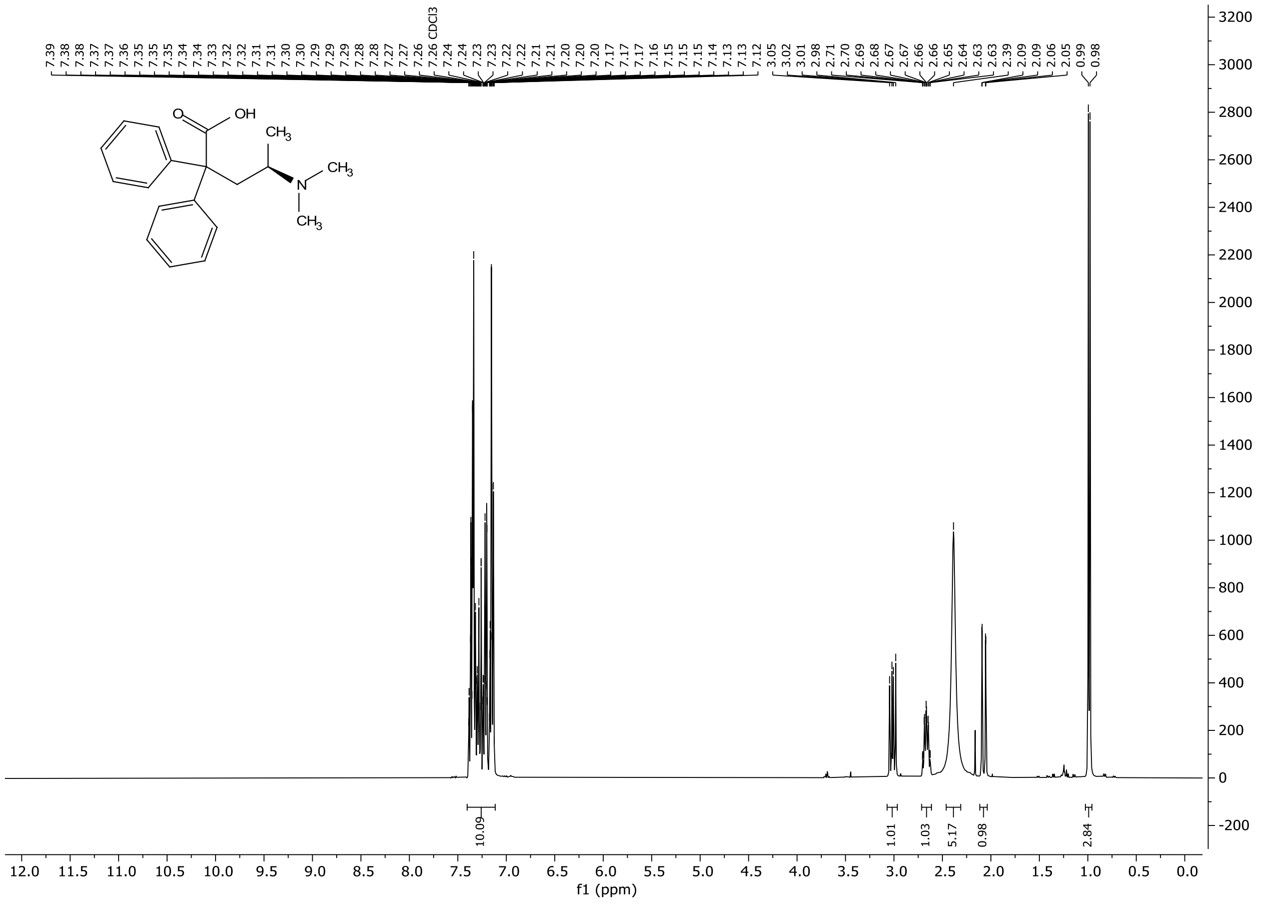

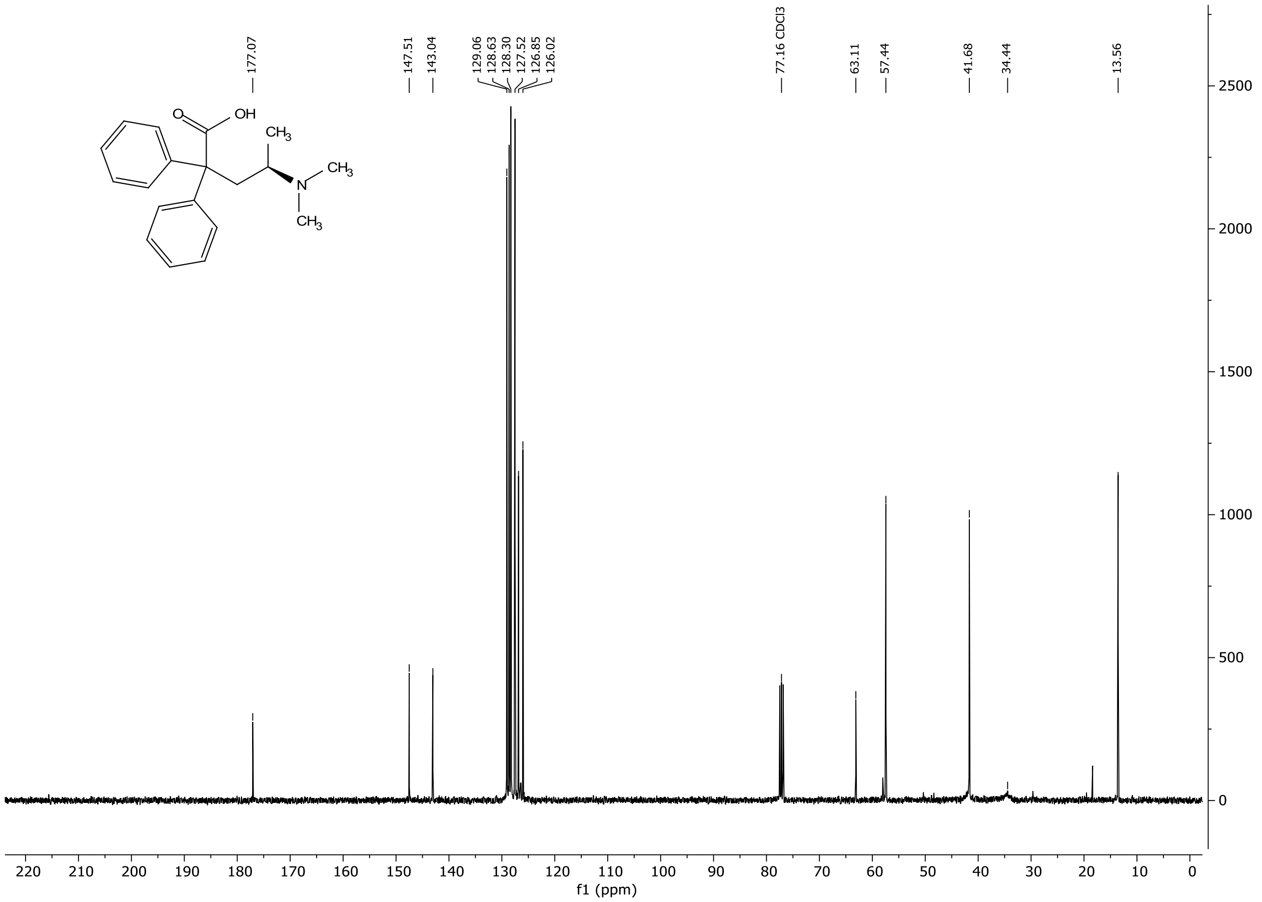

UPLC chromatogram of (*R*)-4-(dimethylamino)-2,2-diphenylpentanoic acid ((*R*)-**DDVA**).

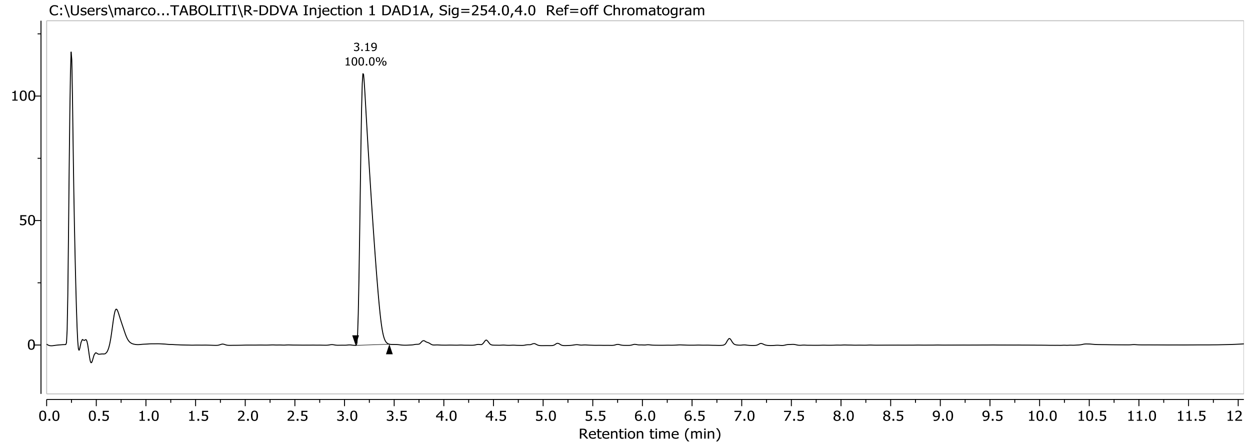

Chiral HPLC chromatogram of (*R*)-4-(dimethylamino)-2,2-diphenylpentanoic acid ((*R*)-**DDVA**).

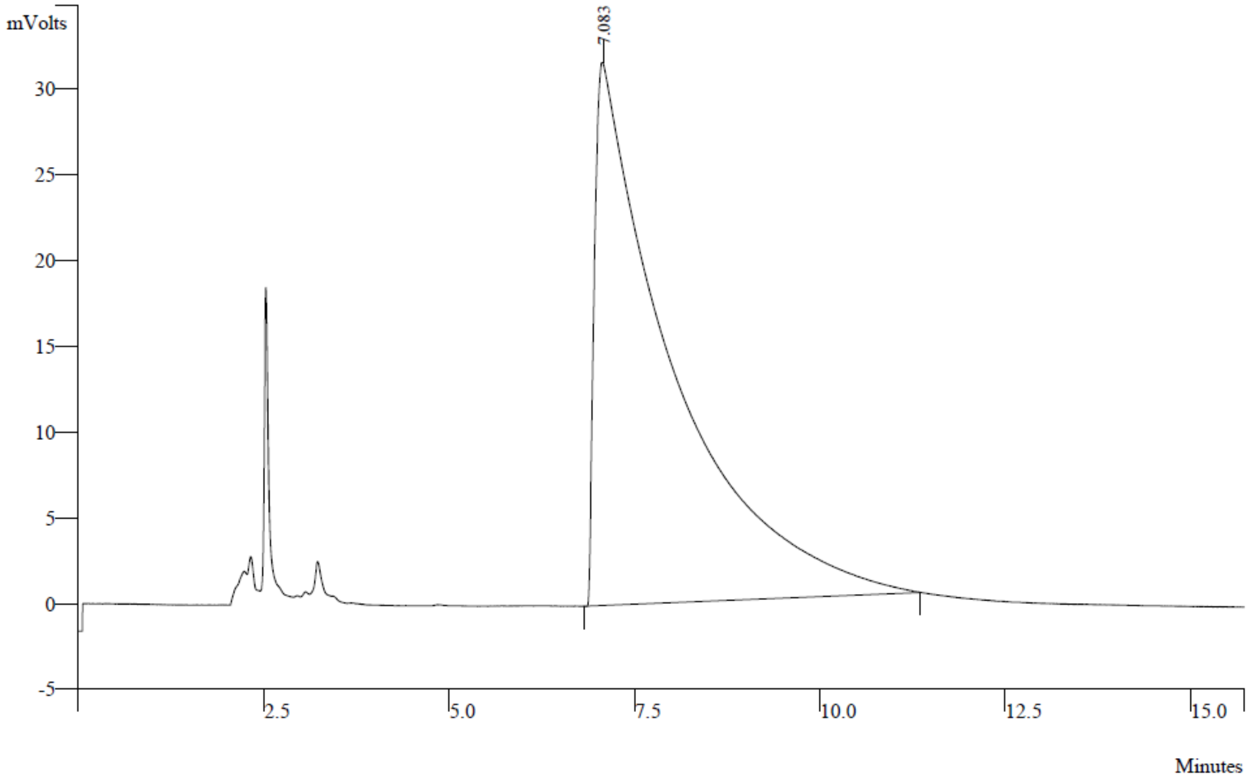

Peak No	Ret Time (min)	Peak Area (counts)	Result ()
1	7,083	2281372	100,00
		**2281372**	**100,00**

^{1}H and ^{13}C NMR spectra of (*S*)-4-(dimethylamino)-2,2-diphenylpentanoic acid ((*S*)-**DDVA**).

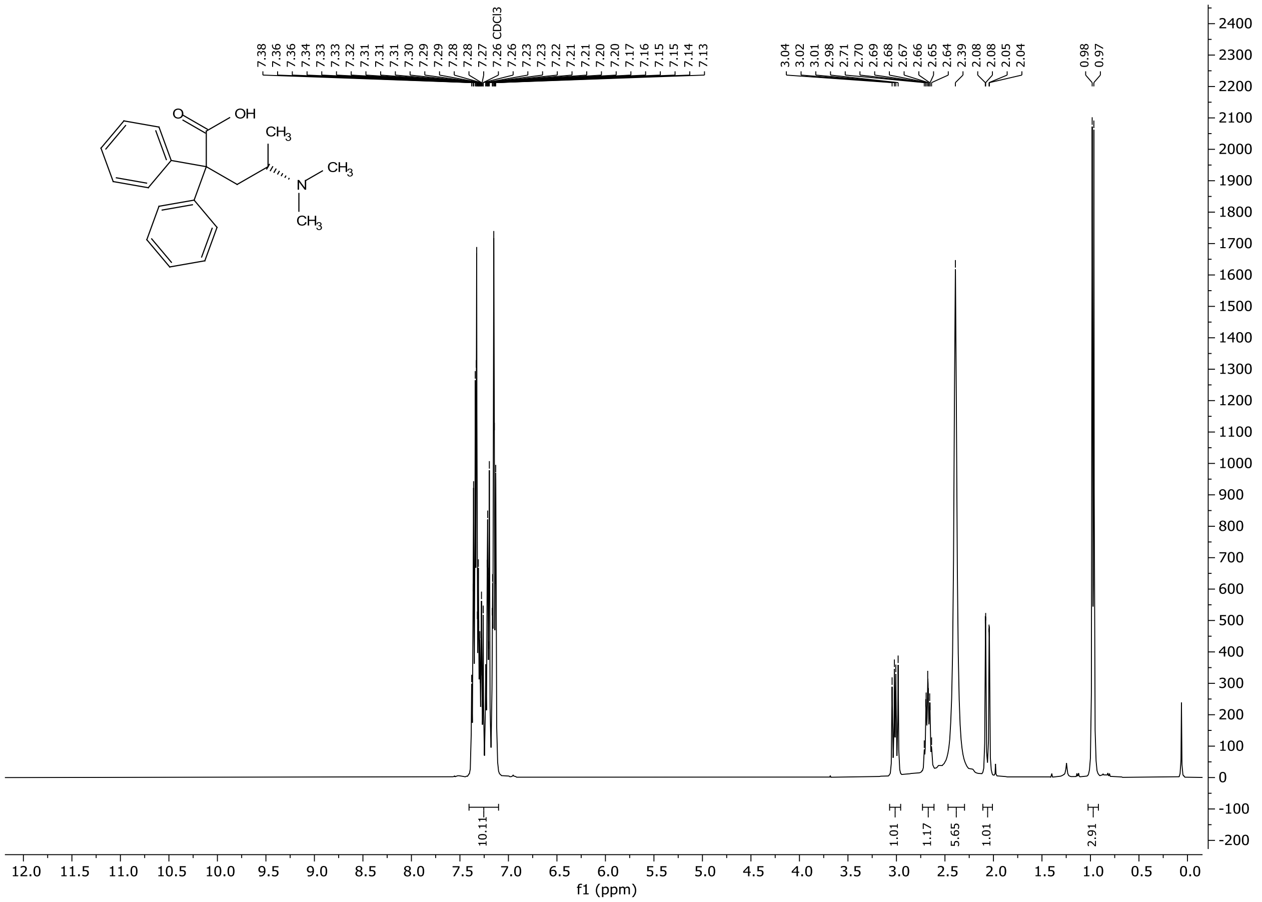

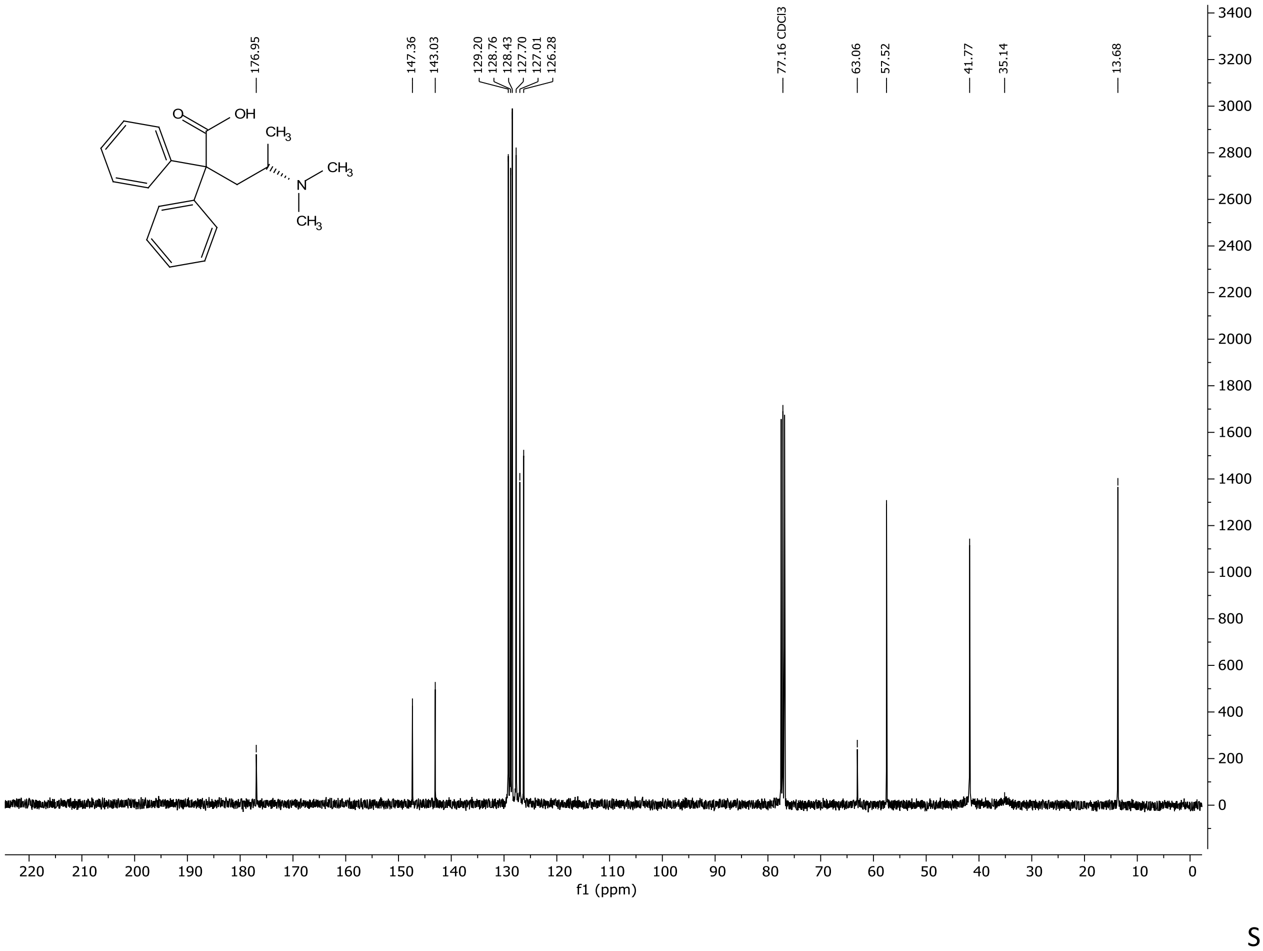

UPLC chromatogram of (*R*)-4-(dimethylamino)-2,2-diphenylpentanoic acid ((*S*)-**DDVA**).

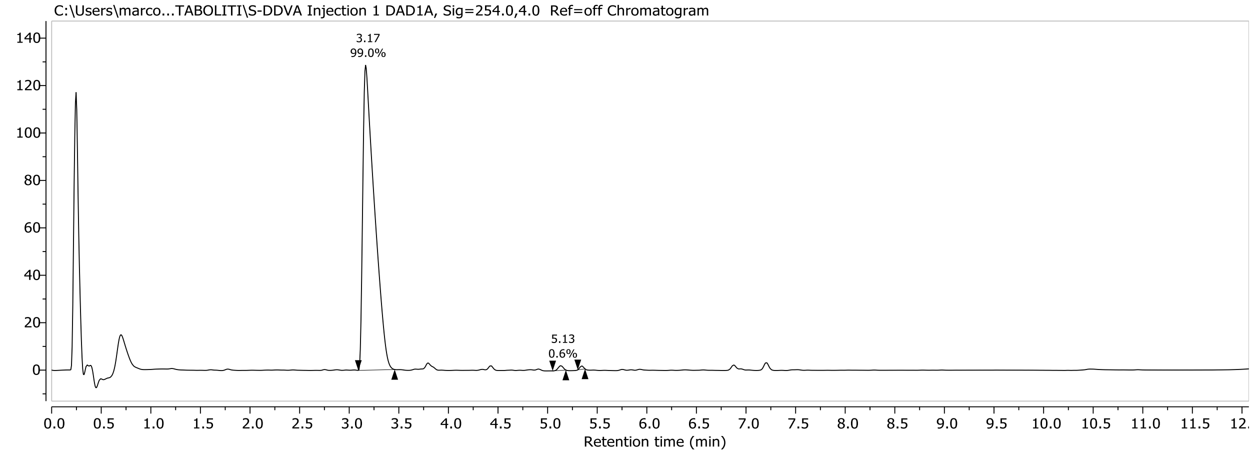

Chiral HPLC chromatogram of (*R*)-4-(dimethylamino)-2,2-diphenylpentanoic acid ((*S*)-**DDVA**).

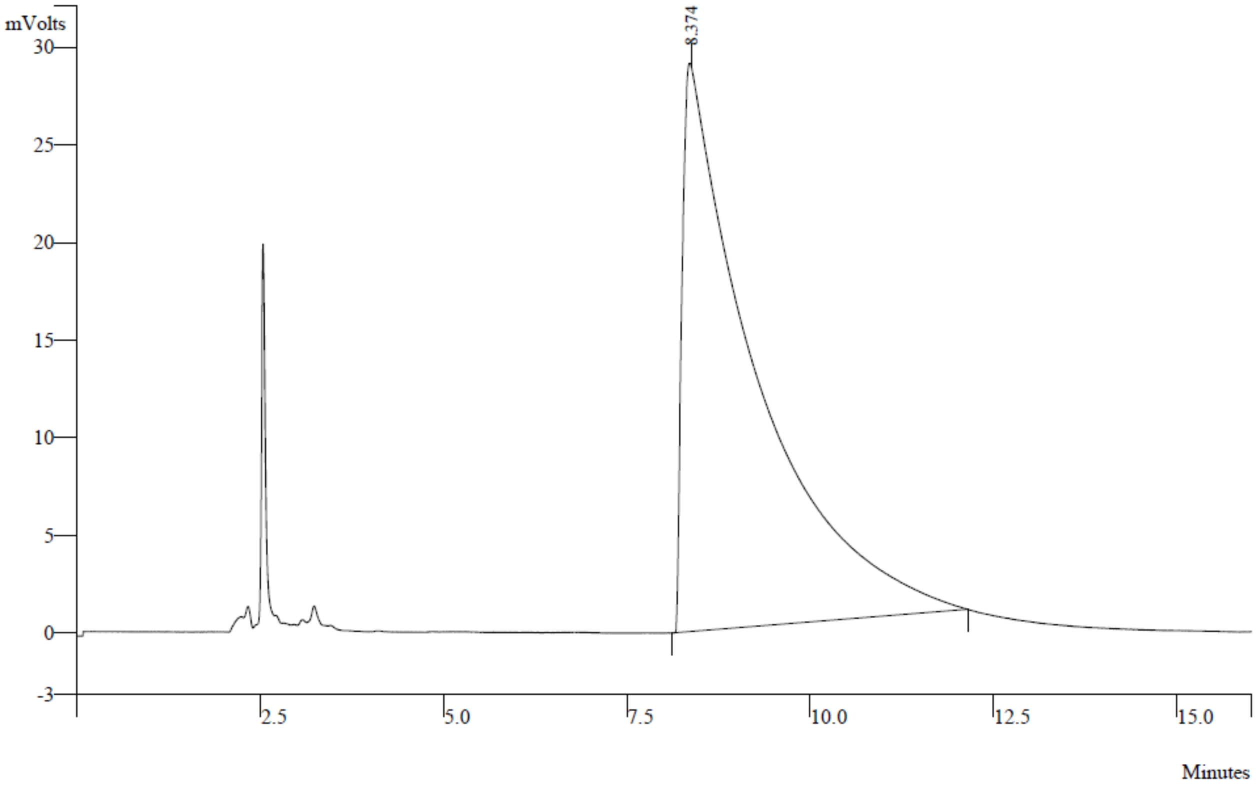

Peak No	Ret Time (min)	Peak Area (counts)	Result ()
1	8,374	2023526	100,00
		**2023526**	**100,00**

^{1}H and ^{13}C NMR spectra of (*R*)-1,5-dimethyl-3,3-diphenylpyrrolidin-2-one ((*R*)-**DDPO**).

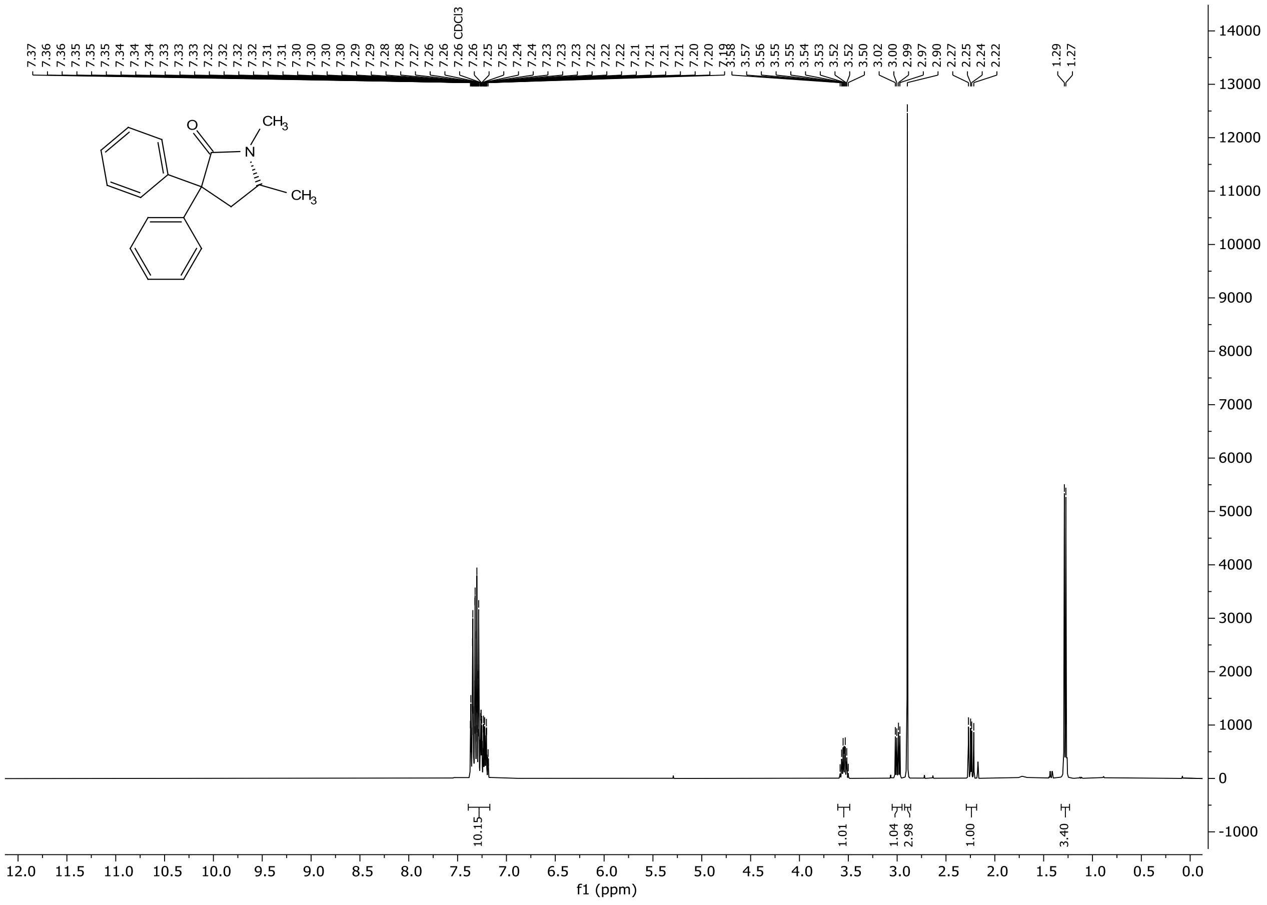

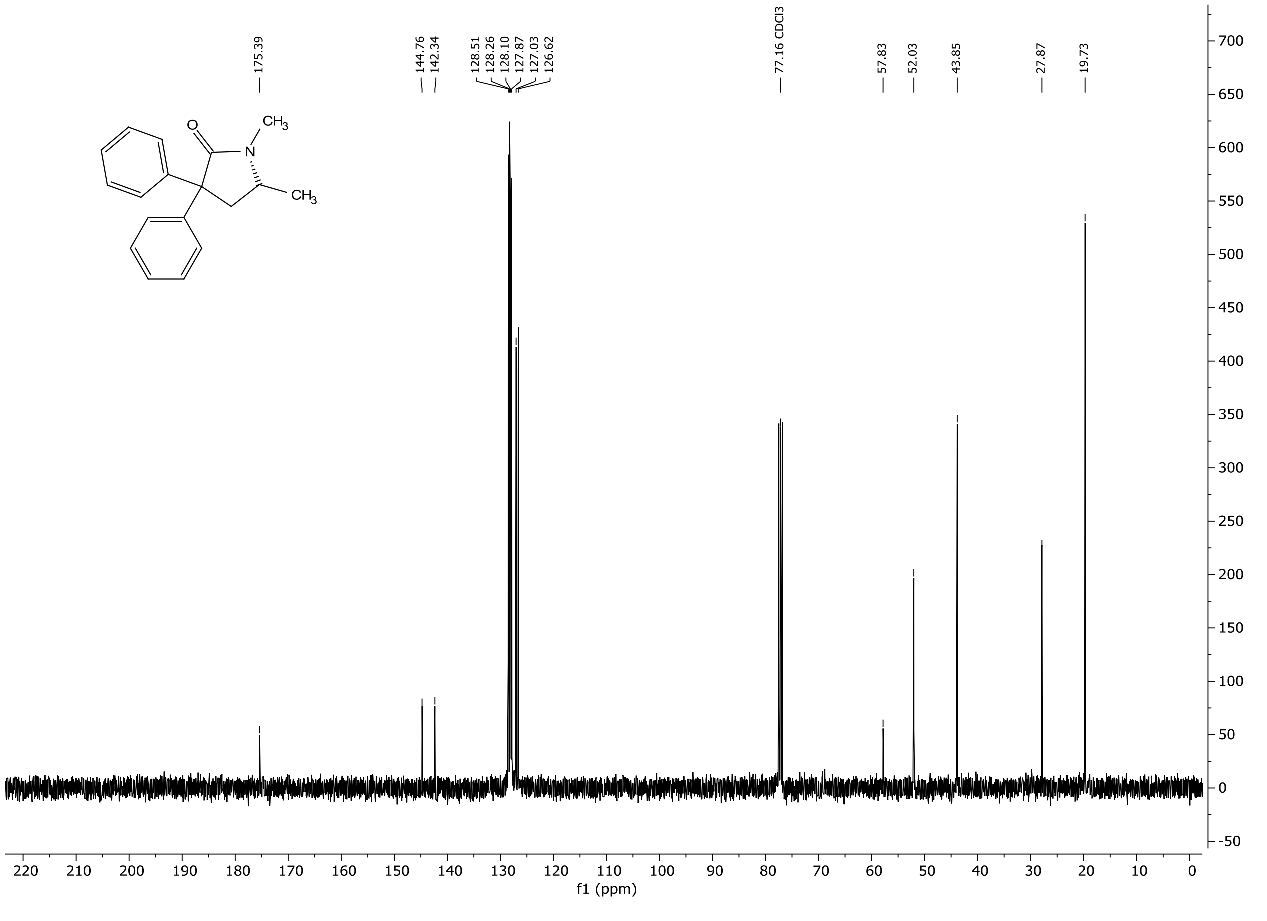

UPLC chromatogram of (*R*)-1,5-dimethyl-3,3-diphenylpyrrolidin-2-one ((*R*)-**DDPO**).

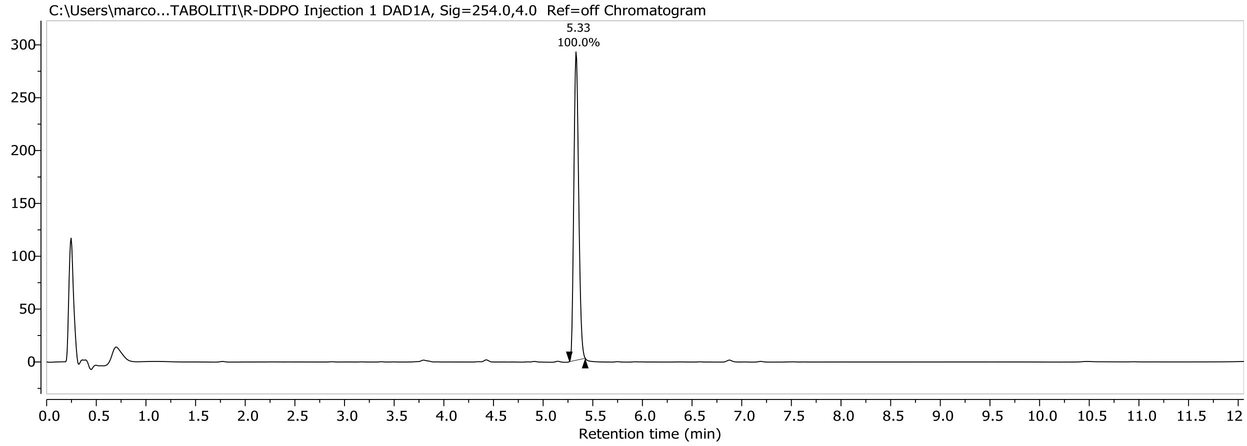

Chiral HPLC chromatogram of (*R*)-1,5-dimethyl-3,3-diphenylpyrrolidin-2-one ((*R*)-**DDPO**).

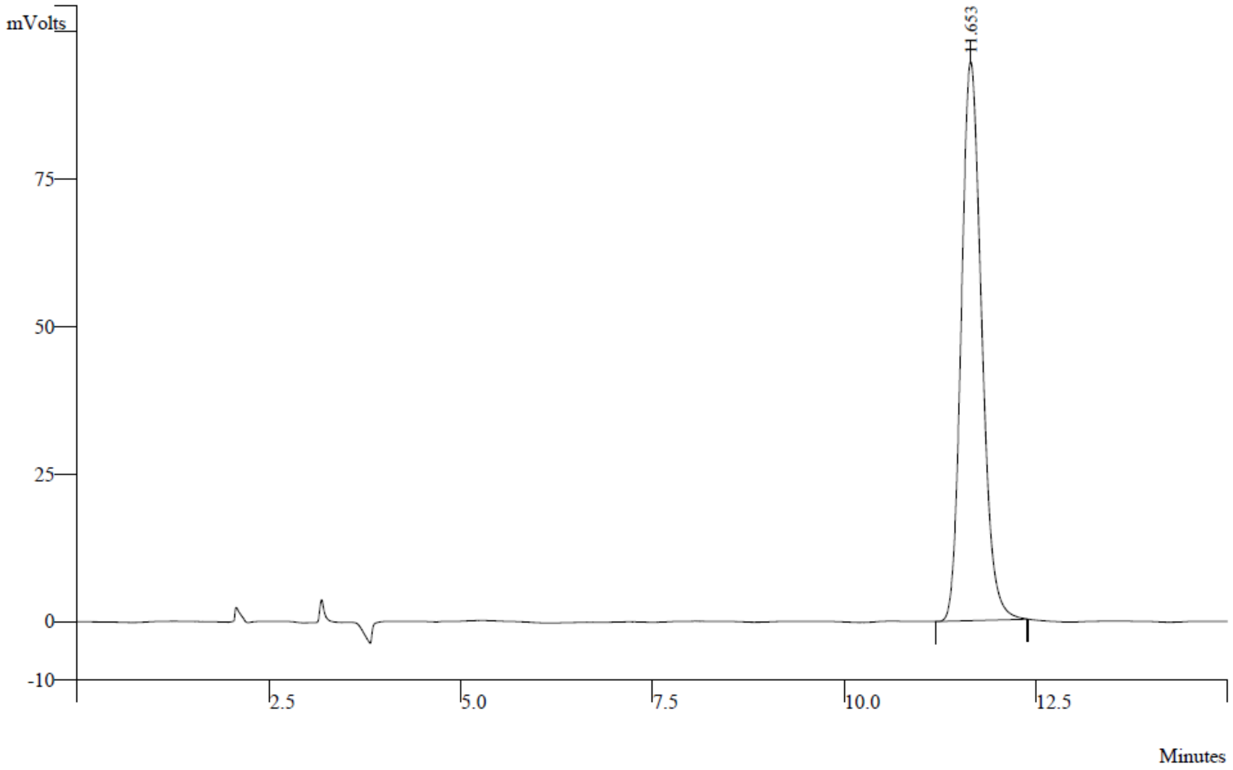

Peak No	Ret Time (min)	Peak Area (counts)	Result ()
1	11.653	1746918	100,00
		**1746918**	**100,00**

^{1}H and ^{13}C NMR spectra of (*S*)-1,5-dimethyl-3,3-diphenylpyrrolidin-2-one ((*S*)-**DDPO**).

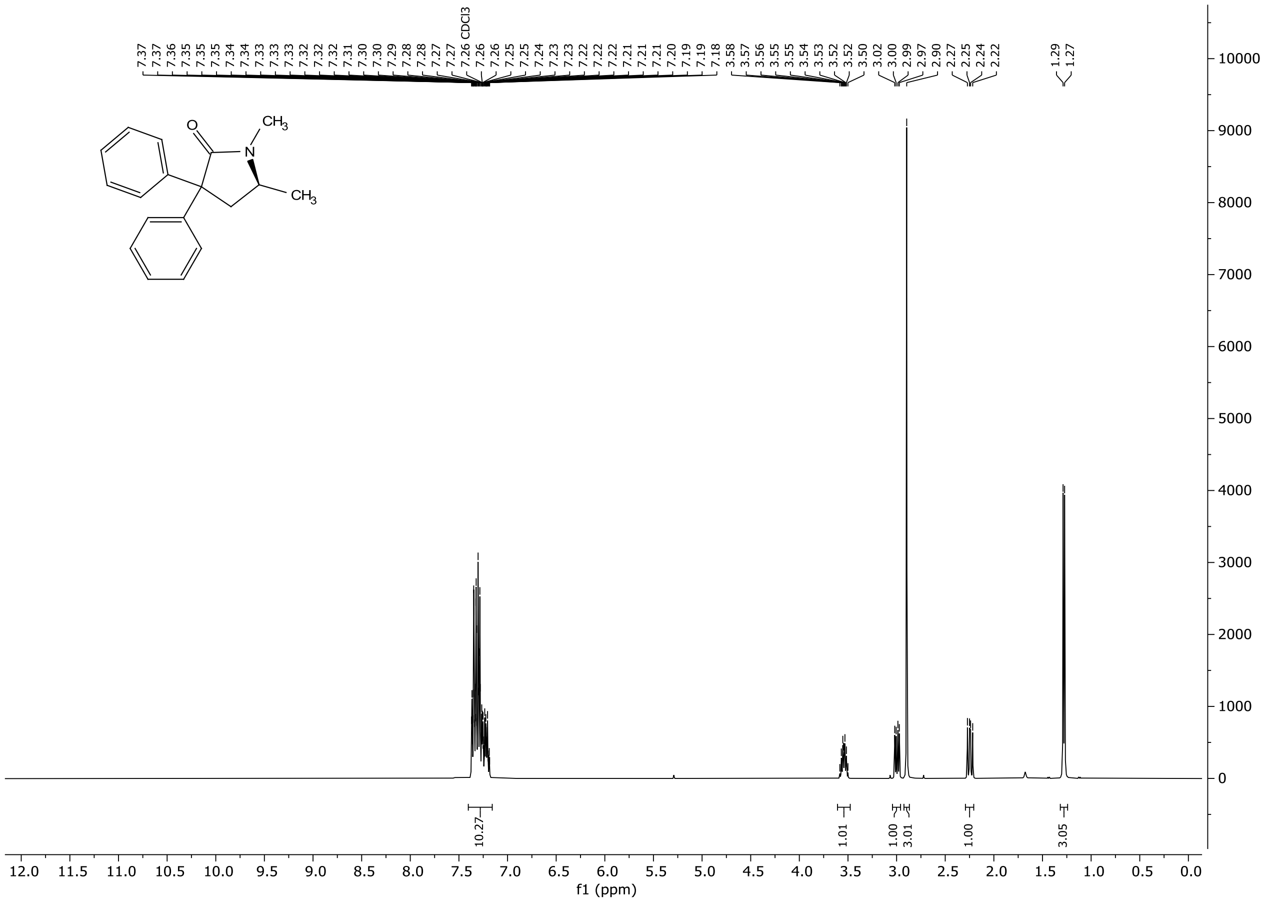

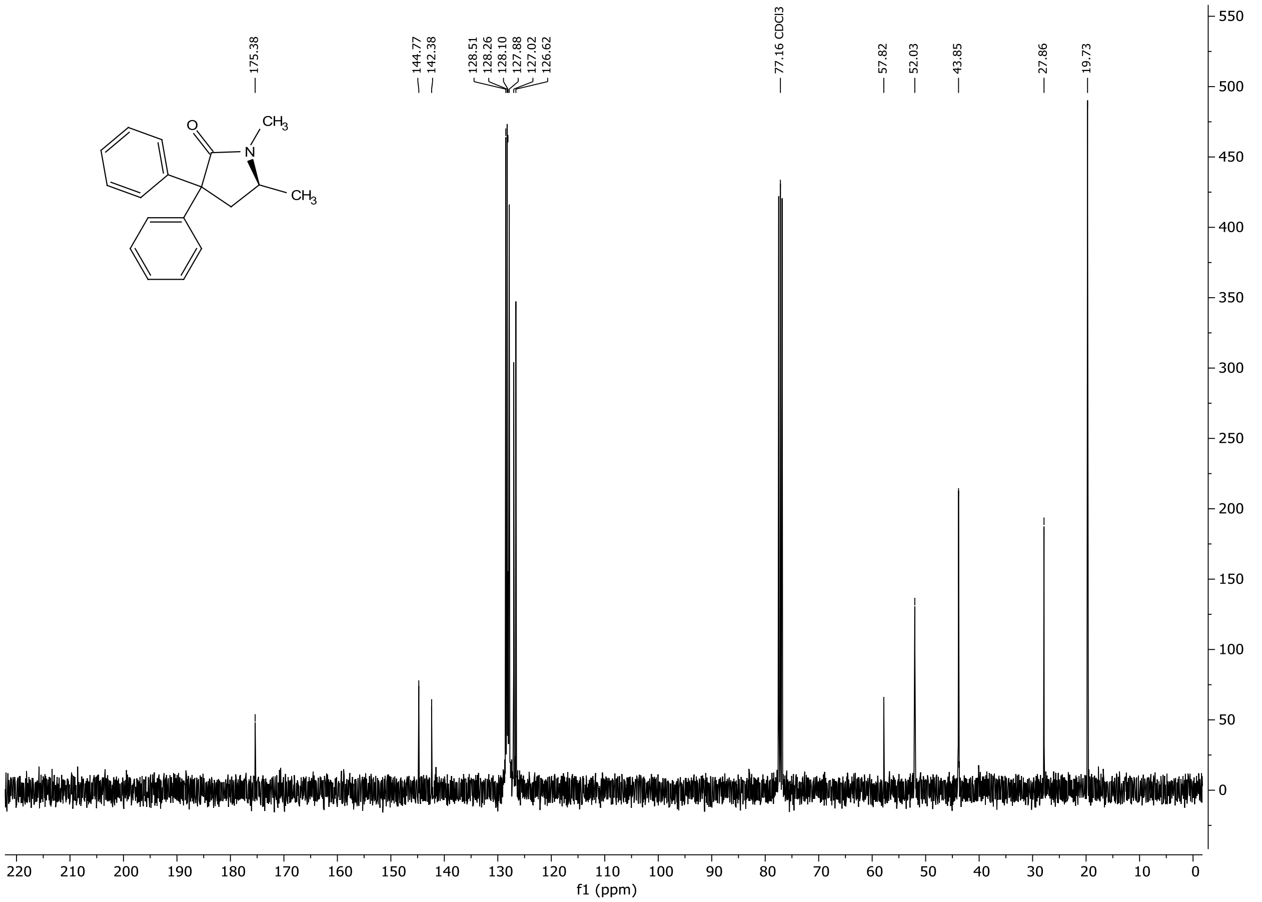

UPLC chromatogram of (*S*)-1,5-dimethyl-3,3-diphenylpyrrolidin-2-one ((*S*)-**DDPO**).

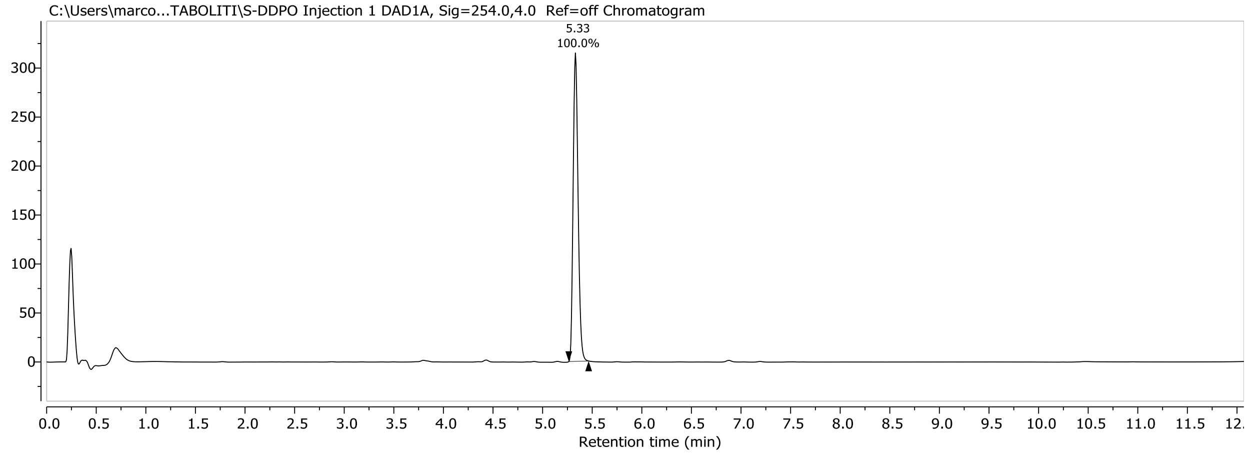

Chiral HPLC chromatogram of (*S*)-1,5-dimethyl-3,3-diphenylpyrrolidin-2-one ((*S*)-**DDPO**).

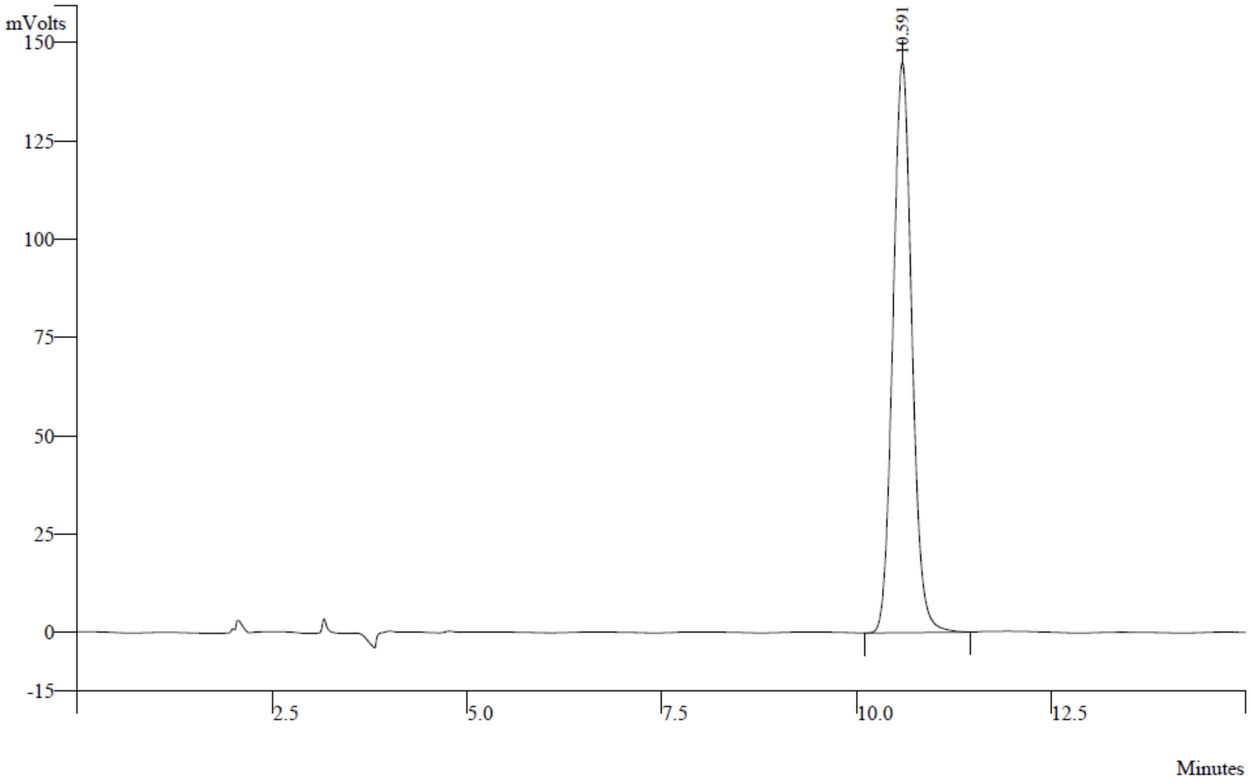

Peak No	Ret Time (min)	Peak Area (counts)	Result ()
1	10,591	2461410	100,00
		**2461410**	**100,00**

^{1}H and ^{13}C NMR spectra of (*R*)-N-(4-cyano-4,4-diphenylbutan-2-yl)benzamide ((*R*)-**6**)

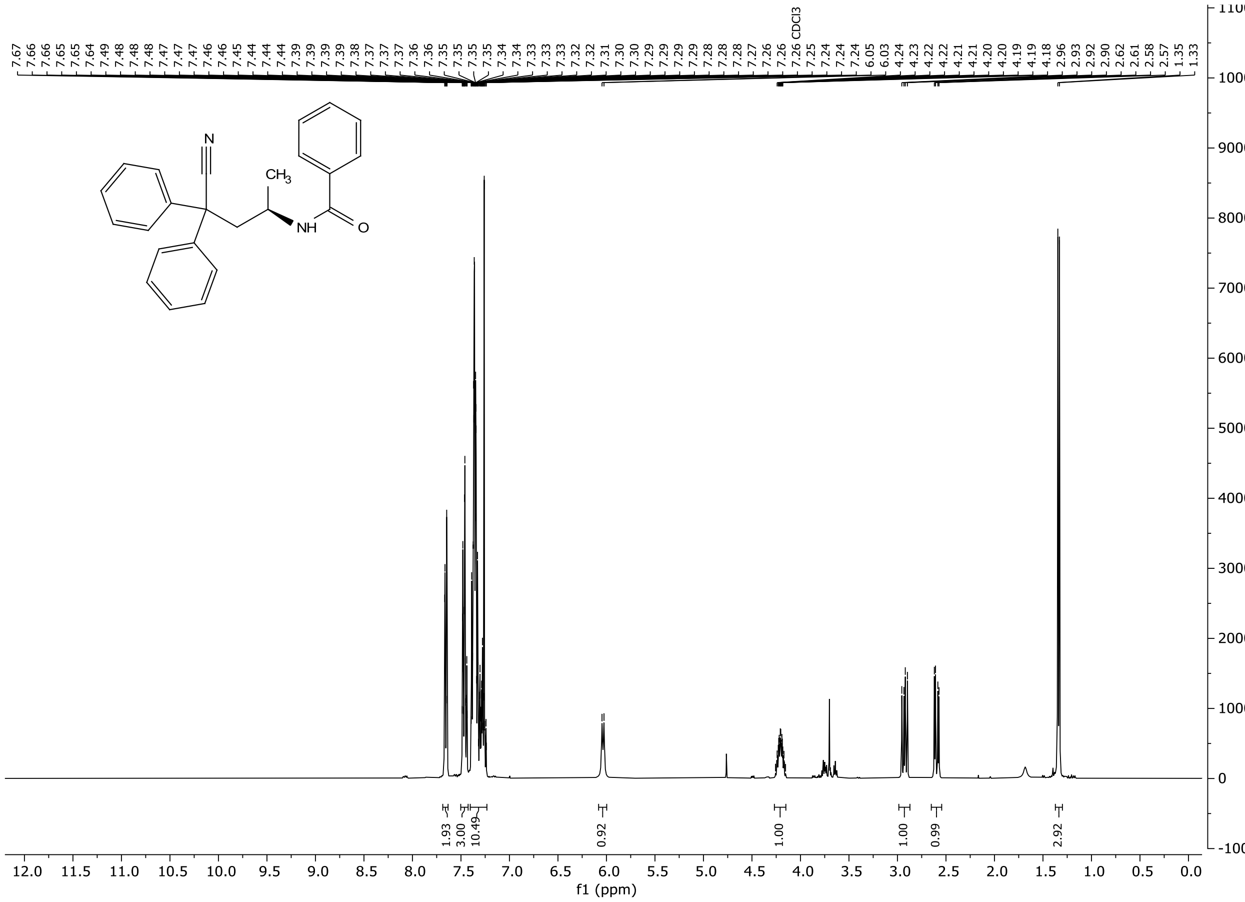

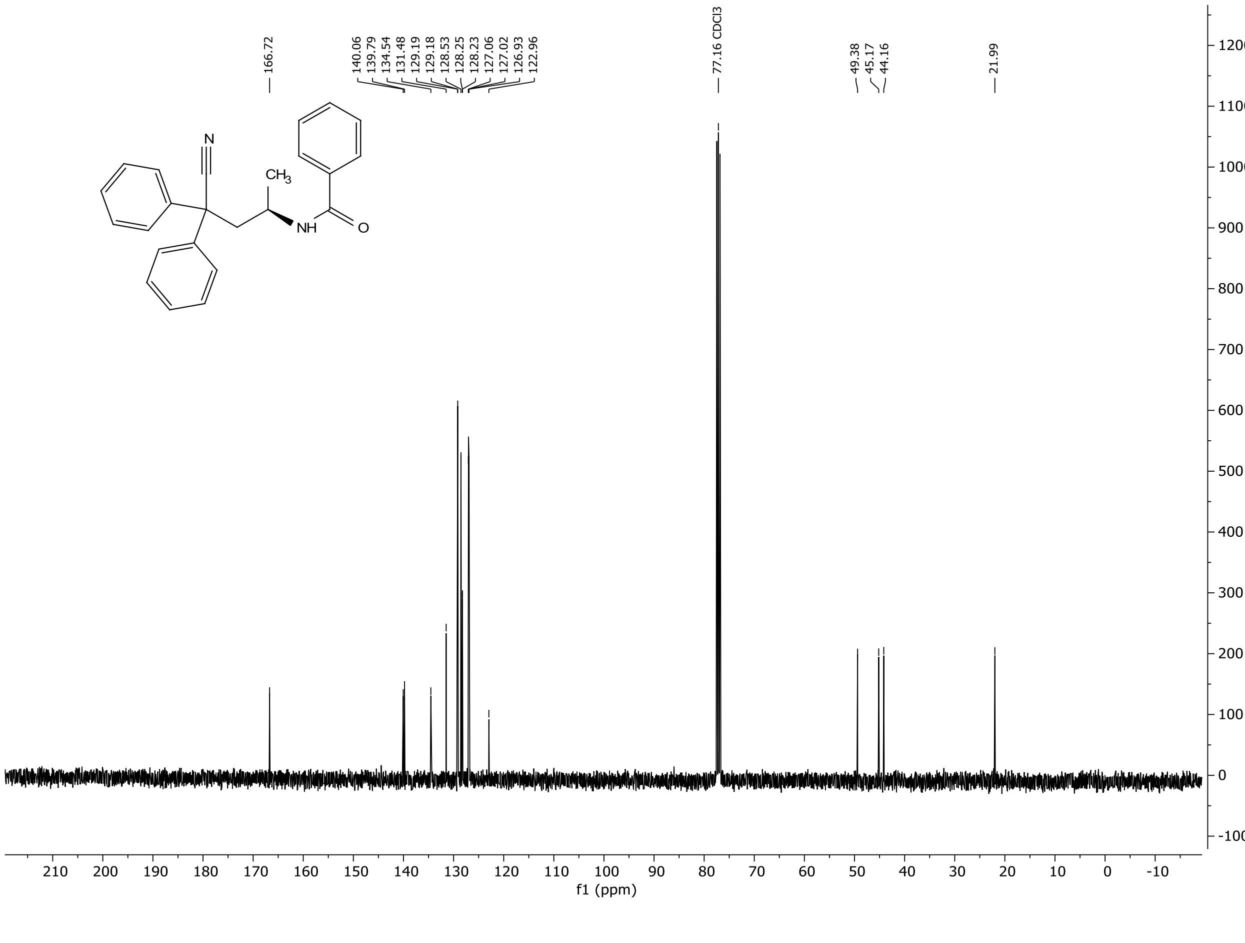

^{1}H and ^{13}C NMR spectra of (*S*)-N-(4-cyano-4,4-diphenylbutan-2-yl)benzamide ((*S*)-**6**)

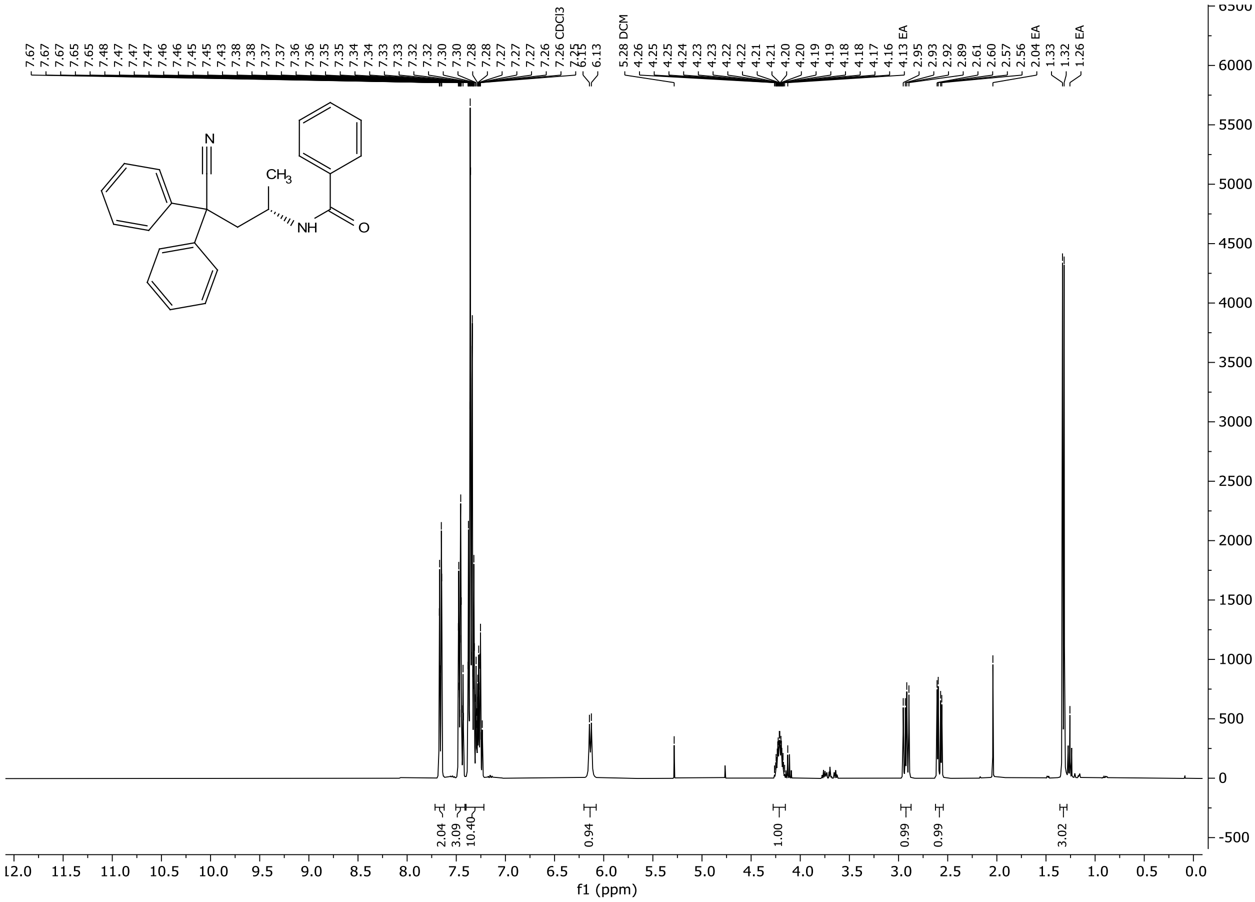

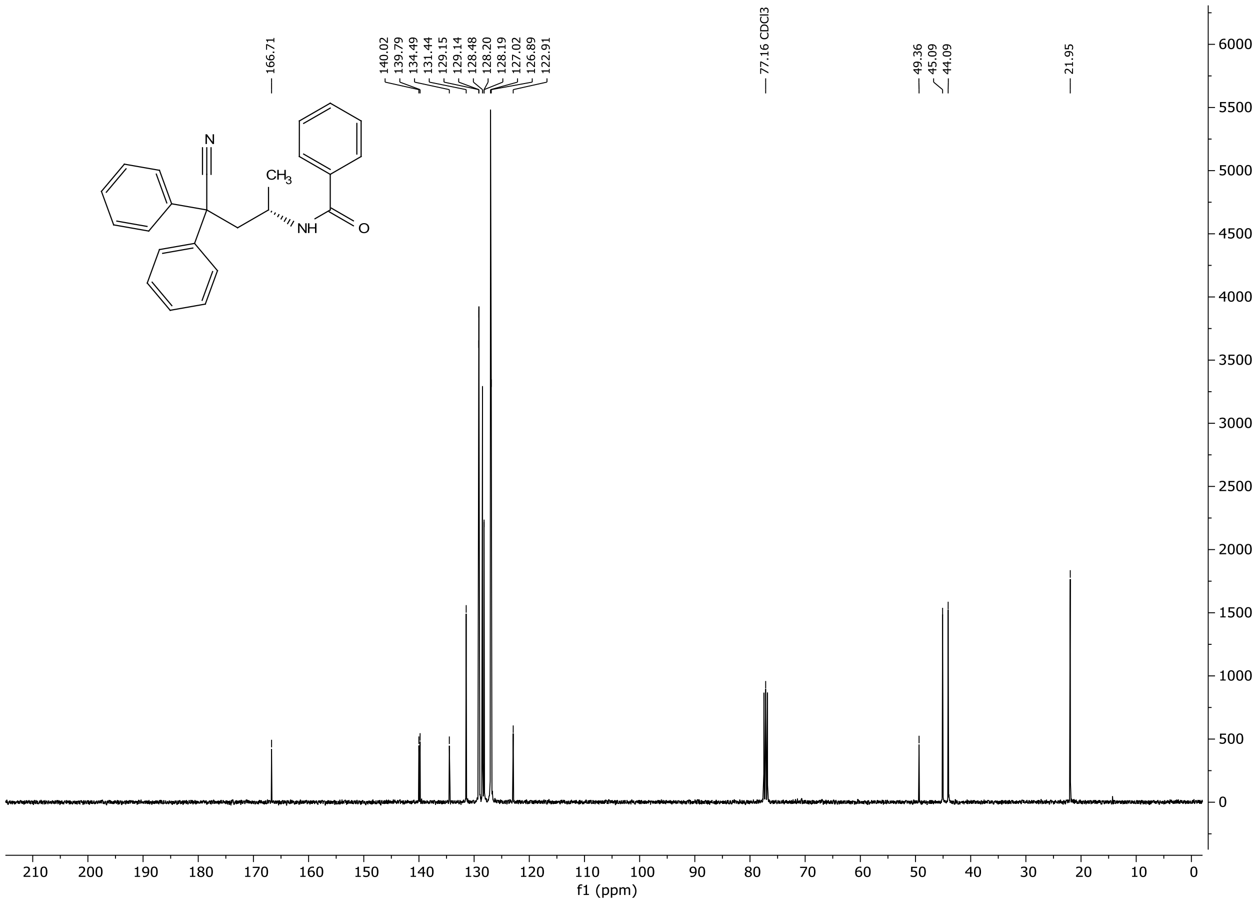

^{1}H and ^{13}C NMR spectra of (*R*)-N-(5-oxo-4,4-diphenylheptan-2-yl)benzamide ((*R*)-**7**).

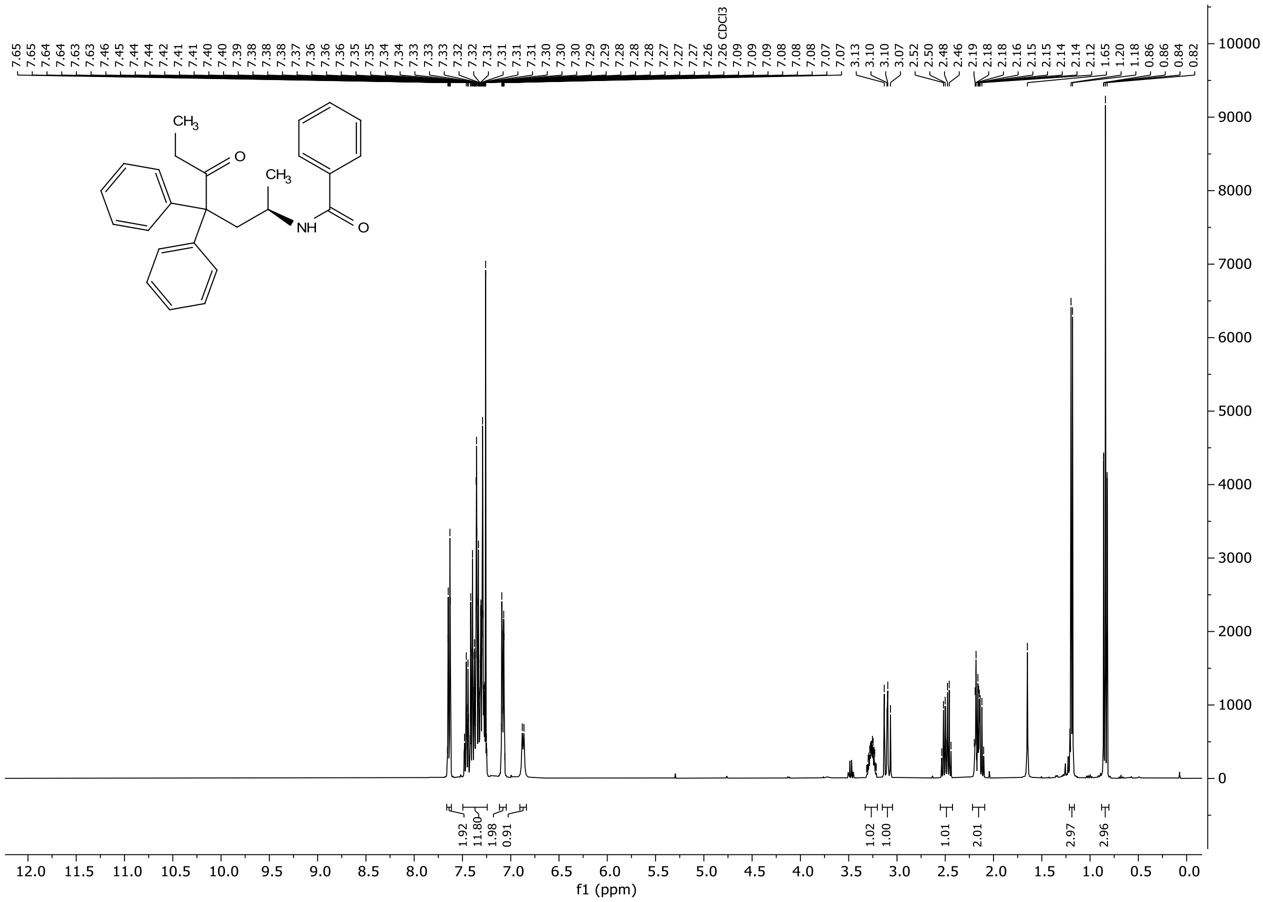

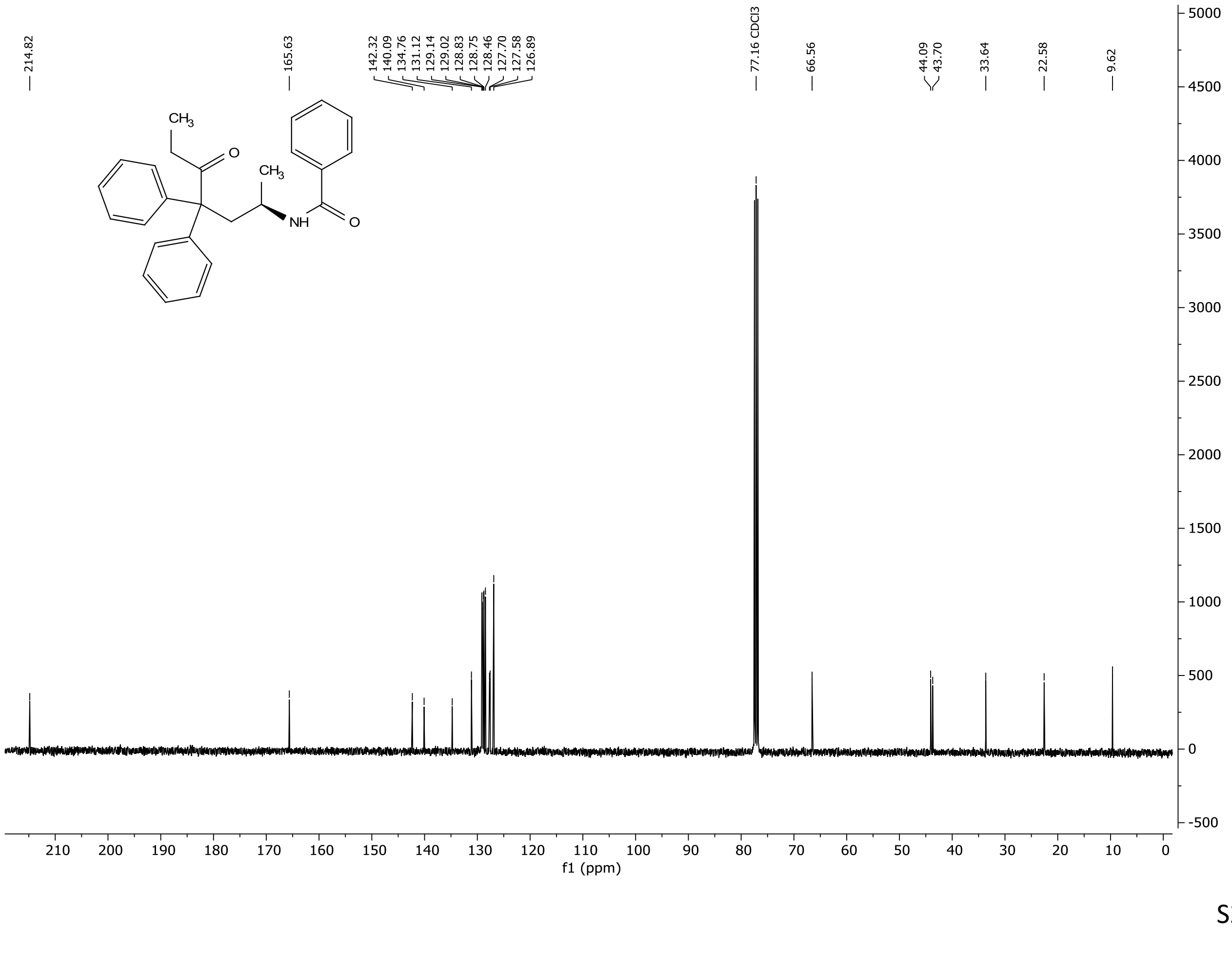

^{1}H and ^{13}C NMR spectra of (*S*)-N-(5-oxo-4,4-diphenylheptan-2-yl)benzamide ((*S*)-**7**).

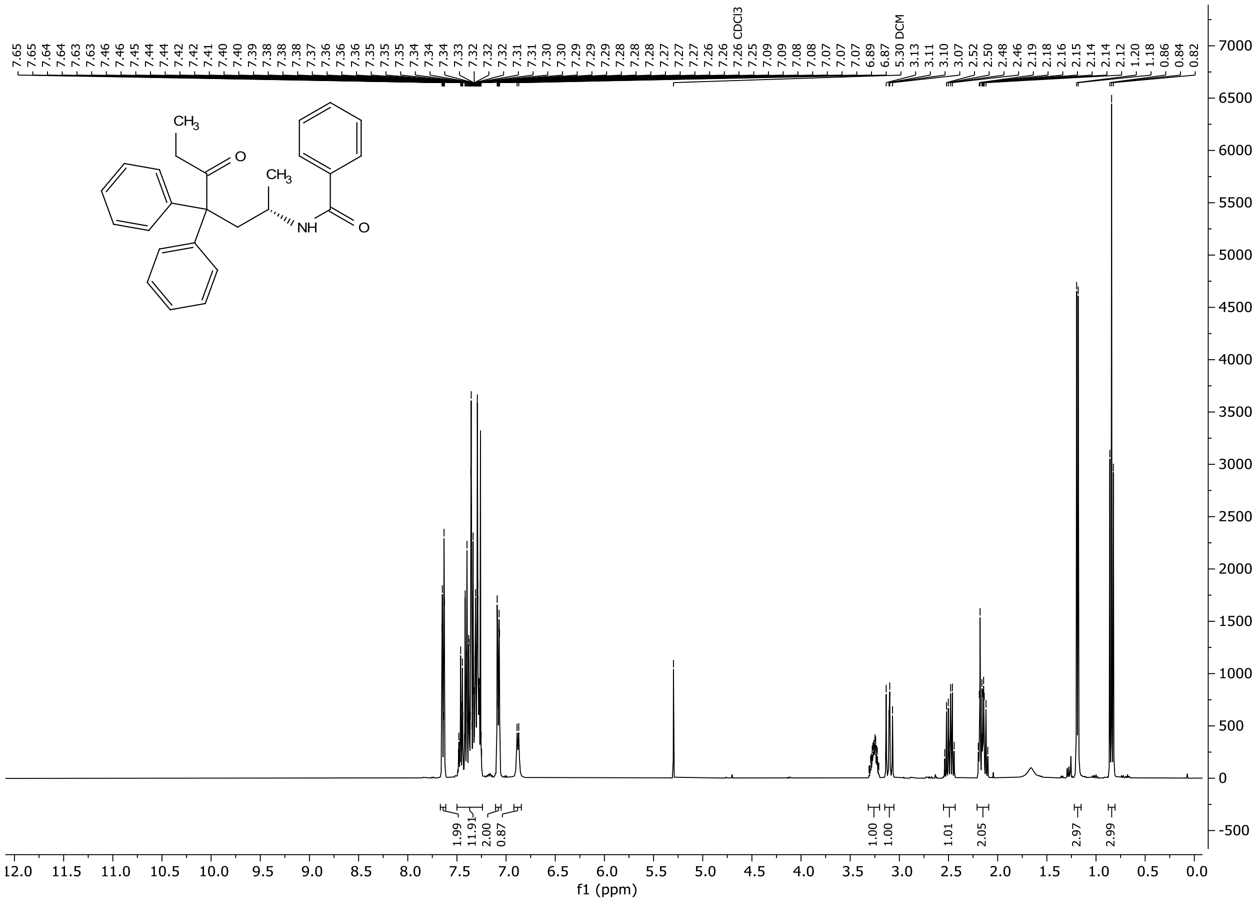

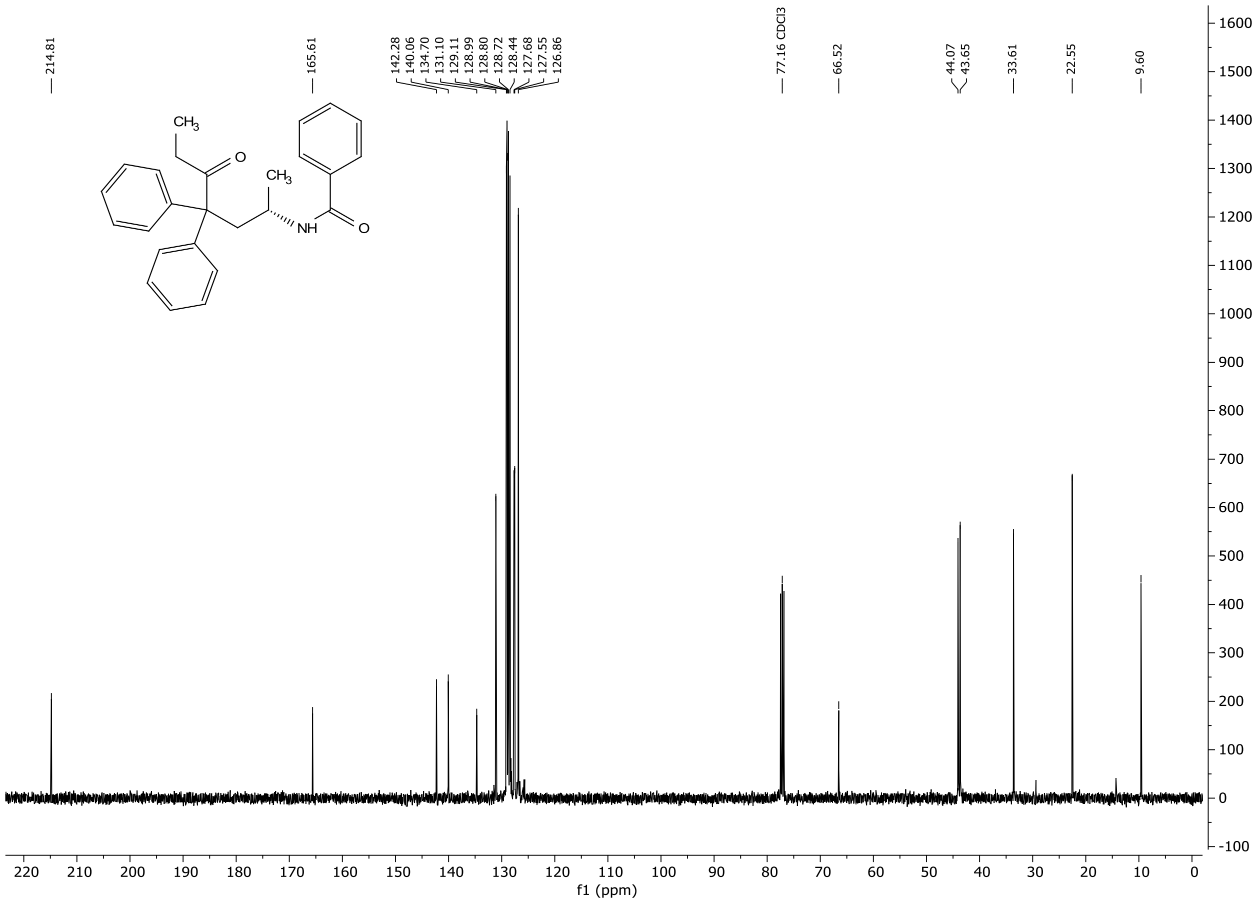

^{1}H and ^{13}C NMR spectra of (*R*)-5-ethyl-2-methyl-4,4-diphenyl-3,4-dihydro-2H-pyrrole ((*R*)-**EMDP**).

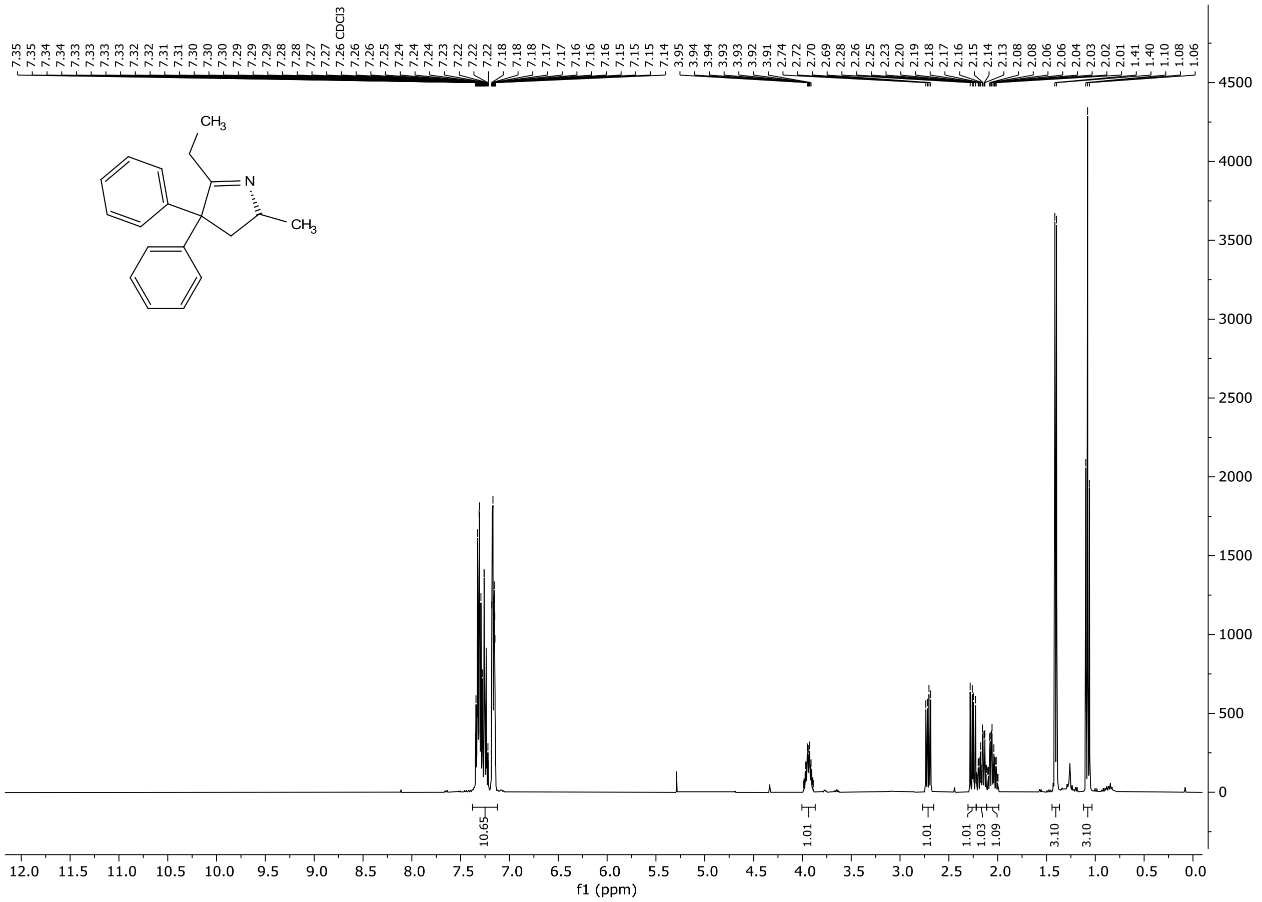

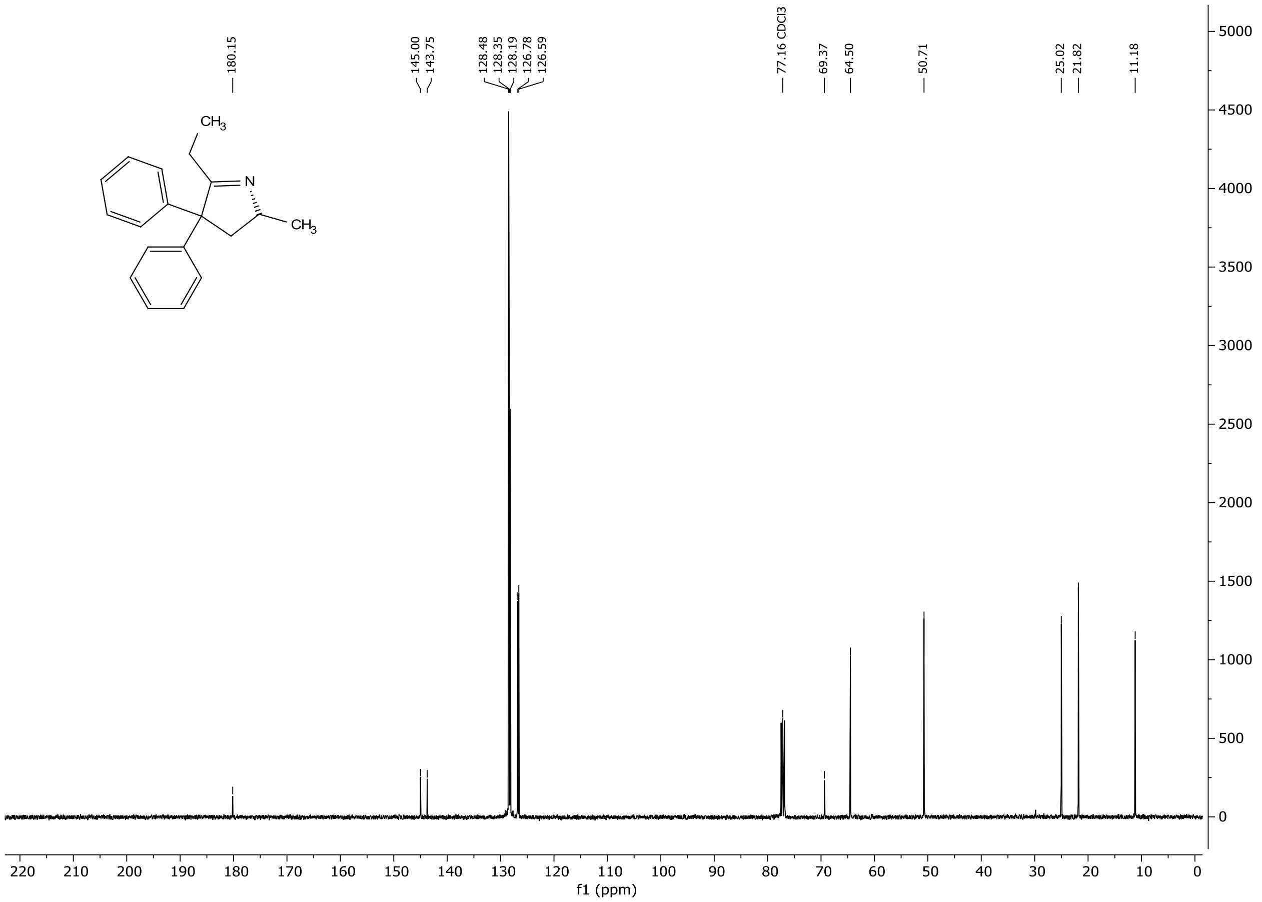

UPLC chromatogram of (*R*)-5-ethyl-2-methyl-4,4-diphenyl-3,4-dihydro-2H-pyrrole ((*R*)-**EMDP**).

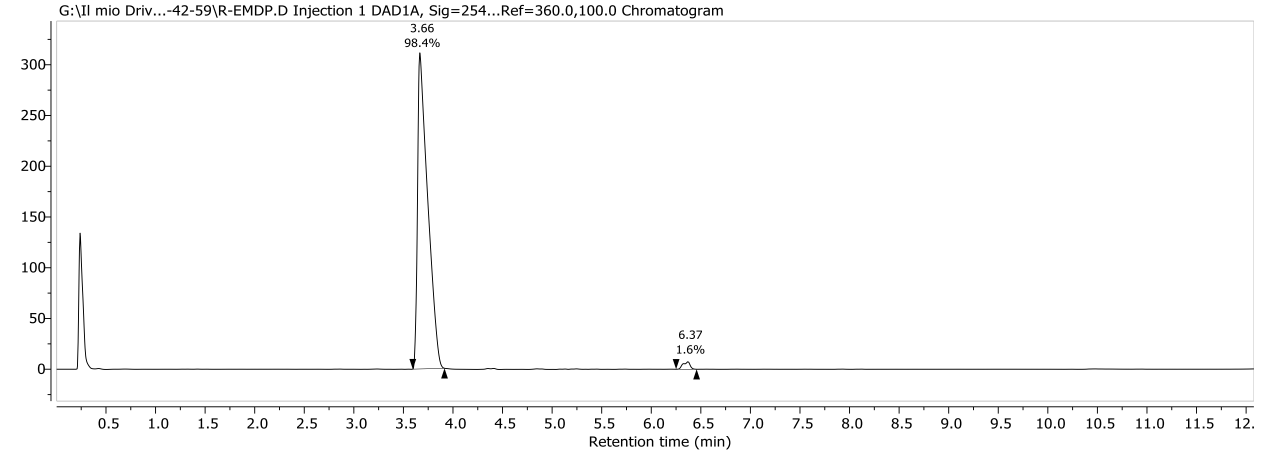

Chiral HPLC chromatogram of (*R*)-5-ethyl-2-methyl-4,4-diphenyl-3,4-dihydro-2H-pyrrole ((*R*)-**EMDP**).

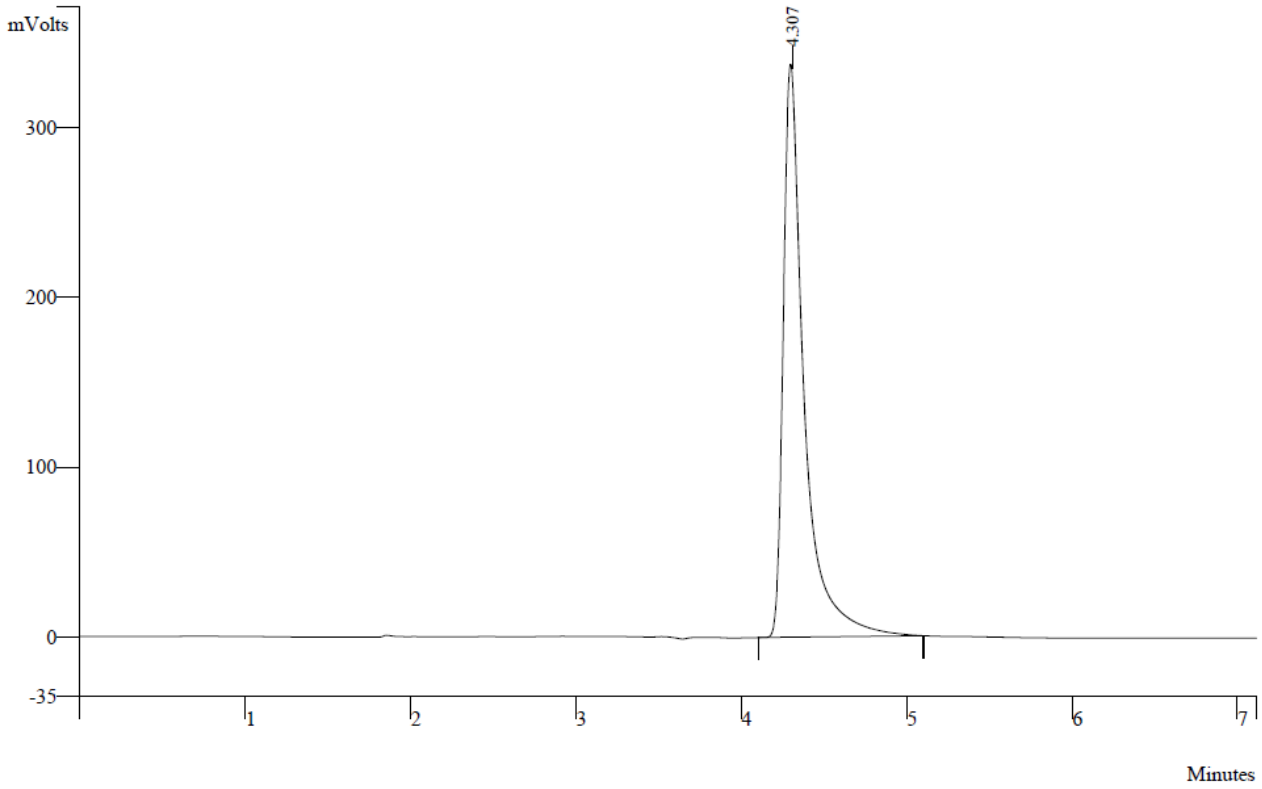

Peak No	Ret Time (min)	Peak Area (counts)	Result ()
1	4,307	2957405	100,00
		**2957405**	**100,00**

^1H and ^{13}C NMR spectra of (*S*)-5-ethyl-2-methyl-4,4-diphenyl-3,4-dihydro-2H-pyrrole ((*S*)-**EMDP**).

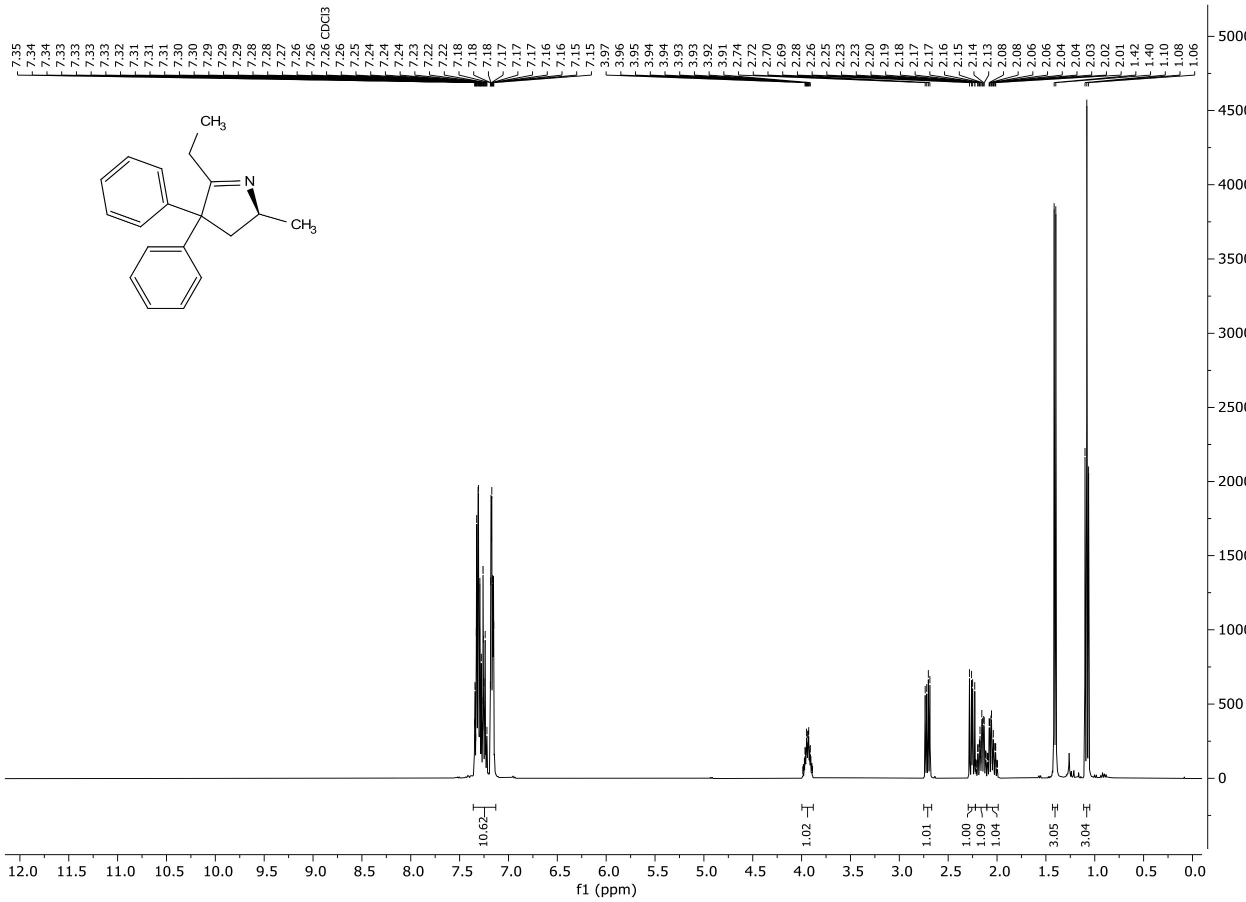

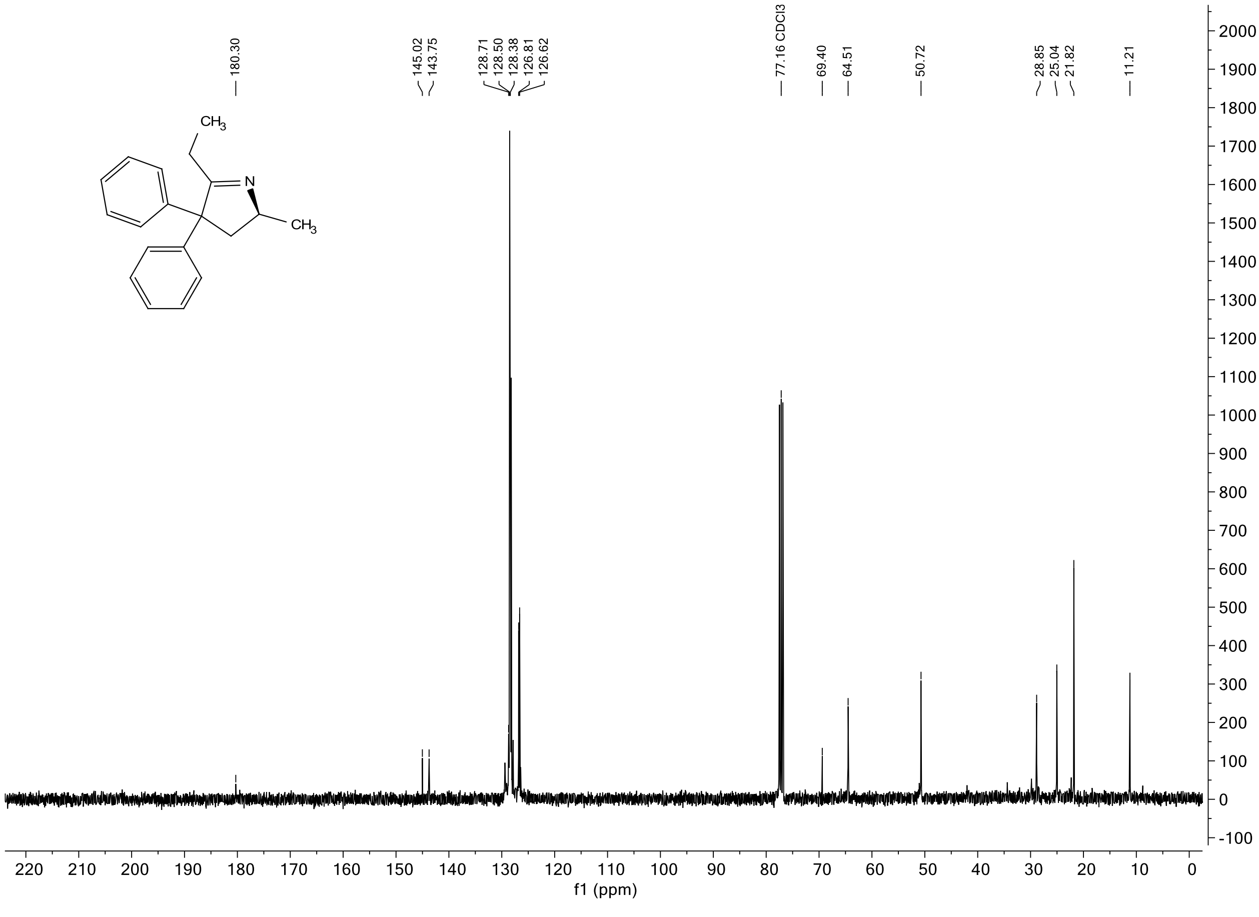

UPLC chromatogram of (*S*)-5-ethyl-2-methyl-4,4-diphenyl-3,4-dihydro-2H-pyrrole ((*S*)-**EMDP**).

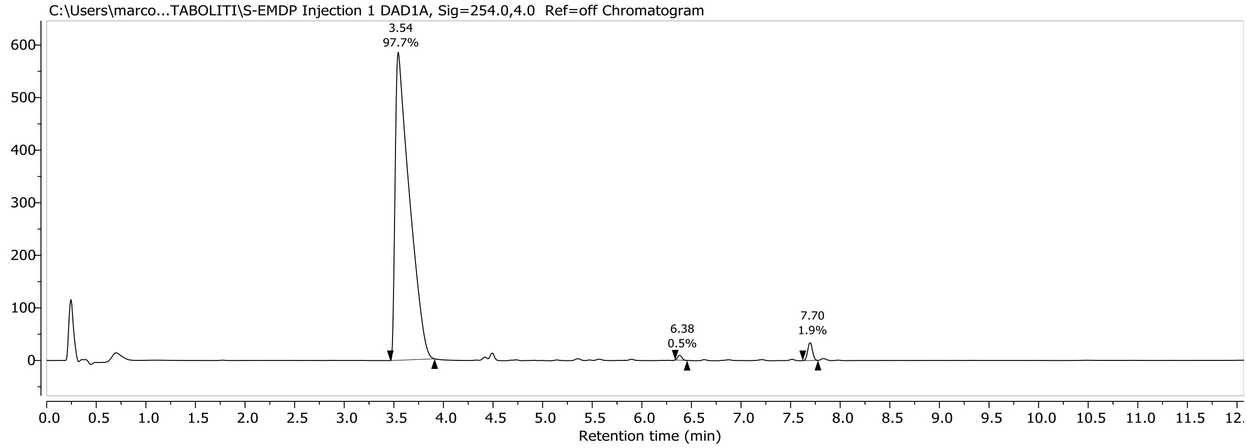

Chiral HPLC chromatogram of (*S*)-5-ethyl-2-methyl-4,4-diphenyl-3,4-dihydro-2H-pyrrole ((*S*)-**EMDP**).

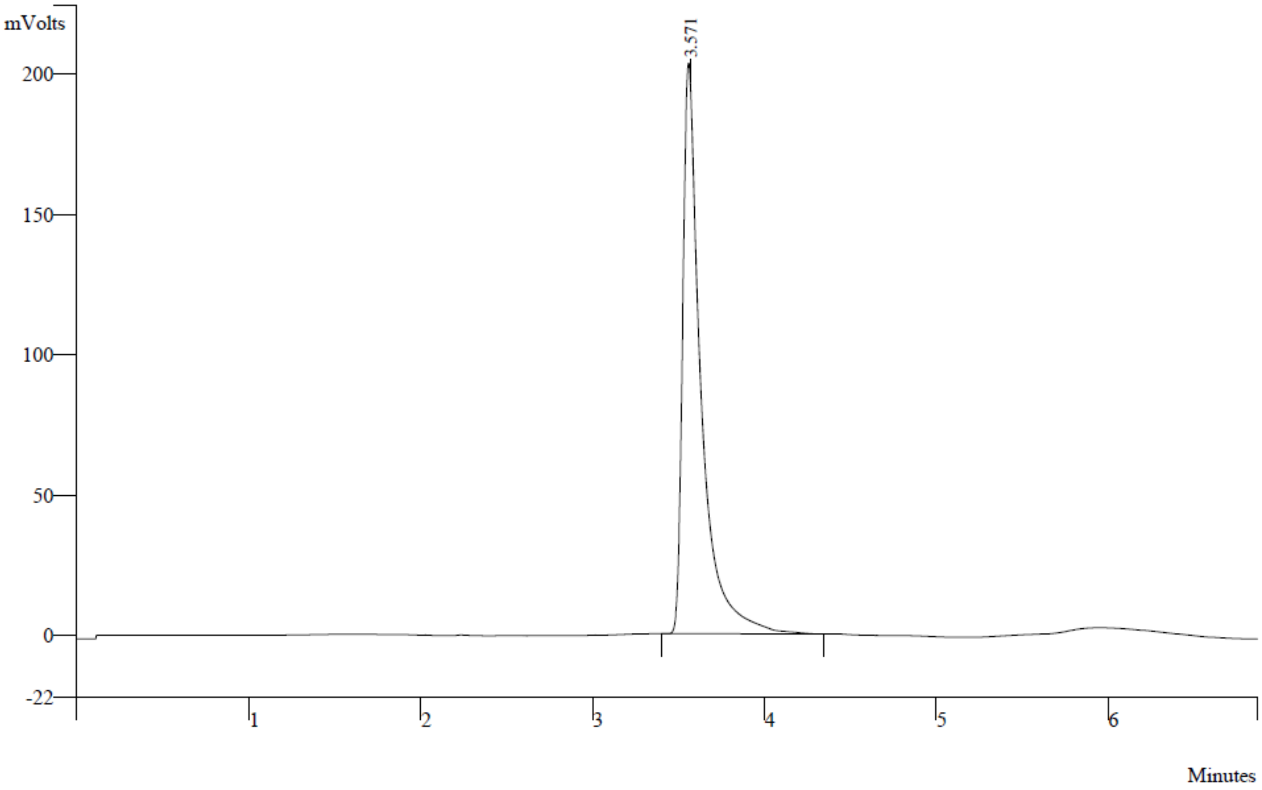

Peak No	Ret Time (min)	Peak Area (counts)	Result ()
1	3,571	1512610	100,00
		**1512610**	**100,00**

^1H and ^{13}C NMR spectra of (*R*)-2-ethyl-1,5-dimethyl-3,3-diphenyl-3,4-dihydro-5H-pyrrolium chloride ((*R*)-**EDDP**)

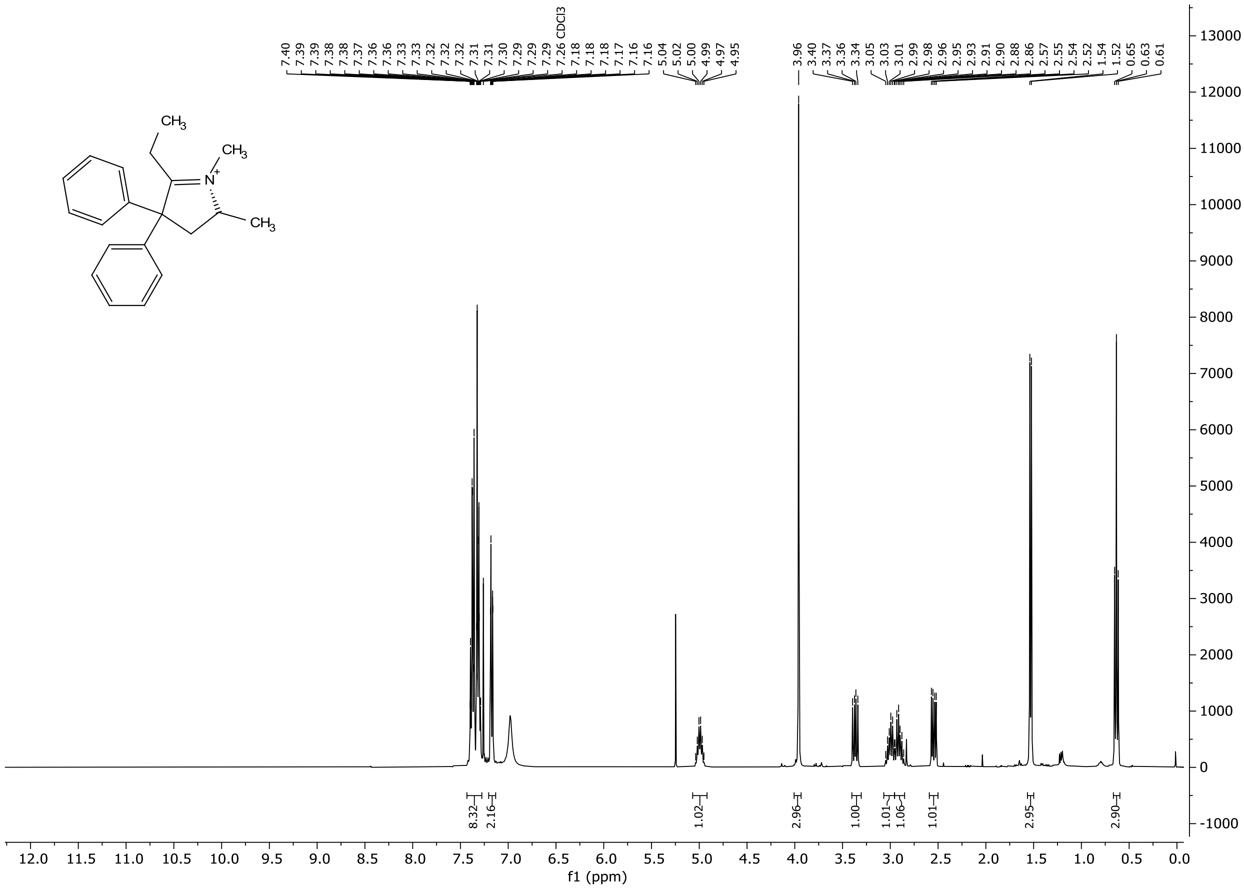

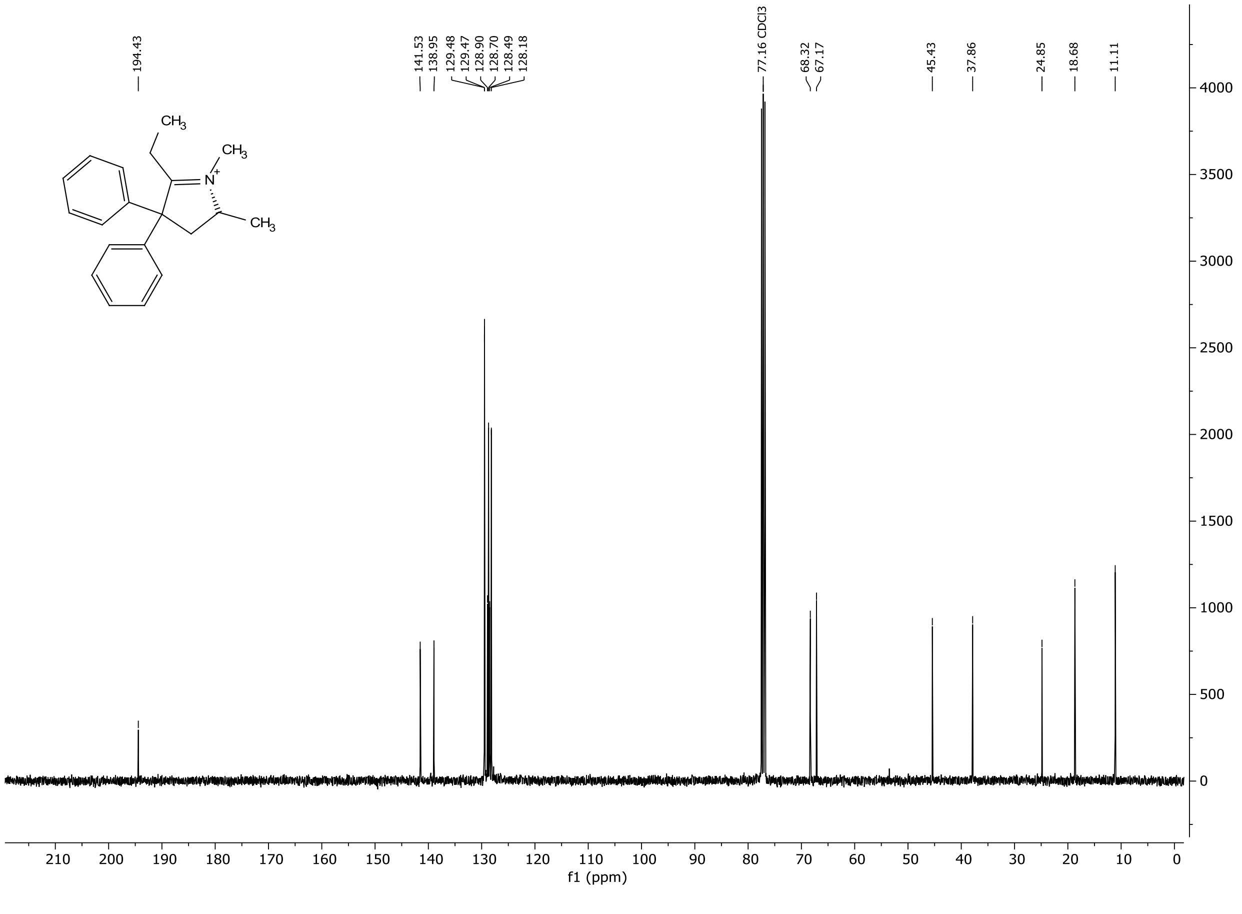

UPLC chromatogram of (*S*)-2-ethyl-1,5-dimethyl-3,3-diphenyl-3,4-dihydro-5H-pyrrolium chloride ((*R*)-**EDDP**)

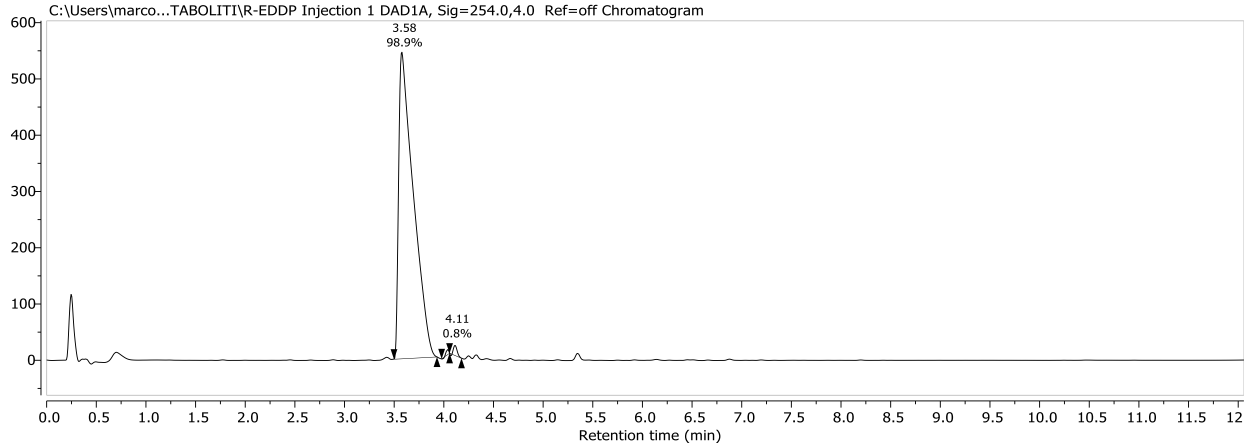

^{1}H and ^{13}C NMR spectra of (*S*)-2-ethyl-1,5-dimethyl-3,3-diphenyl-3,4-dihydro-5H-pyrrolium chloride ((*S*)-**EDDP**)

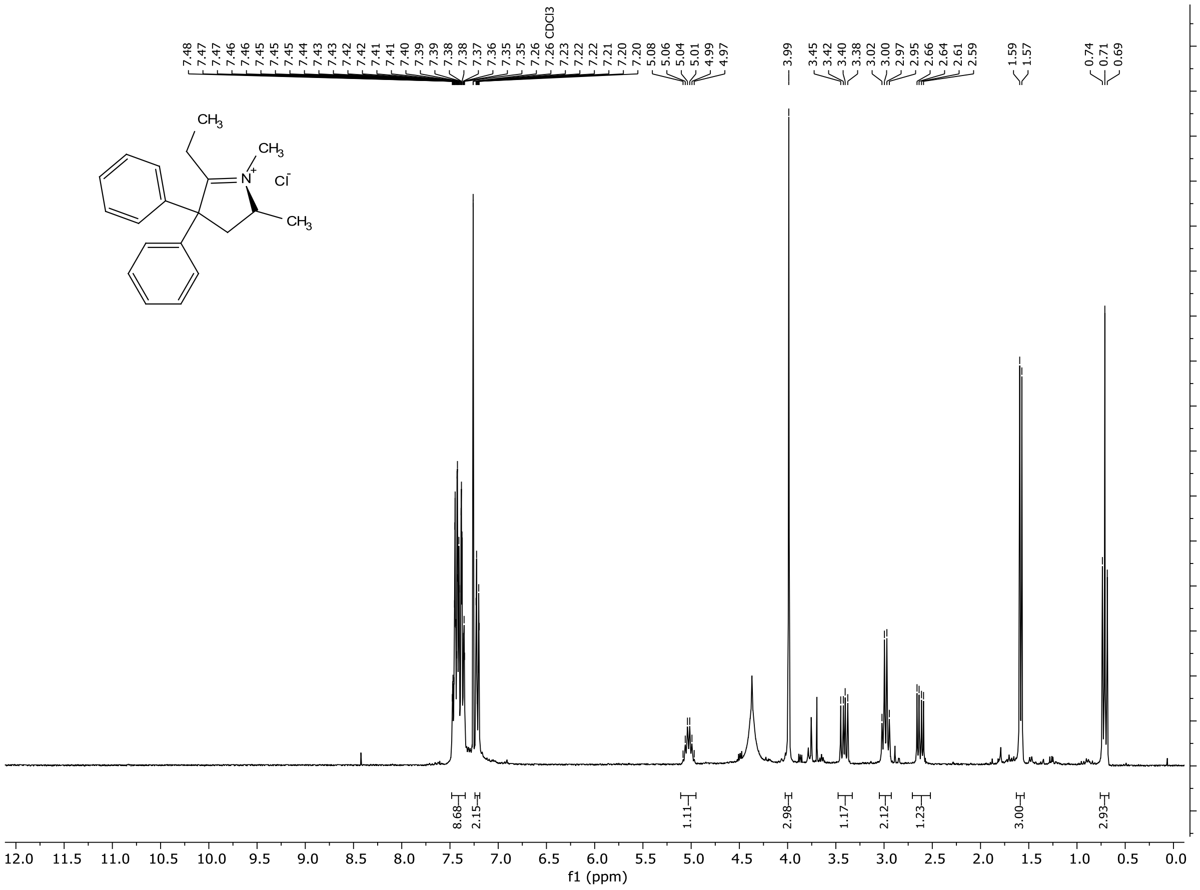

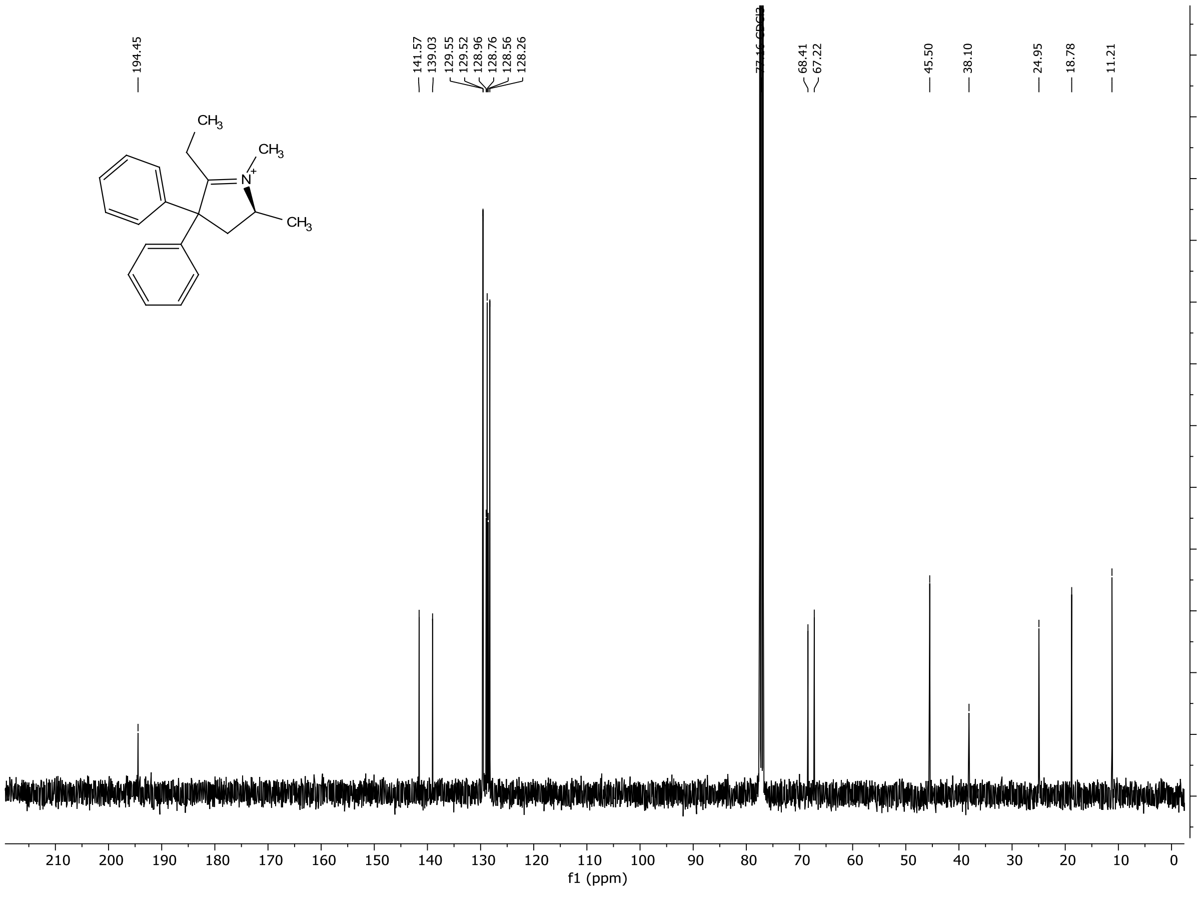

UPLC chromatogram of (*S*)-2-ethyl-1,5-dimethyl-3,3-diphenyl-3,4-dihydro-5H-pyrrolium chloride ((*S*)-**EDDP**)

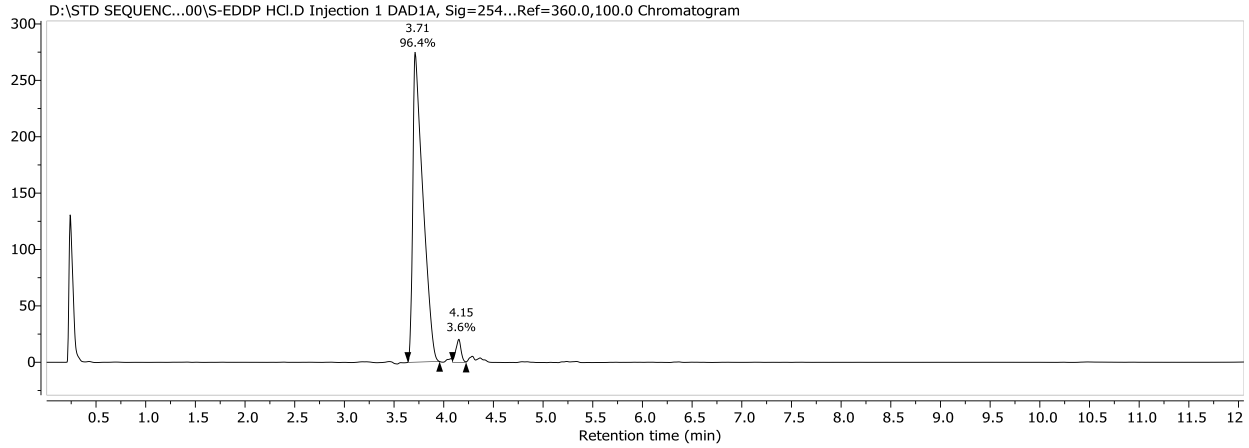

^{1}H and ^{13}C NMR spectra of (3*S*,6*R*)-6-(benzamido)-4,4-diphenylheptan-3-ol ((3*S*,6*R*)-**8**)

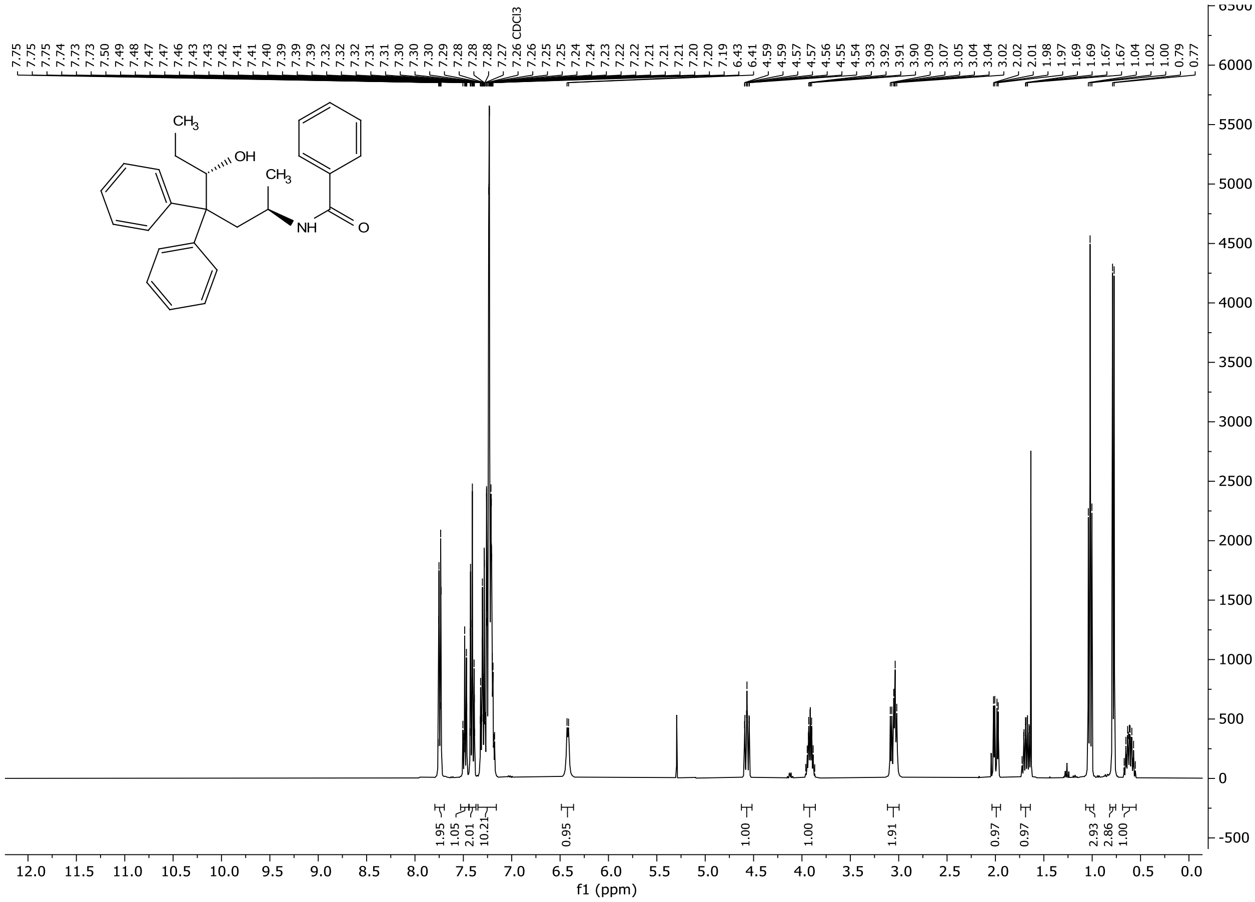

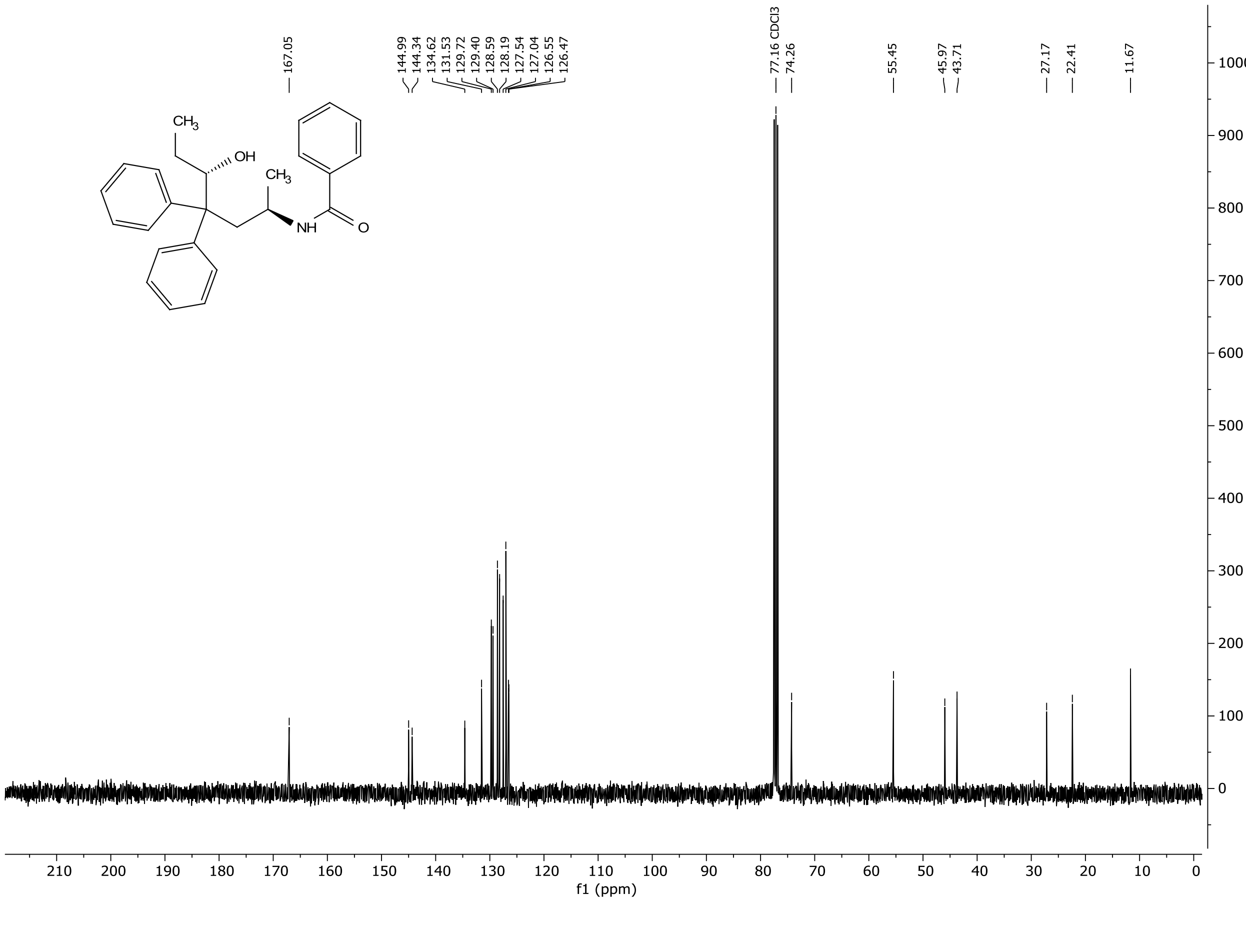

Chiral HPLC chromatogram of (3*S*,6*R*)-6-(benzamido)-4,4-diphenylheptan-3-ol ((3*S*,6*R*)-**8**)

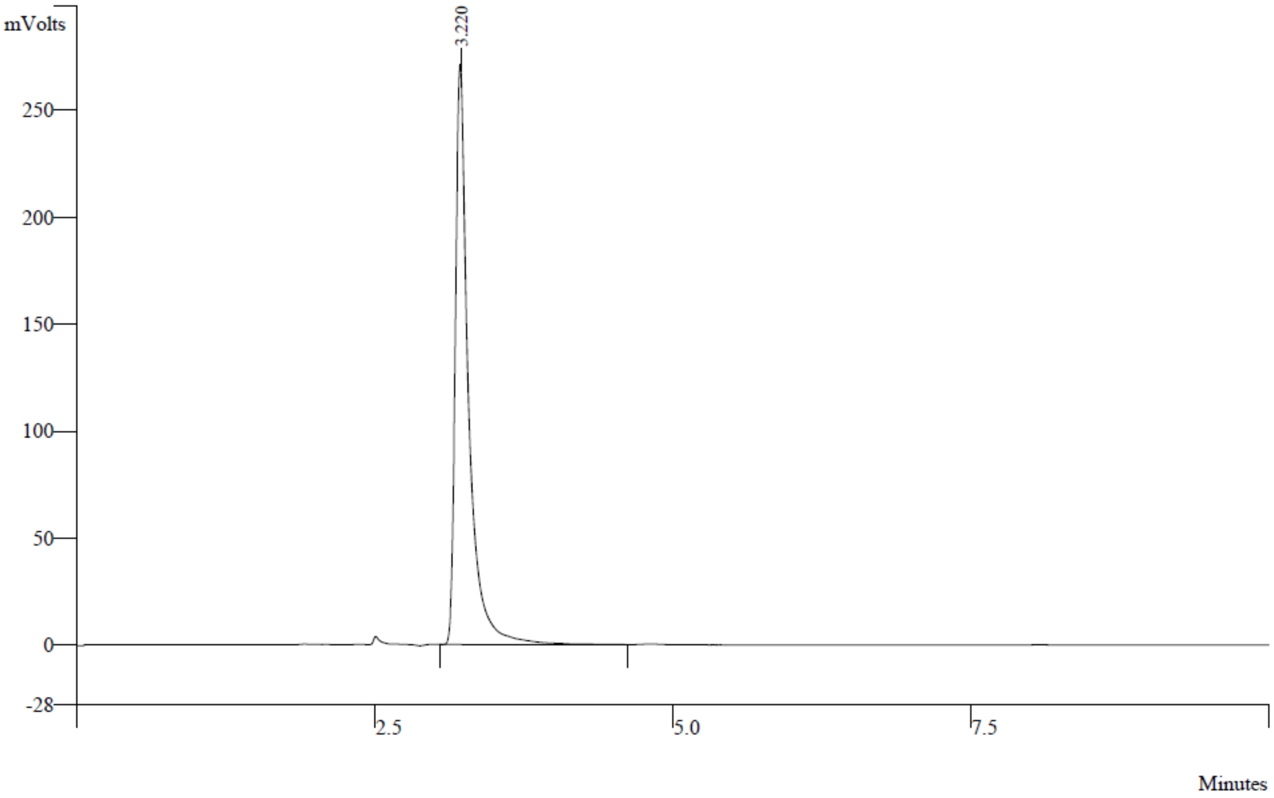

Peak No	Ret Time (min)	Peak Area (counts)	Result ()
1	3,220	2104082	100,00
		**2104082**	**100,00**

^{1}H and ^{13}C NMR spectra of (3*R*,6*S*)-6-(benzamido)-4,4-diphenylheptan-3-ol ((3*R*,6*S*)-**8**)

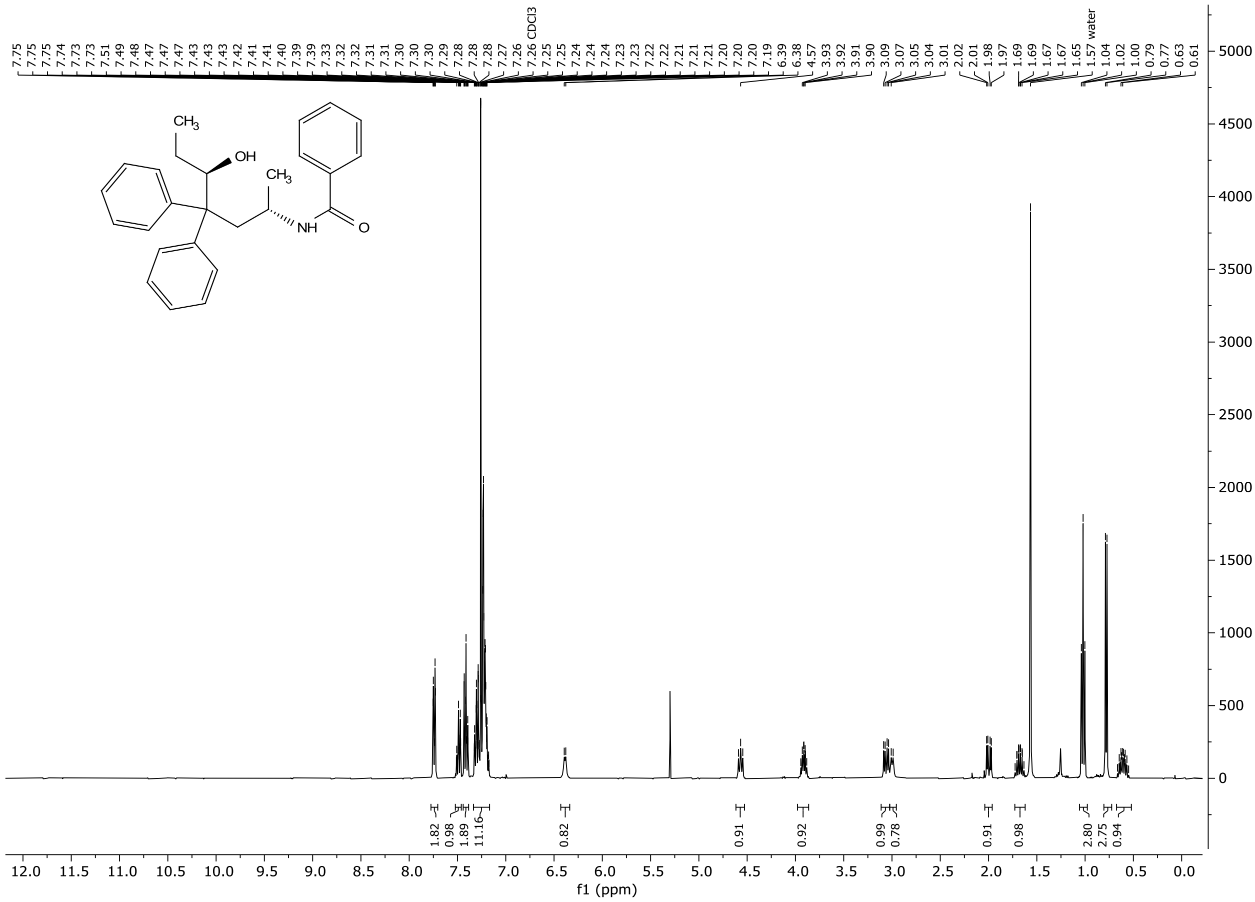

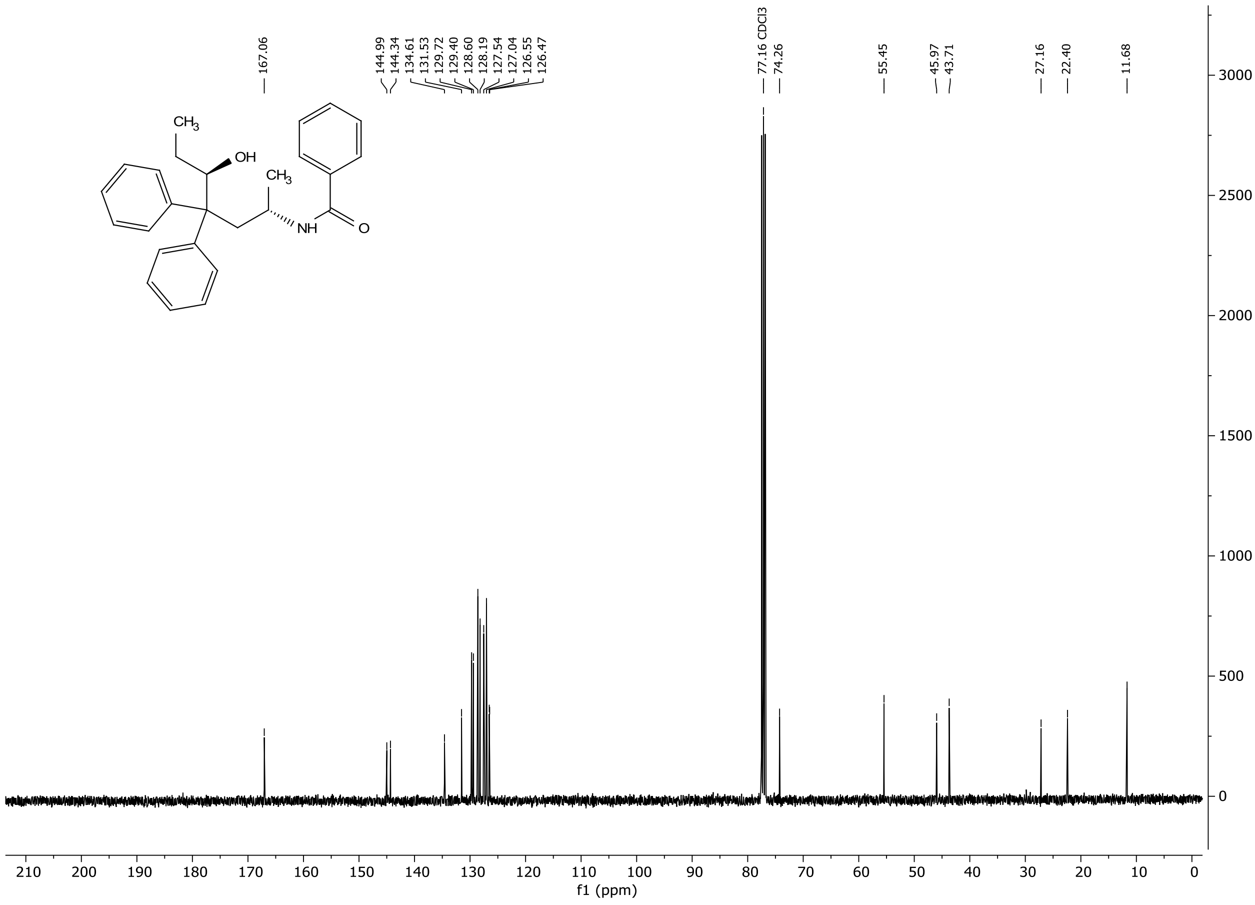

Chiral HPLC chromatogram of (3*R*,6*S*)-6-(benzamido)-4,4-diphenylheptan-3-ol ((3*R*,6*S*)-**8**)

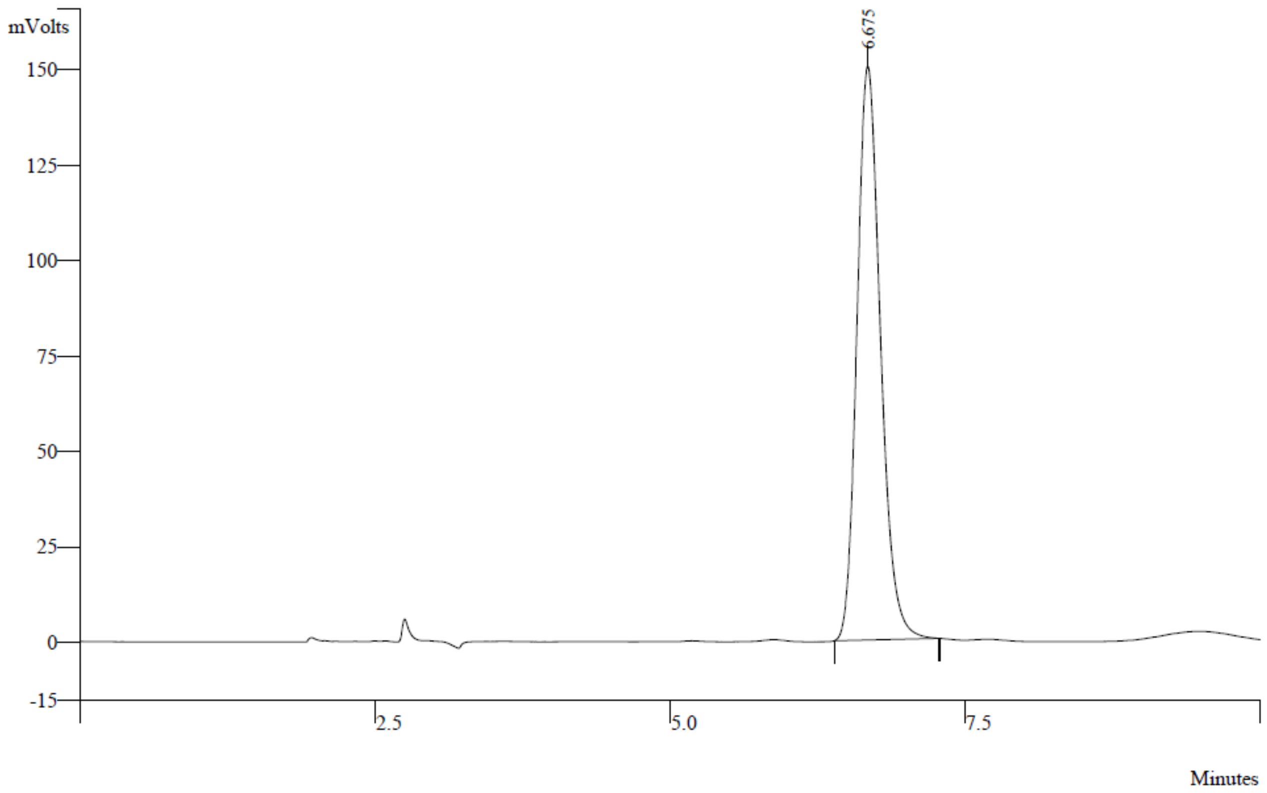

Peak No	Ret Time (min)	Peak Area (counts)	Result ()
1	6,675	2088919	100,00
		**2088919**	**100,00**

^{1}H and ^{13}C NMR spectra of (3*S*,6*S*)-6-(benzamido)-4,4-diphenylheptan-3-ol ((3*S*,6*S*)-**8**)

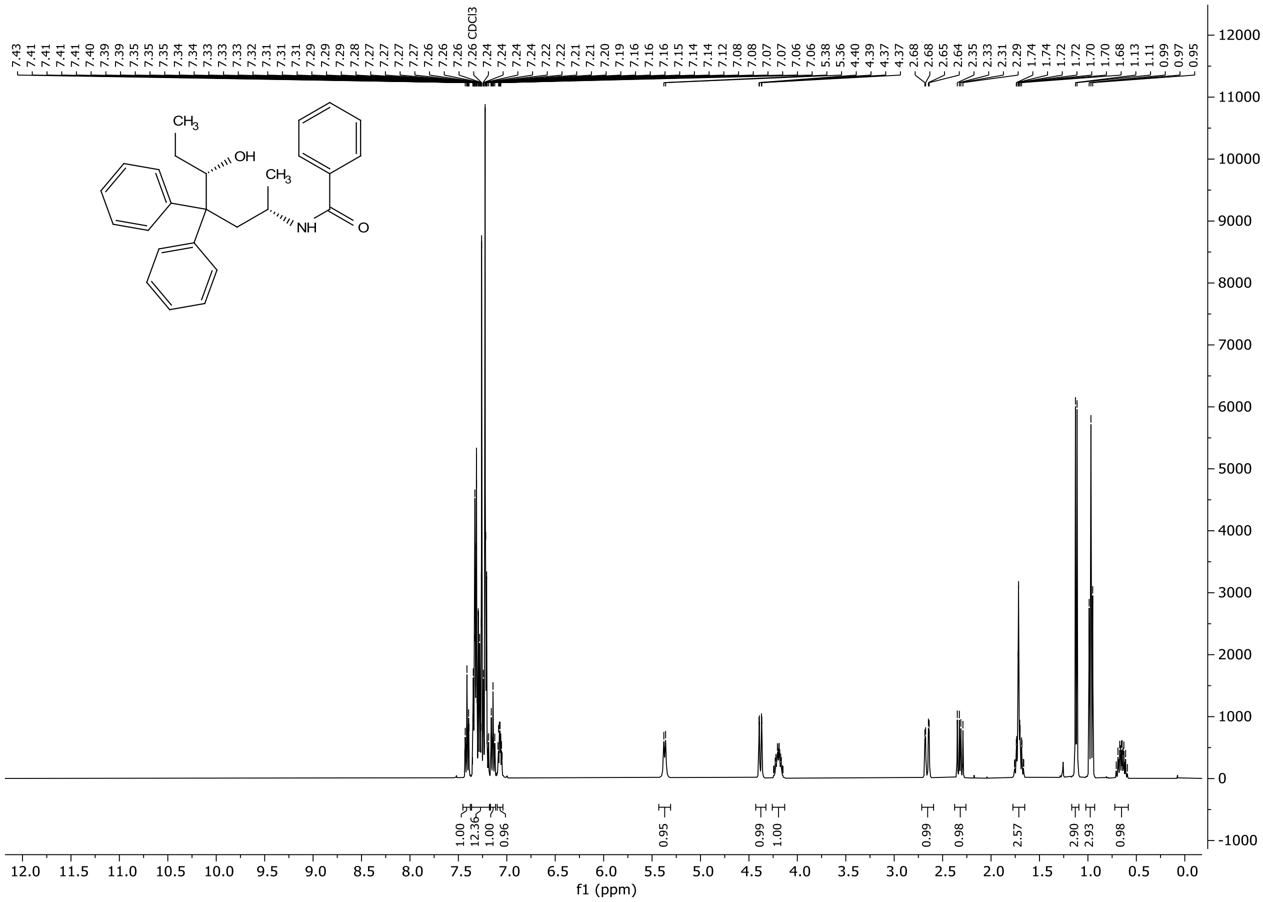

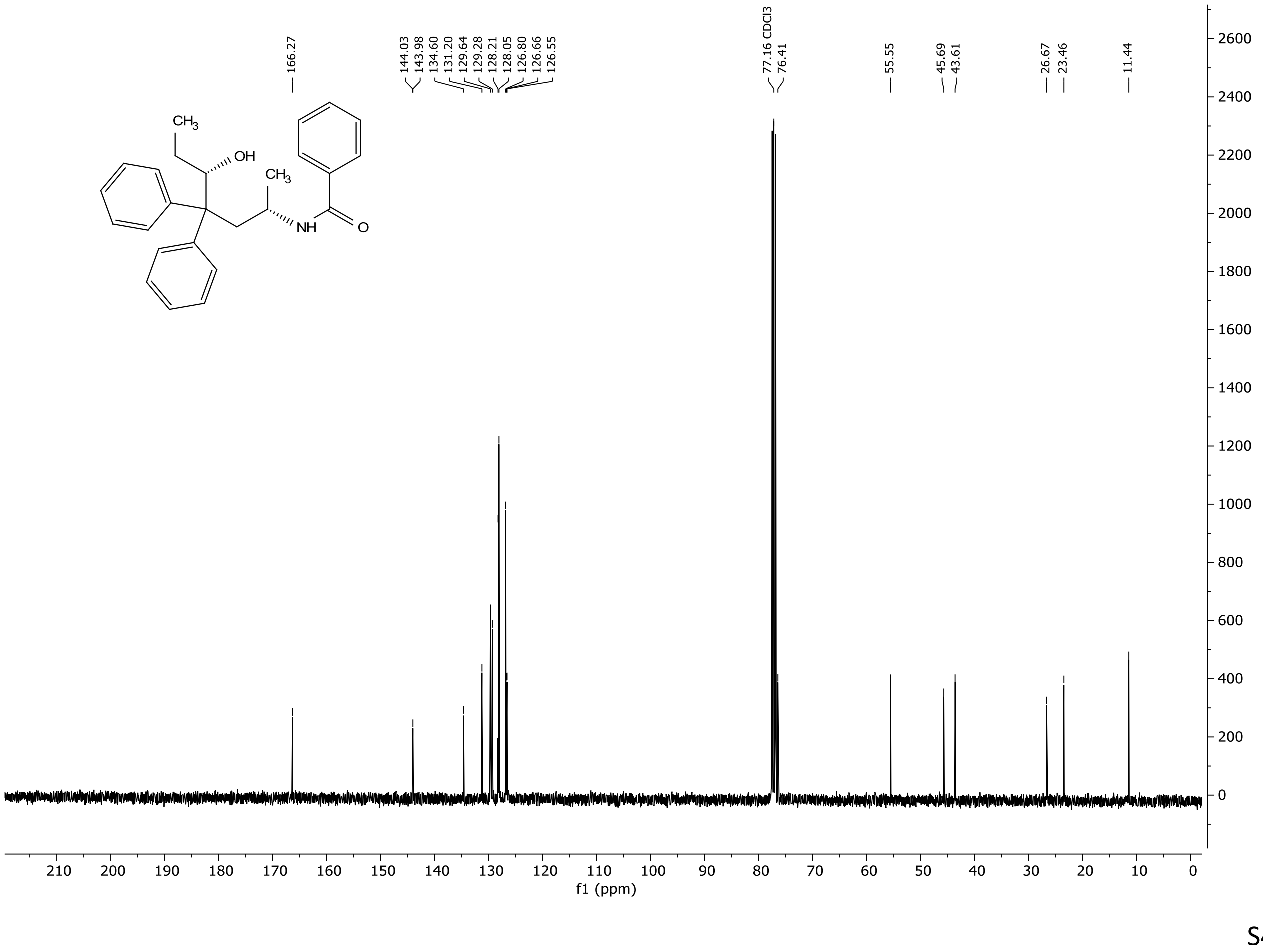

Chiral HPLC chromatogram of (3*S*,6*S*)-6-(benzamido)-4,4-diphenylheptan-3-ol ((3*S*,6*S*)-**8**)

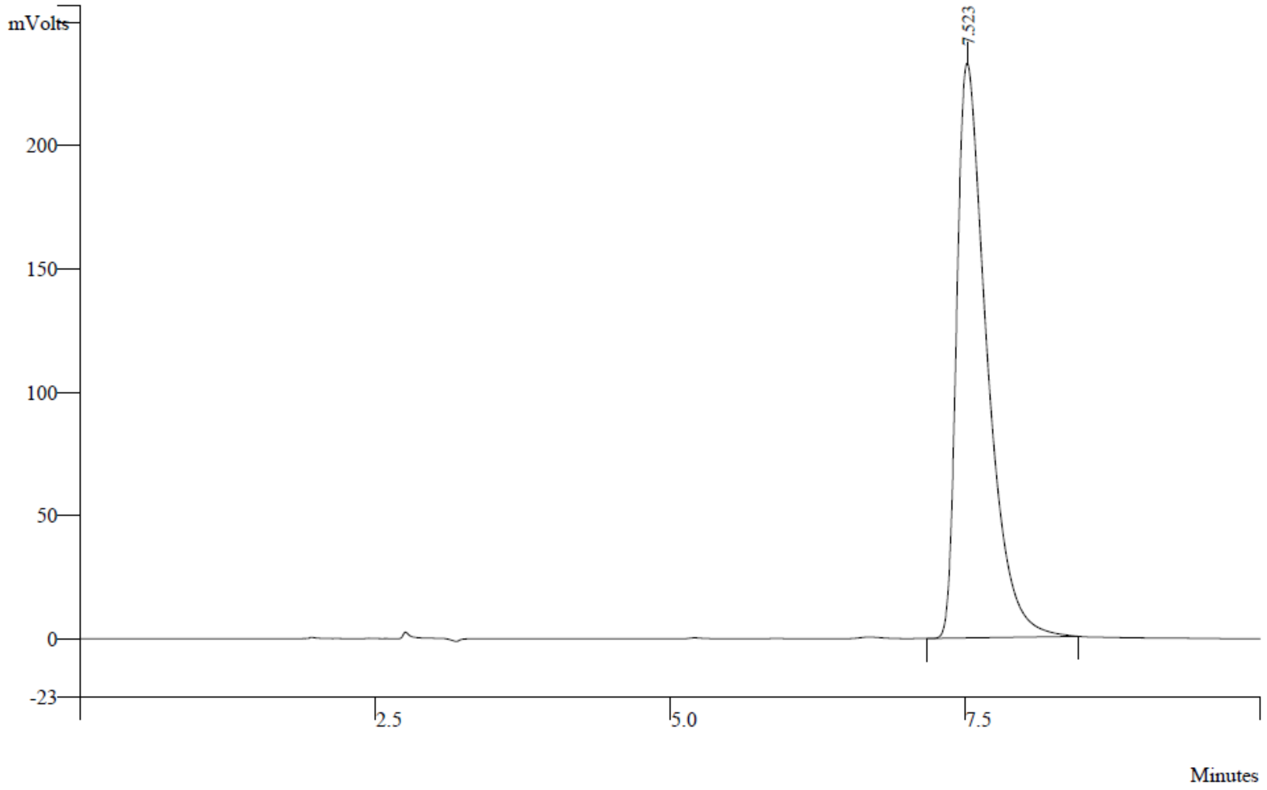

Peak No	Ret Time (min)	Peak Area (counts)	Result ()
1	7,523	4135760	100,00
		**4135760**	**100,00**

^{1}H and ^{13}C NMR spectra of (3*R*,6*R*)-6-(benzamido)-4,4-diphenylheptan-3-ol ((3*R*,6*R*)-**8**)

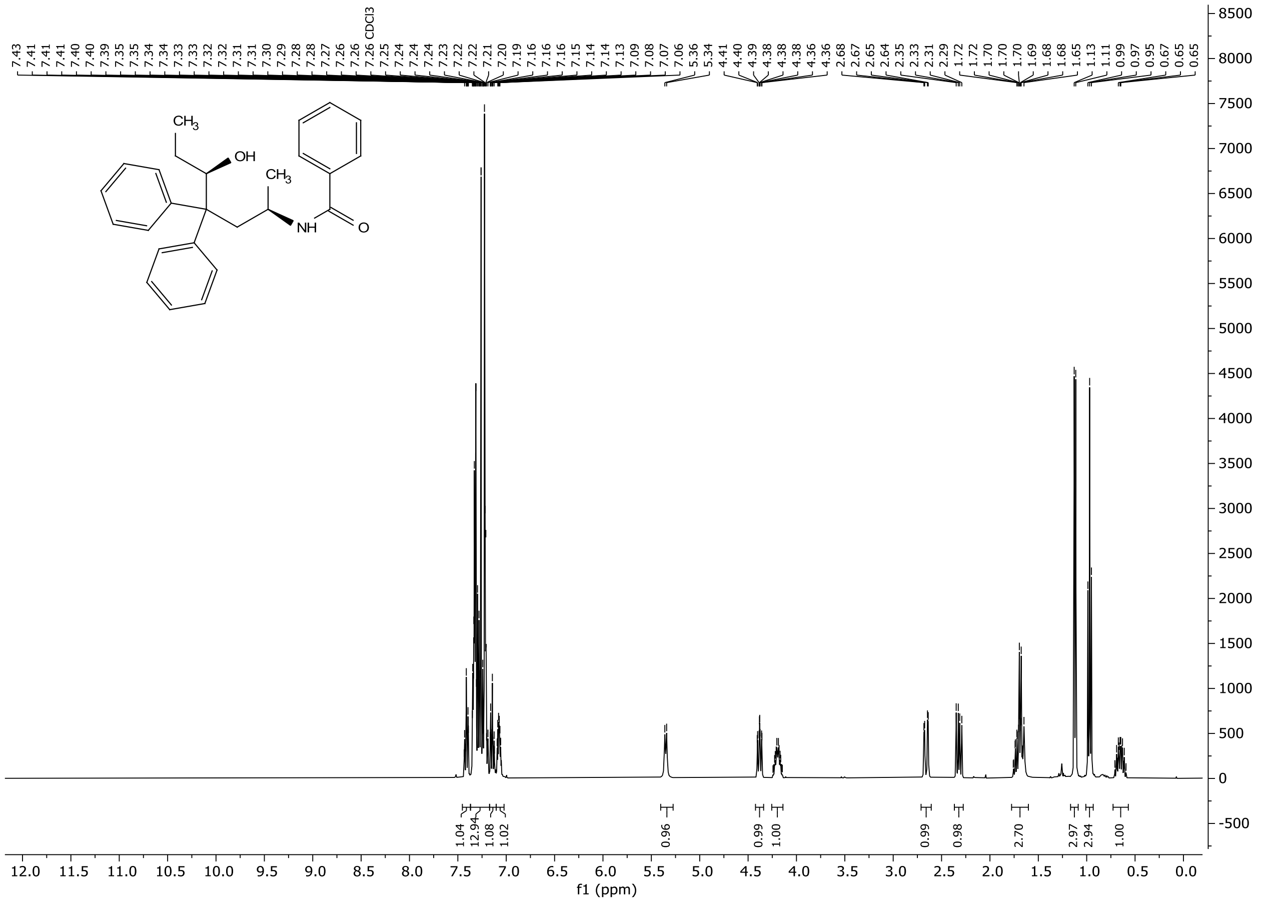

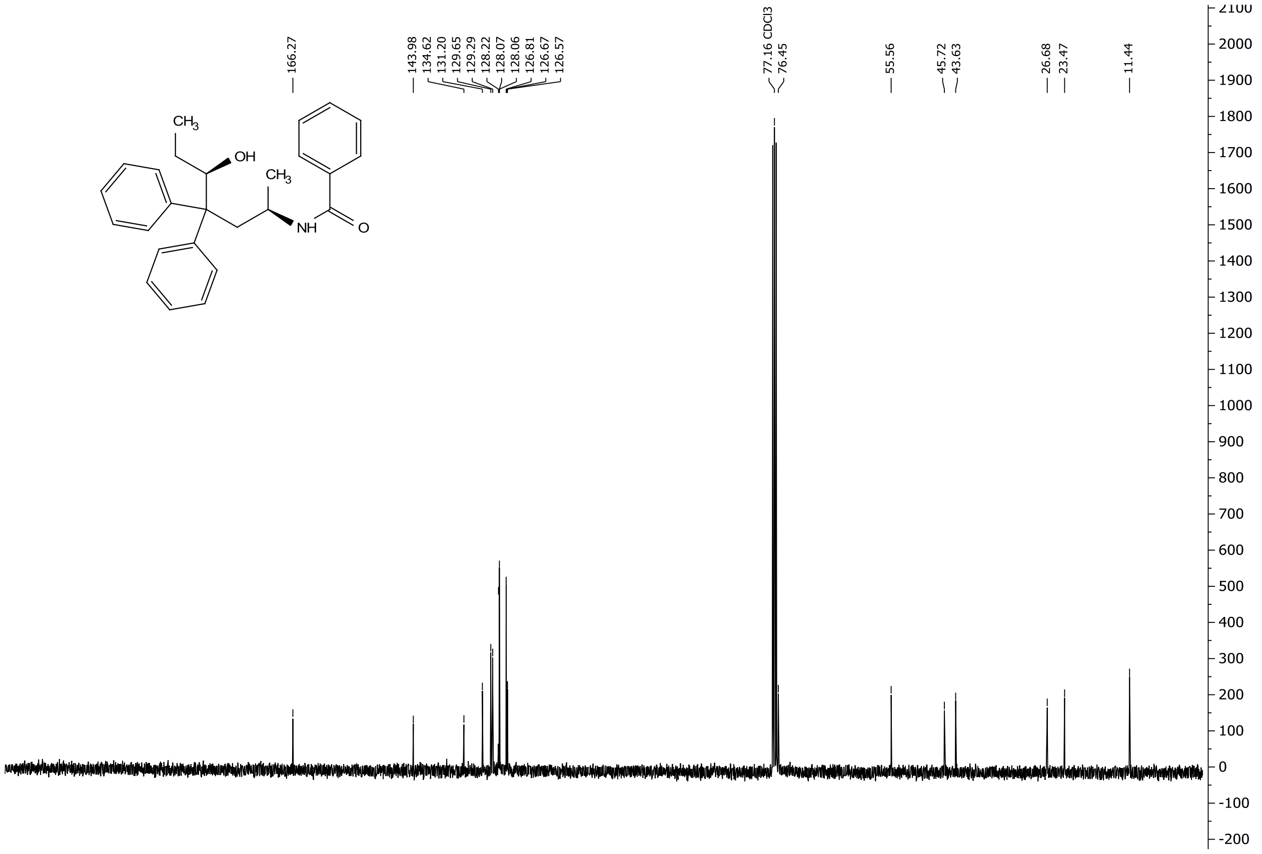

Chiral HPLC chromatogram of (3*R*,6*R*)-6-(benzamido)-4,4-diphenylheptan-3-ol ((3*R*,6*R*)-**8**)

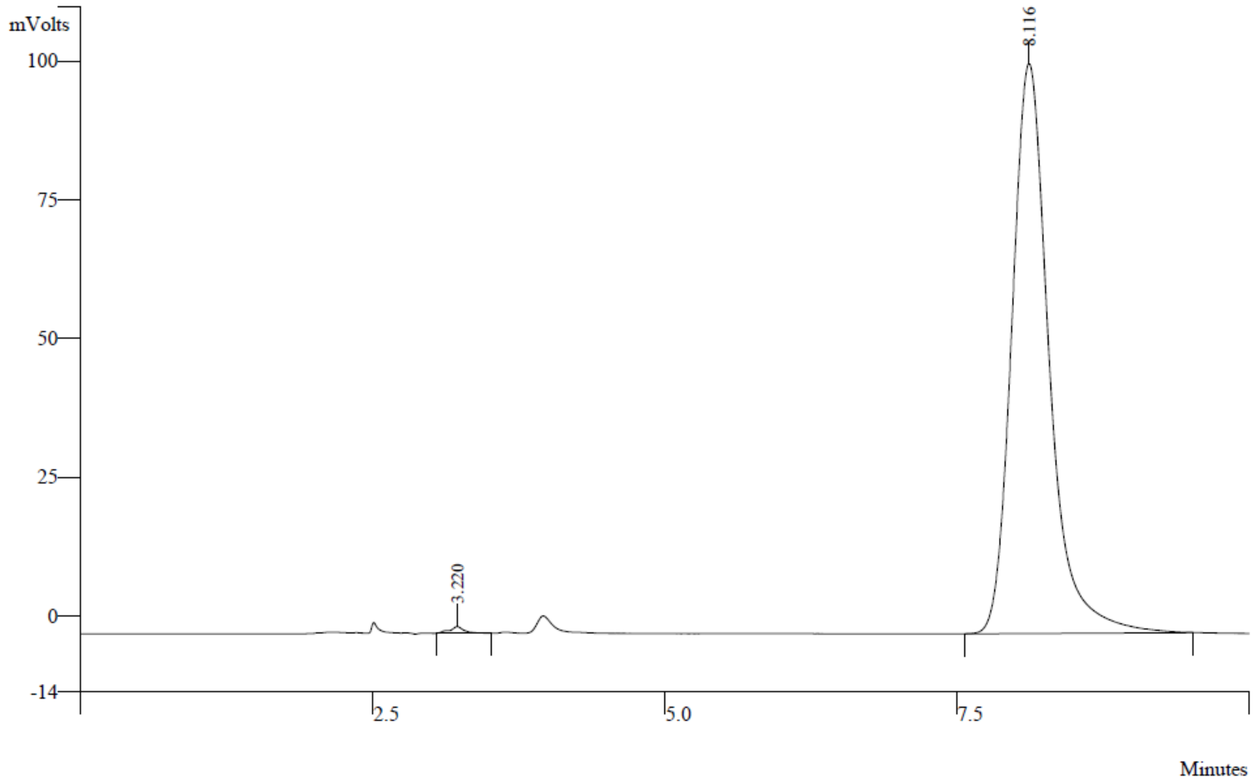

Peak No	Ret Time (min)	Peak Area (counts)	Result ()
1	3,220	10566	0,46
2	8,116	2279687	99,54
		**2290253**	**100,00**

^{1}H and ^{13}C NMR spectra of (3*S*,6*S*)-6-(benzylamino)-4,4-diphenylheptan-3-ol (3*S*,6*S*)-**9**

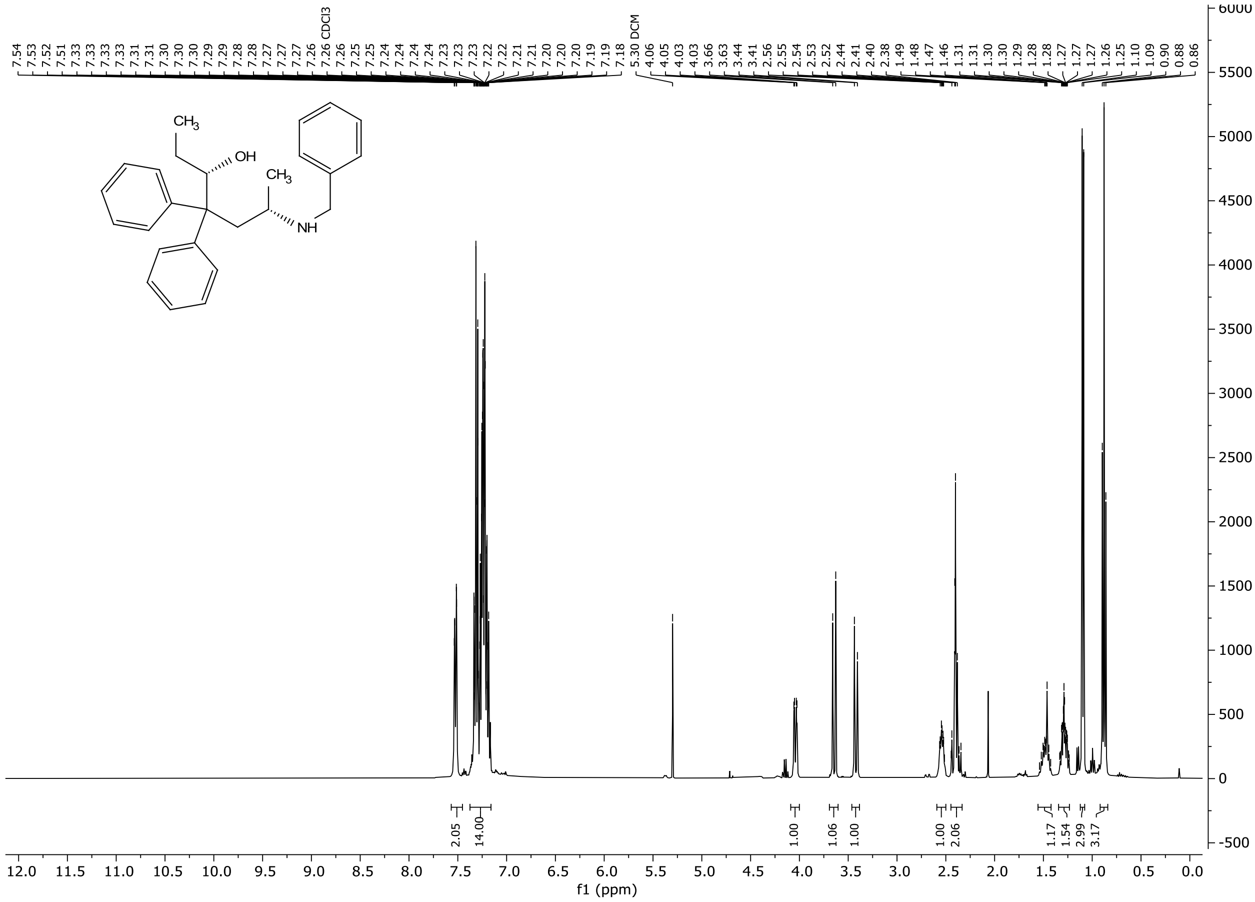

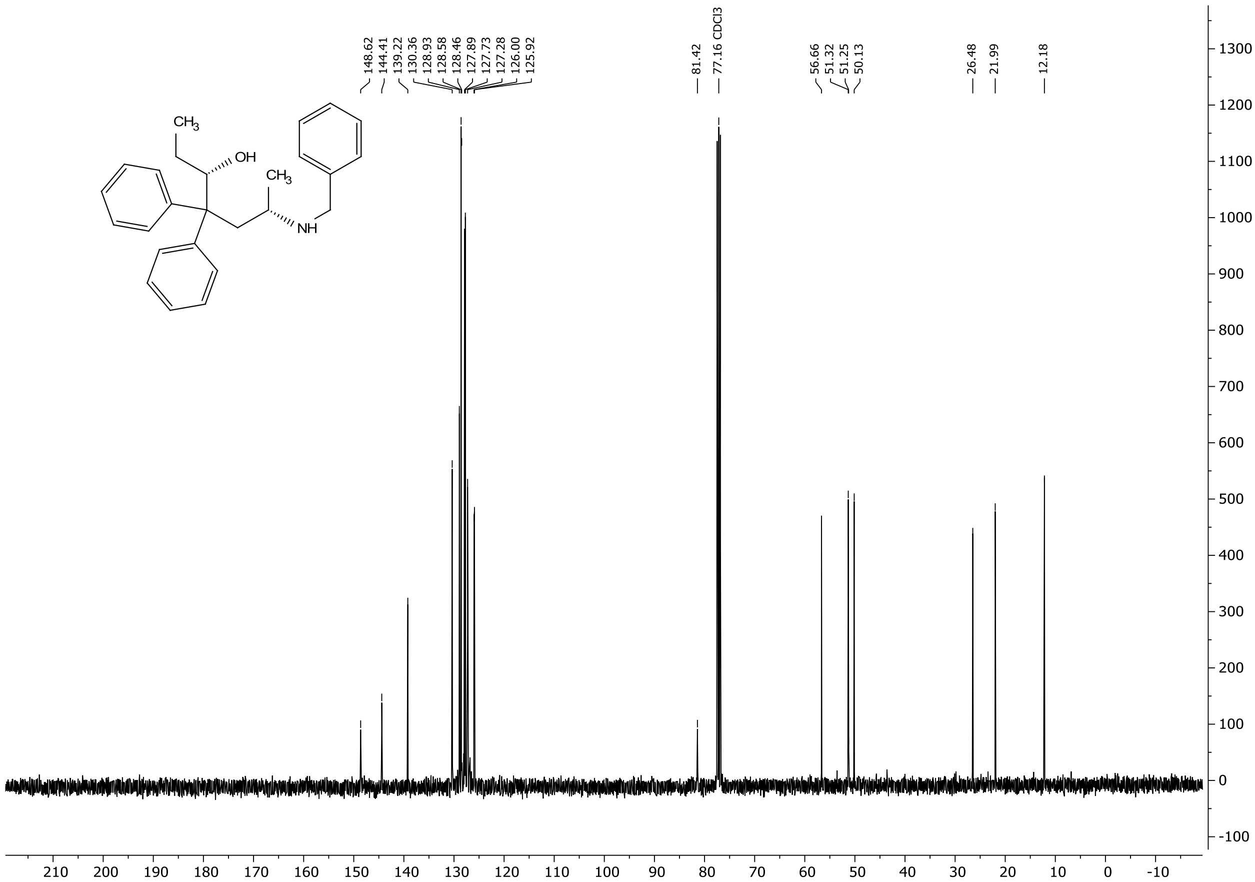

^{1}H and ^{13}C NMR spectra of (3*R*,6*R*)-6-(benzylamino)-4,4-diphenylheptan-3-ol (3*R*,6*R*)-**9**

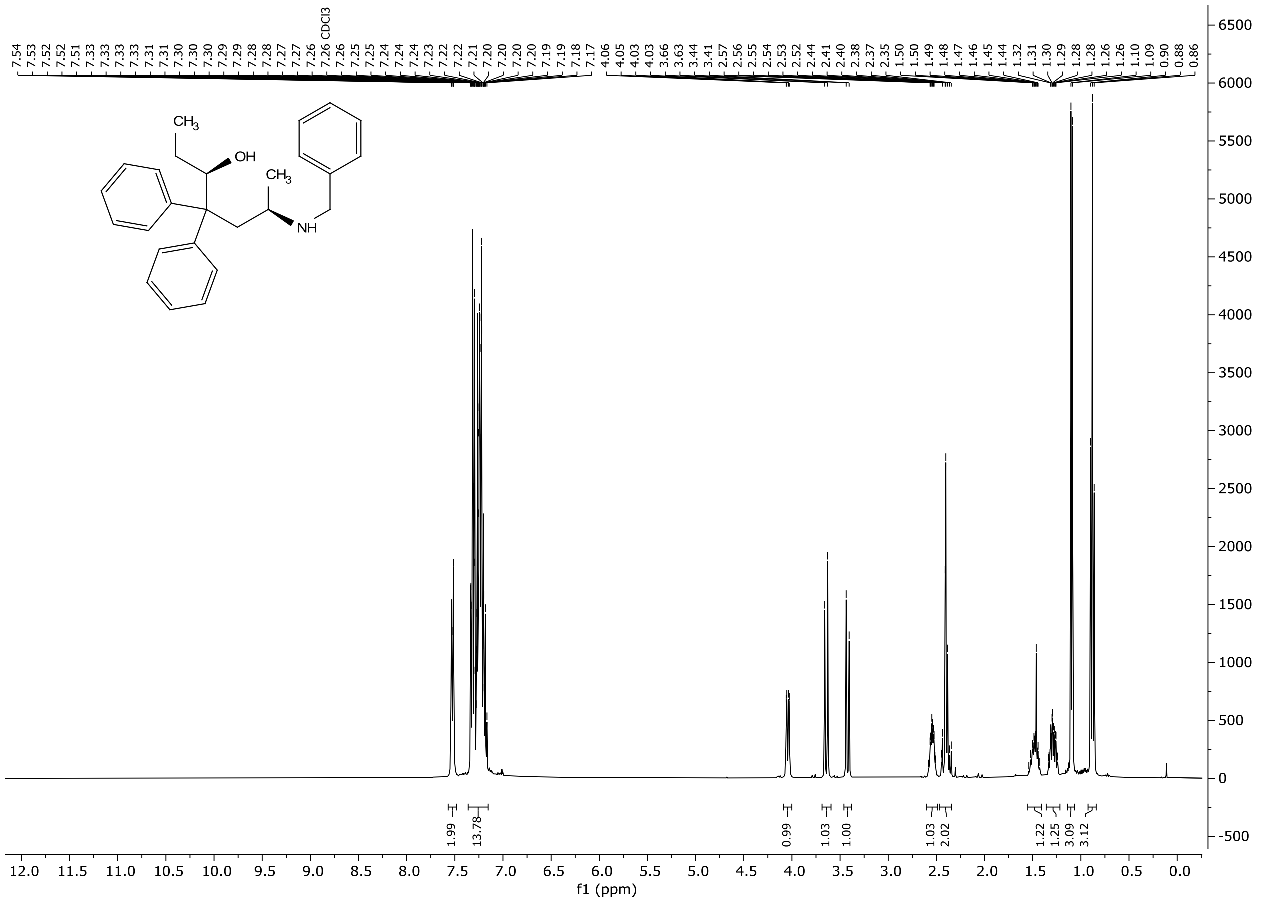

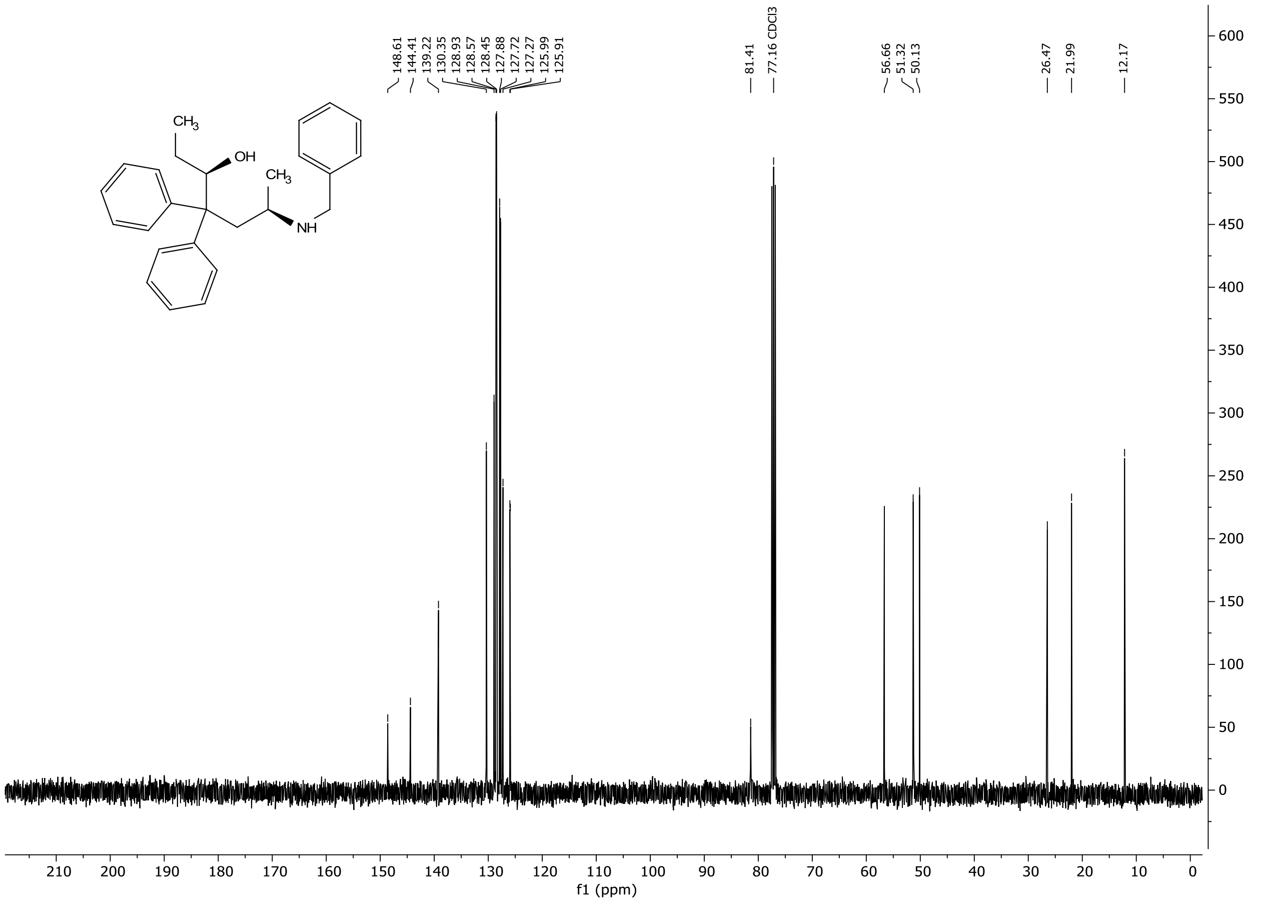

^{1}H and ^{13}C NMR spectra of (3*R*,6*S*)-6-(benzylamino)-4,4-diphenylheptan-3-ol (3*R*,6*S*)-**9**

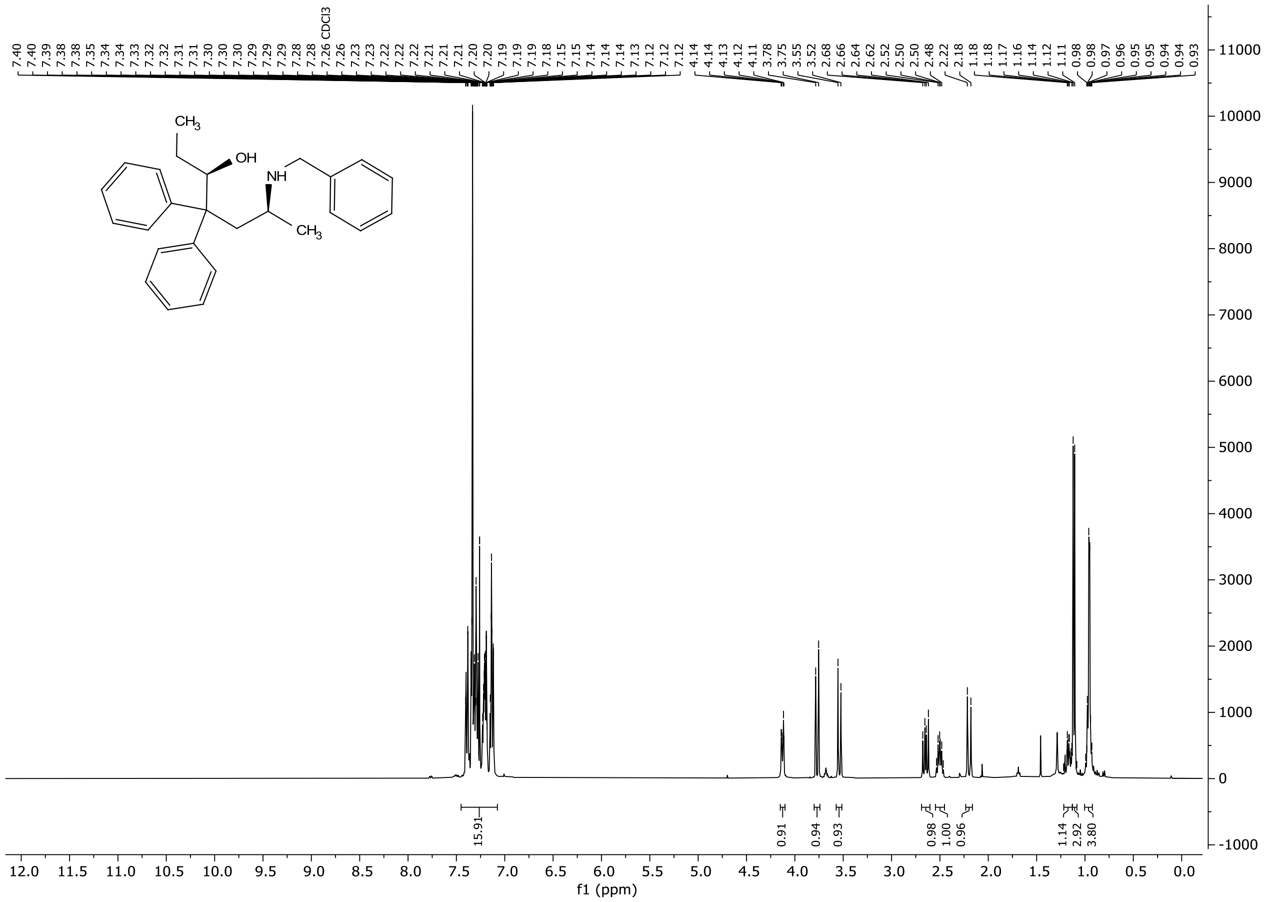

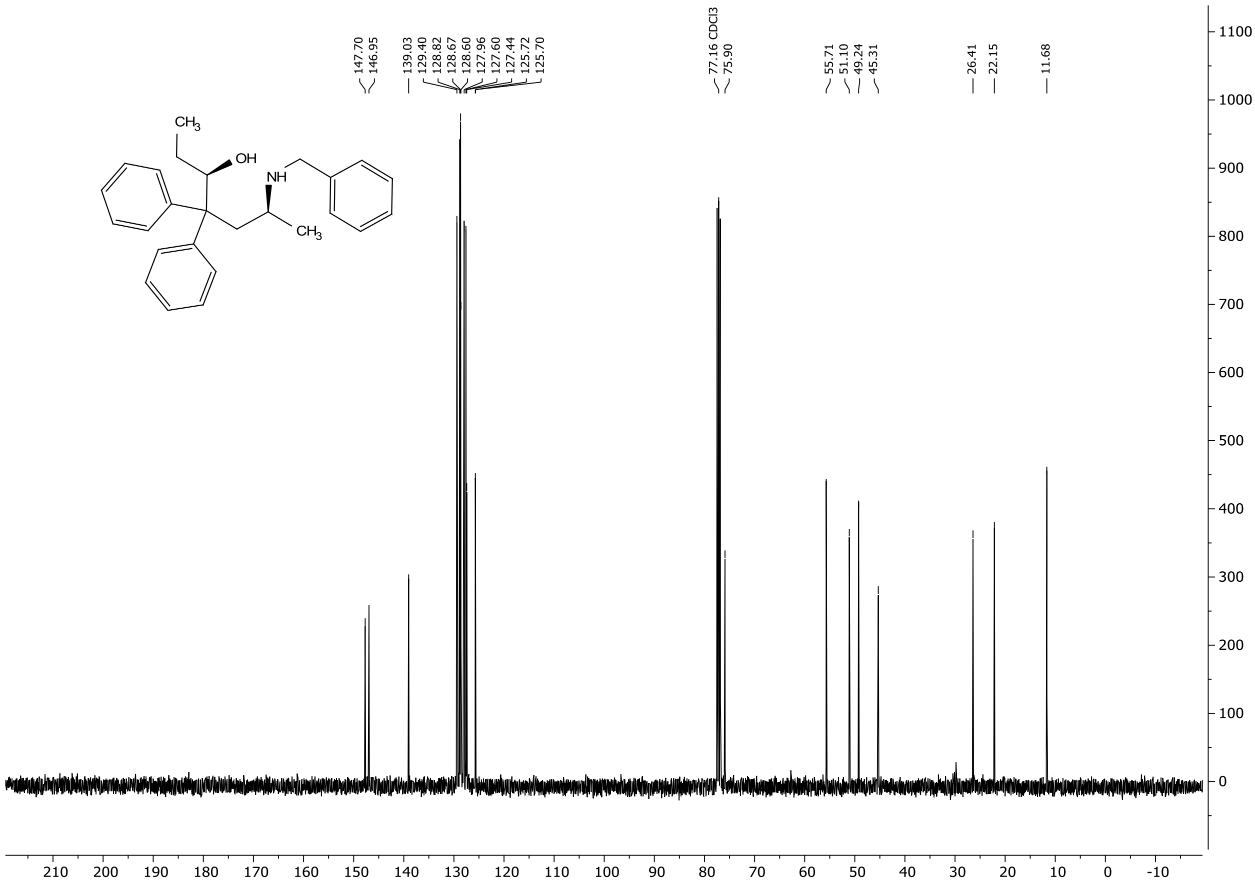

^{1}H and ^{13}C NMR spectra of (3*S*,6*R*)-6-(benzylamino)-4,4-diphenylheptan-3-ol (3*S*,6*R*)-**9**

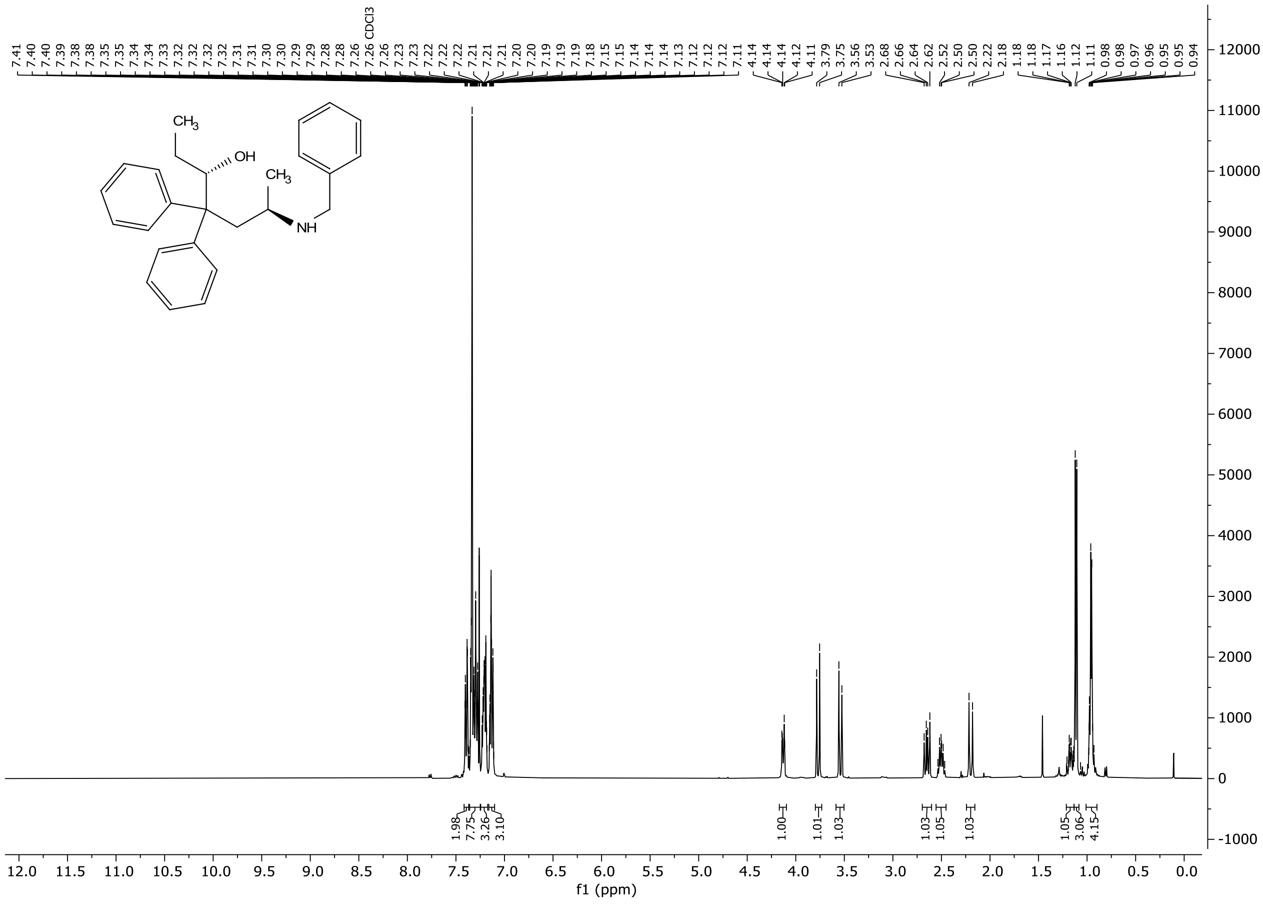

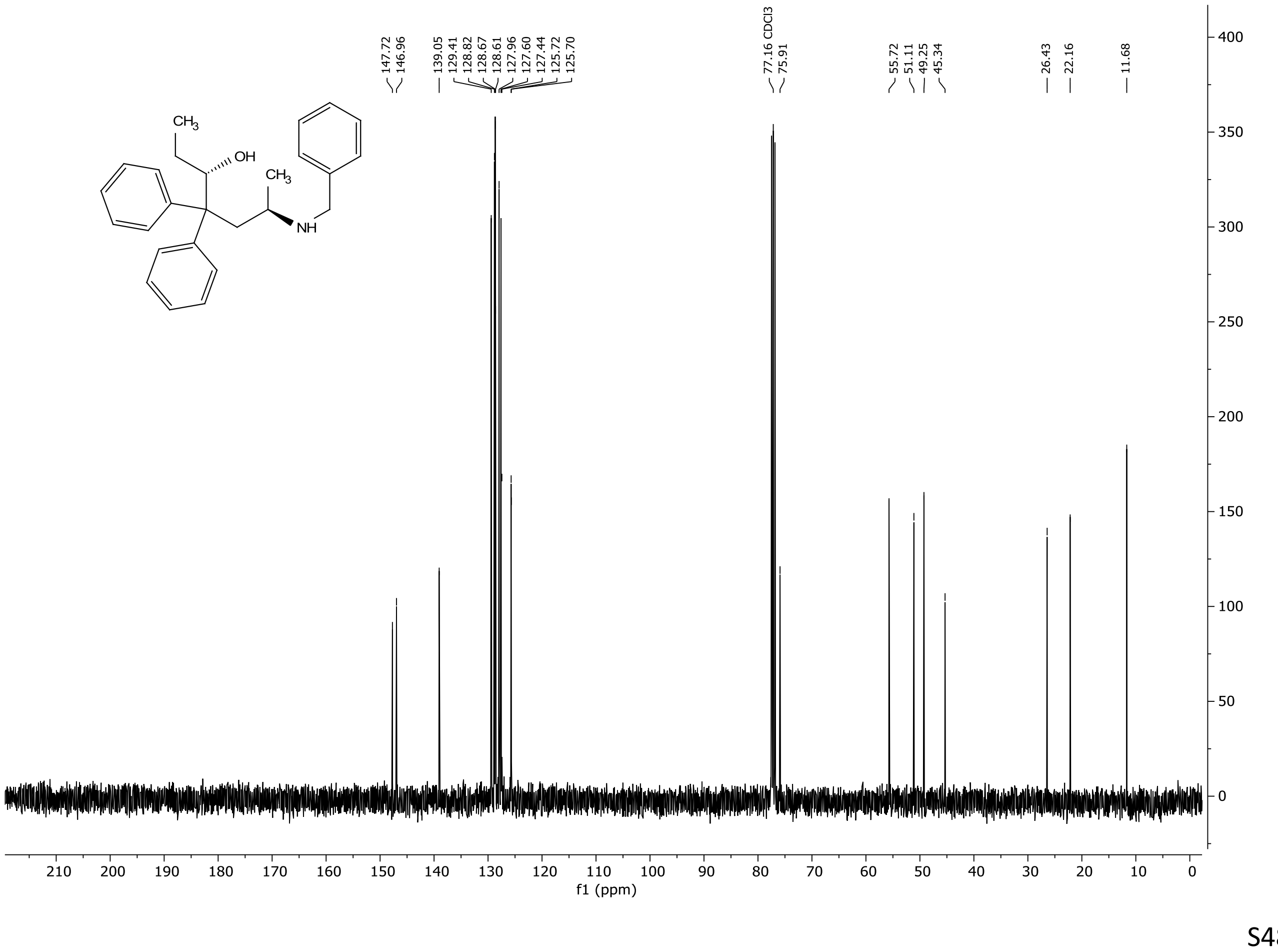

^{1}H and ^{13}C NMR spectra of (3*R*,6*R*)-6-(benzyl(methyl)amino)-4,4-diphenylheptan-3-ol ((3*R*,6*R*)-**10**)

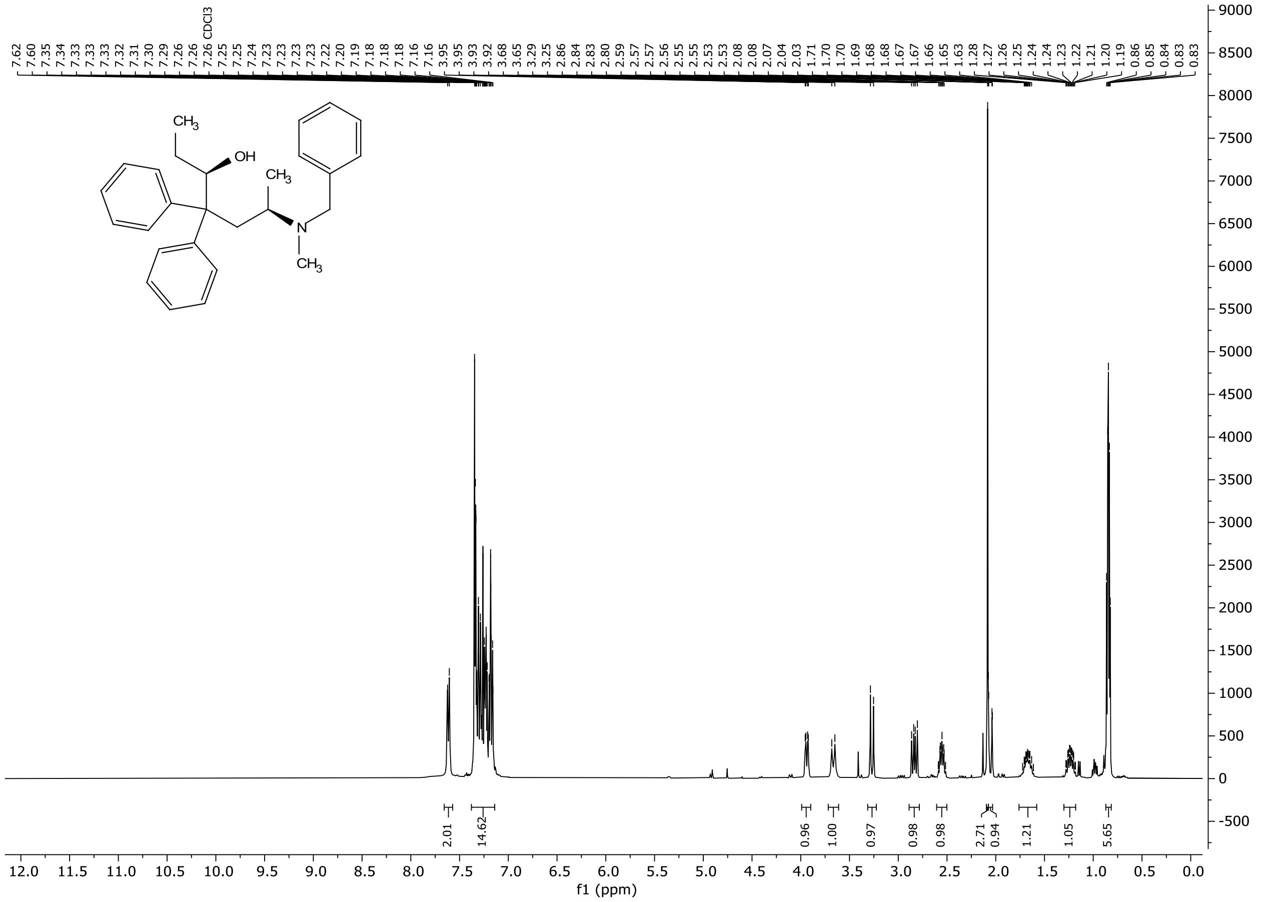

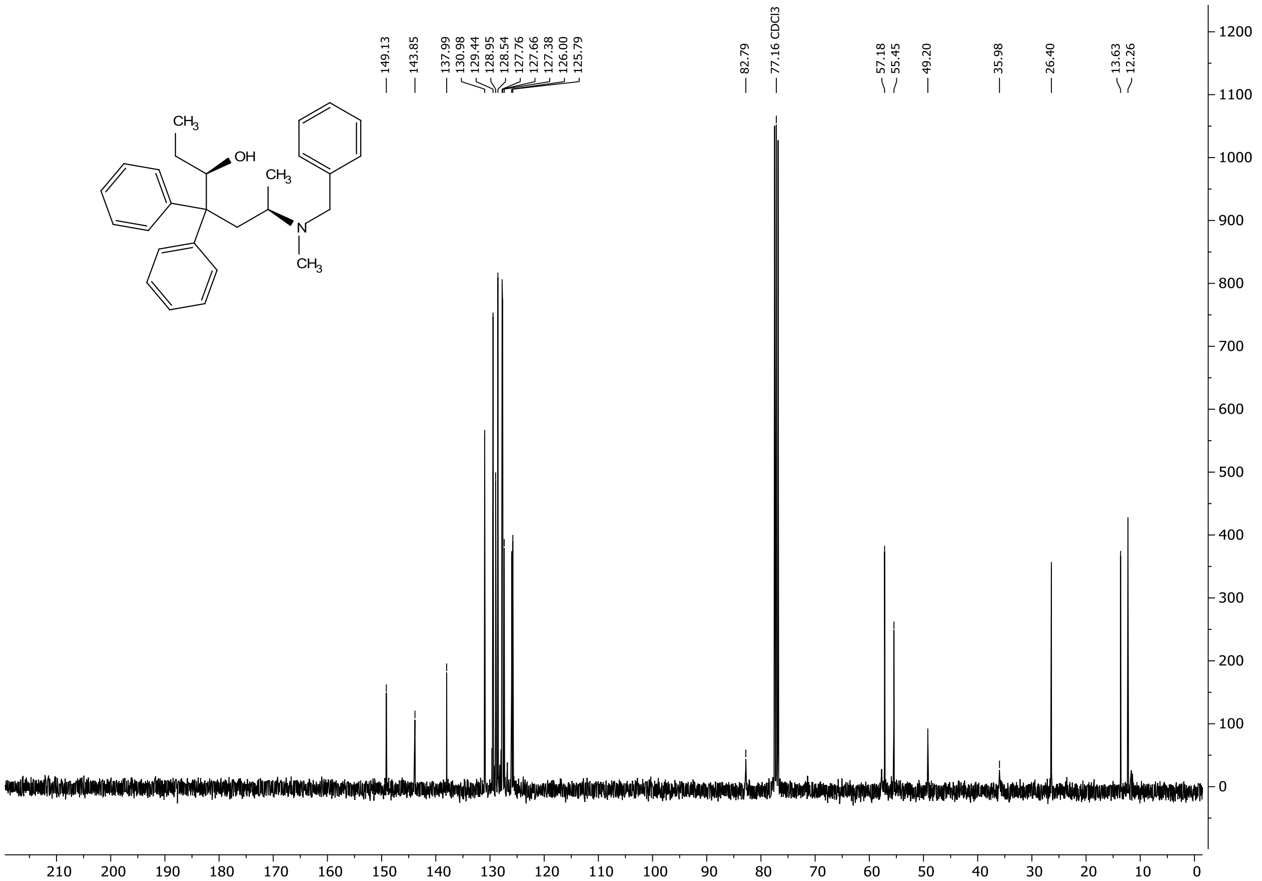

^{1}H and ^{13}C NMR spectra of (3*S*,6*S*)-6-(benzyl(methyl)amino)-4,4-diphenylheptan-3-ol ((3*S*,6*S*)-**10**)

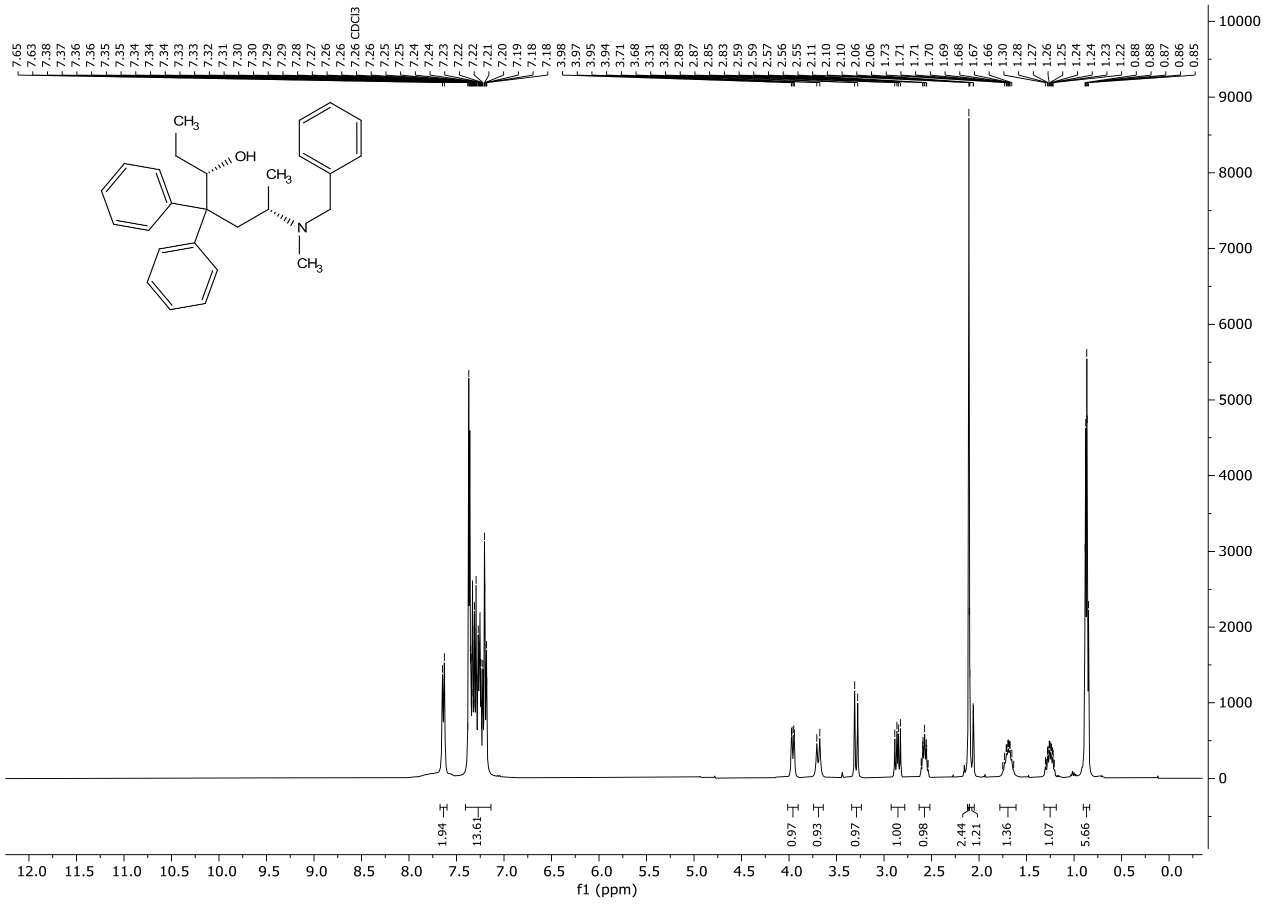

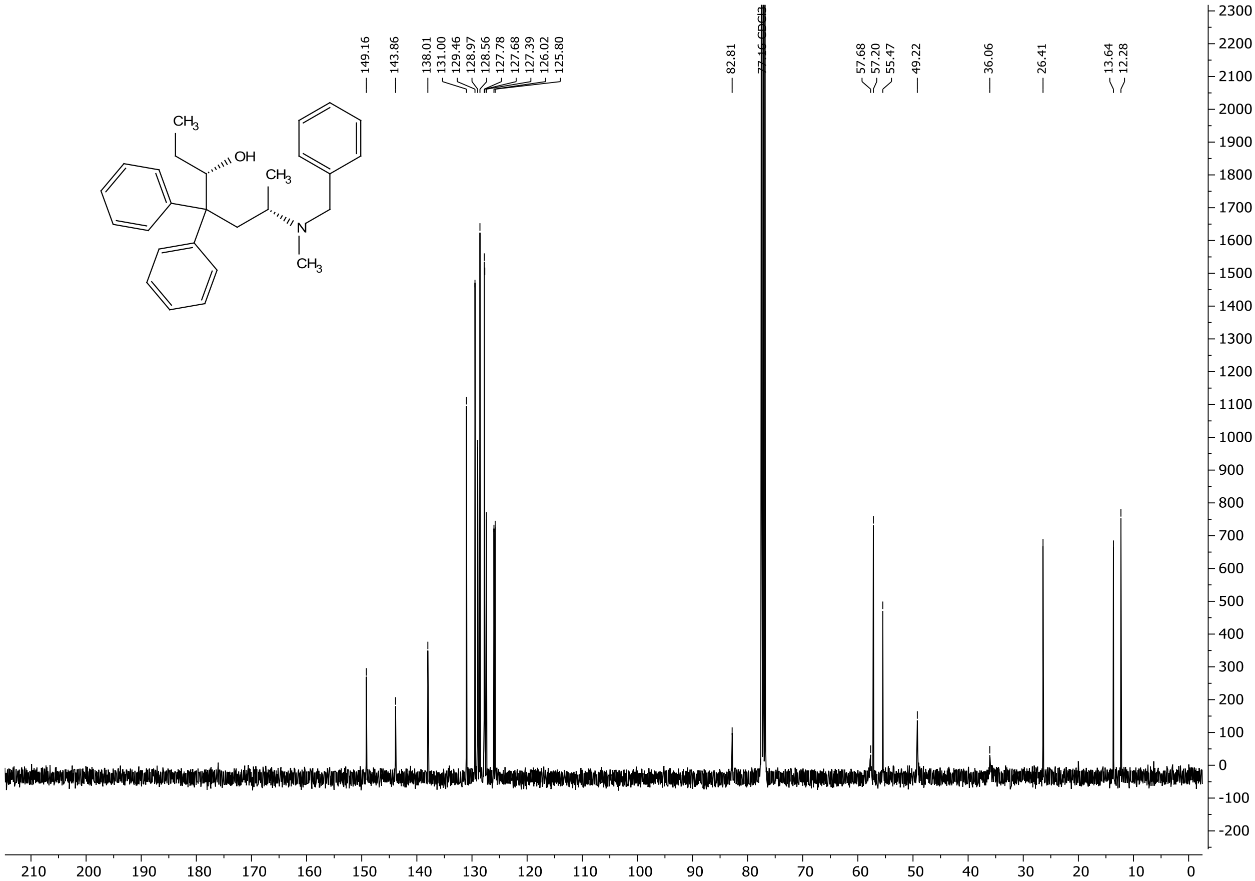

^{1}H and ^{13}C NMR spectra of (3*R*,6*S*)-6-(benzyl(methyl)amino)-4,4-diphenylheptan-3-ol ((3*R*,6*S*)-**10**)

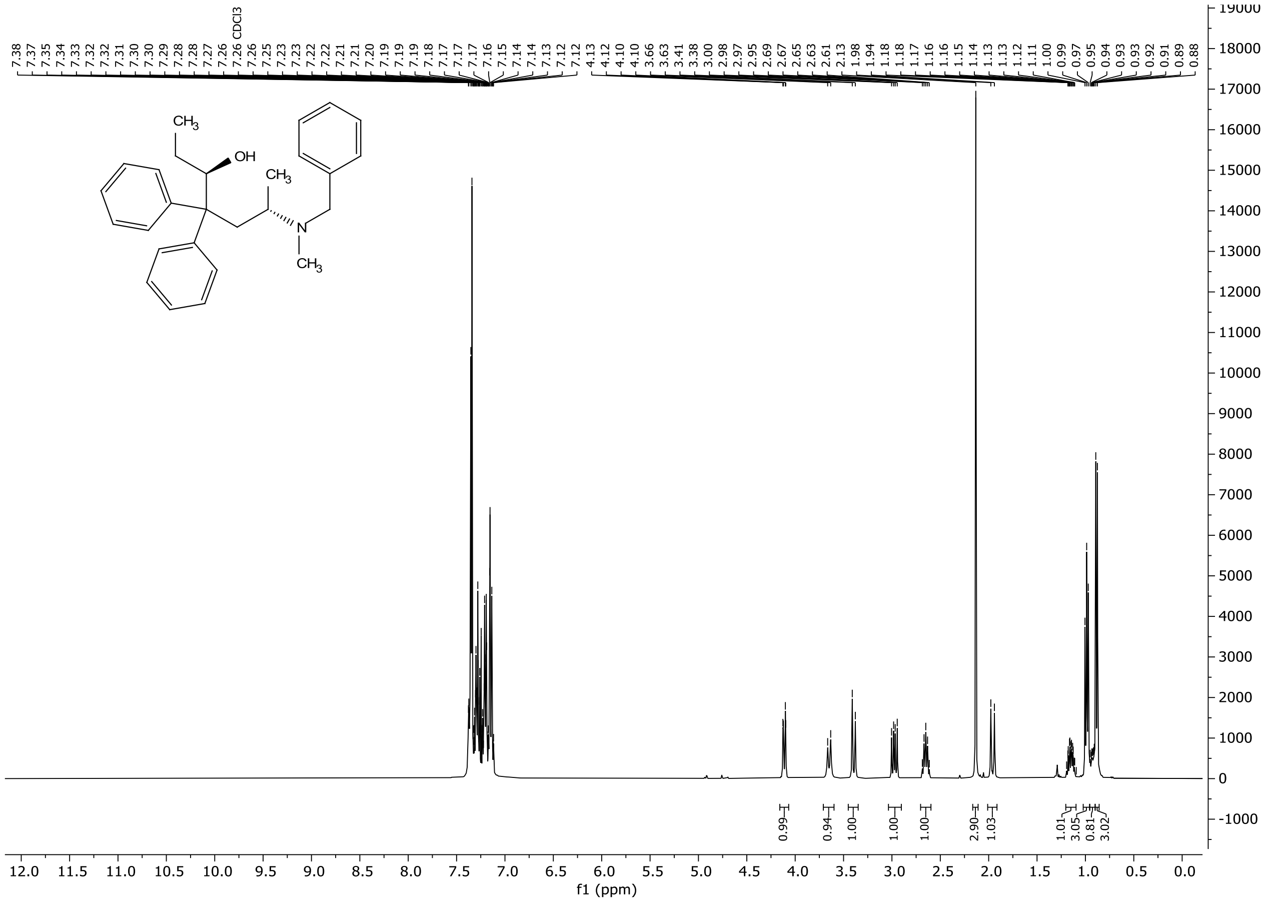

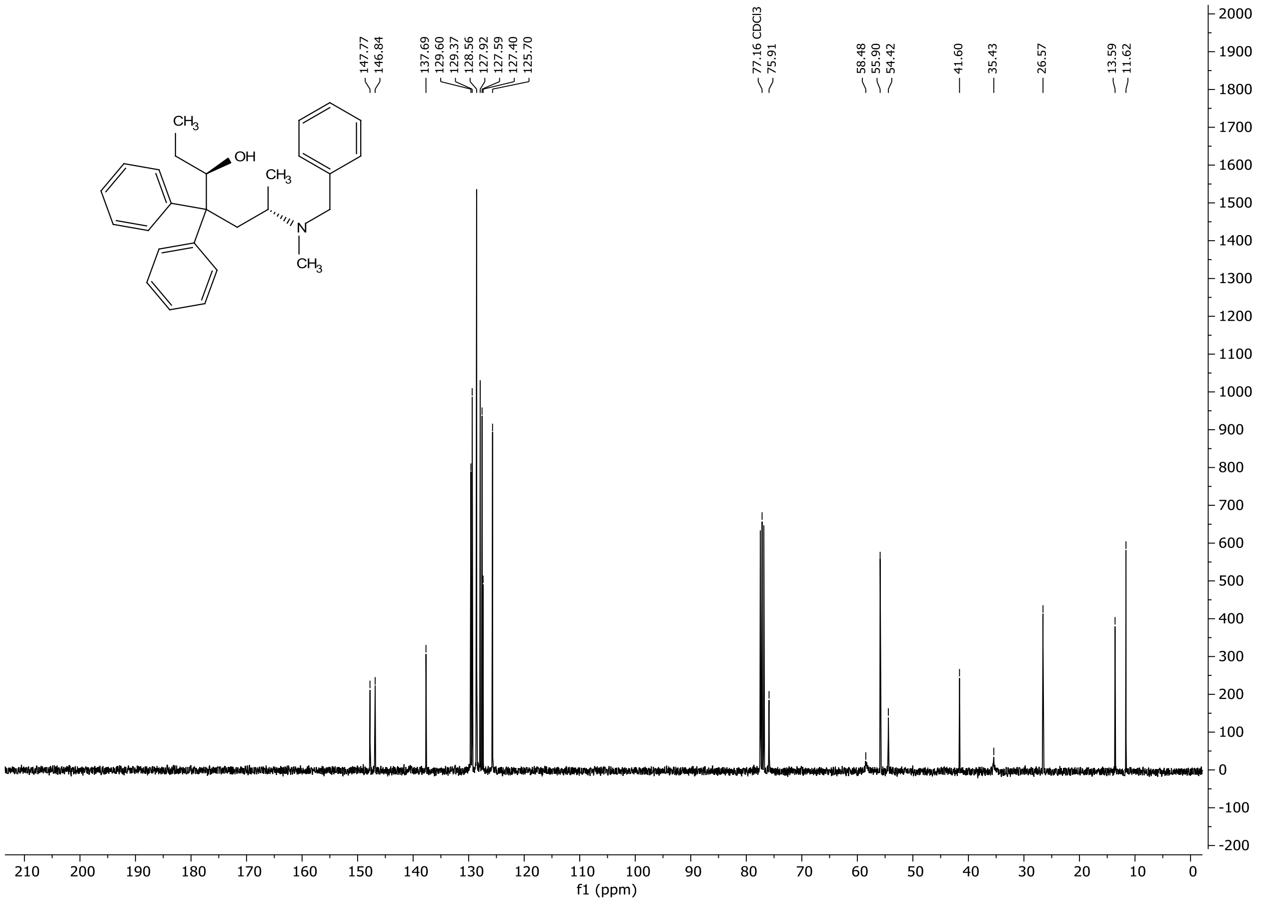

^{1}H and ^{13}C NMR spectra of (3*S*,6*R*)-6-(benzyl(methyl)amino)-4,4-diphenylheptan-3-ol ((3*S*,6*R*)-**10**)

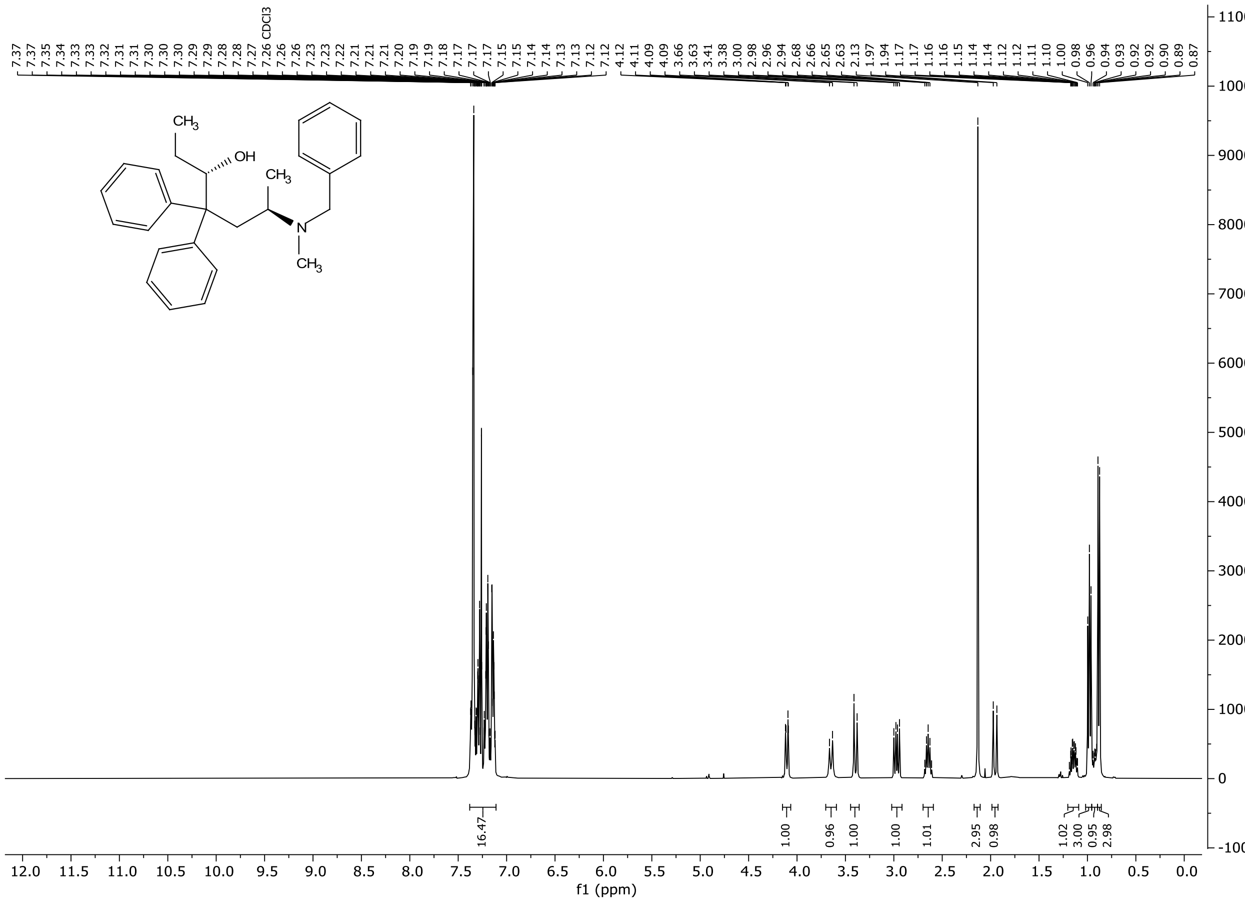

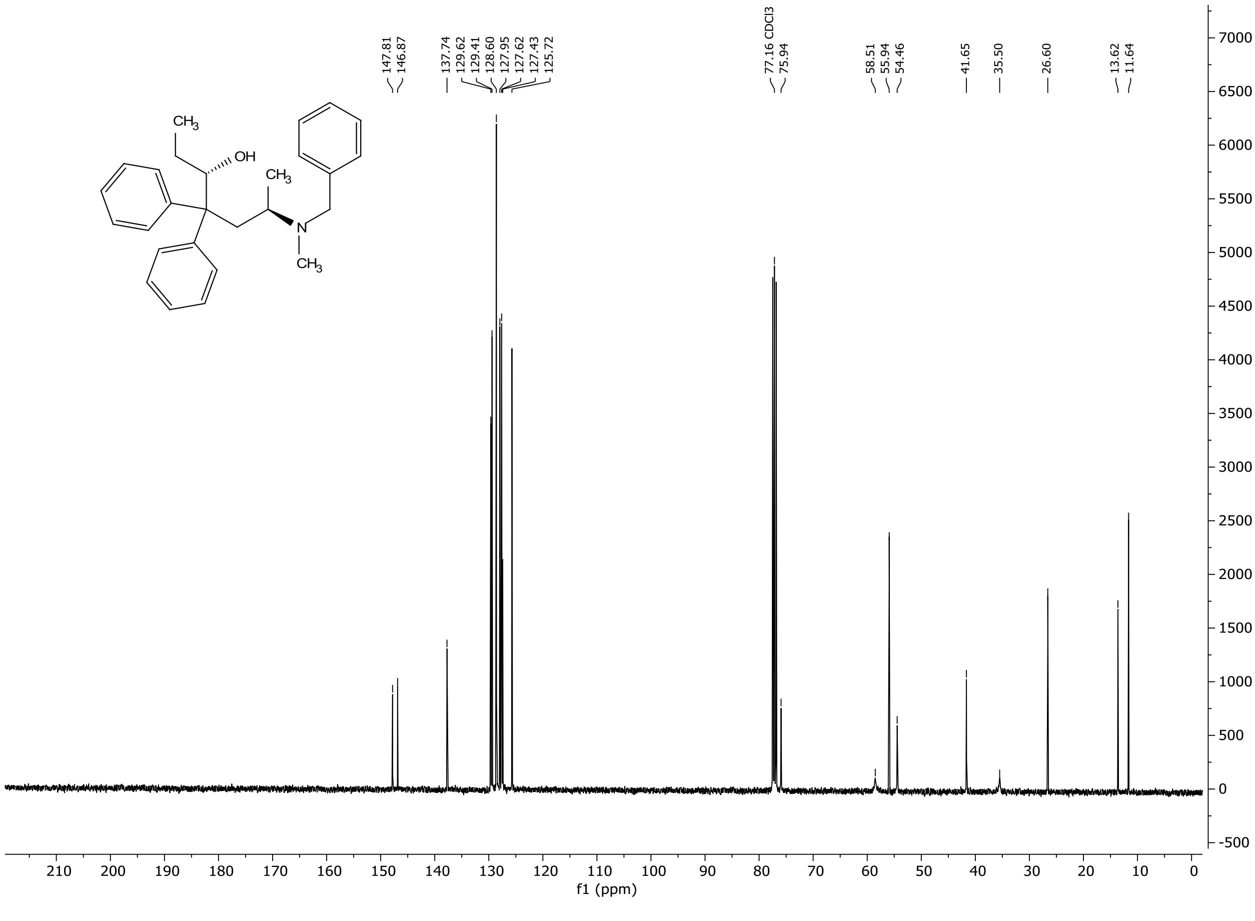

^{1}H and ^{13}C NMR spectra of (3*R*,6*R*)-6-(dimethylamino)-4,4-diphenylheptan-3-ol ((3*R*,6*R*)-**ML**)

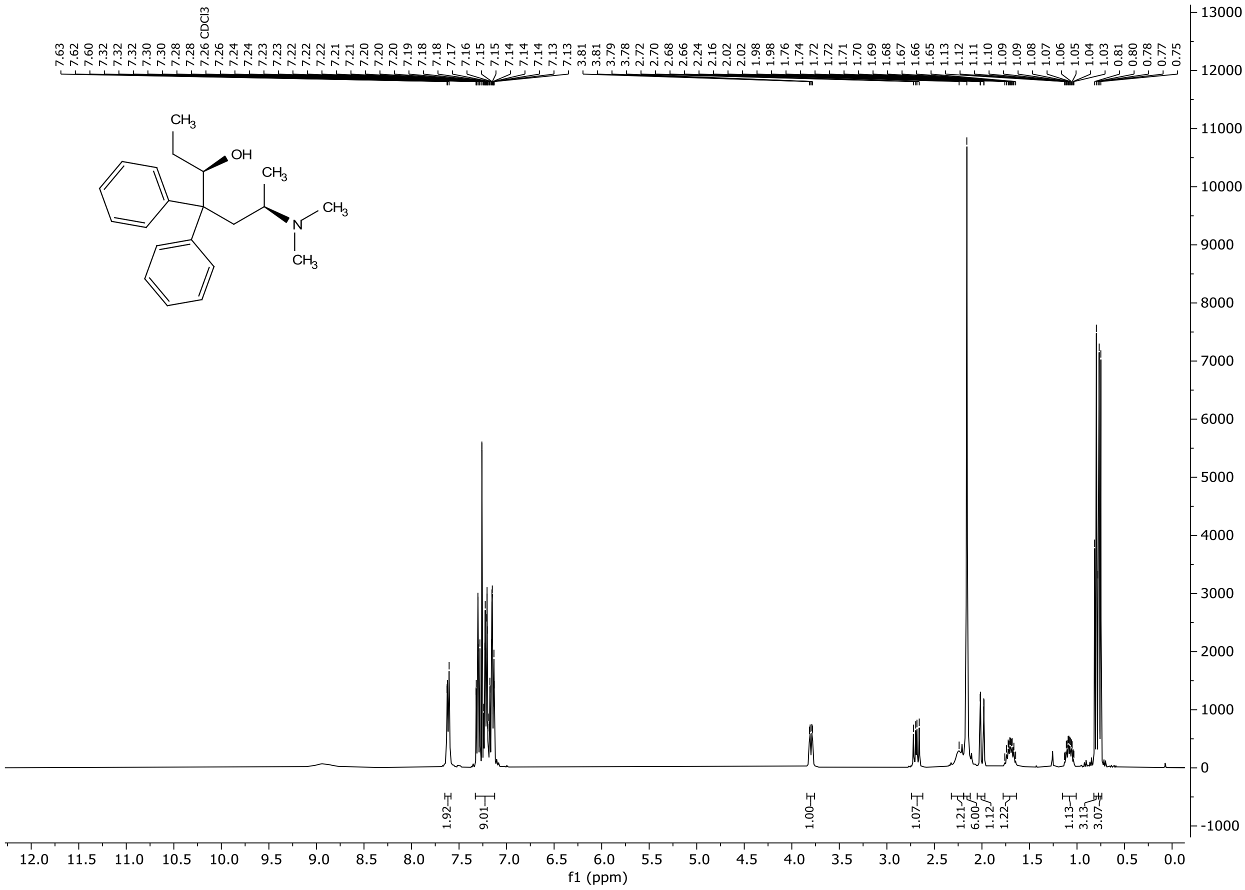

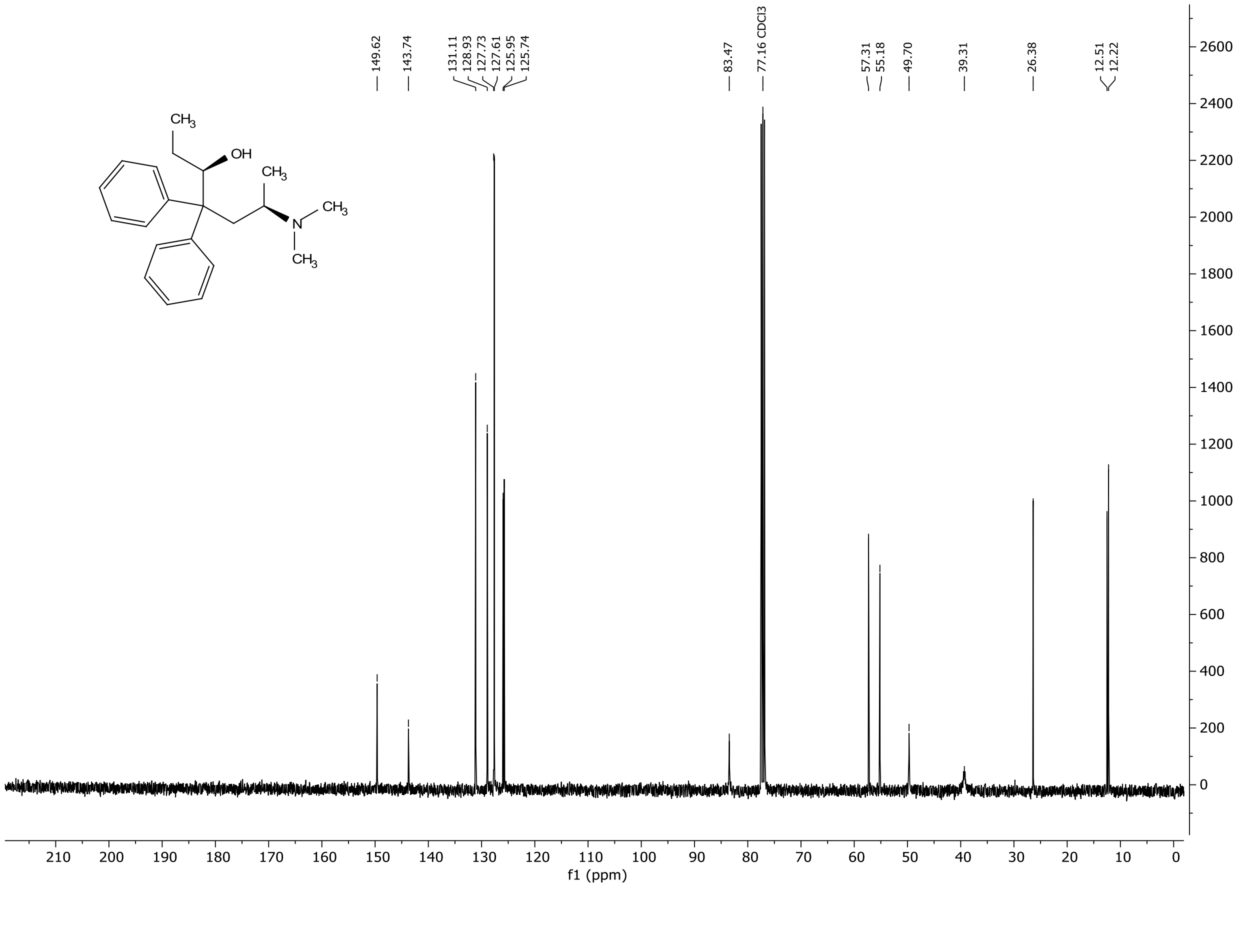

UPLC chromatogram of (3*R*,6*R*)-6-(dimethylamino)-4,4-diphenylheptan-3-ol ((3*R*,6*R*)-**ML**)

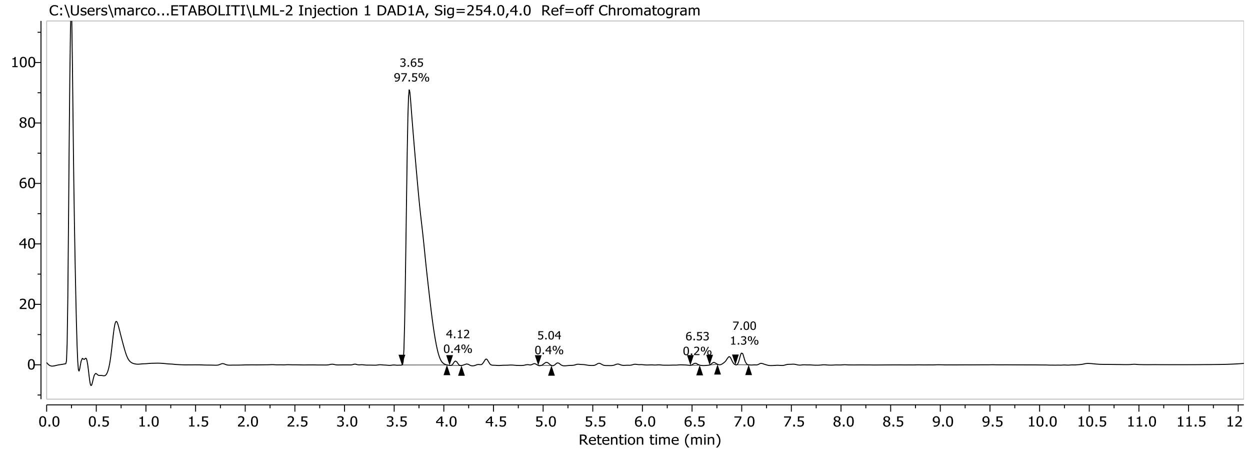

Chiral HPLC chromatogram of (3*R*,6*R*)-6-(dimethylamino)-4,4-diphenylheptan-3-ol ((3*R*,6*R*)-**ML**)

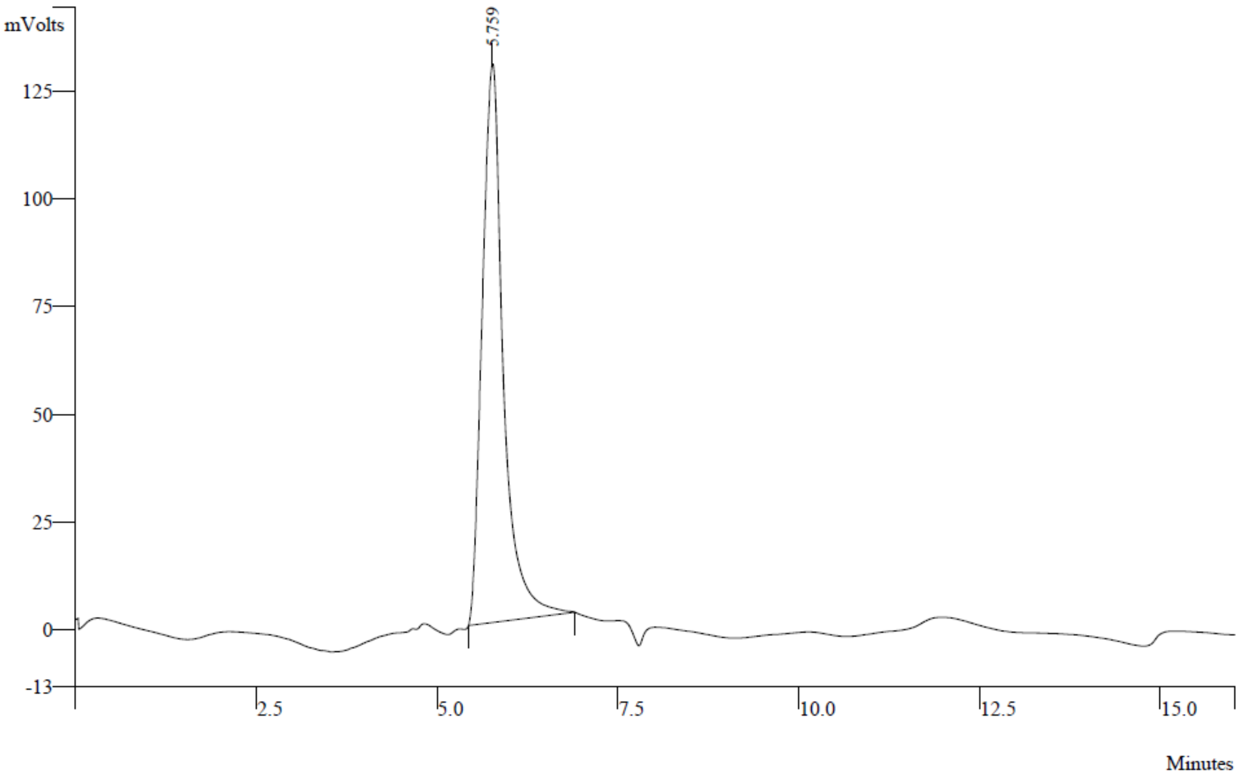

Peak No	Ret Time (min)	Peak Area (counts)	Result ()
1	5,759	2635491	100,00
		**2635491**	**100,00**

^{1}H and ^{13}C NMR spectra of (3*S*,6*S*)-6-(dimethylamino)-4,4-diphenylheptan-3-ol ((3*S*,6*S*)-**ML**)

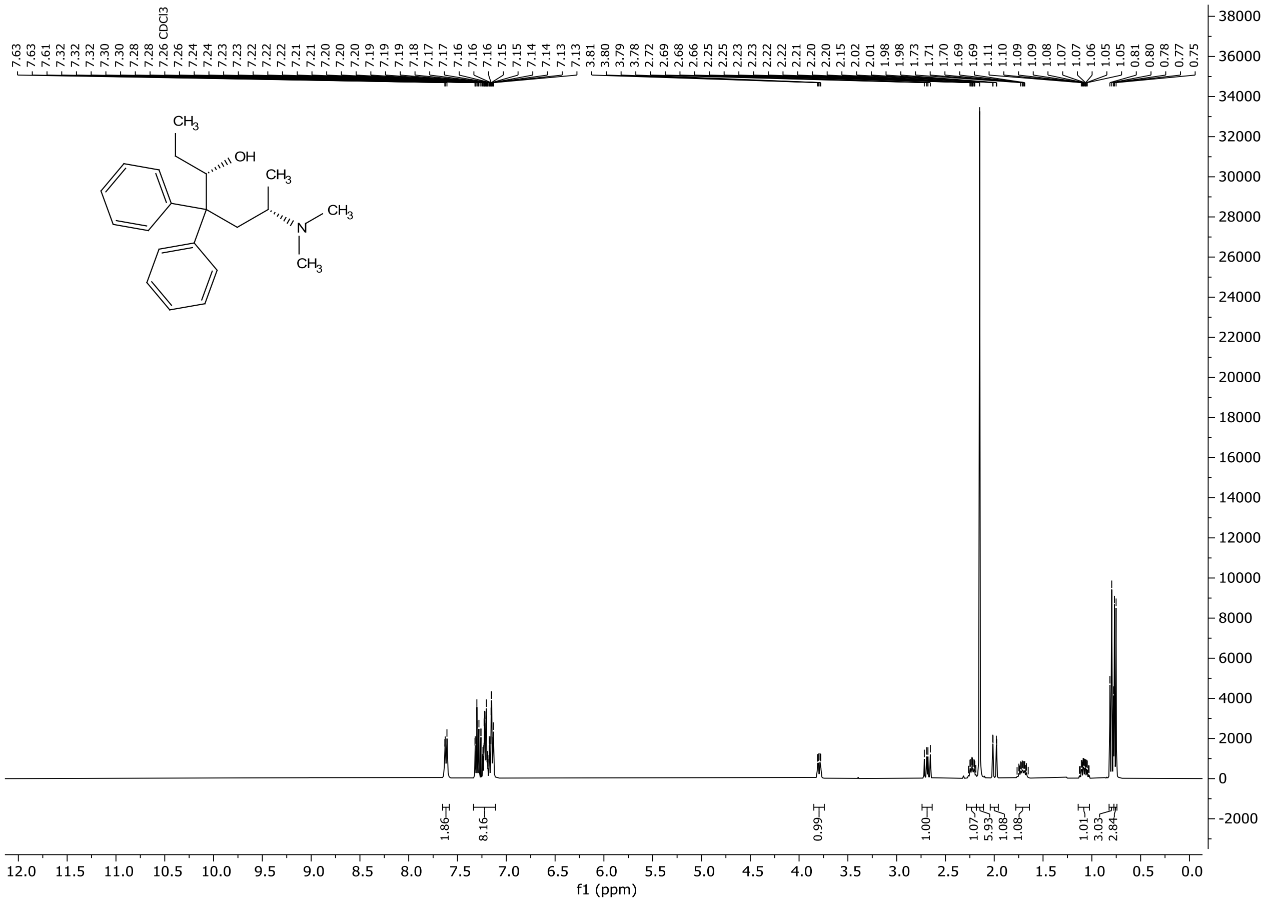

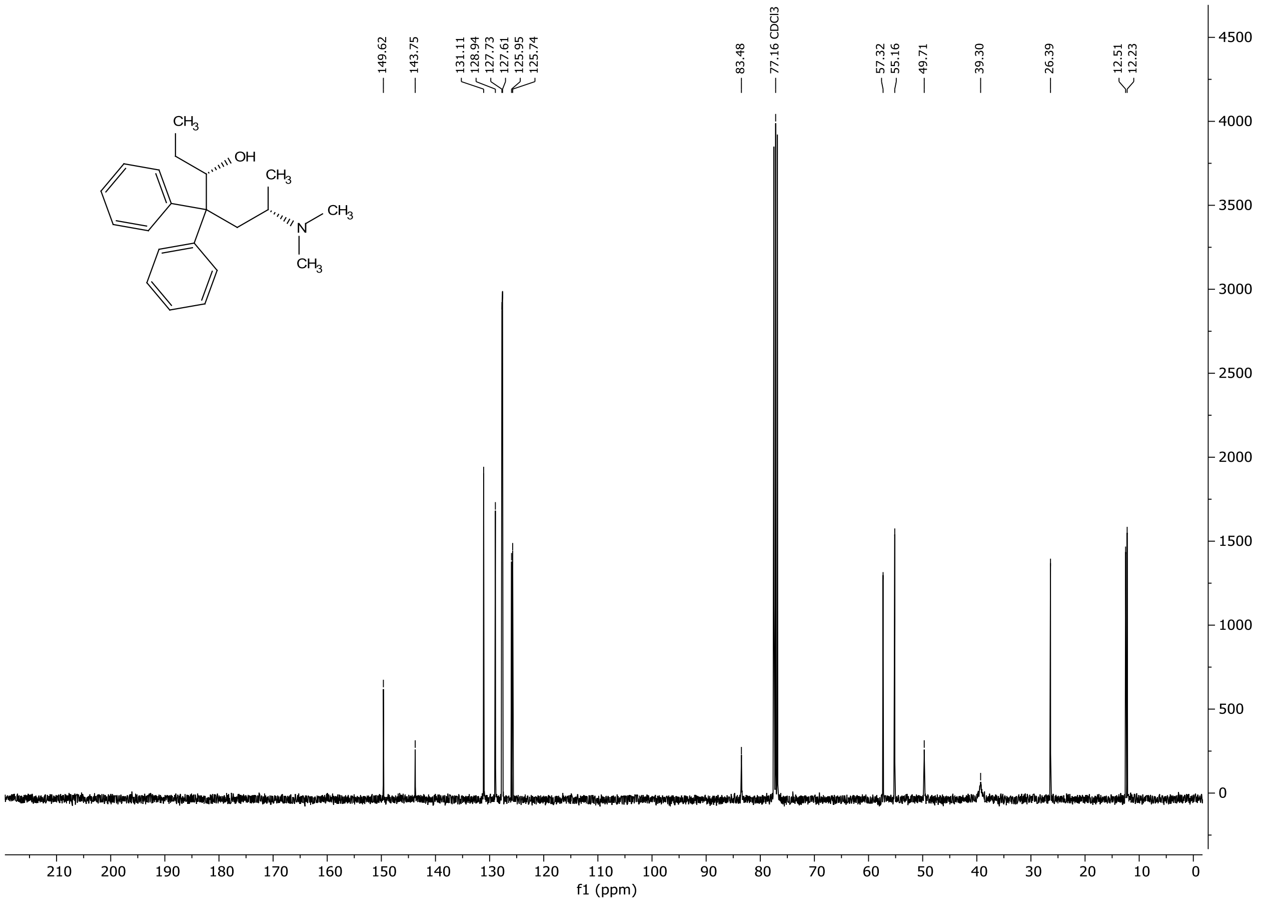

UPLC chromatogram of (3*S*,6*S*)-6-(dimethylamino)-4,4-diphenylheptan-3-ol ((3*S*,6*S*)-**ML**)

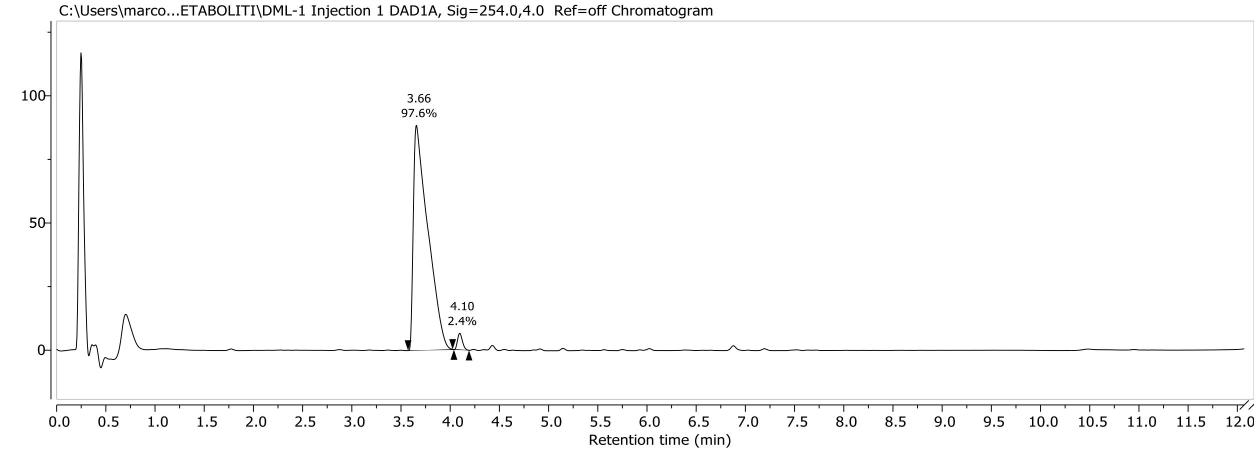

Chiral HPLC chromatogram of (3*S*,6*S*)-6-(dimethylamino)-4,4-diphenylheptan-3-ol ((3*S*,6*S*)-**ML**)

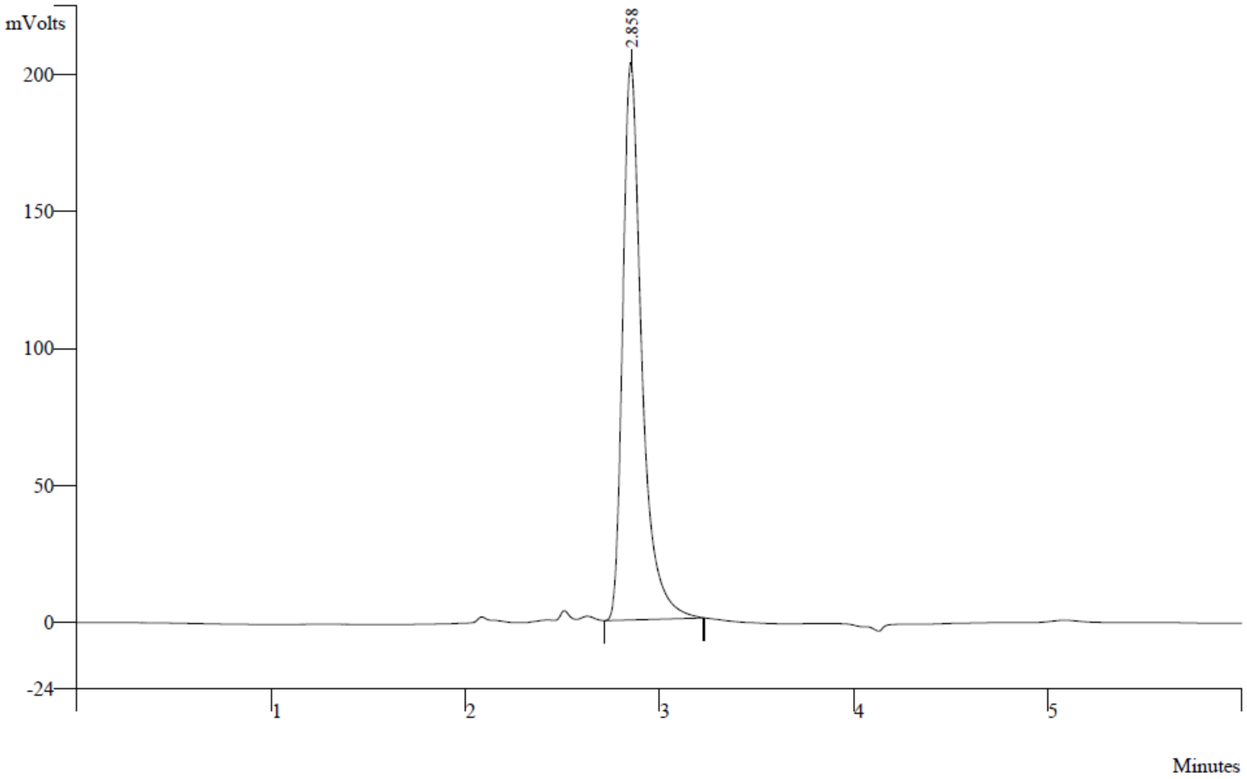

Peak No	Ret Time (min)	Peak Area (counts)	Result ()
1	2,858	1390248	100,00
		**1390248**	**100,00**

^{1}H and ^{13}C NMR spectra of (3*S*,6*R*)-6-(dimethylamino)-4,4-diphenylheptan-3-ol ((3*S*,6*R*)-**ML**)

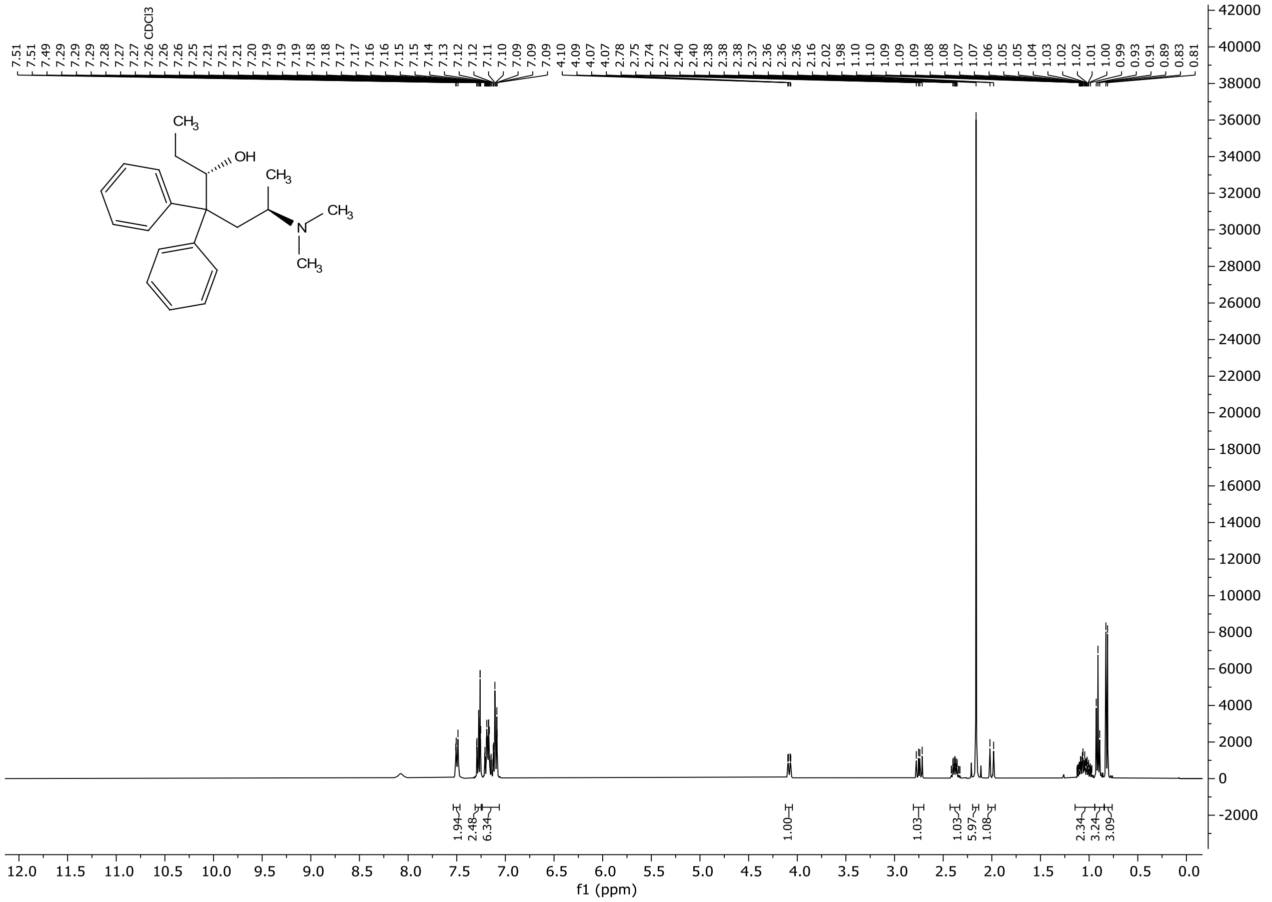

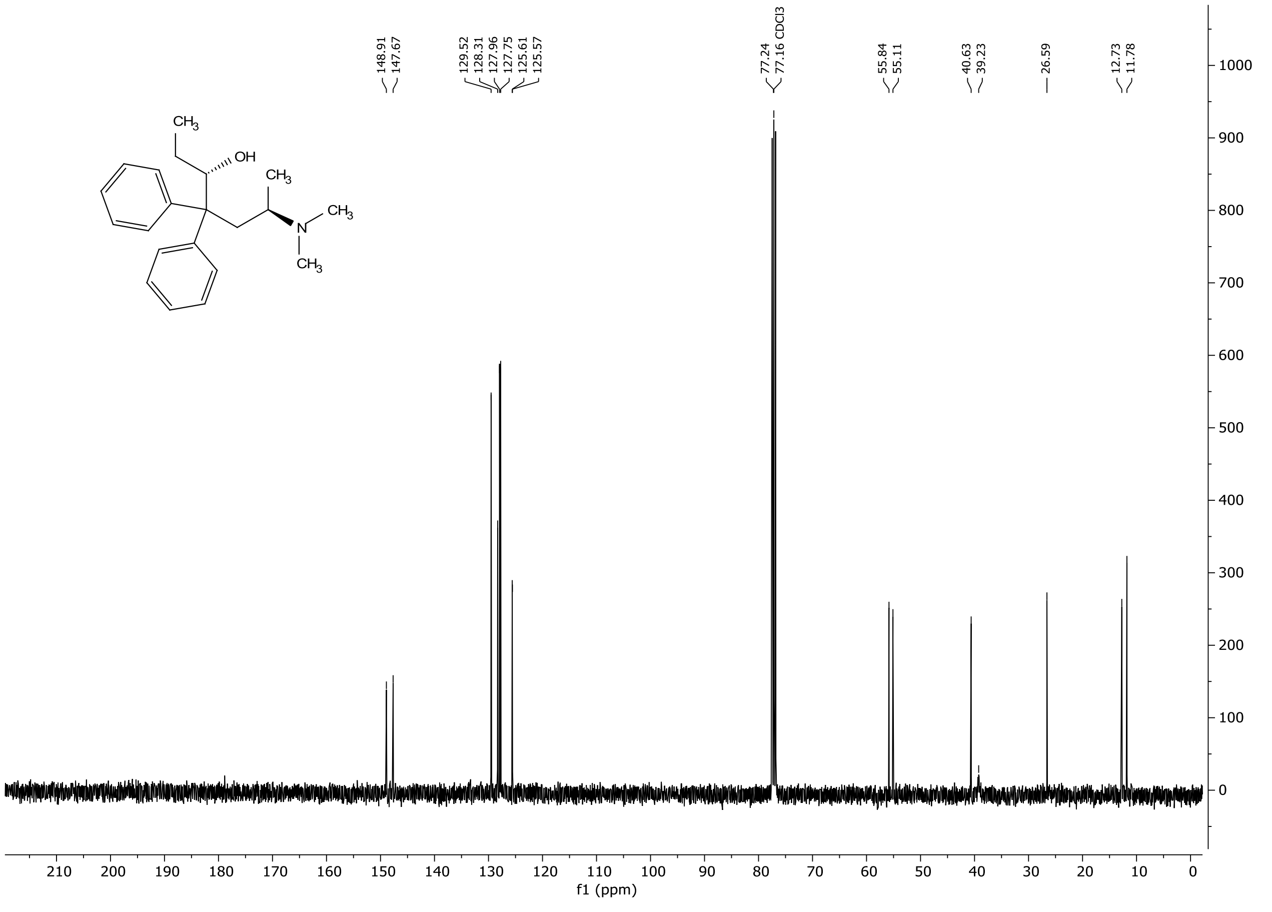

UPLC chromatogram of (3*S*,6*R*)-6-(dimethylamino)-4,4-diphenylheptan-3-ol ((3*S*,6*R*)-**ML**)

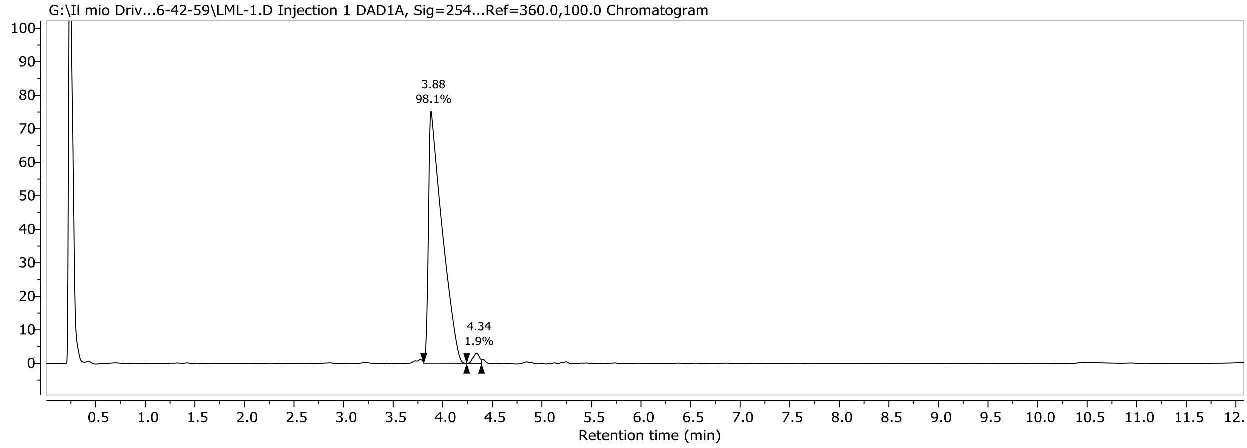

Chiral HPLC chromatogram of (3*S*,6*R*)-6-(dimethylamino)-4,4-diphenylheptan-3-ol ((3*S*,6*R*)-**ML**)

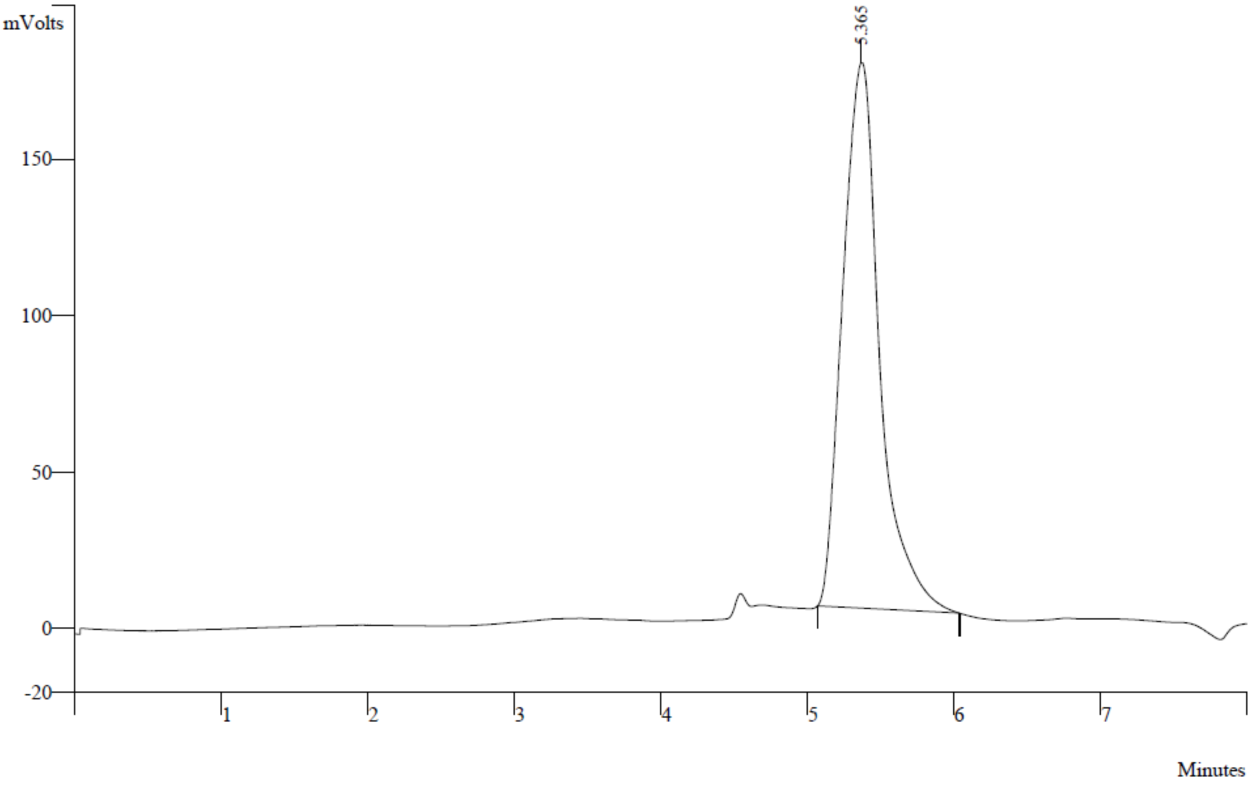

Peak No	Ret Time (min)	Peak Area (counts)	Result ()
1	5,365	3069777	100,00
		**3069777**	**100,00**

^1H and ^{13}C NMR spectra of (3*R*,6*S*)-6-(dimethylamino)-4,4-diphenylheptan-3-ol ((3*R*,6*S*)-**ML**)

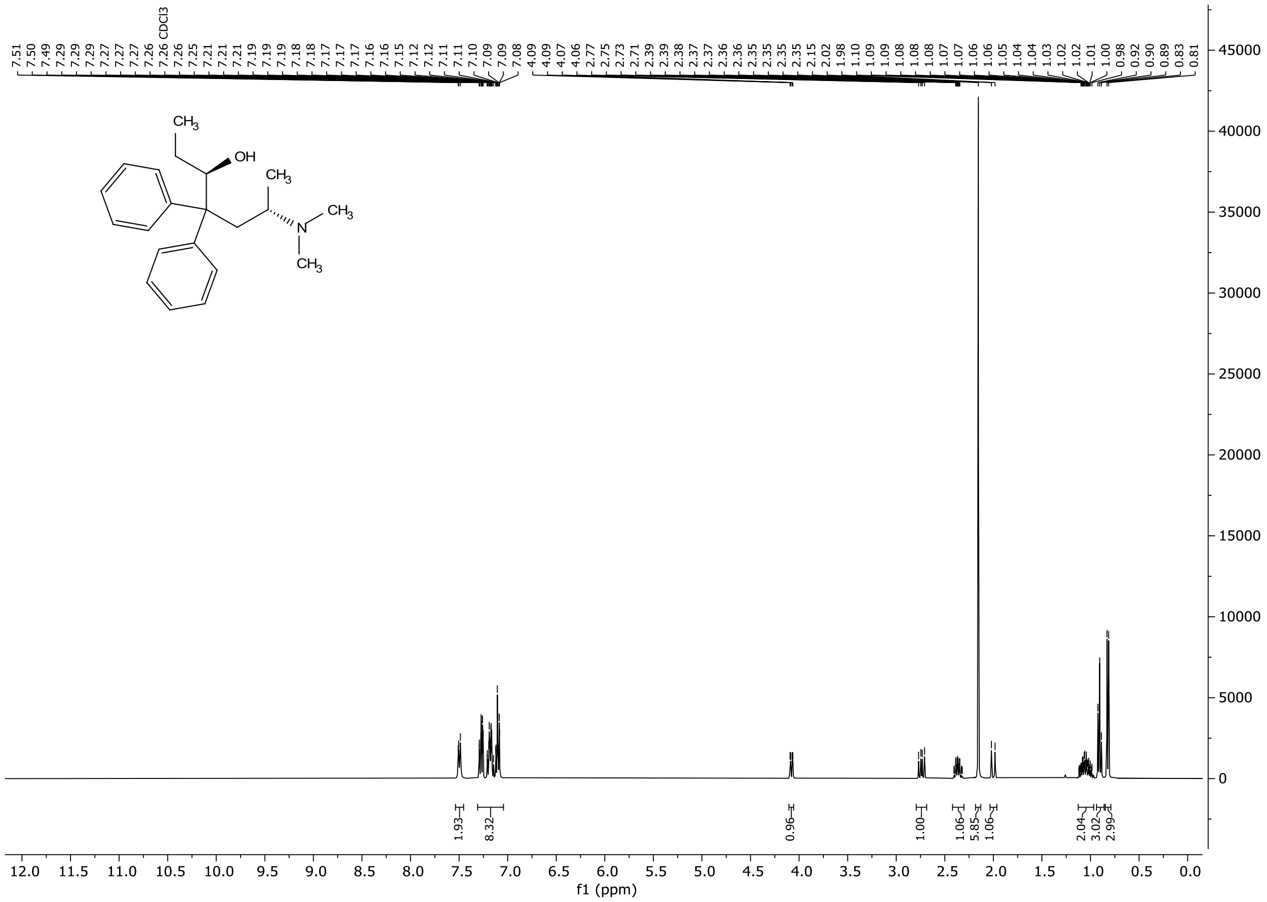

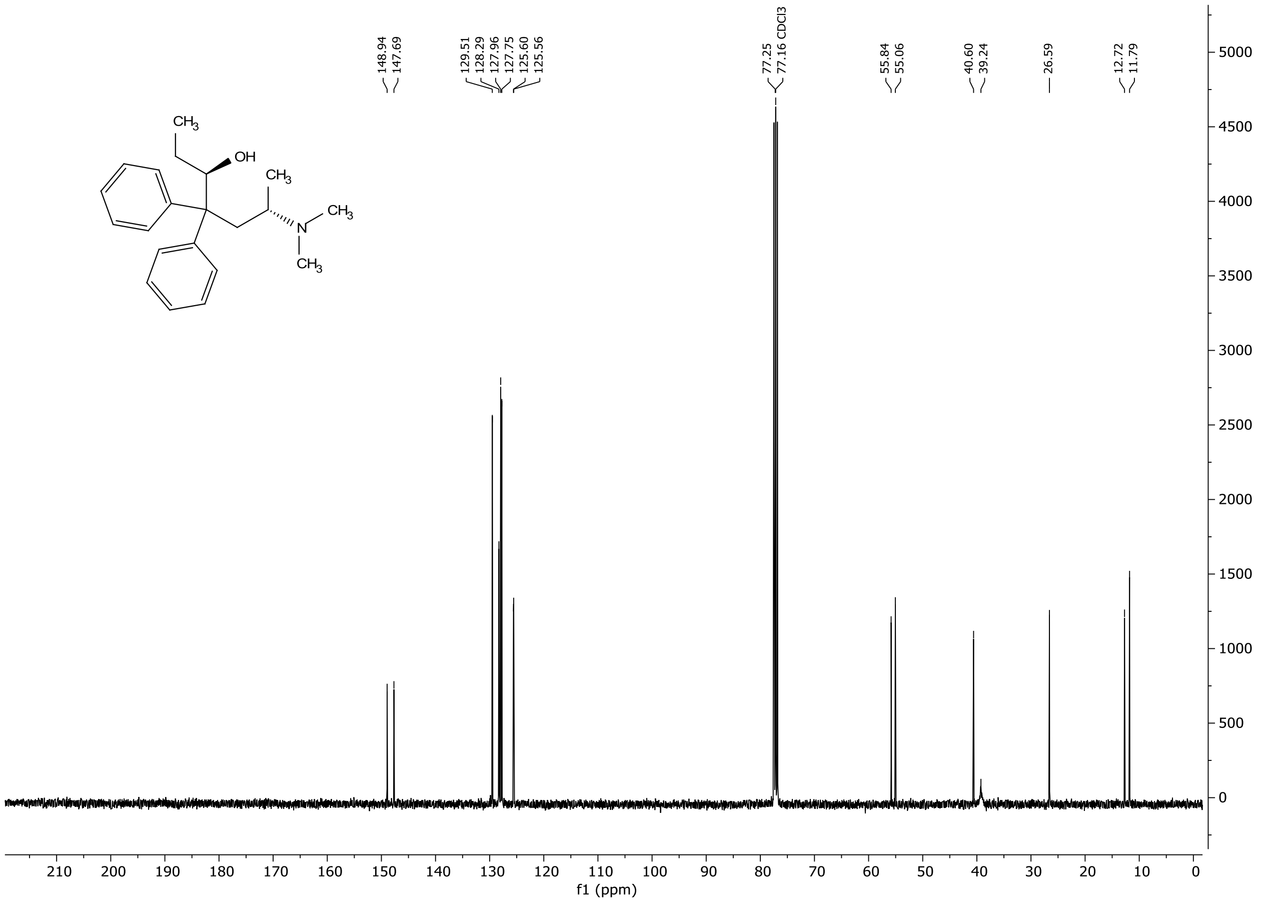

UPLC chromatogram of (3*R*,6*S*)-6-(dimethylamino)-4,4-diphenylheptan-3-ol ((3*R*,6*S*)-**ML**)

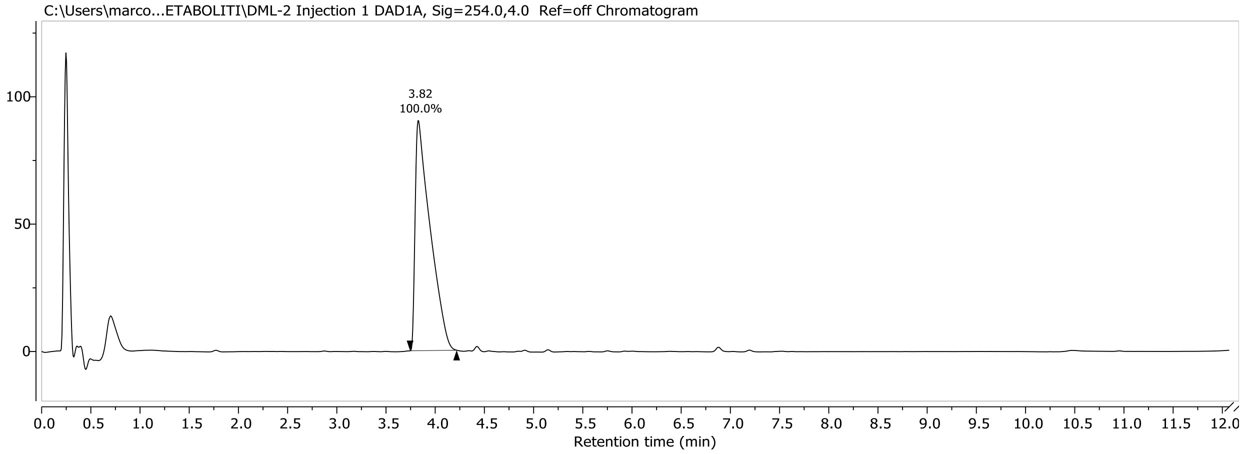

Chiral HPLC chromatogram of (3*R*,6*S*)-6-(dimethylamino)-4,4-diphenylheptan-3-ol ((3*R*,6*S*)-**ML**)

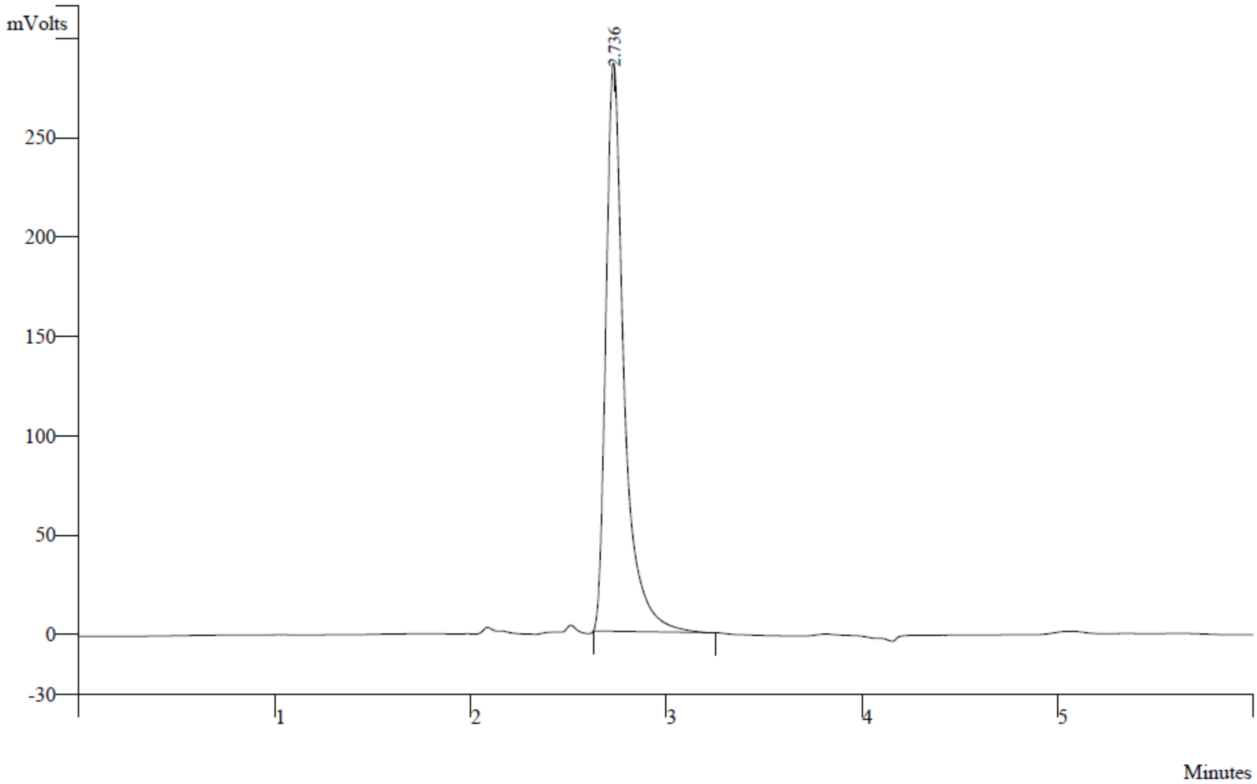

Peak No	Ret Time (min)	Peak Area (counts)	Result ()
1	2.736	1851669	100,00
		**1851669**	**100,00**

^{1}H and ^{13}C NMR spectra of (3*R*,6*R*)-6-amino-4,4-diphenylheptan-3-ol ((3*R*,6*R*)-**DNML**)

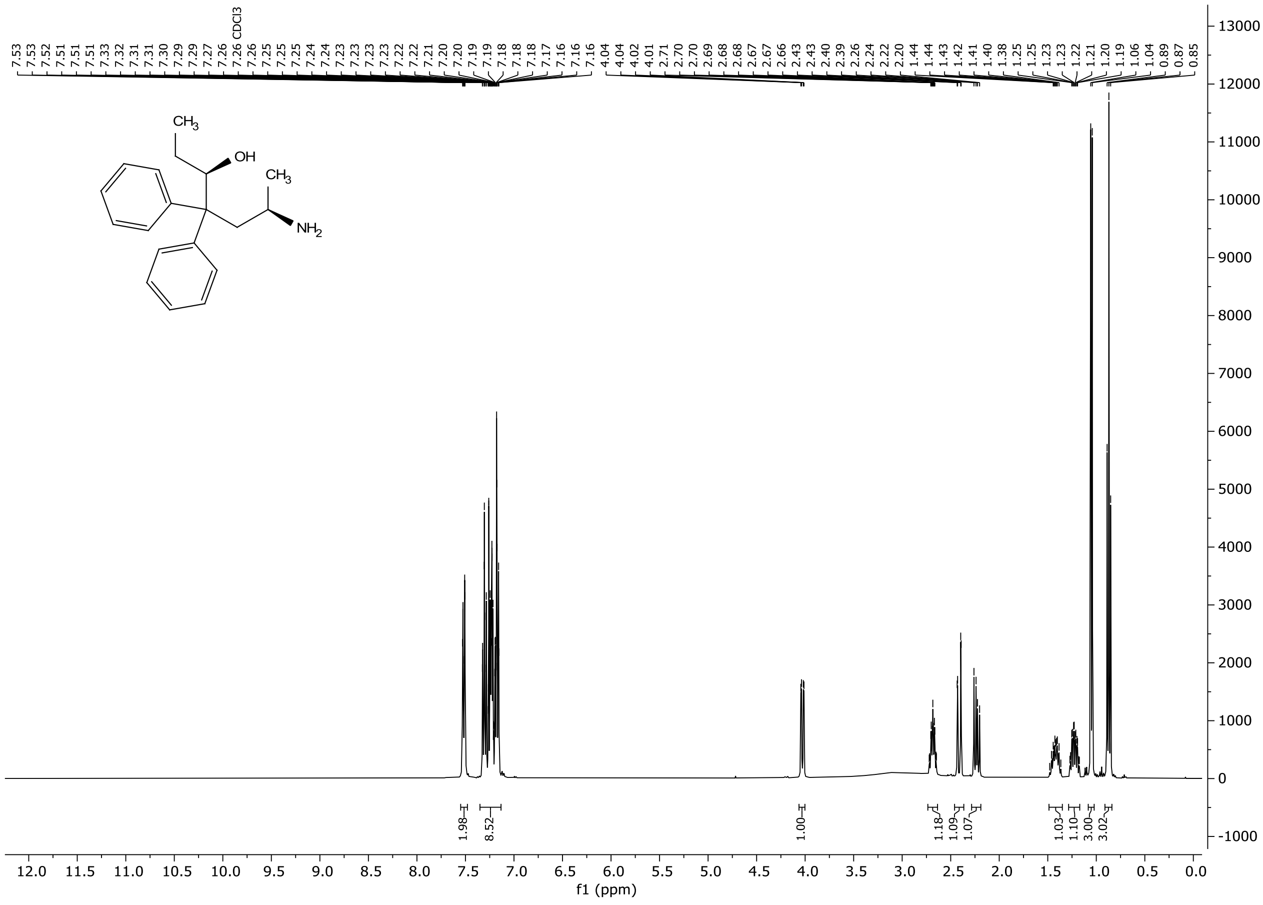

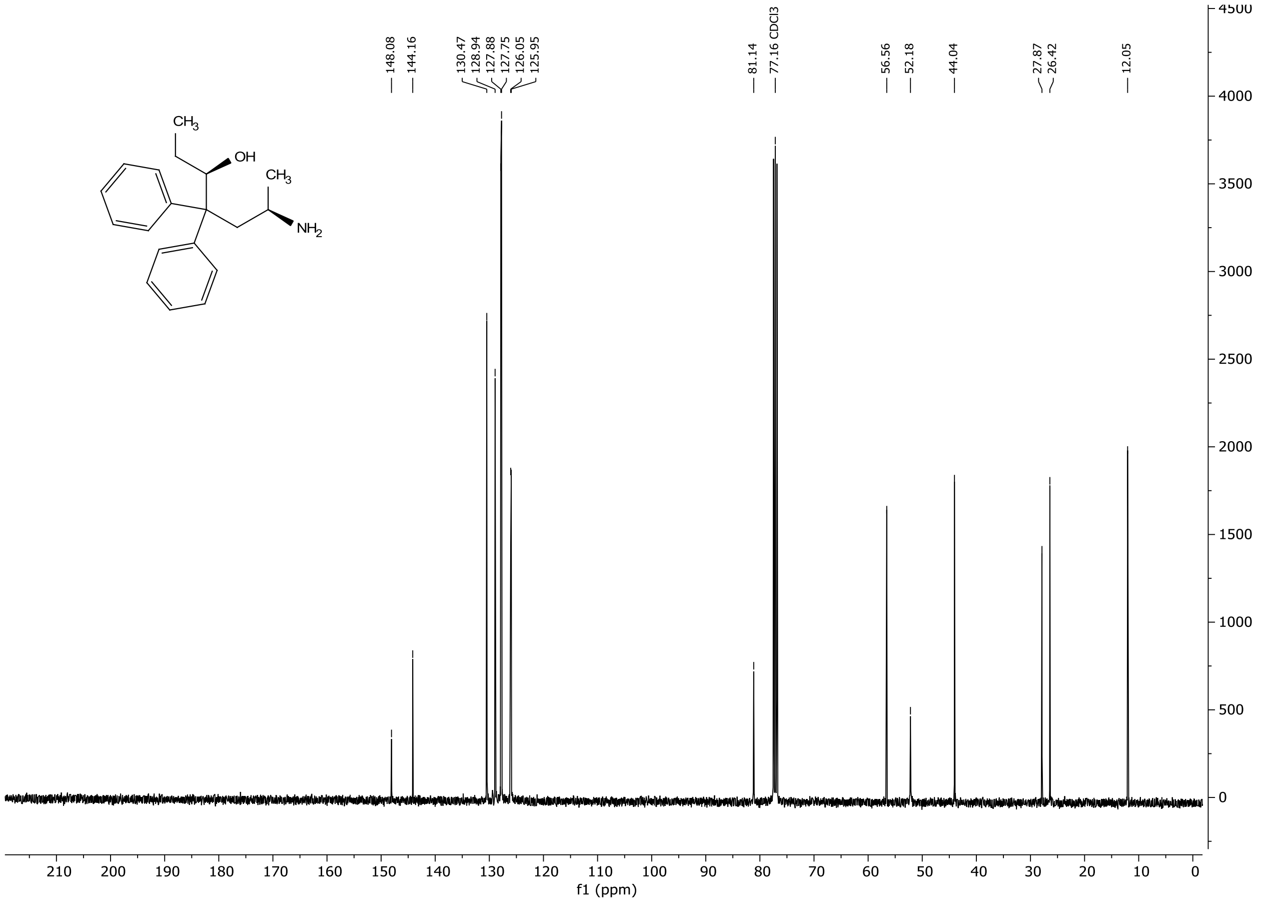

UPLC chromatogram of (3*R*,6*R*)-6-amino-4,4-diphenylheptan-3-ol ((3*R*,6*R*)-**DNML**)

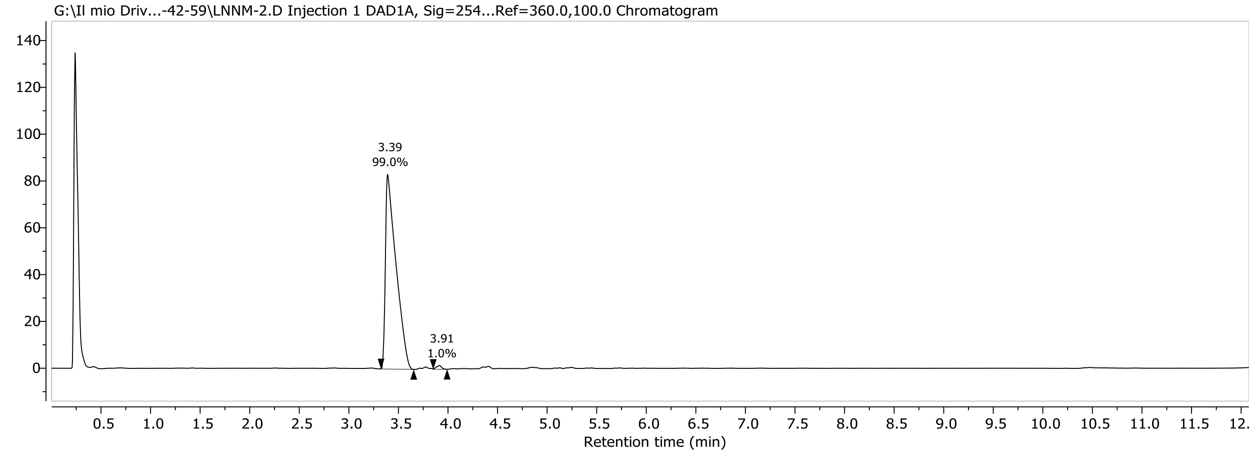

Chiral HPLC chromatogram of (3*R*,6*R*)-6-amino-4,4-diphenylheptan-3-ol ((3*R*,6*R*)-**DNML**)

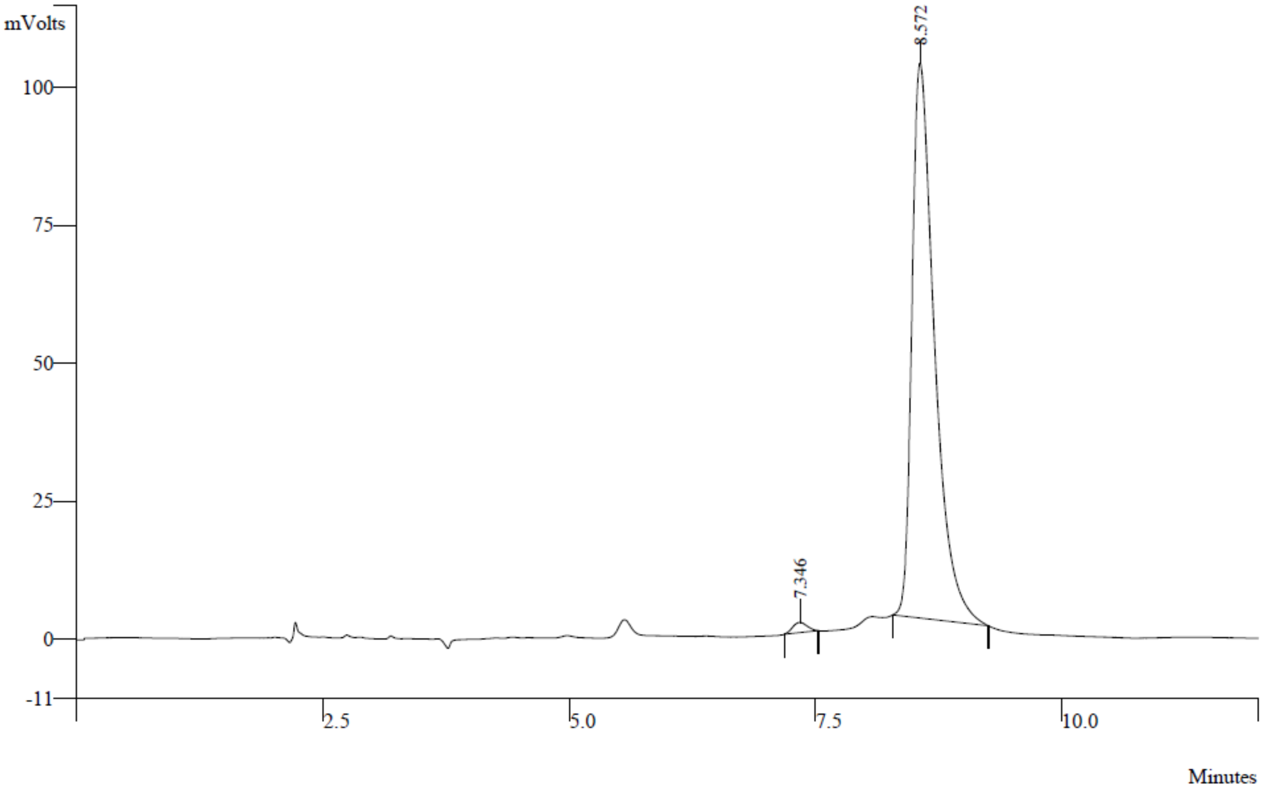

Peak No	Ret Time (min)	Peak Area (counts)	Result ()
1	7,346	19021	1,17
2	8,572	1611083	98,83
		**1630104**	**100,00**

^{1}H and ^{13}C NMR spectra of (3*S*,6*S*)-6-amino-4,4-diphenylheptan-3-ol ((3*S*,6*S*)-**DNML**)

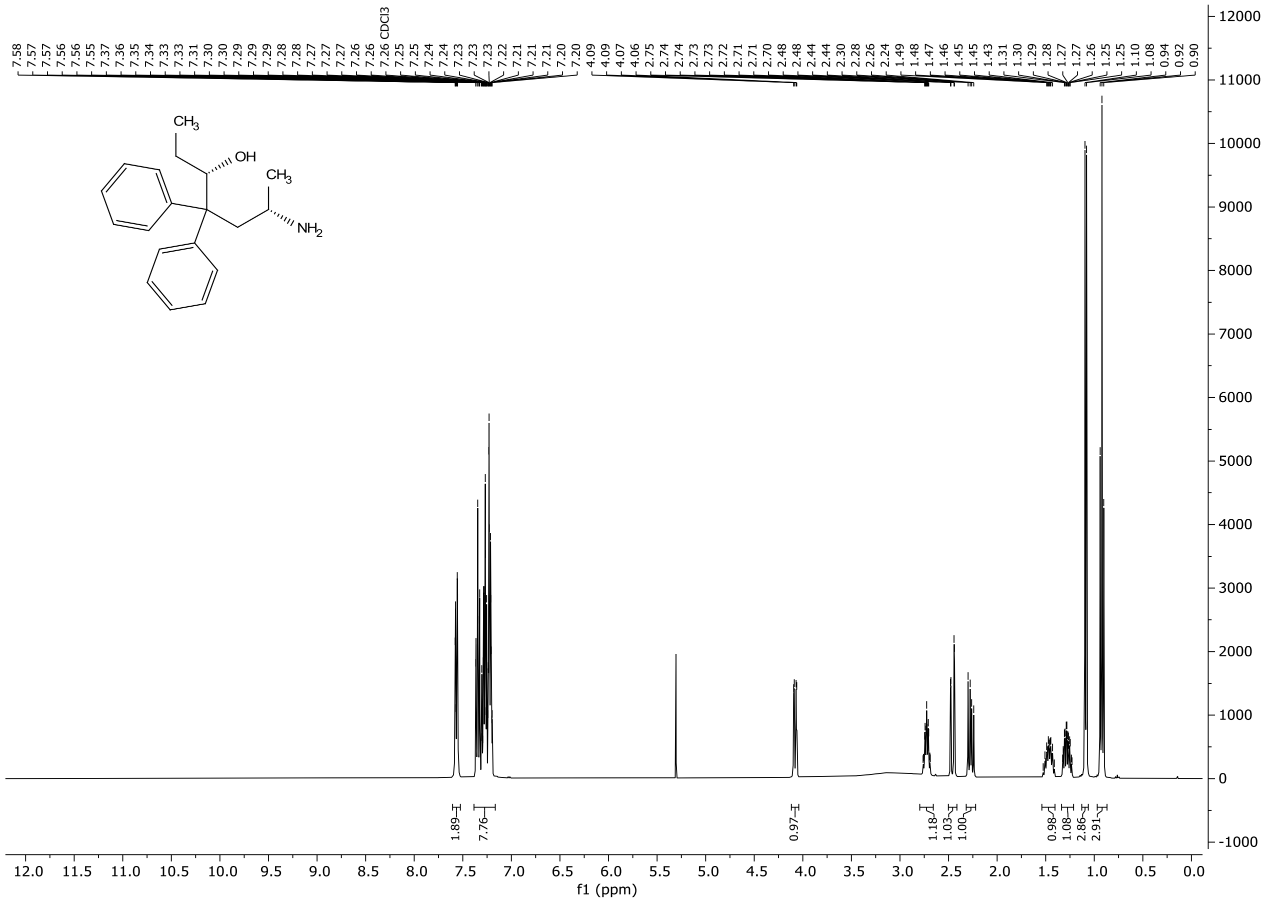

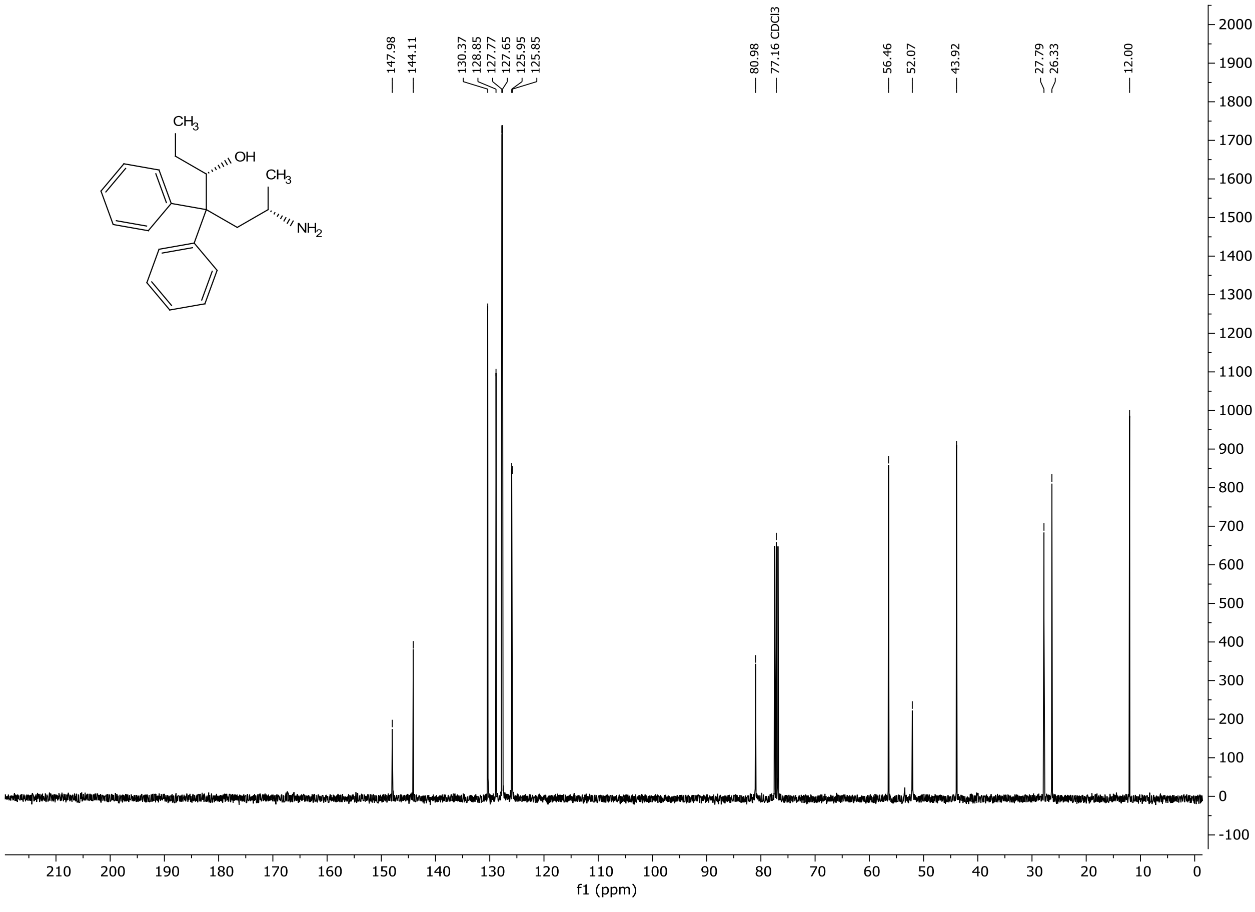

UPLC chromatogram of (3*S*,6*S*)-6-amino-4,4-diphenylheptan-3-ol ((3*S*,6*S*)-**DNML**)

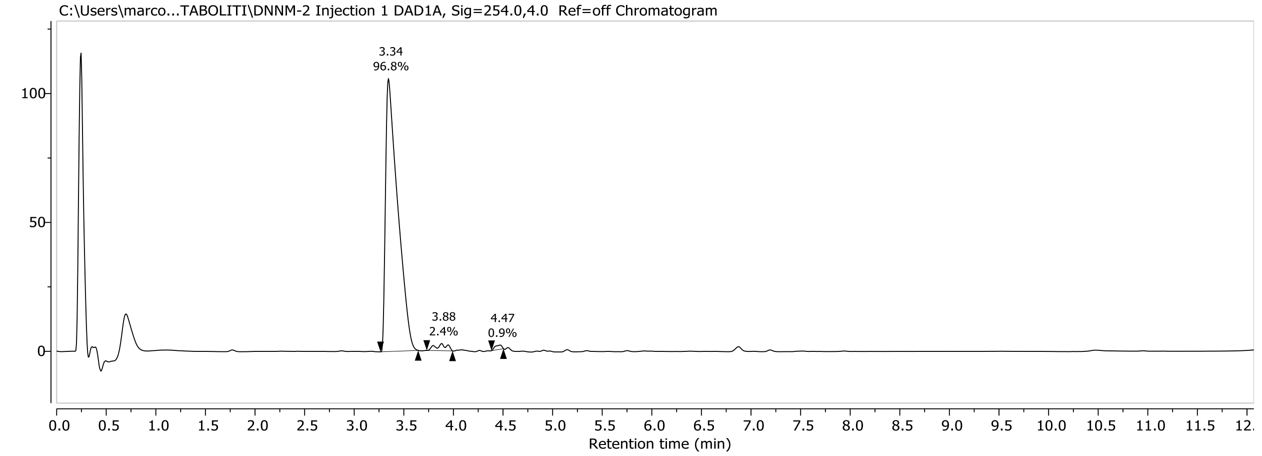

Chiral HPLC chromatogram of (3*S*,6*S*)-6-amino-4,4-diphenylheptan-3-ol ((3*S*,6*S*)-**DNML**)

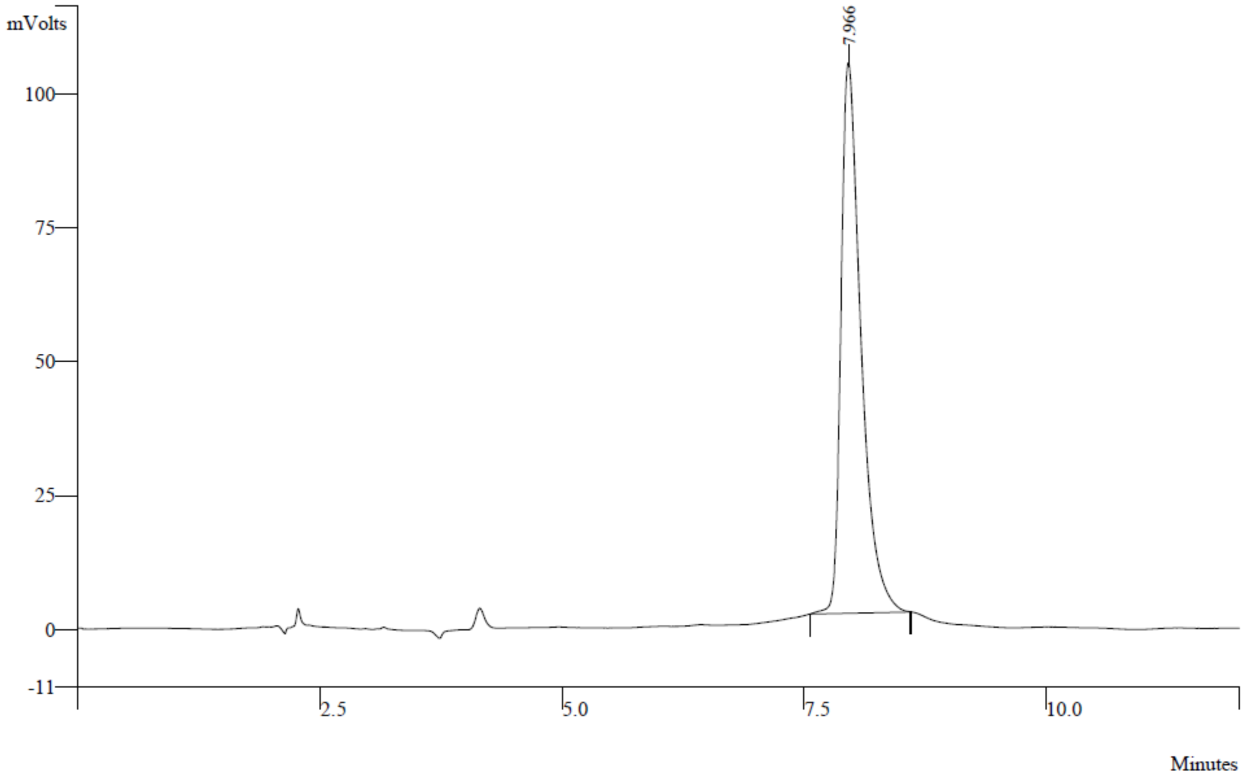

Peak No	Ret Time (min)	Peak Area (counts)	Result ()
1	7,966	1483410	100,00
		**1483410**	**100,00**

^{1}H and ^{13}C NMR spectra of (3*S*,6*R*)-6-amino-4,4-diphenylheptan-3-ol ((3*S*,6*R*)-**DNML**)

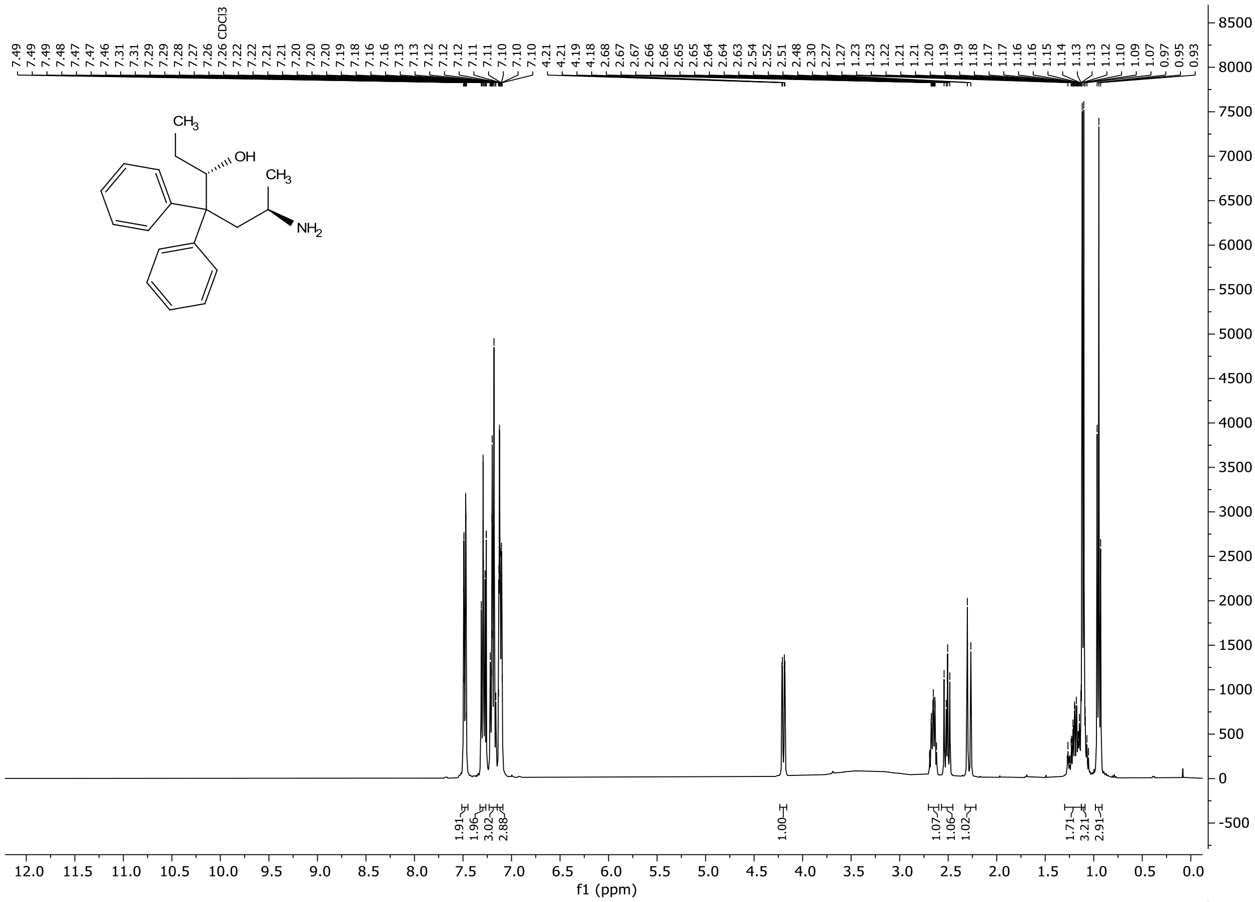

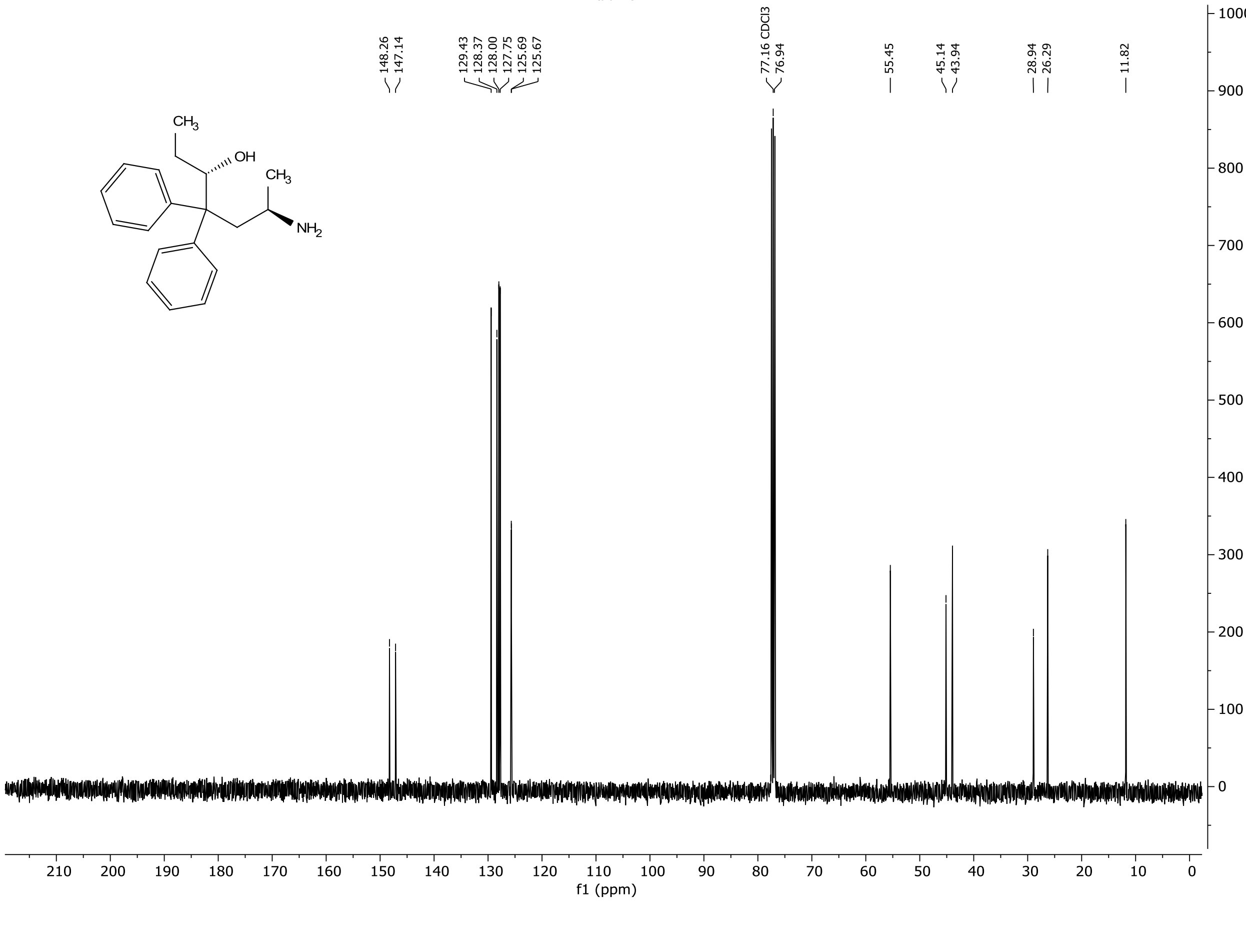

UPLC chromatogram of (3*S*,6*R*)-6-amino-4,4-diphenylheptan-3-ol ((3*S*,6*R*)-**DNML**)

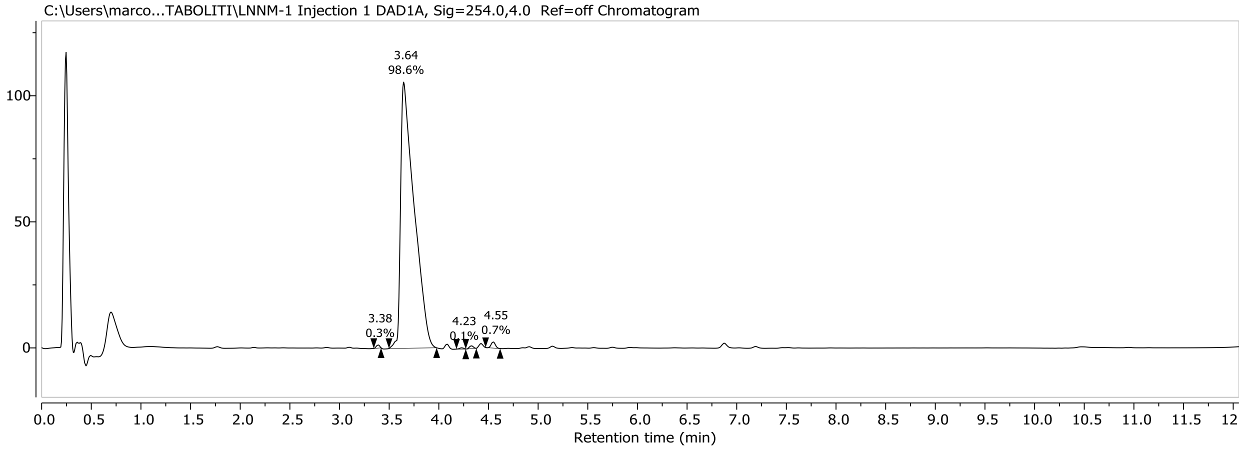

Chiral HPLC chromatogram of (3*S*,6*R*)-6-amino-4,4-diphenylheptan-3-ol ((3*S*,6*R*)-**DNML**)

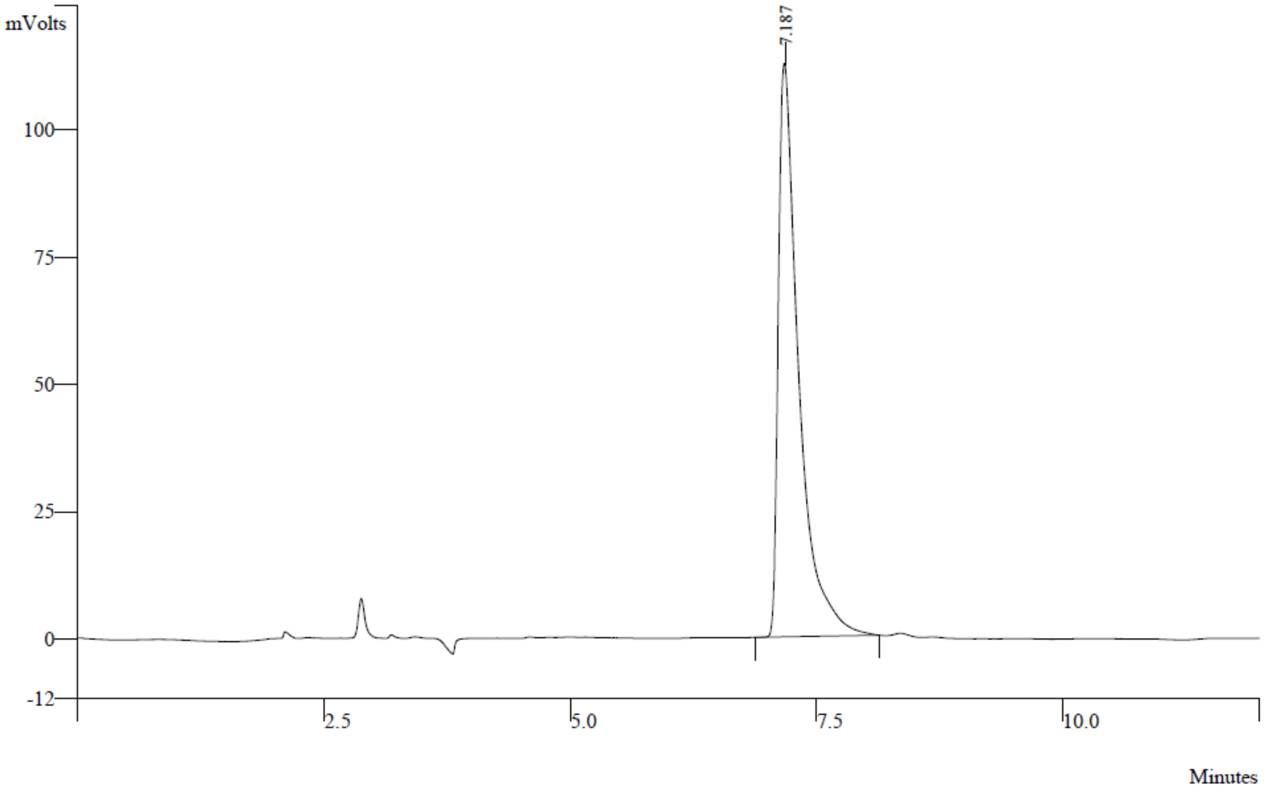

Peak No	Ret Time (min)	Peak Area (counts)	Result ()
1	7,187	1626923	100,00
		**1626923**	**100,00**

^{1}H and ^{13}C NMR spectra of (3*R*,6*S*)-6-amino-4,4-diphenylheptan-3-ol ((3*R*,6*S*)-**DNML**)

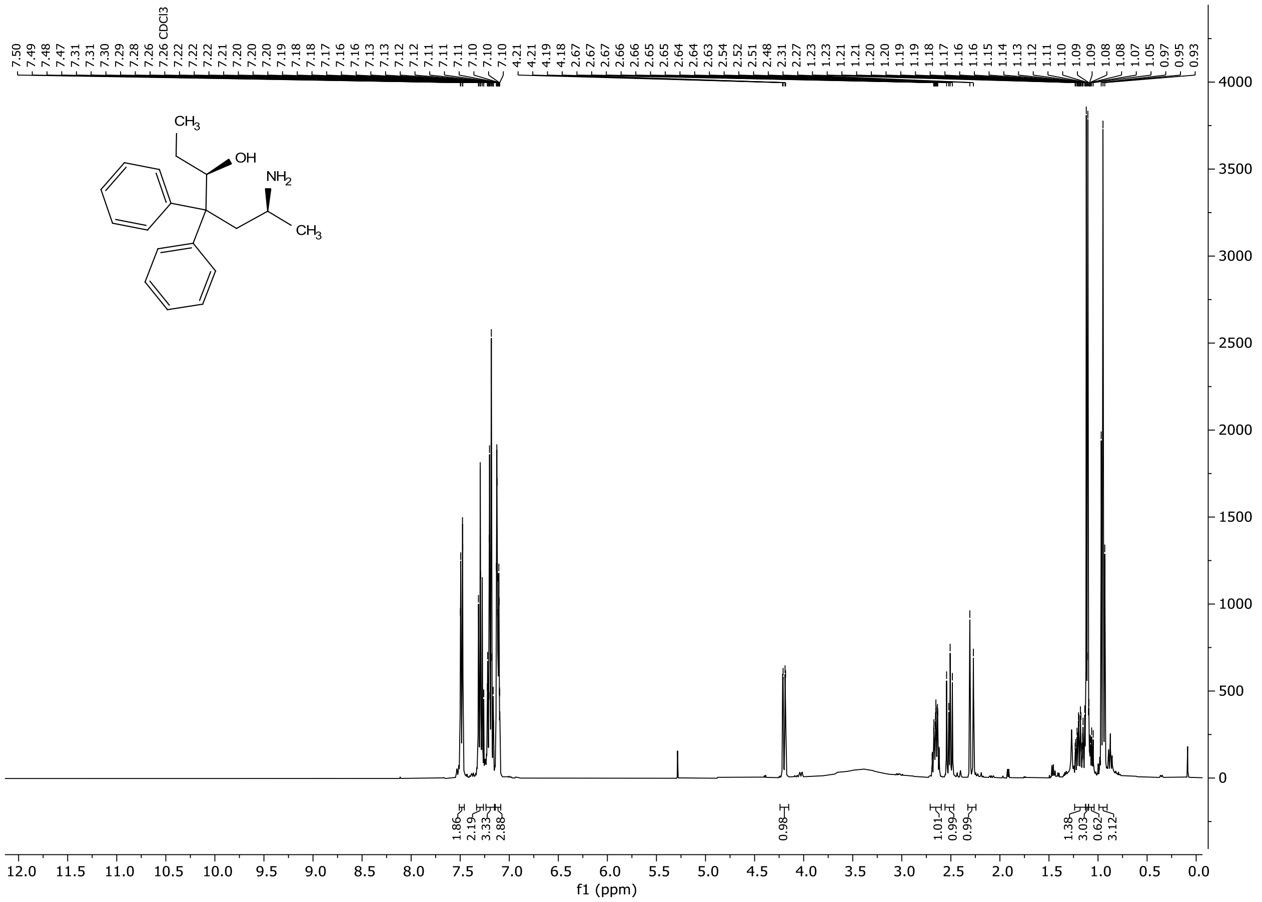

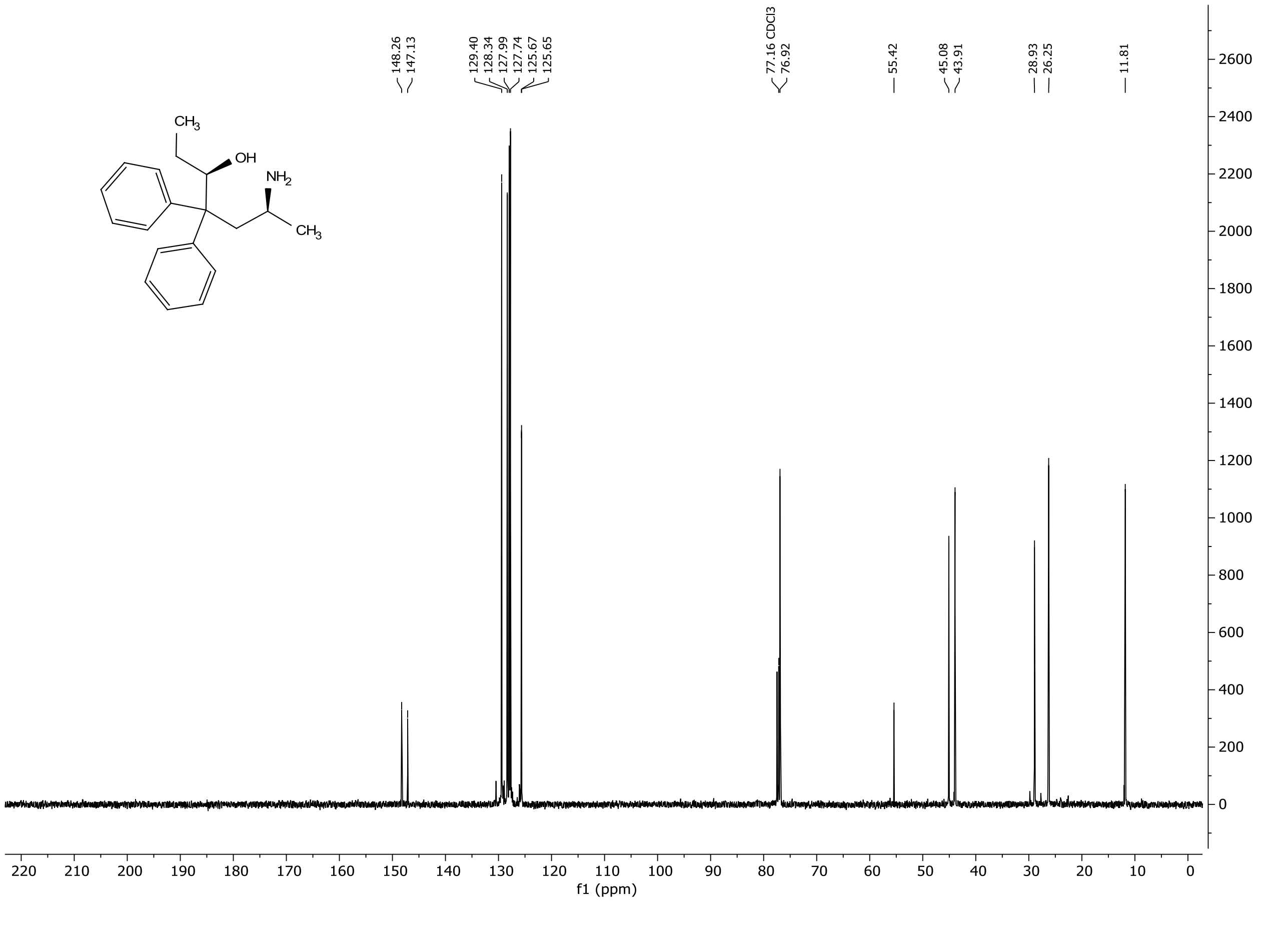

UPLC chromatogram of (3*R*,6*S*)-6-amino-4,4-diphenylheptan-3-ol ((3*R*,6*S*)-**DNML**)

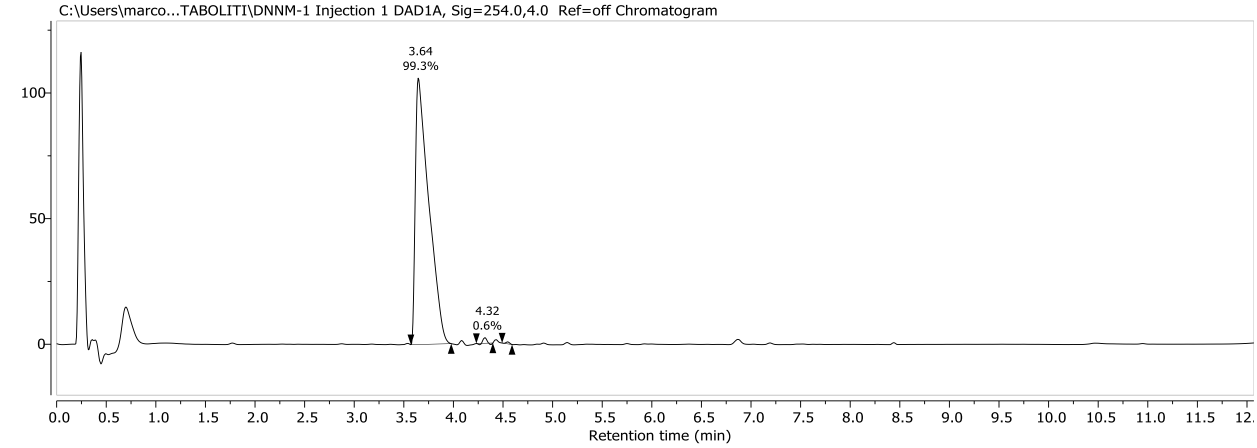

Chiral HPLC chromatogram of (3*R*,6*S*)-6-amino-4,4-diphenylheptan-3-ol ((3*R*,6*S*)-**DNML**)

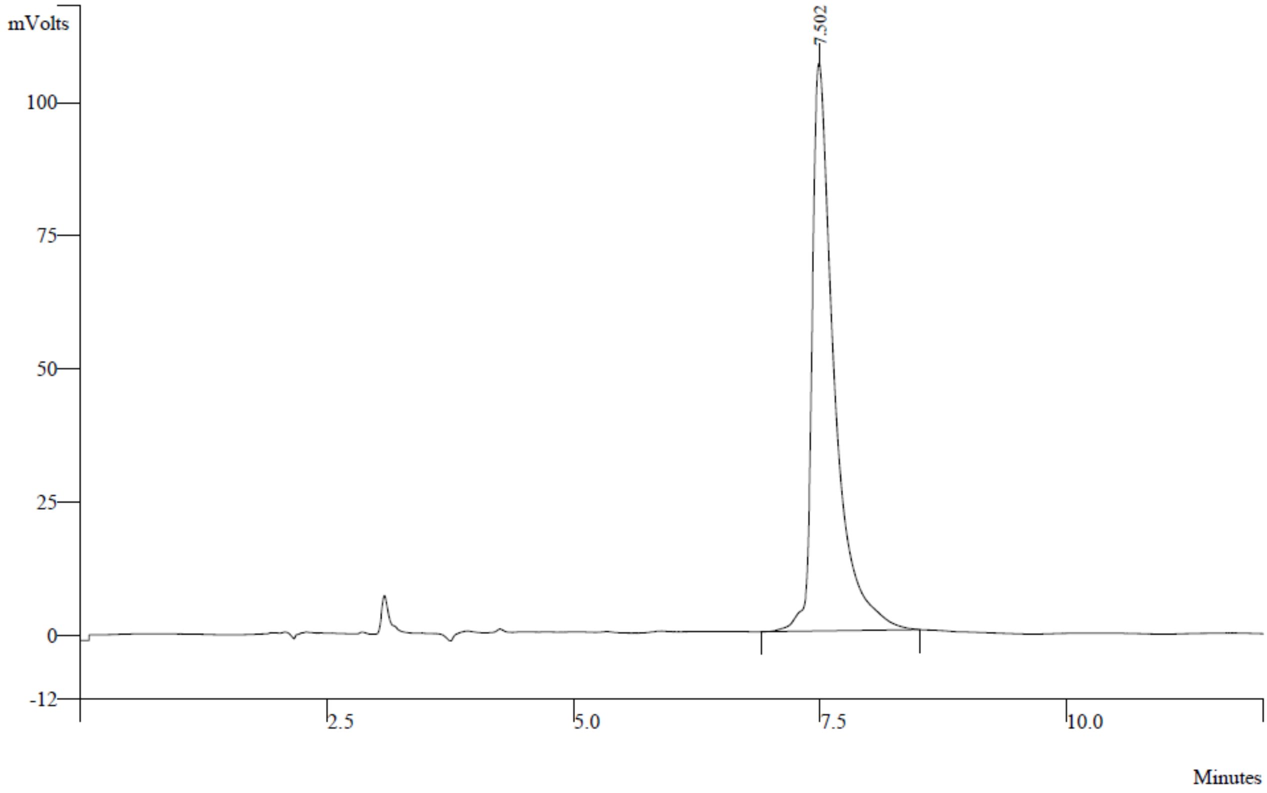

Peak No	Ret Time (min)	Peak Area (counts)	Result ()
1	7,502	1669193	100,00
		**1669193**	**100,00**

^{1}H and ^{13}C NMR spectra of (3*R*,6*R*)-6-(methylamino)-4,4-diphenylheptan-3-ol ((3*R*,6*R*)-**NML**)

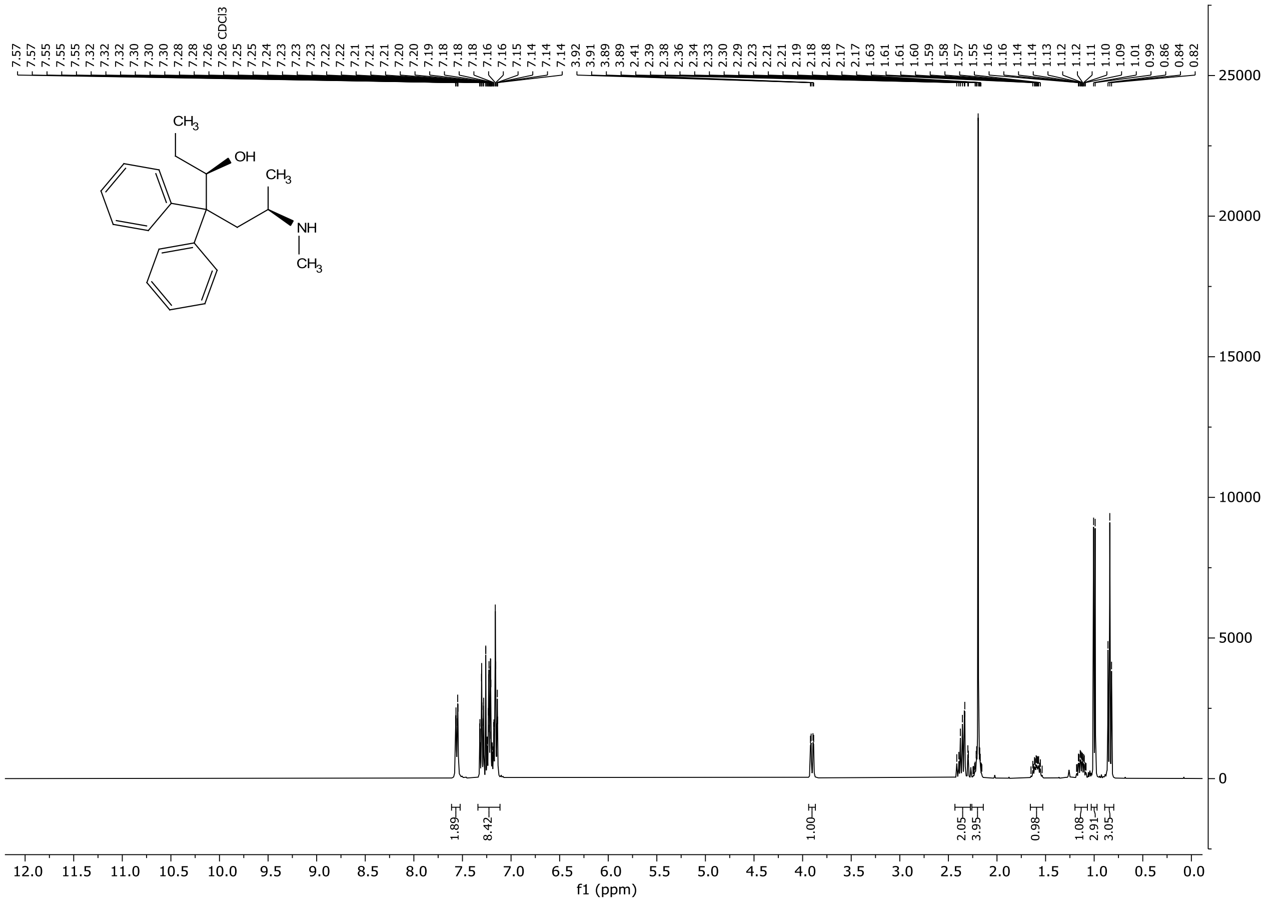

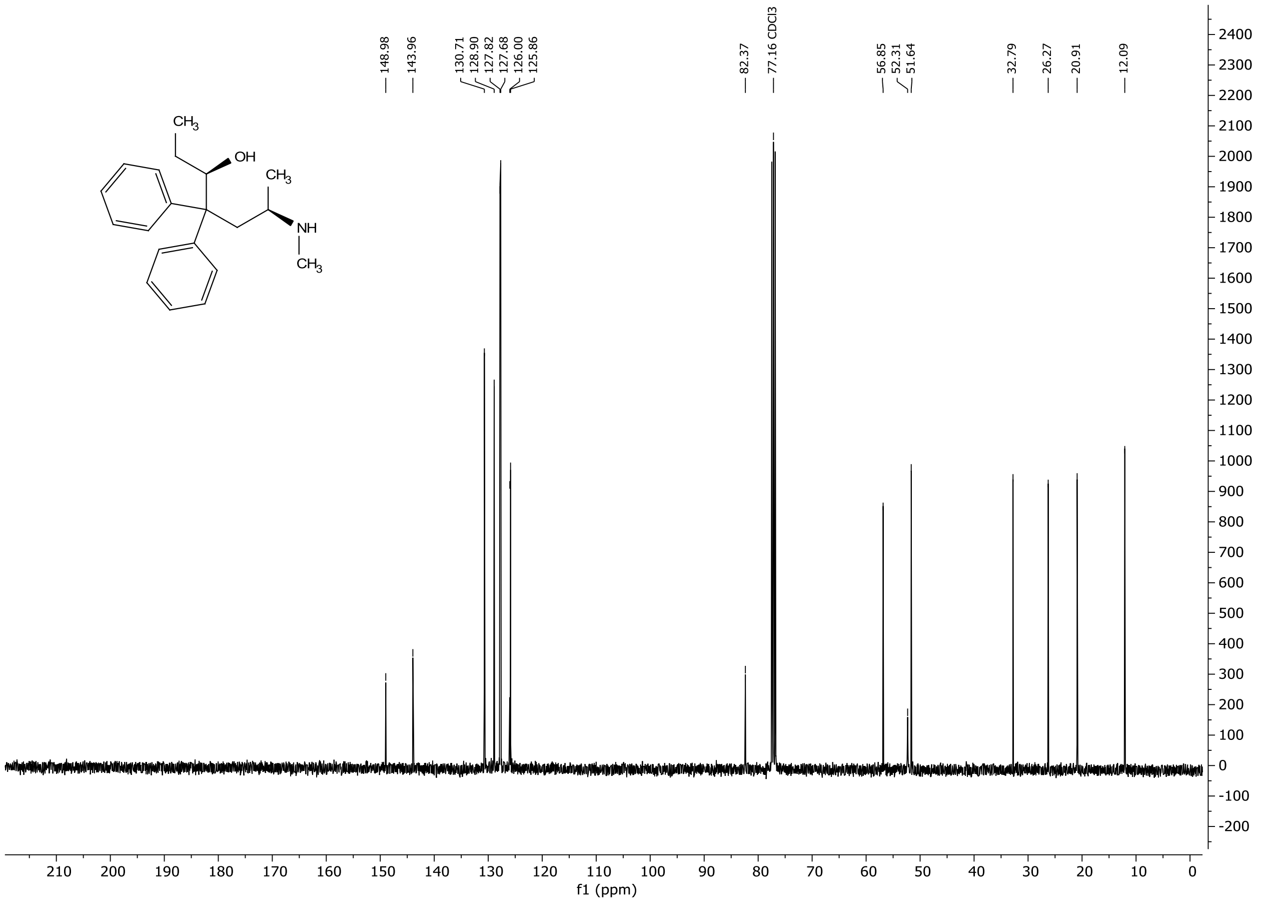

UPLC chromatogram of (3*R*,6*R*)-6-(methylamino)-4,4-diphenylheptan-3-ol ((3*R*,6*R*)-**NML**)

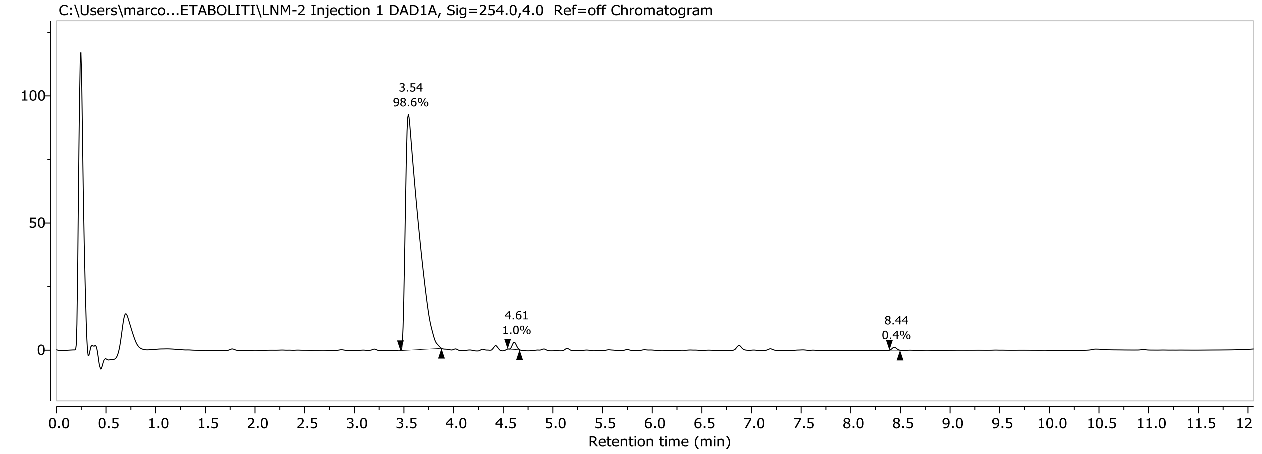

Chiral HPLC chromatogram of (3*R*,6*R*)-6-(methylamino)-4,4-diphenylheptan-3-ol ((3*R*,6*R*)-**NML**)

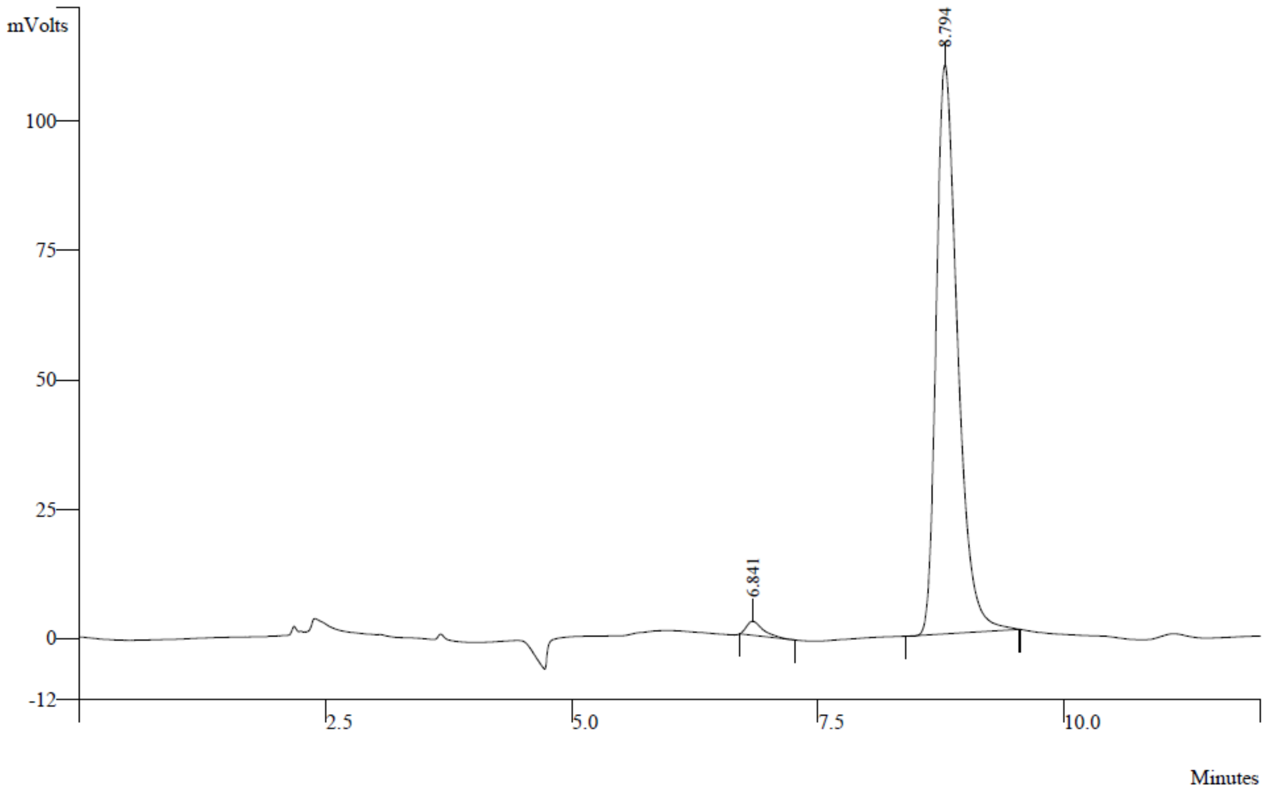

Peak No	Ret Time (min)	Peak Area (counts)	Result ()
1	6,841	31593	1,86
2	8,794	1669820	98,14
		**1701413**	**100,00**

^{1}H and ^{13}C NMR spectra of (3*S*,6*S*)-6-(methylamino)-4,4-diphenylheptan-3-ol ((3*S*,6*S*)-**NML**)

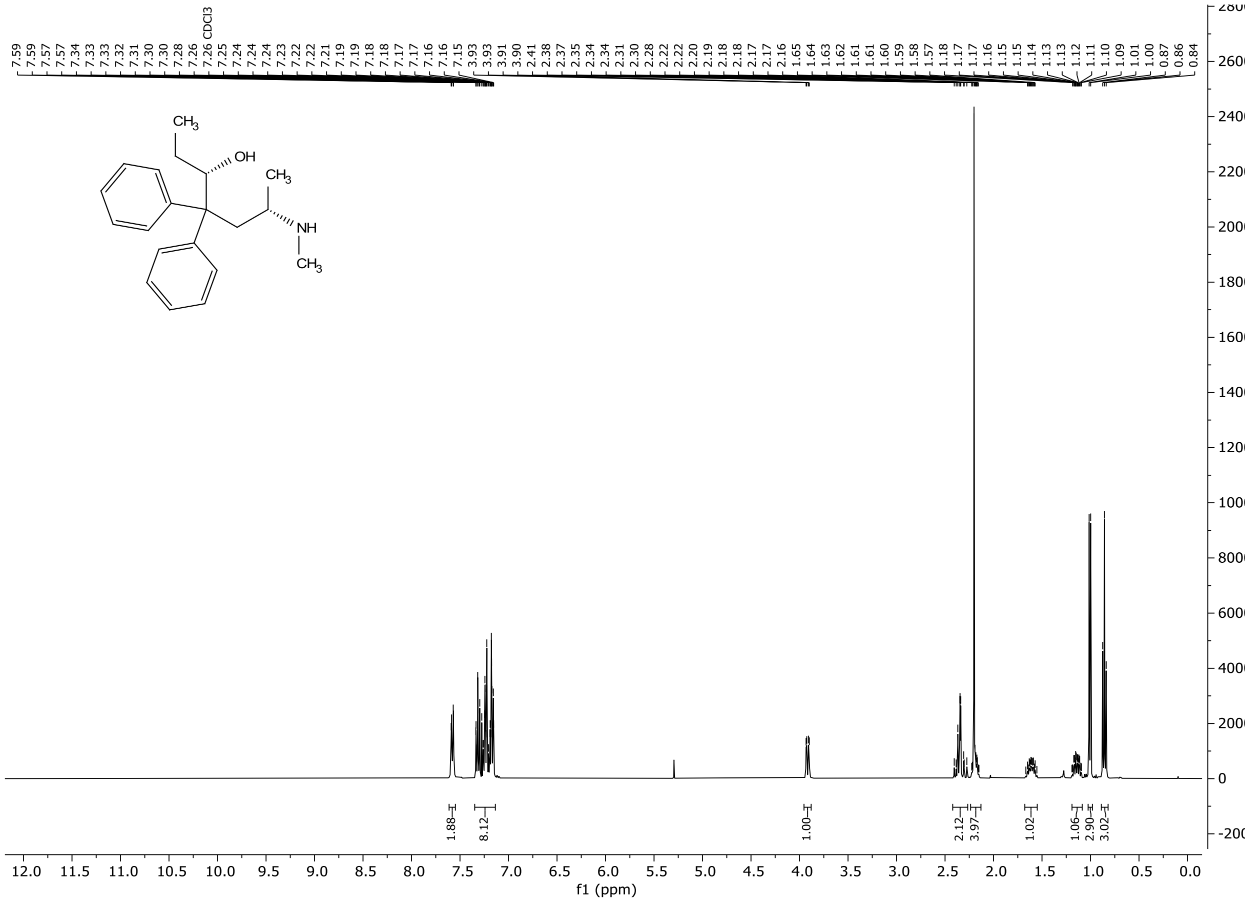

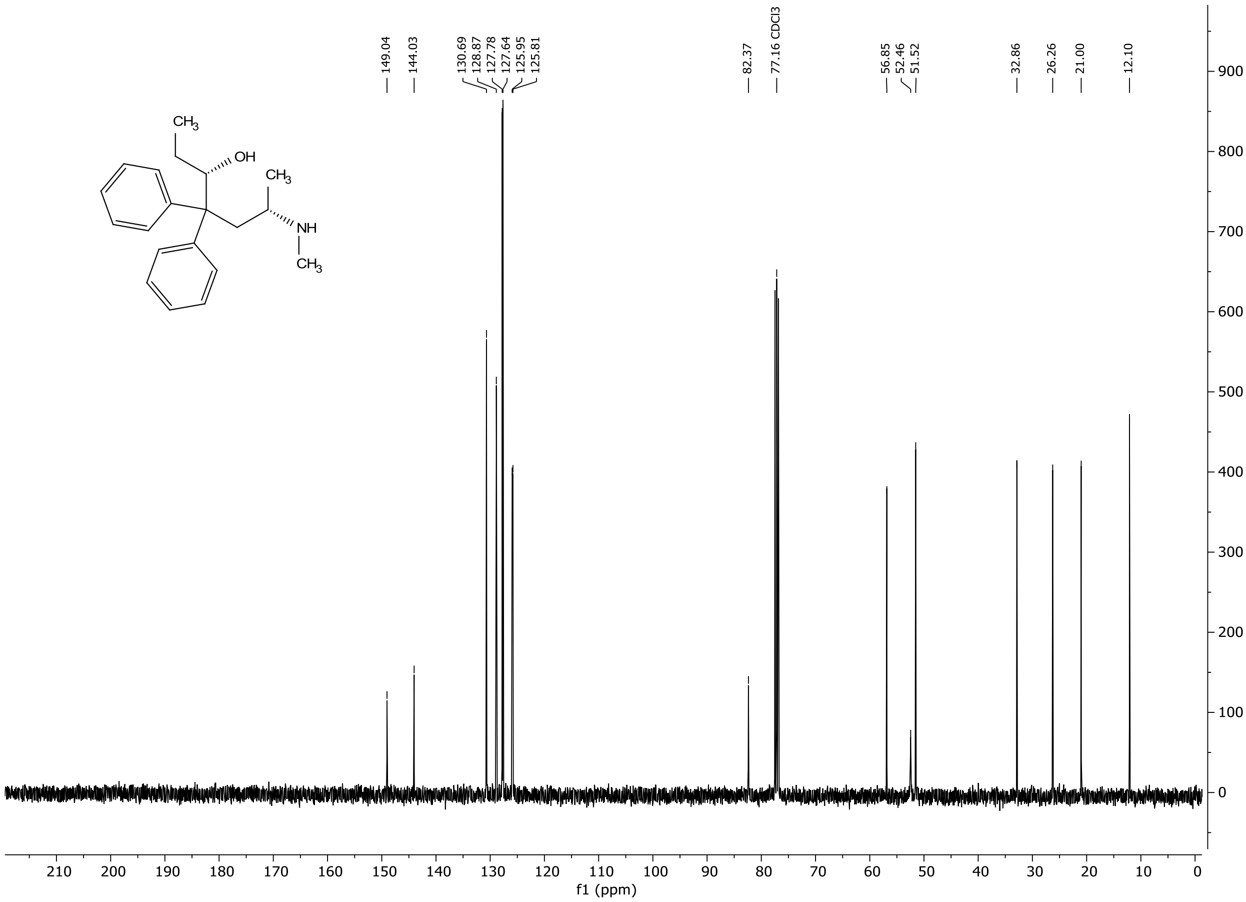

UPLC chromatogram of (3*S*,6*S*)-6-(methylamino)-4,4-diphenylheptan-3-ol ((3*S*,6*S*)-**NML**)

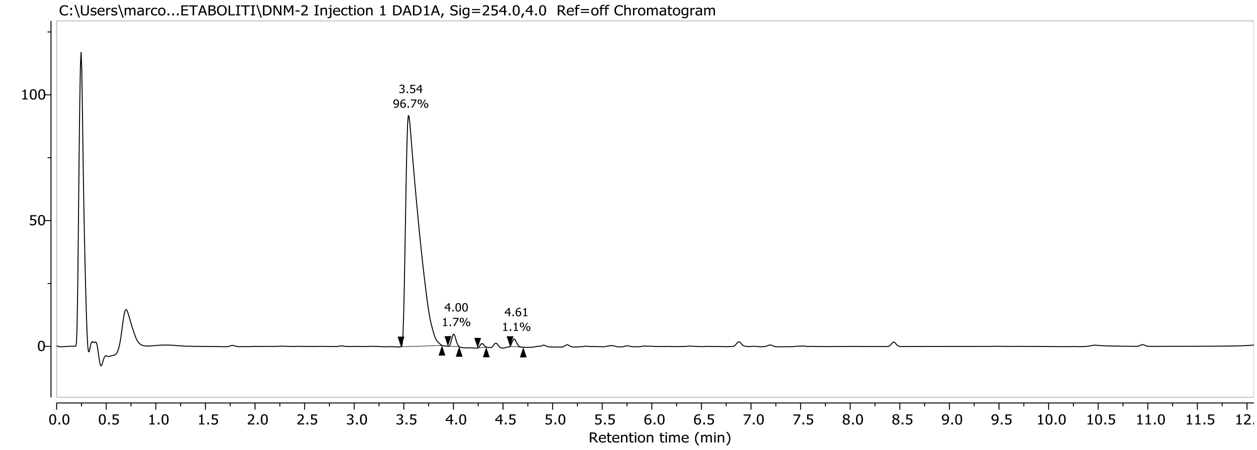

Chiral HPLC chromatogram of (3*S*,6*S*)-6-(methylamino)-4,4-diphenylheptan-3-ol ((3*S*,6*S*)-**NML**)

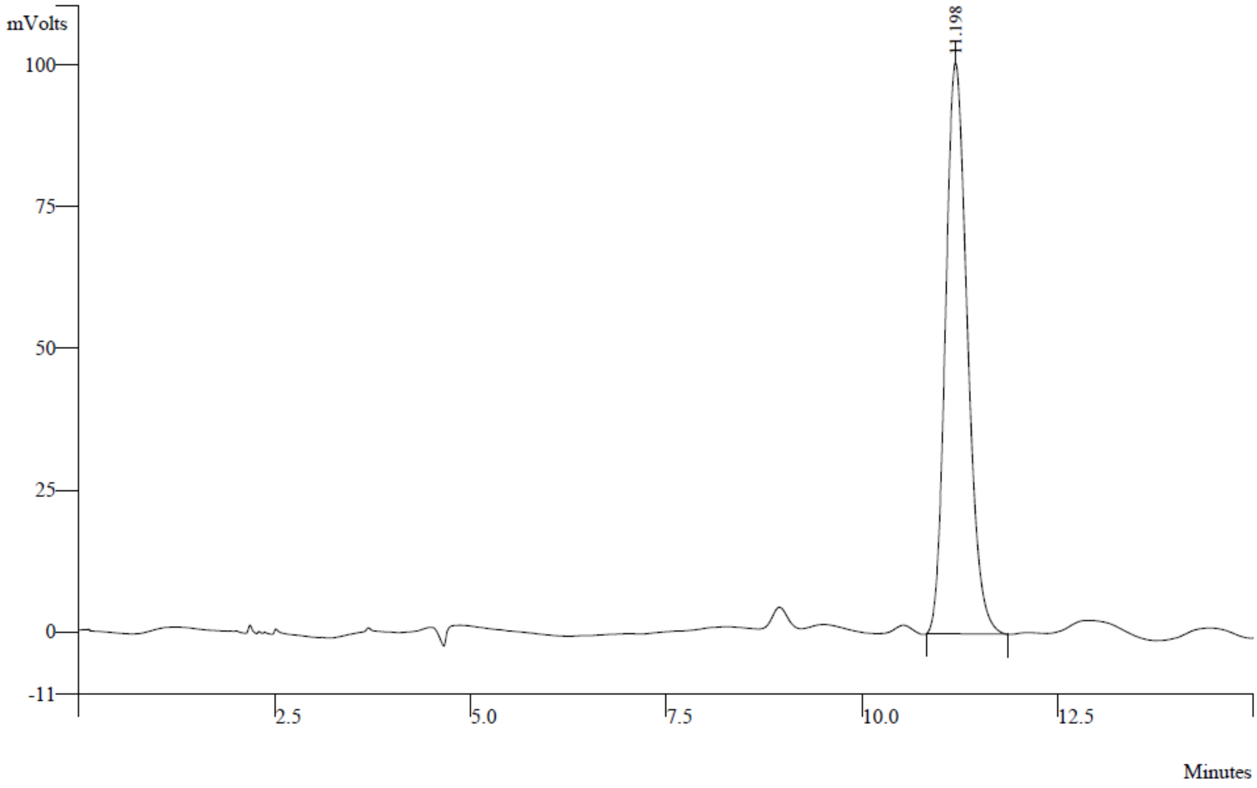

Peak No	Ret Time (min)	Peak Area (counts)	Result ()
1	11,198	1958368	100,00
		**1958368**	**100,00**

^{1}H and ^{13}C NMR spectra of (3*S*,6*R*)-6-(methylamino)-4,4-diphenylheptan-3-ol ((3*S*,6*R*)-**NML**)

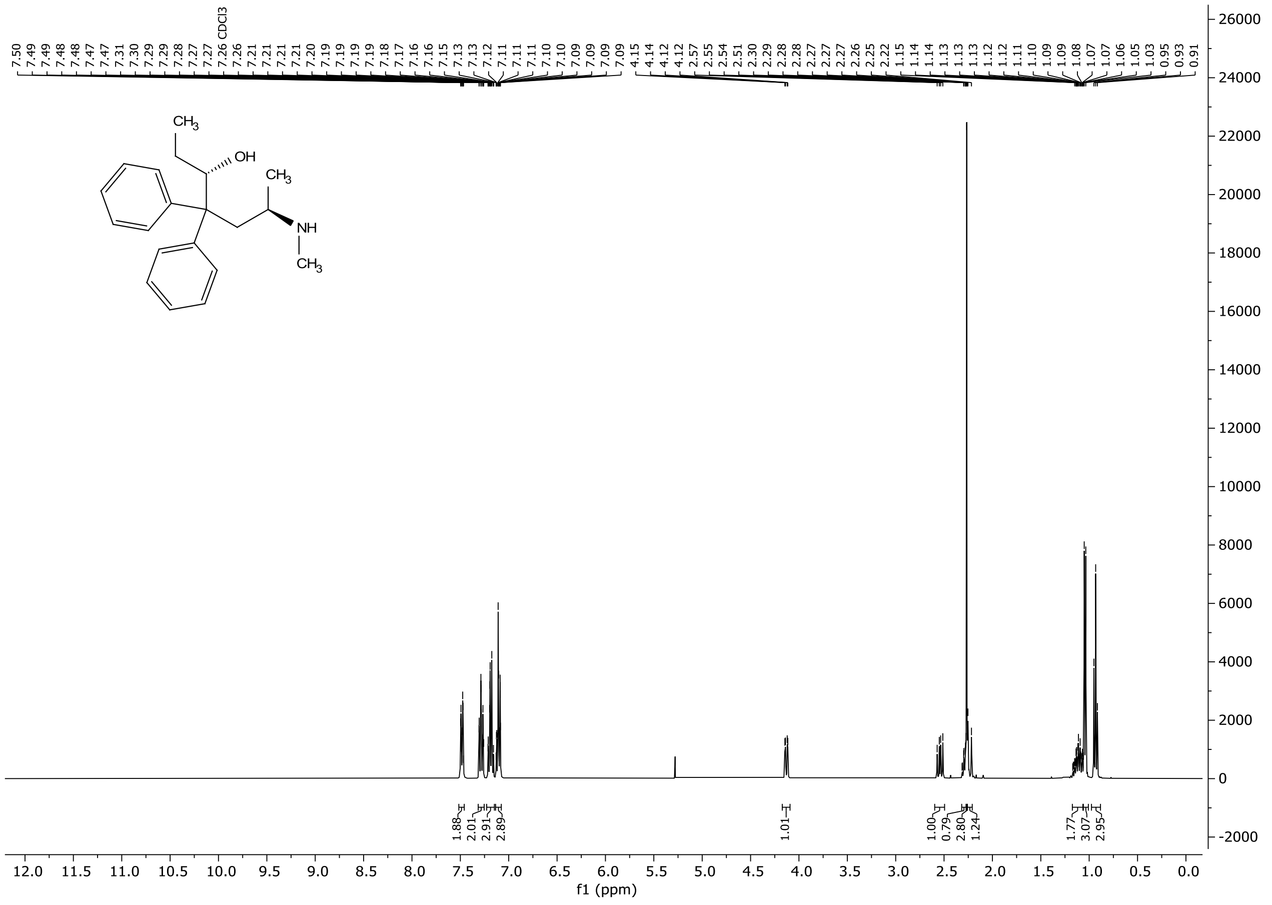

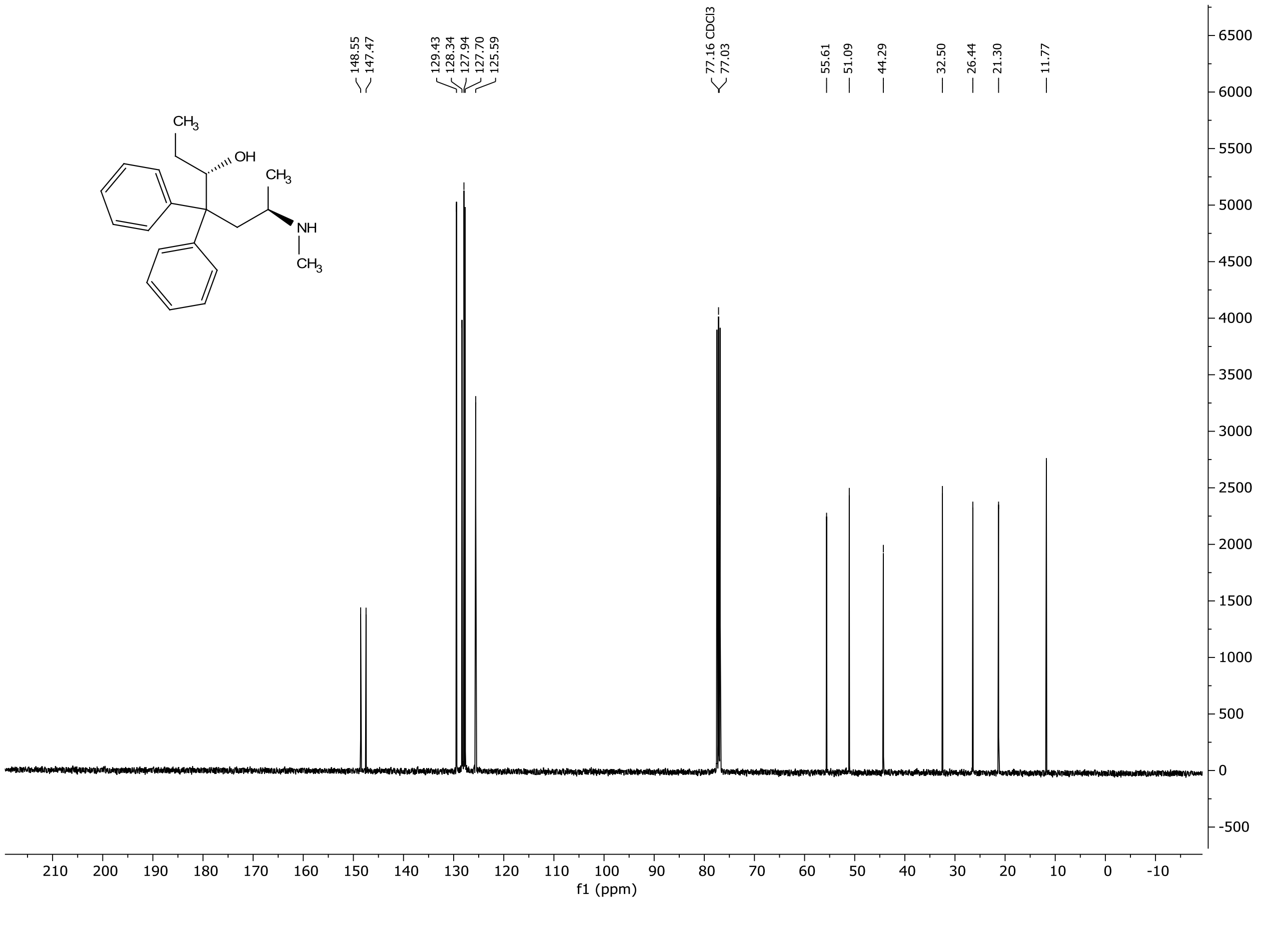

UPLC chromatogram of (3*S*,6*R*)-6-(methylamino)-4,4-diphenylheptan-3-ol ((3*S*,6*R*)-**NML**)

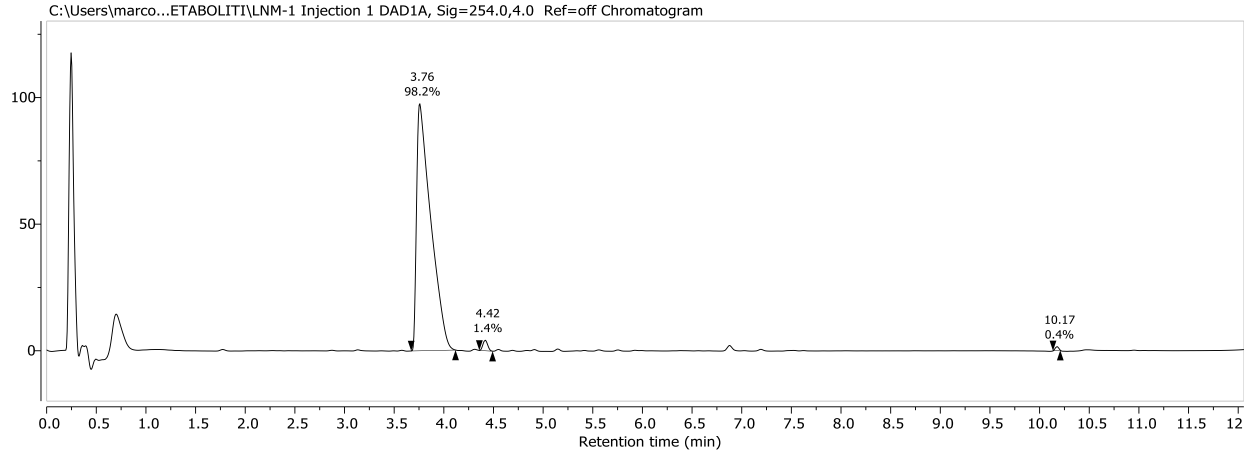

Chiral HPLC chromatogram of (3*S*,6*R*)-6-(methylamino)-4,4-diphenylheptan-3-ol ((3*S*,6*R*)-**NML**)

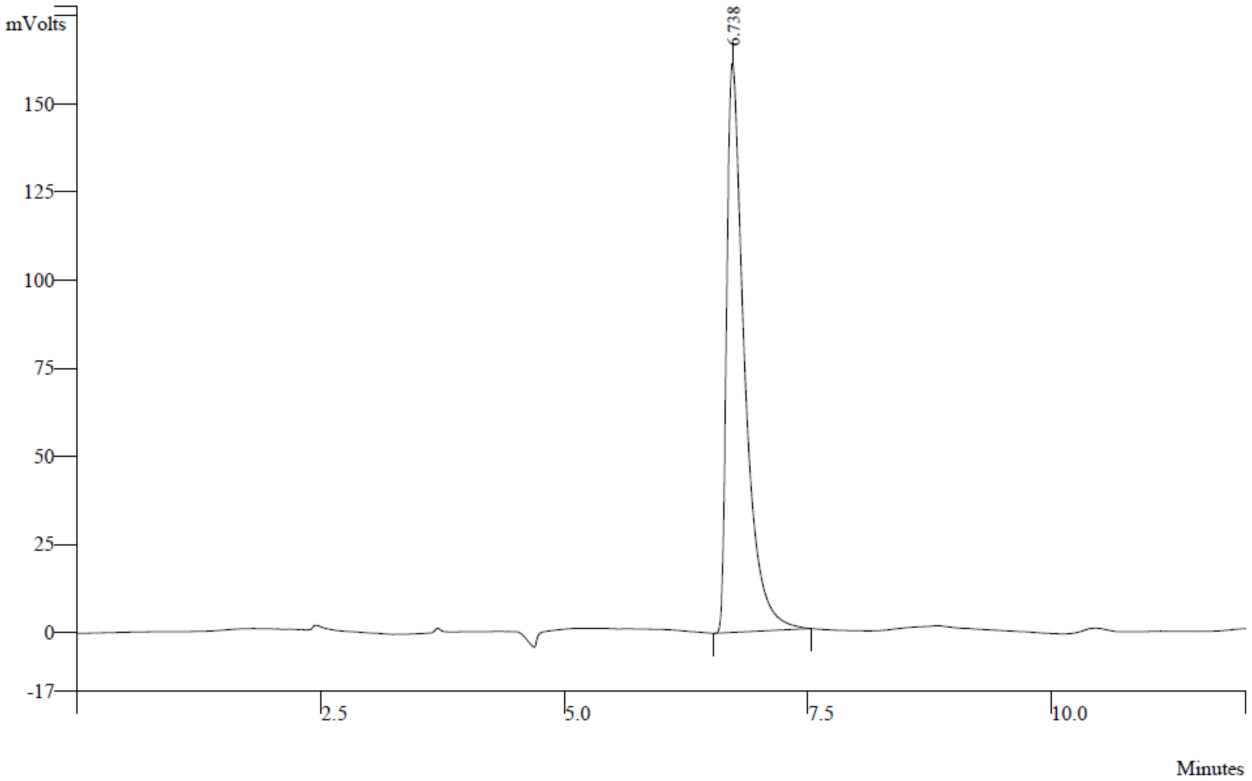

Peak No	Ret Time (min)	Peak Area (counts)	Result ()
1	6,738	2068803	100,00
		**2068803**	**100,00**

^{1}H and ^{13}C NMR spectra of (3*R*,6*S*)-6-(methylamino)-4,4-diphenylheptan-3-ol ((3*R*,6*S*)-**NML**)

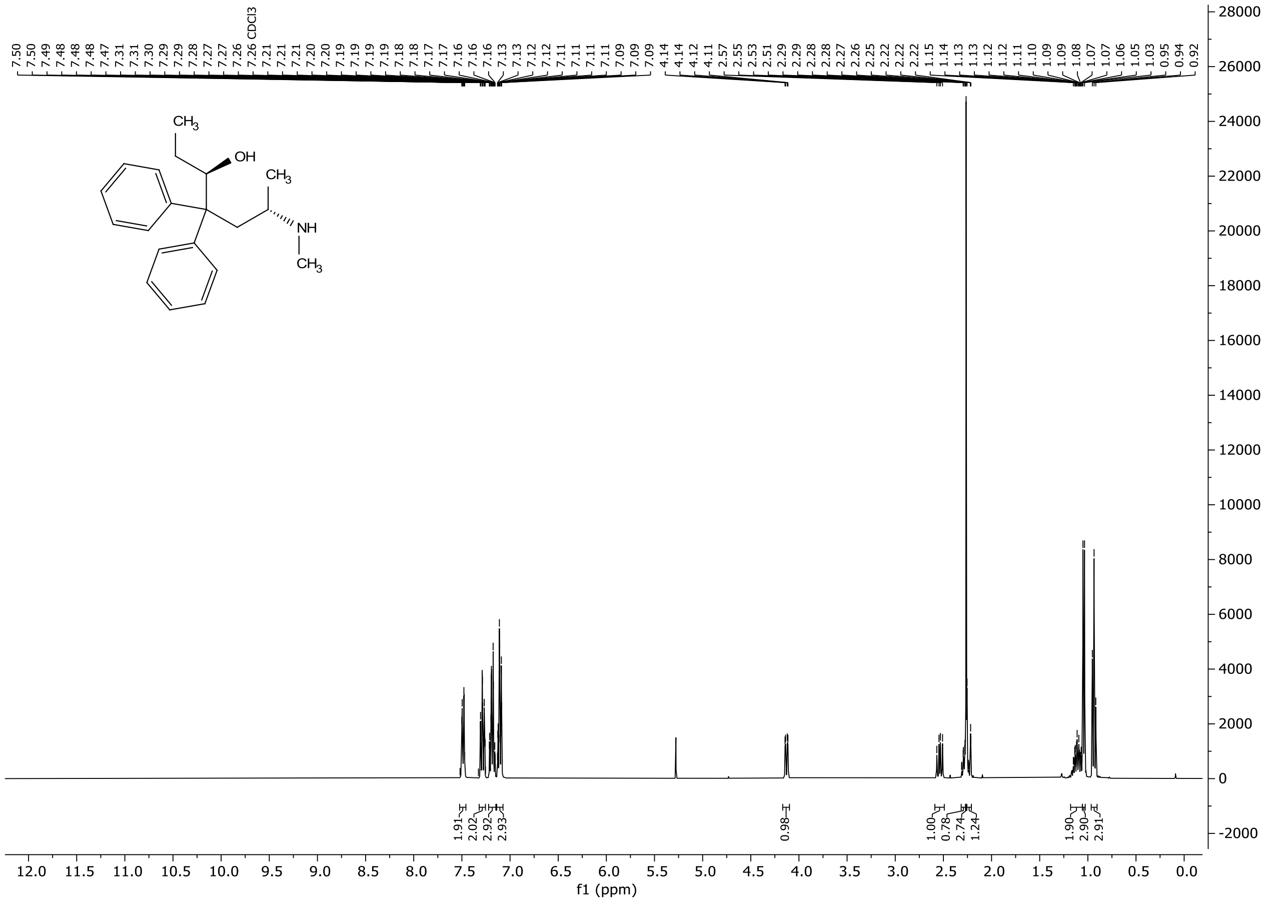

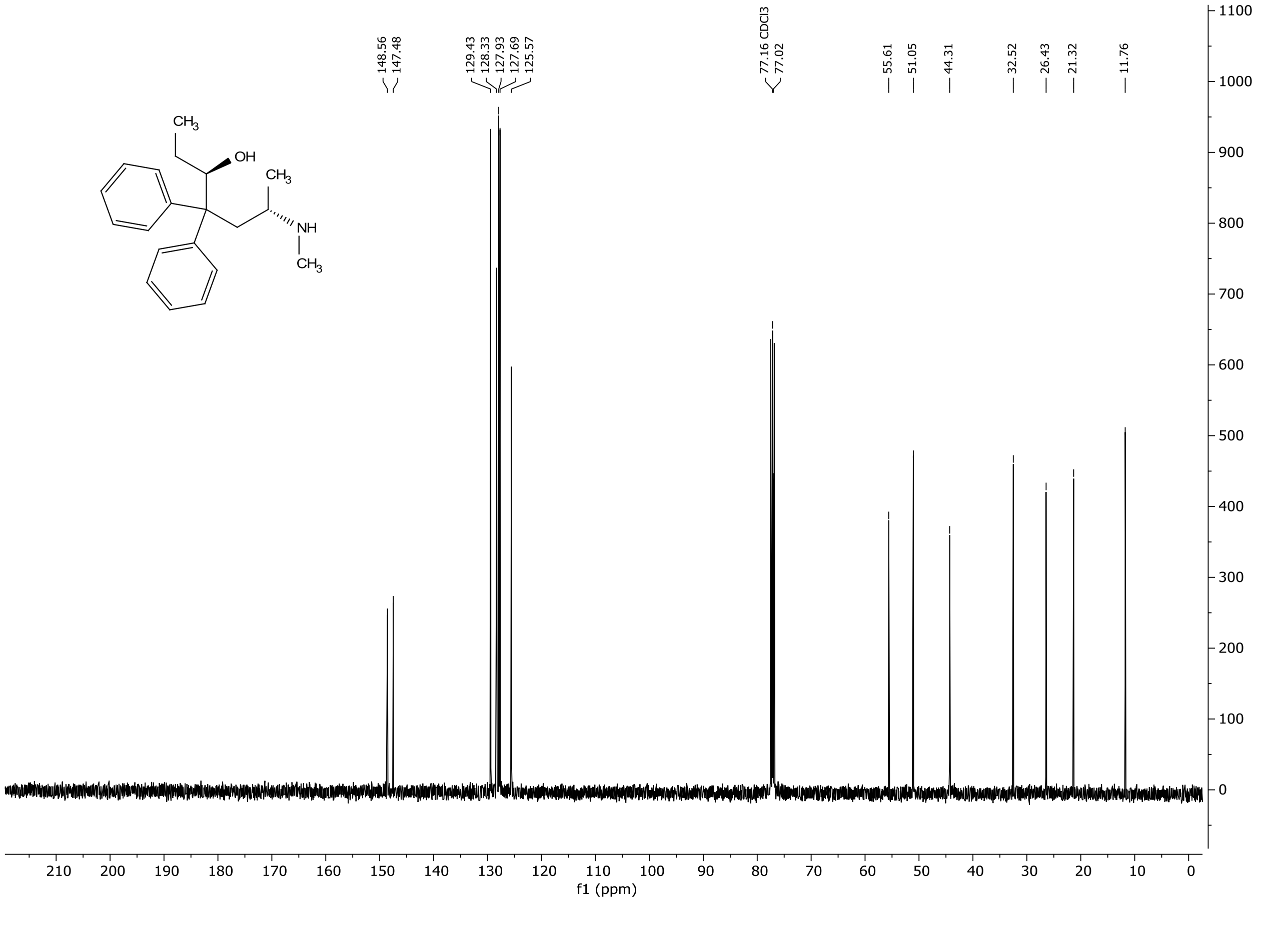

UPLC chromatogram of (3*R*,6*S*)-6-(methylamino)-4,4-diphenylheptan-3-ol ((3*R*,6*S*)-**NML**)

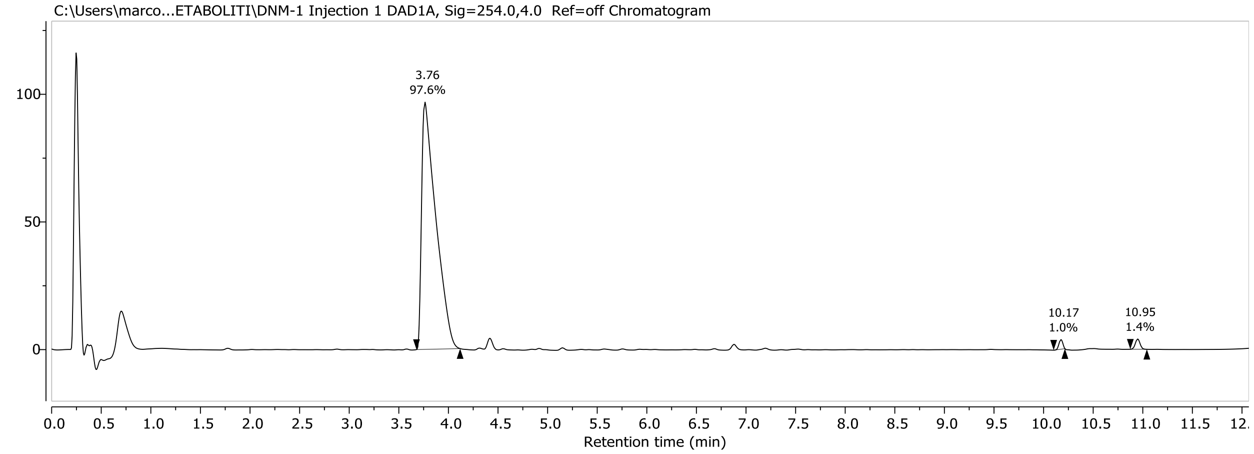

Chiral HPLC chromatogram of (3*R*,6*S*)-6-(methylamino)-4,4-diphenylheptan-3-ol ((3*R*,6*S*)-**NML**)

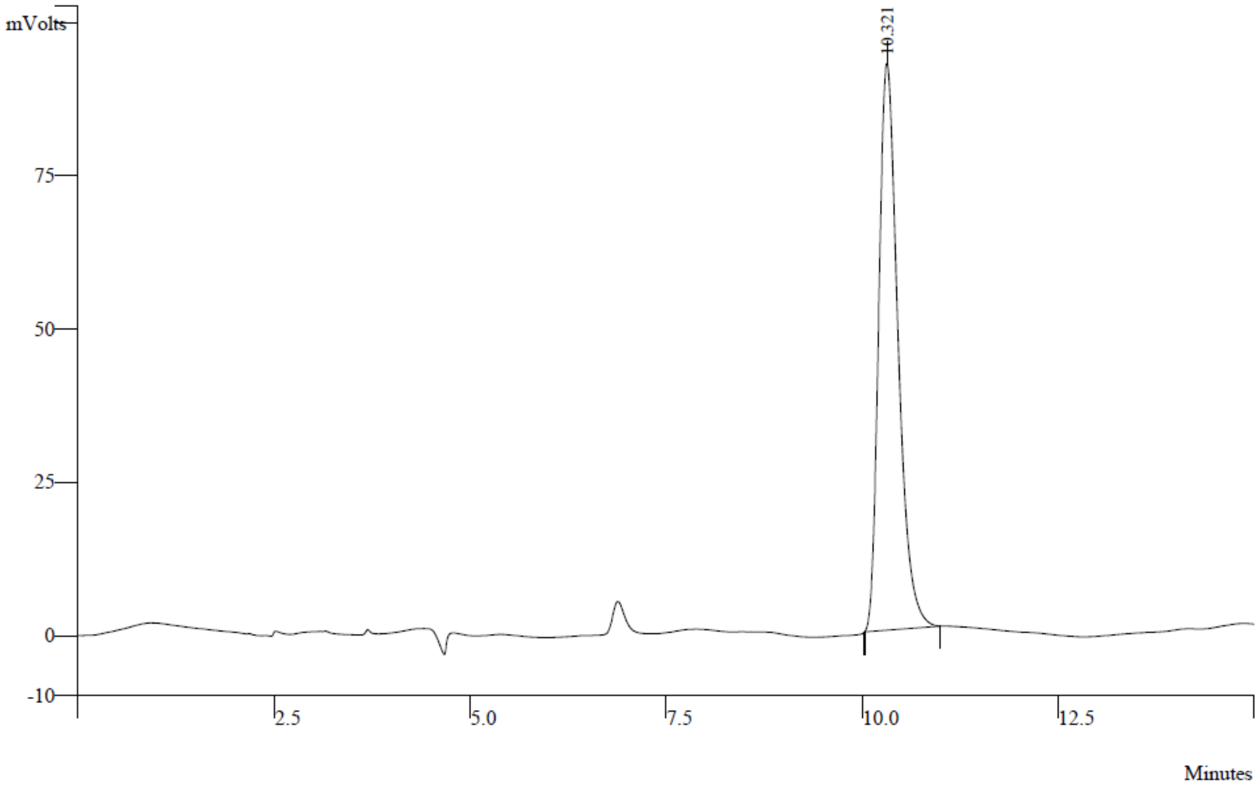

Peak No	Ret Time (min)	Peak Area (counts)	Result ()
1	10,321	1579396	100,00
		**1579396**	**100,00**

X-ray crystallography

<u>Experimental</u>. Single crystals suitable for the X-ray experiment of the hydrochlorides of (3*S*,6*S*)-, (3*R*,6*S*)-, (3*R*,6*R*)-, (3*S*,6*R*)-**ML**, **NML**, and **8** were obtained after a long and patient exploration of the best crystallization conditions, by testing several different crystallization solvents and mixtures thereof, temperatures, crystallization conditions and also by testing when necessary different counter anions, usually by a two-solvent mixture such as dichloromethane/*n*-heptane (all **NML**); ethyl acetate/diethyl ether ((3*S*,6*S*)-**ML** and (3*R*,6*R*)-**ML**); tetrahydrofuran/*n*-heptane ((3*R*,6*S*)-**ML**); dichloromethane/HCl in diethyl ether (vapor exchange, (3*S*,6*R*)-**ML**); dichloromethane/*n*-pentane ((3*S*,6*R*)-**8**); toluene/*n*-pentane ((3*R*,6*R*)-**8**); (3*R*,6*S*)-**8** and (3*S*,6*S*)-**8** were instead crystallized from an acetonitrile solution.

In all cases, several specimens were screened in order to select the most promising items, which were picked up with a nylon loop and mounted on the top of the goniometer head of a Rigaku-OD Gemini E diffractometer, equipped with sealed tube Enhance Cu X–ray source and with an EOS CCD area detector, replaced after diffractometer refurbishment with an Atlas detector, kindly provided by Prof. K. Rissanen. All the raw data diffraction were collected at room temperature in the range 296(1)-302(2) K, through the ω–scans technique, using graphite-monochromated Cu Kα radiation (λ=1.54184 Å). In most cases (9/12), samples proved to be very little diffracting, so data acquisition was performed in a 512×512-pixel mode and 4×4-pixel binning, otherwise with 1024×1024-pixel mode and 2×2-pixel binning. Data collection, reduction, and finalization were made using the *CrysAlisPro* software, Version 1.171.42. Data collections were carried over allowing for the greatest possible redundancy and correcting data for Lorentz and polarization effects. An absorption correction was performed empirically, by means of a multi-scan approach, with the scaling algorithm *SCALE3 ABSPACK*, using equivalent reflections.

Accurate unit cell parameters were obtained by the least-squares refinement of the widest possible number of strongest reflections, chosen throughout the whole data collection. The crystal and equipment stability were checked by monitoring two reference frames every 50 frames for all compounds; besides, a manual data reduction was performed at the end of all data collections to account for sample wobbling/motion. No sample degradation, nor significant change in peak intensities were observed during all experiments. All structures were solved by direct phasing and refined by full-matrix least squares based on F_o^2 with the SHELXT[1] and SHELXL[2] programs through the OLEX2 program interface[3]; the latter has been used also to provide the graphics material, in the form of ORTEP-like[4] representations (Figures S1 to S9). Non-H atoms were allowed to vibrate anisotropically in the last cycles of refinement, whereas the hydrogen atoms were placed in calculated positions and refined as a riding model, with their displacement parameters calculated as 1.2 (or 1.5 for the methyl groups) times the Ueq of the 'parent' atom.

The examination of the diffraction data of (3*R*,6*R*)- and (3*S*,6*S*)-**NML** showed that these compounds crystallized as non-merohedral two-component twins. In (3*S*,6*S*)-**NML**, the second component was

individuated by a rotation of 179.99° about the [0.00 0.00 1.00] direction in the reciprocal space and the two components account for 73.89% and 26.11%, respectively, of the diffraction peaks. Likewise, in (3*R*,6*R*)-**NML** the second component is rotated by a 173.11° about the [0.02 0.99 -0.12] direction in the reciprocal space and the first and second components account for 73.76% and 26.24% of the diffraction peaks. In both cases, twin data finalization [a] showed that the R_{int} values of the data pertaining to the minor component were significantly worse than that of the major component; however, considering both components in the refinement significantly lowered the quality of the final solution only in the case of (3*R*,6*R*)-**NML**; for this reason, the structure of this compound was solved using solely the data of the major component. Notably, the asymmetric units of the compounds (3*R*,6*S*)-**ML** and (3*R*,6*R*)-ML contain two independent molecules, which show identical configuration of the chiral centers. Also interestingly, the hydrochloride of (3*S*,6*R*)-**ML** is the only compound of the whole series which contains a co-crystallized HCl molecule accompanying the salt, probably due to the specific crystallization conditions (vapor exchange between a DCM solution of the freebase and a large excess of an HCl solution in Et_2O). Full listings of atomic coordinates, bond lengths and angles, anisotropic thermal parameters of all compounds are available in the form of .cif files, that have been deposited at the Cambridge Crystallographic Data Center (CCDC), with deposition numbers 2369660 - 2369671. The data can be obtained free of charge from The Cambridge Crystallographic Data Centre via www.ccdc.cam.ac.uk/structures.

Brief structural comment. A summary of data collection and refinement parameters for the crystal structures of the hydrochlorides of (3*S*,6*S*)-, (3*R*,6*S*)-, (3*R*,6*R*)-, (3*S*,6*R*)-**ML**, -**NML**, and -**8** are reported in Tables S2 and S3, respectively. A complete list of bond distances and angles are available in the pertinent .cif files deposited at CCDC. All the above compounds crystallize either in the monoclinic or in the orthorhombic crystal system. In particular, one half of the structures has been solved in the $P2_1$ and the other one in the $P2_12_12_1$ space group. When a compound crystallizes in the orthorhombic crystal system, the unit cell always hosts four molecules. This is true only for two of the compounds crystallizing in the monoclinic system, that is, the hydrochlorides of (3*R*,6*S*)-**ML** and (3*R*,6*R*)-**ML**, which are also the only compounds showing two independent molecules in the asymmetric unit.

The absolute configurations of the C2 and C6 chiral centers of investigated compounds were crystallographically determined by means of the anomalous dispersion effect (Flack parameter mostly close to 0.0); a series of ORTEP-like representations of all compounds showing the stereochemistry of the chiral centers has also been attached to the SI (Figures S10-S15). Apparently, there is not a strict correlation between the crystal system and the stereochemistry of the chiral centers. Nevertheless, it can be noted that two thirds of the molecules where the C2 and C6 chiral centers have identical configuration (i.e. *R*, *R* / *S*, *S*) belong to the monoclinic system ((3*S*,6*S*)-**NML**, (3*R*,6*R*)-**ML** and (3*S*,6*S*)-**8** and (3*R*,6*R*)-**8**); likewise, two thirds

of the molecules where the two chiral centers have different configurations (i.e. *R*, *S* / *S*, *R*) belong to the orthorhombic system ((3*R*,6*S*)-**NML,** (3*S*,6*R*)-**NML** and (3*R*,6*S*)-**8** and (3*S*,6*R*)-**8**).

An inspection of the metrical data for all compounds (see deposited .cif files) did not reveal bond lengths or angles showing values outside the expected ones for this kind of molecules and thus do not deserve any comment. Quite interestingly, none of the compounds showed co-crystallized solvent molecules in the solid state, despite the use of various different crystallization solvents; the unit cells of all compounds do not present in fact sufficiently large voids to host such molecules. The unique exception to the above is (3*S*,6*R*)-**ML**, which shows a second HCl molecule hydrogen-bonded to the chloride counter anion.

As for nonbonding interactions, despite the presence of (at least) two phenyl rings in all the investigated structures, no π-π interactions have been identified. A list of hydrogen-mediated interactions for which the donor-acceptor (D···A) distance is within 4.0 Å and the pertinent angle (D-H···A) is greater than 120° is listed in Table S1 of the SI. Only few of such interactions are canonical hydrogen bonds; most of them describe approaches of hydrogen atoms bound to carbon atoms to the chloride anions or to the oxygen atom(s). True hydrogen bonds usually are intramolecular contacts and are often the tightest interactions found for a given structure. As expected, the crystal packing of the hydrochlorides is sustained by the interactions formed by the chloride counter anions; in **8**, instead, nonbonding interactions help to stabilize 1D motifs propagating mostly along the crystallographic *a* and *b* axes (not shown here).

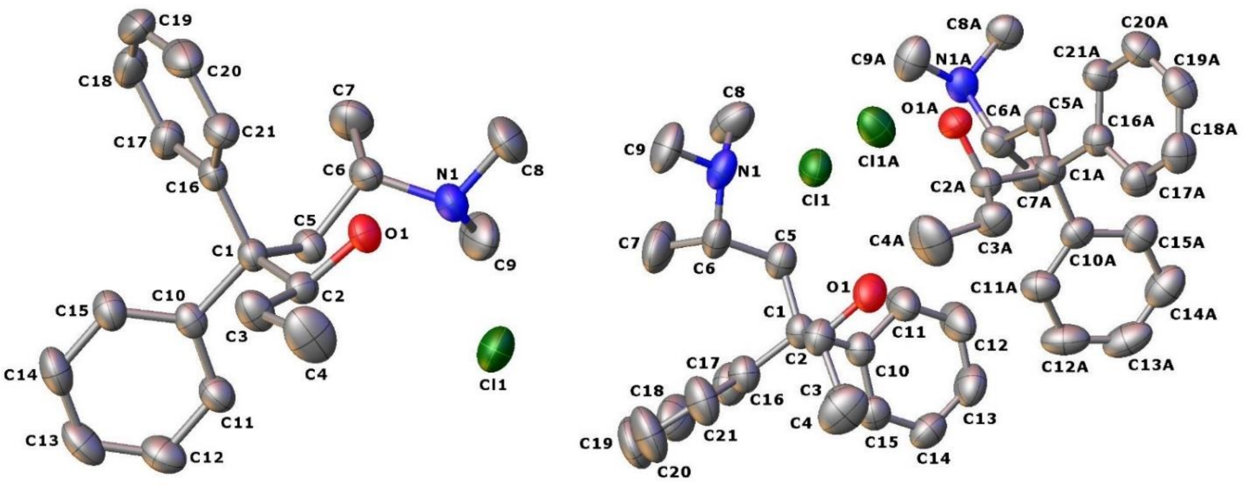

Figure S1. An ORTEP-like representation of the asymmetric unit content of compounds (3*S*,6*S*)-**ML** (left) and (3*R*,6*S*)-**ML** (right; two independent molecules in the asymmetric unit), together with the chosen numbering scheme. Hydrogen atoms omitted for clarity.

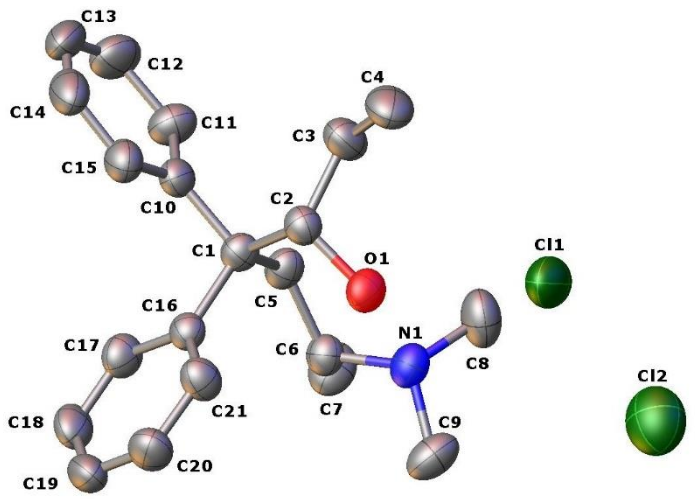

Figure S2. An ORTEP-like representation of the asymmetric unit content of compound (3*S*,6*R*)-**ML**, together with the chosen numbering scheme. Hydrogen atoms omitted for clarity.

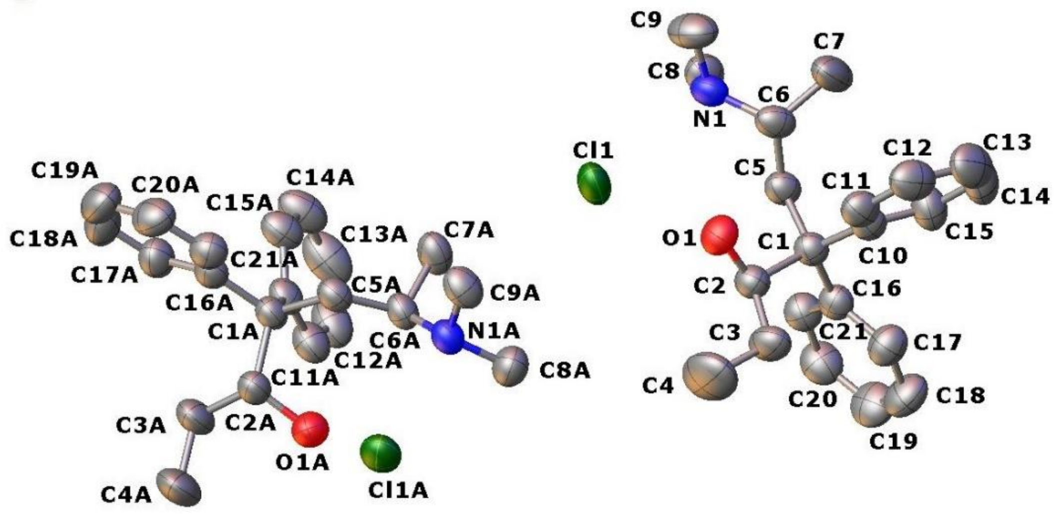

Figure S3. An ORTEP-like representation of the asymmetric unit content of compound (3*R*,6*R*)-**ML** (two independent molecules in the asymmetric unit), together with the chosen numbering scheme. Hydrogen atoms omitted for clarity.

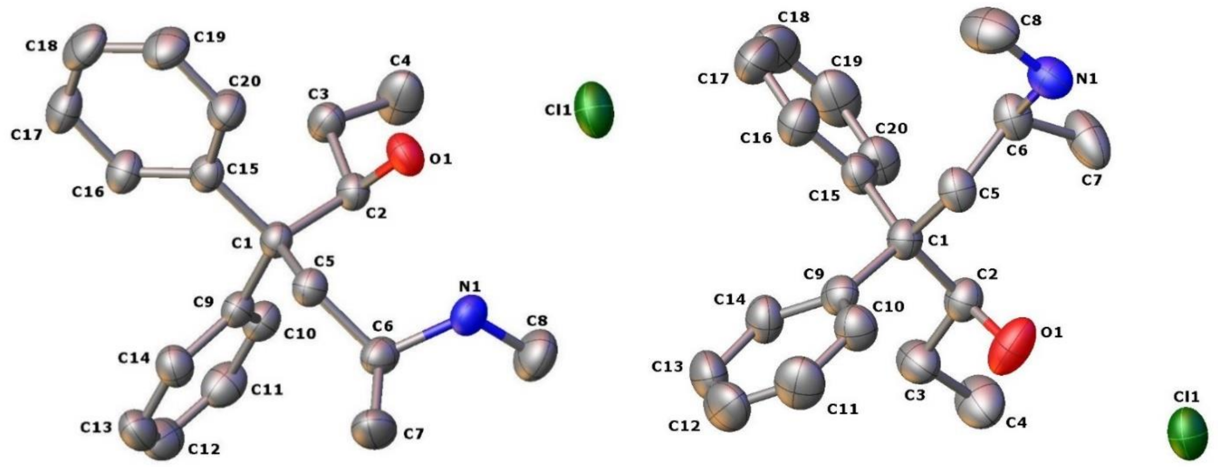

Figure S4. An ORTEP-like representation of the asymmetric unit content of compounds (3*R*,6*S*)-**NML** (left) and (3*S*,6*S*)-**NML** (right), together with the chosen numbering scheme. Hydrogen atoms omitted for clarity.

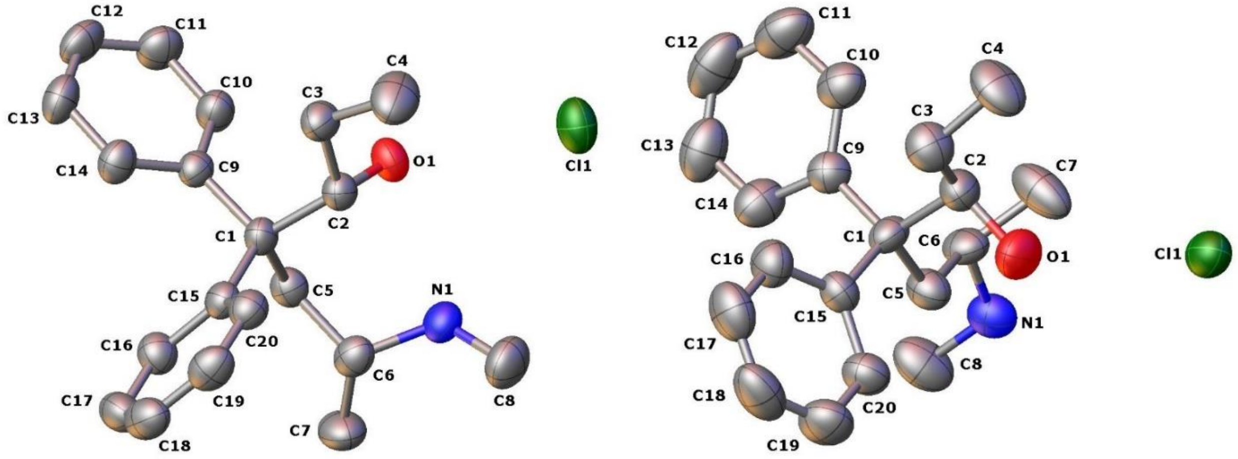

Figure S5. An ORTEP-like representation of the asymmetric unit content of compounds (3*S*,6*R*)-**NML** (left) and (3*R*,6*R*)-**NML** (right), together with the chosen numbering scheme. Hydrogen atoms omitted for clarity.

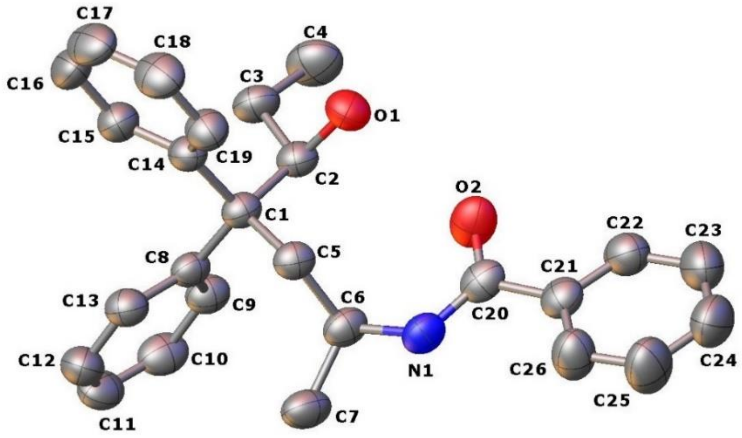

Figure S6. An ORTEP-like representation of the asymmetric unit content of compound (3*R*,6*S*)-**8**, together with the chosen numbering scheme. Hydrogen atoms omitted for clarity.

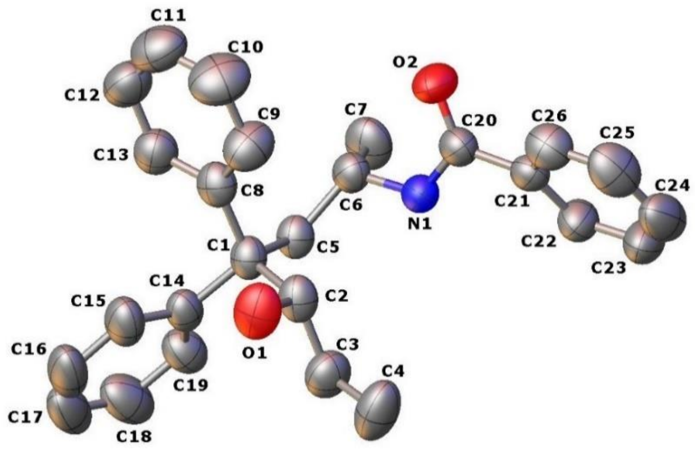

Figure S7. An ORTEP-like representation of the asymmetric unit content of compound (3*S*,6*S*)-**8**, together with the chosen numbering scheme. Hydrogen atoms omitted for clarity.

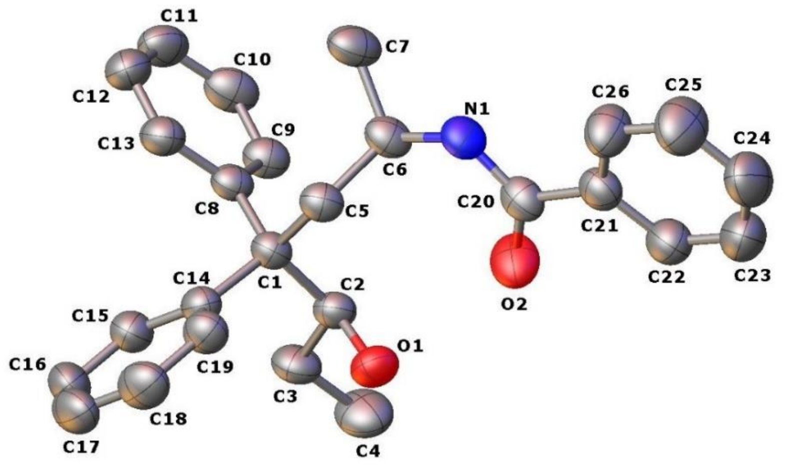

Figure S8. An ORTEP-like representation of the asymmetric unit content of compound (3*S*,6*R*)-**8**, together with the chosen numbering scheme. Hydrogen atoms omitted for clarity.

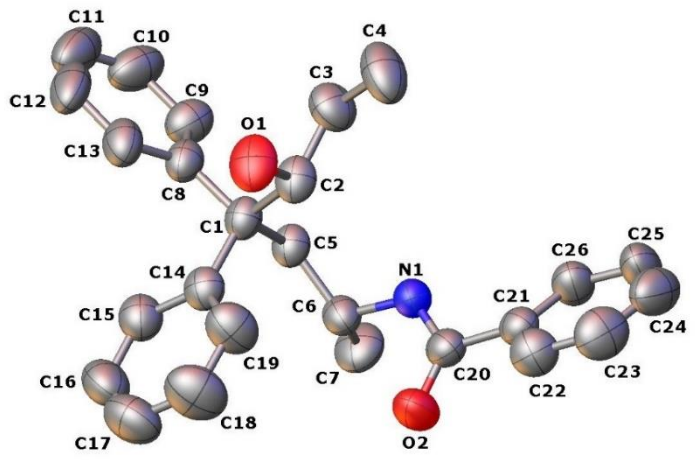

Figure S9. An ORTEP-like representation of the asymmetric unit content of compound (3*R*,6*R*)-**8**, together with the chosen numbering scheme. Hydrogen atoms omitted for clarity.

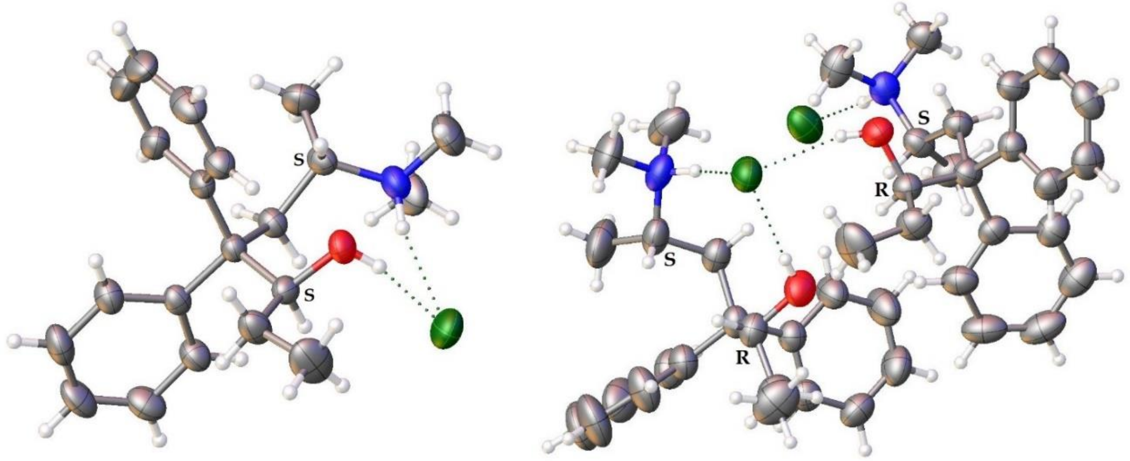

Figure S10. An ORTEP-like drawing of compounds (3*S*,6*S*)-**ML** (left) and (3*R*,6*S*)-**ML** (right) indicating the absolute configuration of the chiral centers. Hydrogen bonds involving chloride counter anions also highlighted.

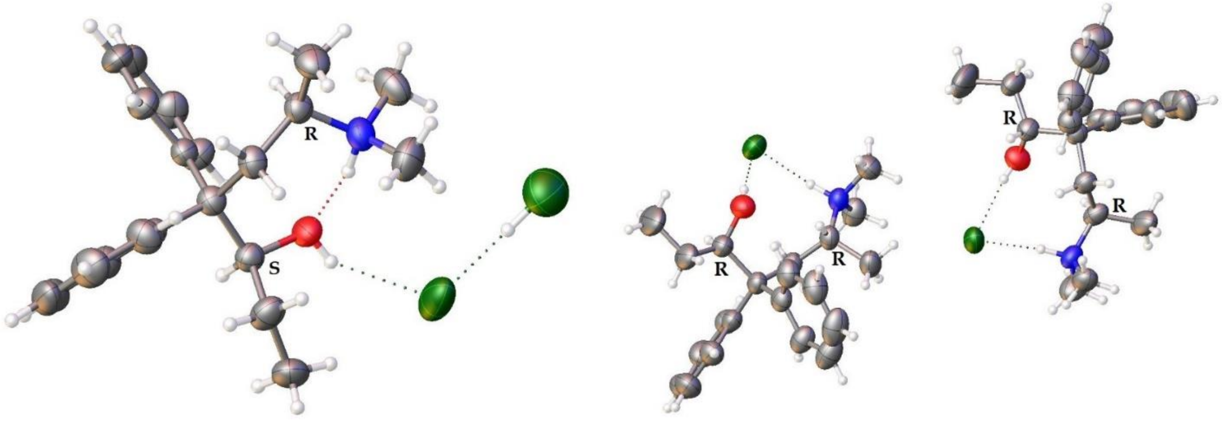

Figure S11. An ORTEP-like drawing of compounds (3*S*,6*R*)-**ML** (left) and (3*R*,6*R*)-**ML** (right) indicating the absolute configuration of the chiral centers. Hydrogen bonds involving chloride counter anions also highlighted.

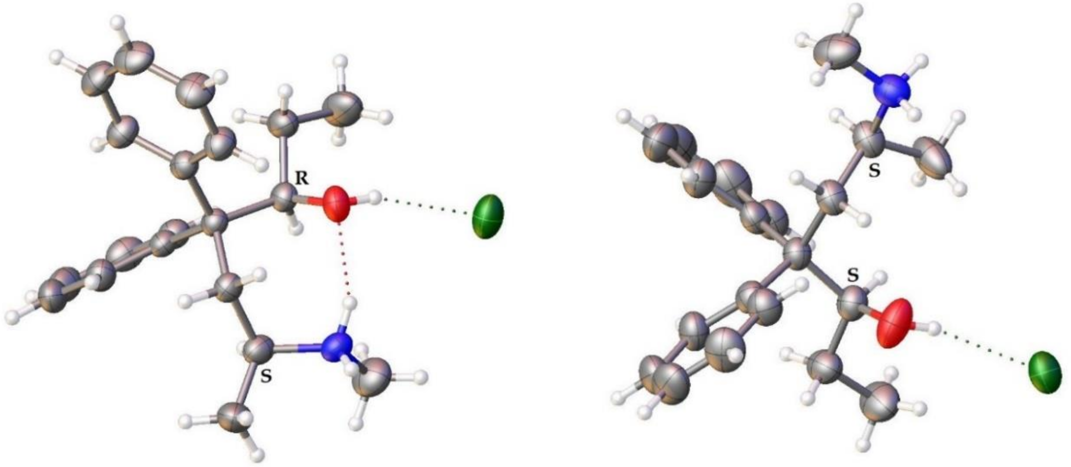

Figure S12. An ORTEP-like drawing of compounds (3*R*,6*S*)-**NML** (left) and (3*S*,6*S*)-**NML** (right) indicating the absolute configuration of the chiral centers. Hydrogen bonds involving chloride counter anions also highlighted.

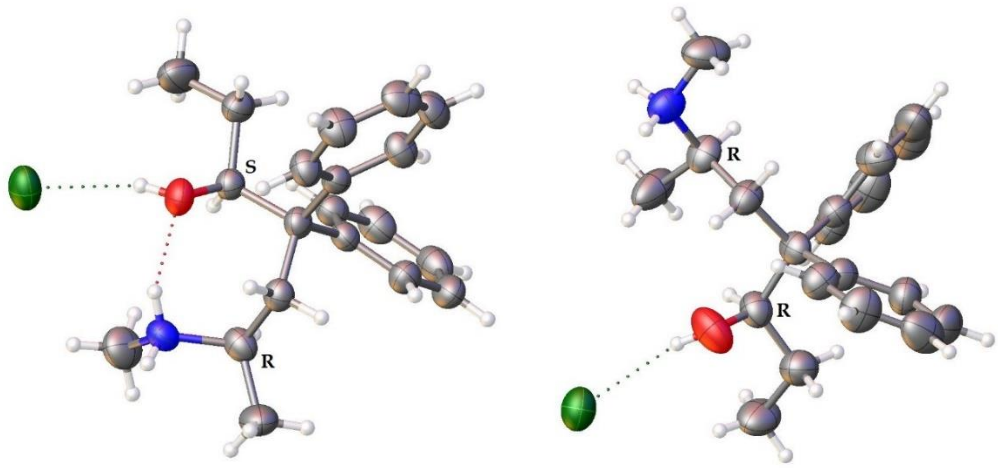

Figure S13. An ORTEP-like drawing of compounds (3*S*,6*R*)-**NML** (left) and (3*R*,6*R*)-**NML** (right) indicating the absolute configuration of the chiral centers. Hydrogen bonds involving chloride counter anions also highlighted.

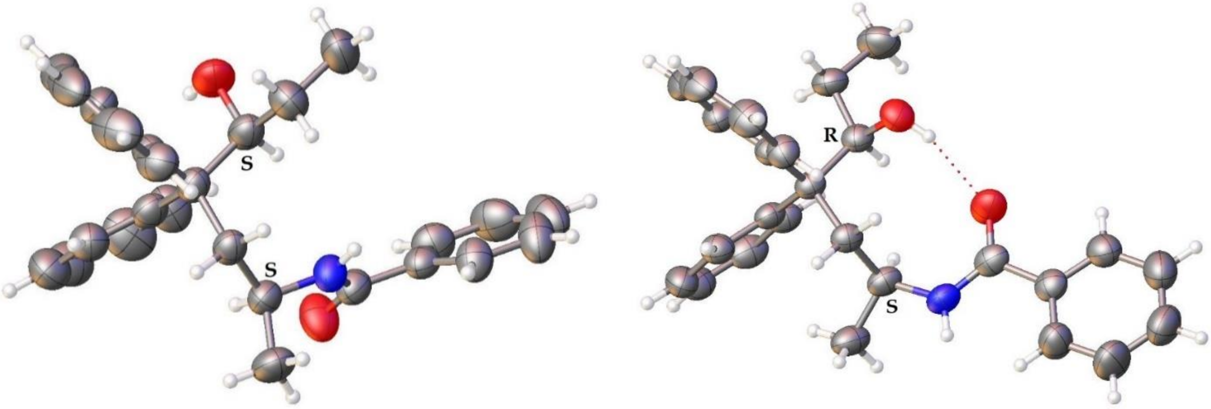

Figure S14. An ORTEP-like drawing of compounds (3*S*,6*S*)-**8** (left) and (3*R*,6*S*)-**8**, (right) indicating the absolute configuration of the chiral centers. Intramolecular hydrogen bonds (if present) also highlighted.

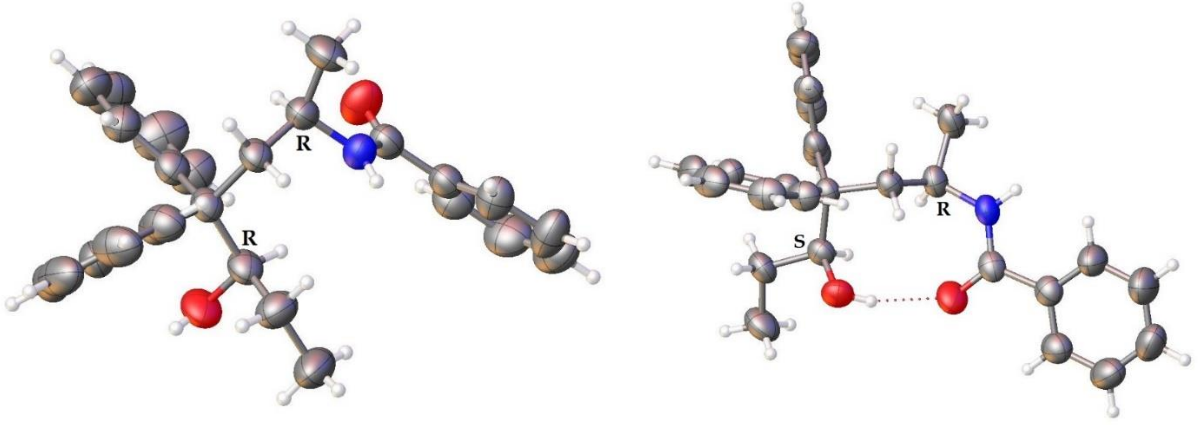

Figure S15. An ORTEP-like drawing of compounds (3*R*,6*R*)-**8** (left) and (3*S*,6*R*)-**8**, (right) indicating the absolute configuration of the chiral centers. Intramolecular hydrogen bonds (if present) also highlighted.

1. G.M. Sheldrick, Acta Crystallogr. 2015, A71, 3-8
2. G.M. Sheldrick, Acta Crystallogr. 2015, C71, 3-8
3. OLEX2, Version 1.5. O.V. Dolomanov, L.J. Bourhis, R.J. Gildea, J.A.K. Howard, H. Puschmann, J. Appl. Cryst. 2009, 42, 339-341
4. C.K. Johnson, ORTEP, Report ORNL–5138, Oak Ridge National Laboratory, Oak Ridge, TN, 1976

Table S1. List of the nonbonding contacts of investigated compounds for which Donor···Acceptor distance is within 4.0 Å and D-H···A angle is greater than 120°; the shortest **intramolecular** interaction for each compound is written in *italics*, the shortest **intermolecular** interaction underlined. Contacts with Donor···Acceptor distance smaller than 3.0 Å marked with an asterisk (*).

**Compound**	**Donor (D) atom**	**Acceptor (A) atom**	**Distance D···A (Å)**	**Angle D–H–A (°)**	**Symmetry op.**
(3*S*,6*S*)-**ML**	*O1*	*Cl1*	*3.086*	*148.0*	*x, y, z*
	N1	Cl1	3.138	161.4	*x, y, z*
	C2	Cl1	3.474	122.3	*x, y, z*
	C8	Cl1	3.897	132.6	*-1/2 + x, 1/2 - y, 1 - z*
	C9	Cl1	3.569	127.0	*-1/2 + x, 1/2 - y, 1 - z*
	C14	Cl1	3.864	153.5	*2 - x, 1/2 + y, 3/2 - z*
(3*R*,6*S*)-**ML**	O1	Cl1	3.120	176.0	*x, y, z*
	N1	*Cl1*	*3.029*	*158.1*	*x, y, z*
	C5	Cl1	3.947	124.2	*x, y, z*
	C7	Cl1	3.517	120.7	*- x, -1/2 + y, 1 - z*
	C7	O1A	3.850	140.1	*- x, -1/2 + y, 1 - z*
	C8	Cl1A	3.660	161.5	*x, y, z*
	C9	Cl1A	3.603	141.3	*2 - x, 1/2 + y, 3/2 - z*
	C9	O1A	3.293	173.1	*-1 + x, y, z*
	C11	Cl1A	3.894	144.7	*x, y, z*
	O1A	Cl1	3.147	162.6	*x, y, z*
	N1A	Cl1A	3.043	169.4	*x, y, z*
	C2A	O1	3.757	166.4	*x, y, z*
	C7A	Cl1	3.946	148.3	*1 + x, y, z*
	C7A	Cl1A	3.847	138.8	*x, y, z*
	C8A	Cl1	3.760	162.9	*1 + x, y, z*
	C8A	Cl1A	3.902	155.9	*1 - x, 1/2 + y, 1 - z*
	C9A	Cl1	3.807	159.7	*x, y, z*
	C11A	O1	3.558	153.4	*x, y, z*
	C21A	Cl1A	3.827	155.5	*1 - x, 1/2 + y, 1 - z*
(3*R*,6*S*)-**NML**	O1	Cl1	3.022	155.8	*x, y, z*
	N1	Cl1	3.896	136.1	*x, y, z*
	N1	Cl1	3.083	167.9	*-1/2 + x, 1/2 - y, 1 - z*
	N1	*O1*	*2.781**	*156.0*	*x, y, z*
	C4	Cl1	3.968	150.5	*x, y, z*
	C7	Cl1	3.976	163.6	*-1 + x, y, z*
	C8	Cl1	3.833	174.4	*-1 + x, y, z*
	C11	Cl1	3.702	166.8	*-1/2 + x, 3/2 - y, 1 - z*
	C18	Cl1	3.883	164.8	*5/2 - x, 1 - y, 1/2 + z*

(3*S*,6*S*)-**NML**	*O1*	*Cl1*	*3.105*	*146.6*	*x, y, z*
	N1	Cl1	3.175	177.9	*x, 1 + y, z*
	N1	Cl1	3.153	158.9	*2 - x, 1/2 + y, 1 - z*
	C5	Cl1	3.634	131.3	*2 - x, 1/2 + y, 1 - z*
	C7	Cl1	3.702	147.1	*x, 1 + y, z*
	C7	Cl1	3.816	147.8	*1 - x, 1/2 + y, 1 - z*
	C20	Cl1	3.996	134.2	*1 - x, 1/2 + y, 1 - z*
(3*S*,6*R*)-**ML**	Cl2	Cl1	3.144	167.6	*x, y, z*
	O1	Cl1	3.112	148.7	*x, y, z*
	N1	*O1*	*2.627**	*163.8*	*x, y, z*
	C4	Cl1	3.738	163.1	*x, y, z*
	C7	Cl1	3.976	170.3	*x, 1 + y, z*
	C8	Cl1	3.705	139.6	*1 - x, -1/2 + y, - z*
	C9	Cl2	3.962	156.5	*2 - x, -1/2 + y, - z*
	C9	Cl2	3.789	163.7	*x, y, z*
	C11	Cl2	3.845	123.0	*1 - x, -1/2 + y, - z*
	C14	O1	3.629	122.4	*1 - x, -1/2 + y, 1 - z*
	C19	O1	3.739	150.1	*2 - x, -1/2 + y, 1 - z*
	C20	Cl1	3.902	133.1	*2 - x, -1/2 + y, 1 - z*
	C21	O1	2.967*	120.0	*x, y, z*
(3*R*,6*R*)-**ML**	O1	Cl1	3.084	172.9	*x, y, z*
	N1	Cl1	3.049	172.3	*x, y, z*
	C5	Cl1	3.913	130.1	*x, y, z*
	C8	Cl1	3.959	159.8	*-x, -1/2 + y, 1 - z*
	C8	Cl1A	3.915	161.4	*-1 + x, y, z*
	C9	Cl1	3.970	127.2	*-x, 1/2 + y, 1 - z*
	C11	*O1*	*2.940**	*126.0*	*x, y, z*
	C19	O1	3.437	156.5	*x, -1 + y, z*
	C21	Cl1	3.786	142.3	*-x, -1/2 + y, 1 - z*
	O1A	Cl1A	3.105	172.9	*x, y, z*
	N1A	Cl1A	3.076	174.5	*x, y, z*
	C5A	Cl1A	3.860	134.8	*x, y, z*
	C8A	Cl1A	3.810	169.0	*1 - x, -1/2 + y, 1 - z*
	C9A	Cl1	3.900	162.0	*x, y, z*
	C9A	O1A	3.292	121.7	*1 - x, 1/2 + y, 1 - z*
	C11A	O1A	3.007	126.6	*x, y, z*
	C21A	Cl1A	3.937	144.1	*1 - x, 1/2 + y, 1 - z*
(3*S*,6*R*)-**NML**	O1	Cl1	3.021	158.1	*x, y, z*
	N1	Cl1	3.892	136.5	*x, y, z*
	N1	Cl1	3.086	168.0	*-1/2 + x, 1/2 - y, 1 - z*
	N1	*O1*	*2.793**	*155.6*	*x, y, z*
	C4	Cl1	3.965	153.4	*x, y, z*
	C7	Cl1	3.976	163.0	*-1 + x, y, z*
	C8	Cl1	3.828	173.6	*-1 + x, y, z*
	C12	Cl1	3.893	164.7	*5/2 - x, 1 - y, -1/2 + z*
	C19	Cl1	3.710	166.0	*-1/2 + x, 3/2 - y, 1 - z*
(3*R*,6*R*)-**NML**	*O1*	*Cl1*	*3.112*	*144.8*	*x, y, z*
	N1	Cl1	3.171	176.7	*1 + x, y, z*
	N1	Cl1	3.160	159.6	*1/2 + x, 3/2 - y, 1 - z*
	C5	Cl1	3.631	130.7	*1/2 + x, 3/2 - y, 1 - z*
	C7	Cl1	3.687	145.7	*1 + x, y, z*
	C7	Cl1	3.780	145.6	*1/2 + x, 1/2 - y, 1 - z*

(3*R*,6*S*)-**8**	*O1*	*O2*	*2.817**	*160.2*	*x, y, z*
	C5	O2	3.996	164.7	*1 - x, 1/2 + y, 3/2 - z*
	C7	O1	3.614	134.4	*1 - x, 1/2 + y, 3/2 - z*
	<u>C10</u>	<u>O2</u>	<u>3.317</u>	<u>121.8</u>	*2 - x, 1/2 + y, 3/2 - z*
	C11	O2	3.279	124.1	*2 - x, 1/2 + y, 3/2 - z*
(3*S*,6*S*)-**8**	*C2*	*N1*	*3.279*	*125.3*	*x, y, z*
	<u>N1</u>	<u>O2</u>	<u>3.031</u>	<u>145.4</u>	*1 - x, 1/2 + y, 1 - z*
	C5	O2	3.878	135.7	*1 - x, 1/2 + y, 1 - z*
	C7	O1	3.558	136.4	*1 + x, y, z*
	C7	O2	3.881	129.0	*1 - x, 1/2 + y, 1 - z*
	C22	O2	3.303	148.8	*1 - x, 1/2 + y, 1 - z*
	C26	O1	3.444	148.9	*- x, -1/2 + y, 1 - z*
(3*S*,6*R*)-**8**	*O1*	*O2*	*2.818**	*160.7*	*x, y, z*
	C5	O2	3.998	164.7	*1 - x, 1/2 + y, 3/2 - z*
	C7	O1	3.613	133.8	*1 - x, 1/2 + y, 3/2 - z*
	C10	O2	3.318	121.9	*- x, 1/2 + y, 3/2 - z*
	<u>C11</u>	<u>O2</u>	<u>3.276</u>	<u>124.2</u>	*- x, 1/2 + y, 3/2 - z*
(3*R*,6*R*)-**8**	*C2*	*N1*	*3.275*	*126.0*	*x, y, z*
	<u>N1</u>	<u>O2</u>	<u>3.023</u>	<u>145.4</u>	*1 - x, -1/2 + y, 1 - z*
	C4	O1	3.980	125.8	*- x, -1/2 + y, 1 - z*
	C5	O2	3.882	135.9	*1 - x, -1/2 + y, 1 - z*
	C7	O1	3.527	134.8	*1 + x, y, z*
	C7	O2	3.870	129.3	*1 - x, -1/2 + y, 1 - z*
	C22	O1	3.461	149.0	*- x, 1/2 + y, 1 - z*
	C26	O2	3.300	149.5	*1 - x, -1/2 + y, 1 - z*

Table S2. Summary of data collection parameters and crystal data for **methadols (MLs)** and **normethadols (NMLs)** hydrochlorides.

**Compound**	(3*S*,6*S*)-**ML**	(3*R*,6*S*)-**ML**	(3*R*,6*S*)-**NML**	(3*S*,6*S*)-**NML**	(3*S*,6*R*)-**ML**	(3*R*,6*R*)-**ML**	(3*S*,6*R*)-**NML**	(3*R*,6*R*)-**ML**
Radiation (all experiments)	Cu Kα (λ = 1.54184)							
Empirical formula	$C_{21}H_{30}NOCl$	$C_{21}H_{30}NOCl$	$C_{20}H_{28}NOCl$	$C_{20}H_{28}NOCl$	$C_{21}H_{31}NOCl_2$	$C_{21}H_{30}NOCl$	$C_{20}H_{28}NOCl$	$C_{20}H_{28}NOCl$
Formula weight	347.91	347.91	333.88	333.88	384.37	347.91	333.88	333.88
Temperature / K	299(1)	298(1)	301.1(8)	297.9(8)	296(1)	296(1)	297.0(8)	299(1)
Crystal system	orthorhombic	monoclinic	orthorhombic	monoclinic	monoclinic	monoclinic	orthorhombic	orthorhombic
Space group	$P2_12_12_1$	$P2_1$	$P2_12_12_1$	$P2_1$	$P2_1$	$P2_1$	$P2_12_12_1$	$P2_12_12_1$
a / Å	7.78340(10)	9.3591(3)	7.6712(2)	8.1854(2)	9.1436(2)	14.6508(2)	7.6661(6)	7.7697(3)
b / Å	11.7234(2)	14.3104(3)	11.4084(2)	7.7384(3)	10.0286(2)	9.5339(2)	11.4096(8)	8.1354(3)
c / Å	21.8612(3)	15.9110(4)	21.6005(3)	15.0977(4)	12.3266(2)	14.9642(2)	21.6022(11)	30.2456(14)
α / °	90.0	90.0	90.0	90.0	90.0	90.0	90.0	90.0
β / °	90.0	105.373(3)	90.0	91.339(3)	109.093(2)	92.3450(10)	90.0	90.0
γ / °	90.0	90.0	90.0	90.0	90.0	90.0	90.0	90.0
Volume/$Å^3$	1994.79(5)	2054.75(10)	1890.39(6)	956.06(5)	1068.14(4)	2088.44(6)	1889.5(2)	1911.81(14)
Z	4	4	4	2	2	4	4	4
ρ_{calc} Mg / m^3	1.158	1.125	1.173	1.160	1.195	1.107	1.174	1.160
μ / mm^{-1}	1.729	1.679	1.806	1.785	2.783	1.652	1.806	1.785
F(000)	752.0	752.0	720.0	360.0	412.0	752.0	720.0	720.0
Crystal size / mm^3	0.26 × 0.24 × 0.16	0.38×0.22×0.01	0.30×0.22×0.16	0.30×0.26×0.05	0.42×0.36×0.08	0.42×0.40×0.02	0.16×0.08×0.04	0.20×0.18×0.06
Reflections collected	33015	30349	19035	30368	17914	19773	5929	10103
Independent reflections / R_{int}	3665 / 0.0423	6223 / 0.0481	3755 / 0.0336	6934 / 0.0526	3650 / 0.0293	6170 / 0.0327	2827 / 0.0470	2023/ 0.0679
Restraints / parameters	0 / 222	1 / 443	0 / 212	1 / 213	1 / 235	1 / 443	0 / 212	0 / 212
Goodness-of-fit on F^2	1.060	1.045	1.047	0.907	1.036	1.016	1.033	0.820
Final *R* (R_1;wR_2) indexes [$I > 2\sigma(I)$]	0.0375, 0.1018	0.0374, 0.0879	0.0352, 0.0950	0.0414, 0.0979	0.0441, 0.1155	0.0408, 0.1047	0.0446, 0.0926	0.0379, 0.0678
Largest diff. peak / hole / e $Å^{-3}$	0.41 / -0.14	0.25 / -0.14	0.19 / -0.15	0.24 / -0.16	0.35 / -0.33	0.17 / -0.14	0.16 / -0.16	0.17 / -0.12

Table S3. Summary of data collection parameters and crystal data for compounds **8**.

**Compound**	(3*R*,6*S*)-**8**	(3*S*,6*S*)-**8**	(3*S*,6*R*)-**8**	(3*R*,6*R*)-**8**
Radiation (all experiments)	Cu Kα (λ = 1.54184)			
Empirical formula	$C_{26}H_{29}NO_2$	$C_{26}H_{29}NO_2$	$C_{26}H_{29}NO_2$	$C_{26}H_{29}NO_2$
Formula weight	387.50	387.50	387.50	387.50
Temperature / K	299.9(9)	302(2)	297.9(4)	297.9(6)
Crystal system	orthorhombic	monoclinic	orthorhombic	monoclinic
Space group	$P2_12_12_1$	$P2_1$	$P2_12_12_1$	$P2_1$
a / Å	9.3633(2)	8.8458(5)	9.3580(3)	8.8451(3)
b / Å	9.8710(2)	9.5347(5)	9.8801(3)	9.5203(5)
c / Å	23.3471(5)	13.1957(7)	23.3654(8)	13.1914(5)
α / °	90.0	90.0	90.0	90.0
β / °	90.0	95.507(5)	90.0	95.519(3)
γ / °	90.0	90.0	90.0	90.0
Volume/Å^3	2157.86(8)	1107.82(10)	2160.32(12)	1105.67(8)
Z	4	2	4	2
ρ_{calc} Mg / m^3	1.193	1.162	1.191	1.164
μ / mm^{-1}	0.582	0.566	0.581	0.568
F(000)	832.0	416.0	832.0	416.0
Crystal size / mm^3	0.68 × 0.12 × 0.10	0.28 × 0.22 × 0.18	0.44 × 0.24 × 0.12	0.52 × 0.20 × 0.16
Reflections collected	19322	16568	19352	11717
Independent reflections / R_{int}	4228 / 0.0281	4000 / 0.0733	4319 / 0.0452	3658 / 0.0463
Restraints / parameters	0 / 265	1 / 265	0 / 265	1 / 266
Goodness-of-fit on F^2 *	1.022	1.041	1.092	1.034
Final R (R_1;wR_2) indexes [$I > 2\sigma(I)$]	0.0394, 0.1024	0.0488, 0.1142	0.0426, 0.0997	0.0488, 0.1239
Largest diff. peak / hole / e Å^{-3}	0.12 / -0.19	0.18 / -0.14	0.18 / -0.19	0.12 / -0.15

*Goodness–of–fit = $[\Sigma (w (F_o^2 - F_c^2)^2] / (N_{obsvns} - N_{params})]^{1/2}$, based on all data; $R_1 = \Sigma ||F_o| - |F_c||/\Sigma |F_o|$; $wR_2 = [\Sigma w (F_o^2 - F_c^2)^2/\Sigma w (F_o^2)^2]^{1/2}$.

FLIPR assay concentration-response curves

(3*S*,6*R*)-**ML**

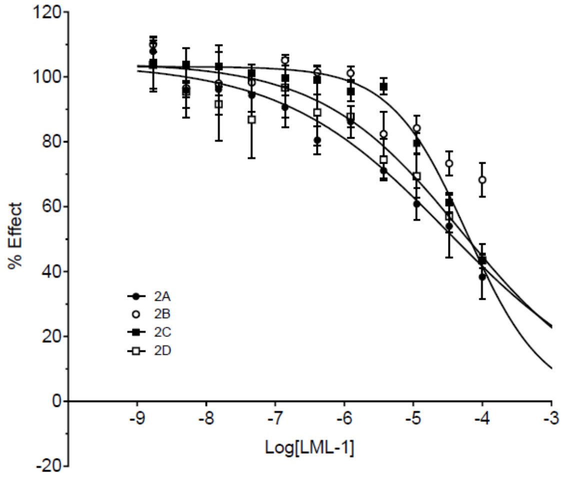

(3*R*,6*R*)-**ML**

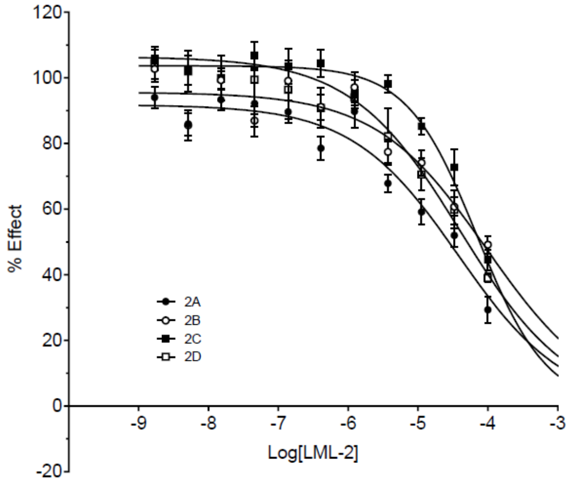

(3*S*,6*S*)-**ML**

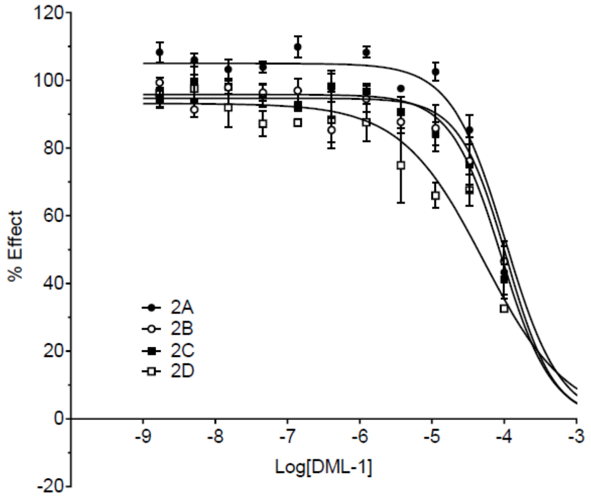

(3*R*,6*S*)-**ML**

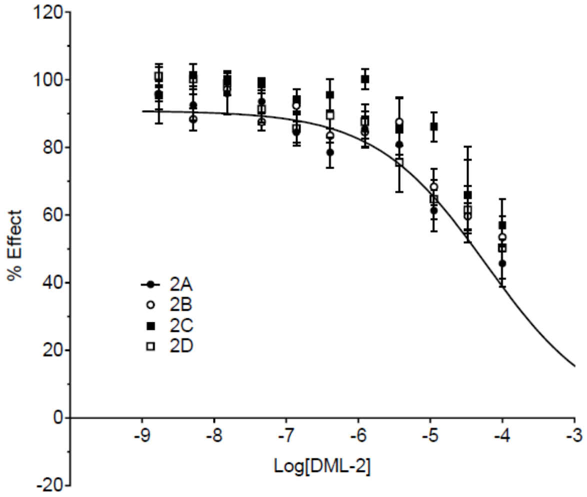

(3*S*,6*R*)-**NML**

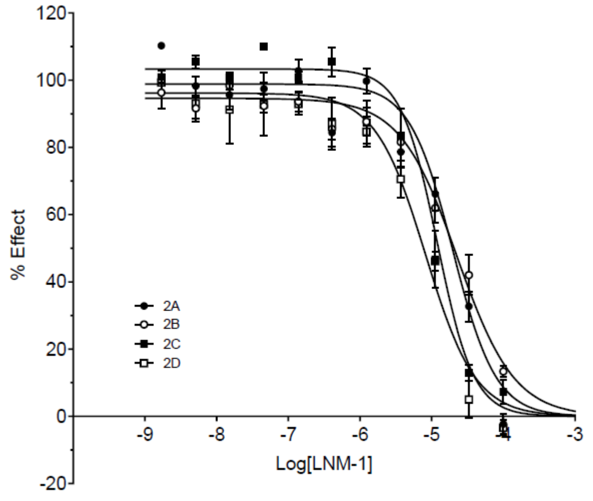

(3*R*,6*R*)-**NML**

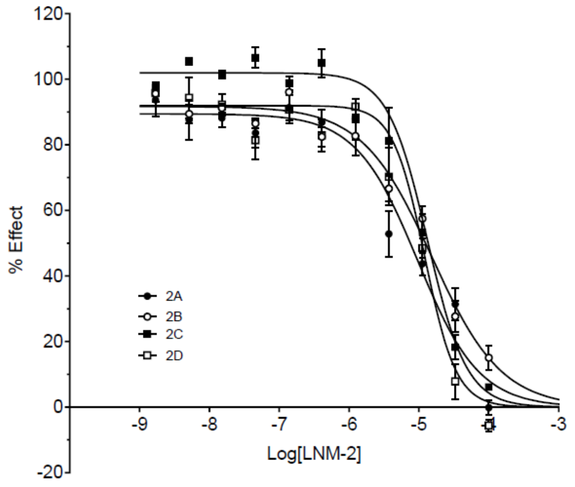

(3*R*,6*S*)-**NML**

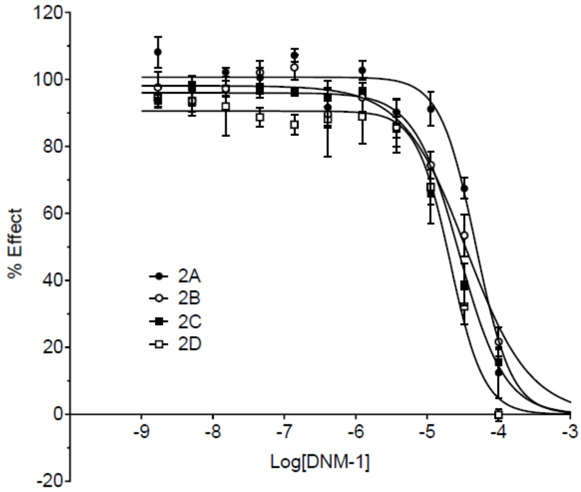

(3*S*,6*S*)-**NML**

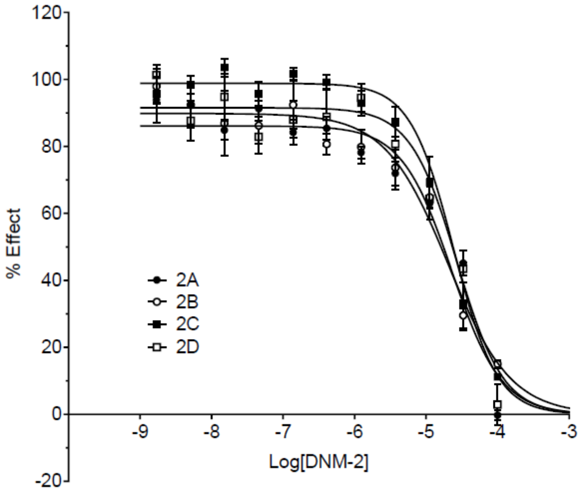

(3*S*,6*R*)-**DNML**

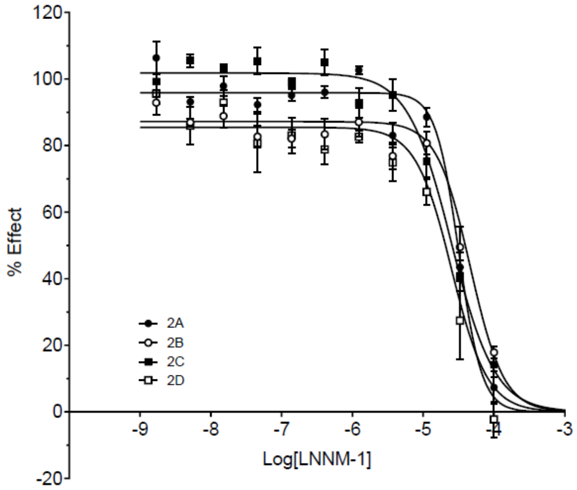

(3*R*,6*R*)-**DNML**

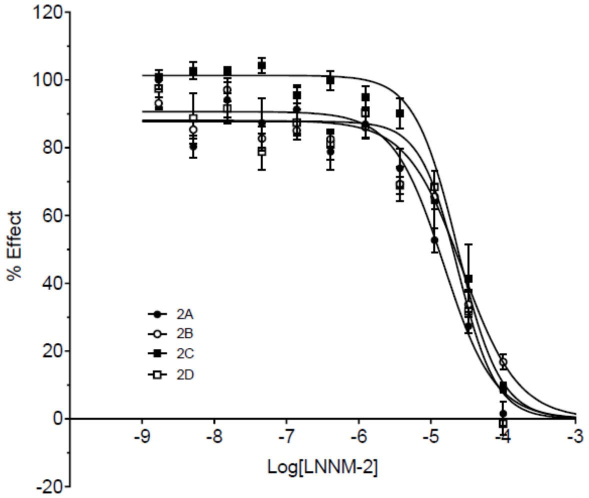

(3*R*,6*S*)-**DNML**

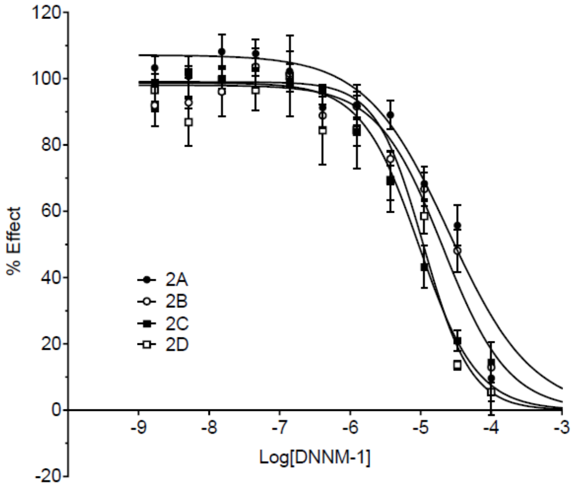

(3*S*,6*S*)-**DNML**

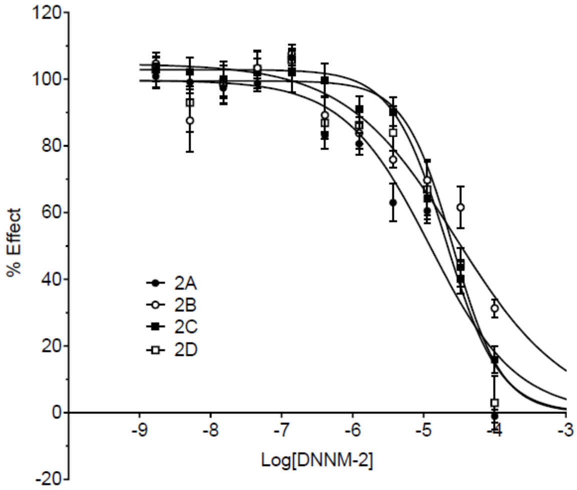

(*R*)-**DDVA**

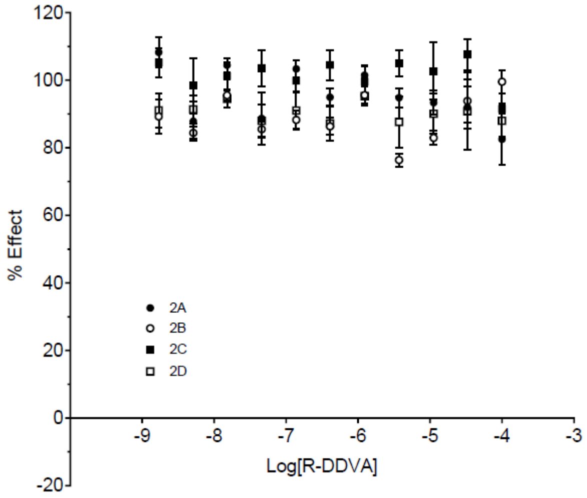

(*S*)-**DDVA**

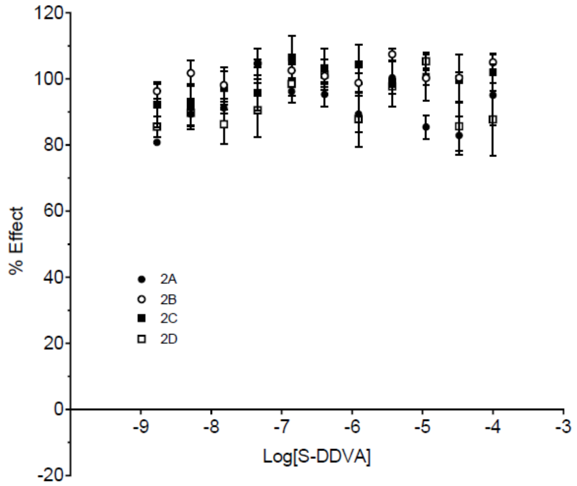

(*R*)-**DDPO**

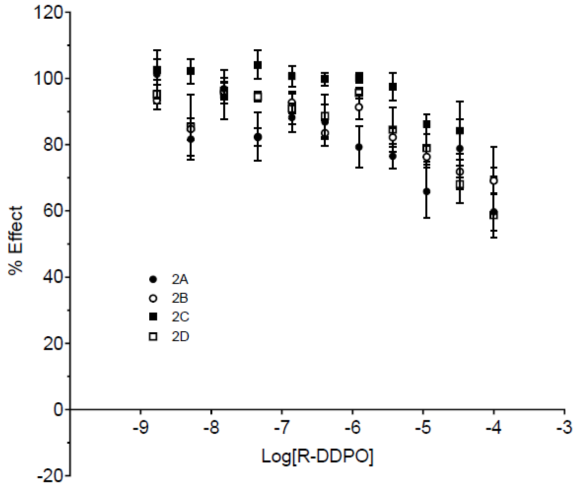

(*S*)-**DDPO**

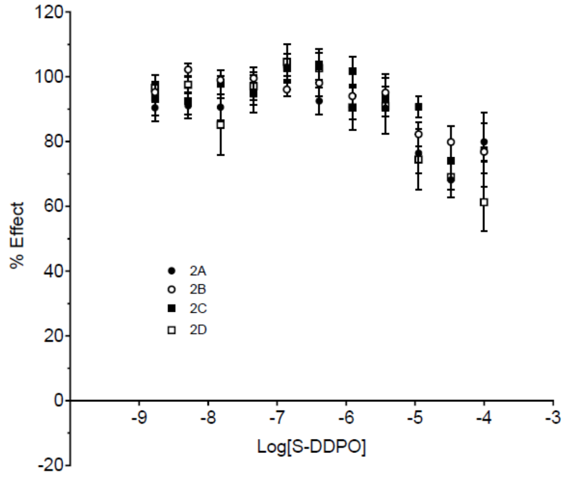

(*R*)-**EDDP**

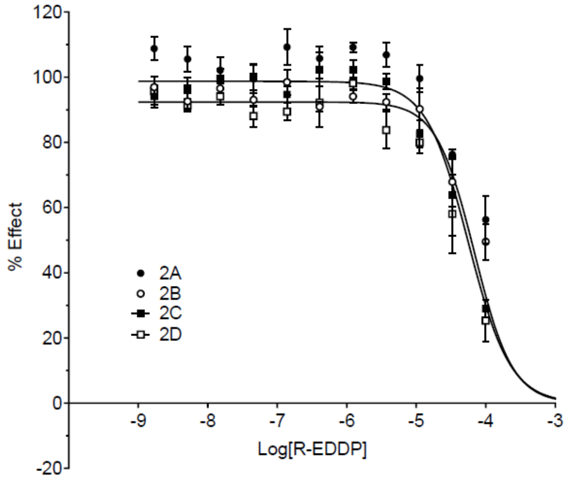

(*S*)-**EDDP**

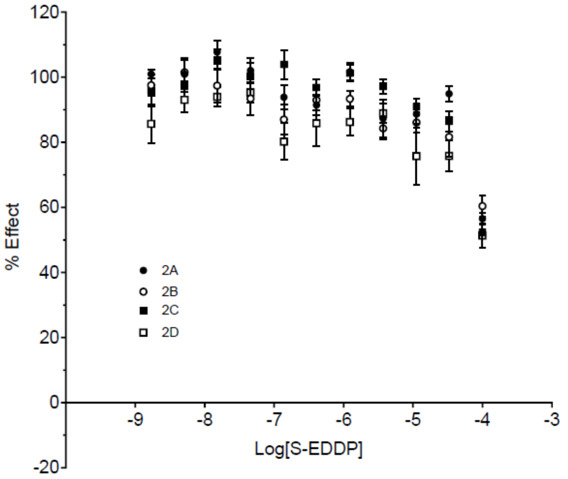

(*R*)-**EMDP**

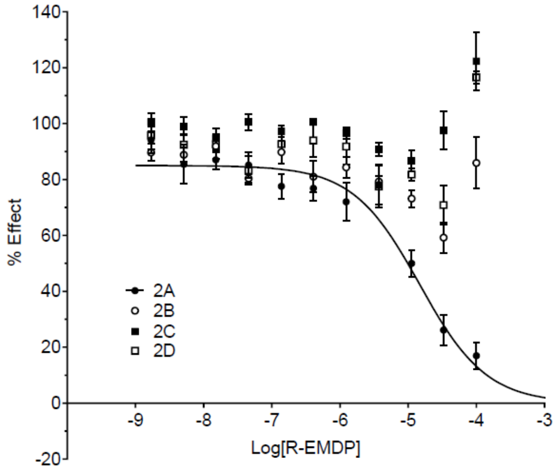
140
120
100
80
60
40
20
0
-20
% Effect
-9
-8
-7
-6
-5
-4
-3
Log[R-EMDP]
2A
2B
2C
2D

(*S*)-**EMDP**

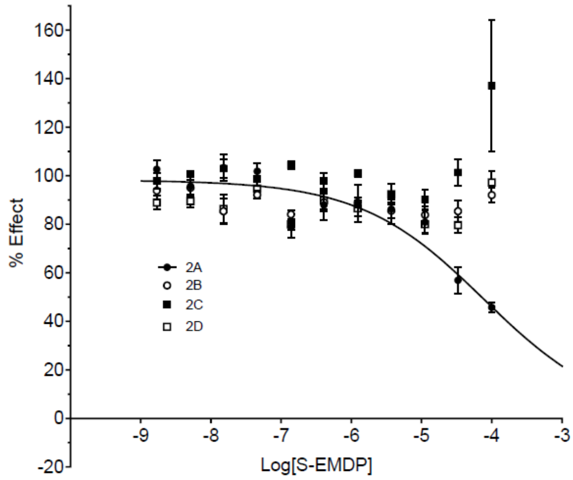
160
140
120
100
80
60
40
20
0
-20
% Effect
-9
-8
-7
-6
-5
-4
-3
Log[S-EMDP]
2A
2B
2C
2D

Radioligand displacement assay results

RECEPTORS, TESTED CONCENTRATIONS		COMPOUNDS									
		**(*S*)-methadone**	(3*S*,6*R*)-**NML**	(3*R*,6*R*)-**NML**	(3*S*,6*R*)-**DNML**	(3*R*,6*R*)-**DNML**	(*R*)-**EMDP**	(3*R*,6*S*)-**NML**	(3*S*,6*S*)-**NML**	(3*R*,6*S*)-**DNML**	(3*S*,6*S*)-**DNML**
**NMDA**	**10 µM**	68	51	57	38	54	4	48	64	63	36
	**1 µM**	23	-5	17	8	12	12	8	9	12	9
**H1**	**30 µM**	73	27	20	28	9	62	43	65	19	24
	**10 µM**	50	7	15	15	3	24	6	33	4	5
**M5**	**10 µM**	73	74	43	39	33	3	46	15	13	9
	**1 µM**	25	21	15	12	-1	6	11	2	9	1
**MOP**	**3 µM**	63	26	11	23	9	8	29	95	26	84
	**0.3 µM**	26	2	4	9	-7	-4	1	65	-3	38
**5-HT$_{1A}$**	**3 µM**	2	-4	-10	-1	4	7	11	1	-1	15
	**0.3 µM**	-2	-1	-9	-11	0	0	5	-4	1	24
**5-HT$_{2A}$**	**3 µM**	72	11	-3	10	8	14	4	-11	9	10
	**0.3 µM**	22	-3	0	9	6	4	0	-10	6	12
**5-HT$_{2C}$**	**3 µM**	77	68	16	27	16	7	5	28	-1	19
	**0.3 µM**	30	15	18	9	7	2	-4	5	3	1
**5-HT$_{5A}$**	**10 µM**	70	42	63	11	-4	0	15	10	16	-12
	**1 µM**	24	4	13	-1	4	1	-1	1	-1	-7
**5-HT$_{7}$**	**10 µM**	58	7	5	5	8	8	28	23	12	4
	**1 µM**	12	11	4	8	12	0	14	12	14	5
**σ1**	**3 µM**	70	26	33	18	47	49	80	77	61	71
	**0.3 µM**	23	-1	4	-4	14	11	47	46	17	46
**NET**	**10 µM**	17	9	14	22	6	27	14	12	5	3
	**1 µM**	5	4	-2	0	4	-5	10	-12	0	-5
**SERT**	**10 µM**	74	61	69	36	20	7	45	25	65	82
	**1 µM**	21	10	18	15	-1	3	12	2	29	35

Radioligand binding assay results, % inhibition of binding. Relevant data (>50% inhibition) are highlighted in green.

Radioligand displacement assay concentration-response curves

Mu opioid receptor (MOP)

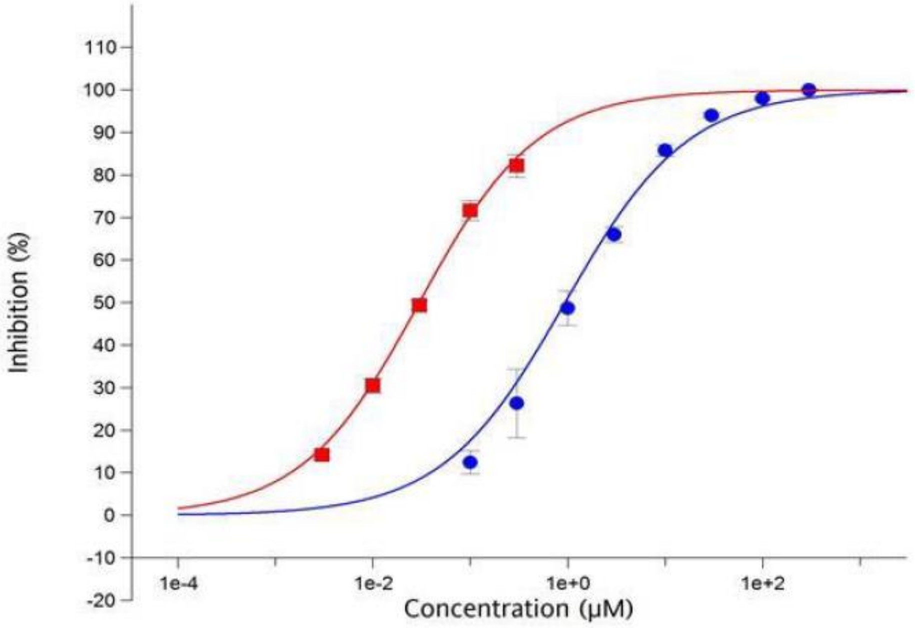

Compound Name	IC50	Ki	nH
Dextromethadone hydrochloride (1275146)	1.13 ± 0.19 μM	0.46 ± 0.078 μM	0.81 ± 0.12
DAMGO	0.031 ± 0.001 μM	12.7 ± 0.58 nM	0.74 ± 0.015

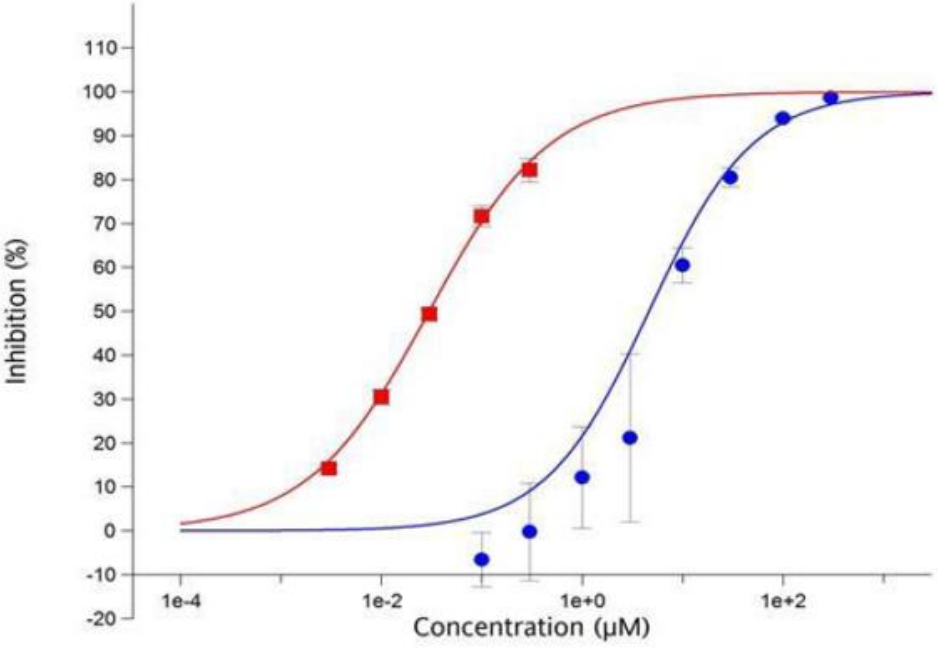

Compound Name	IC50	Ki	nH
(3S,6R)-NML	7.47 ± 2.85 μM	3.03 ± 1.16 μM	1.27 ± 0.42
DAMGO	0.031 ± 0.001 μM	12.7 ± 0.58 nM	0.74 ± 0.015

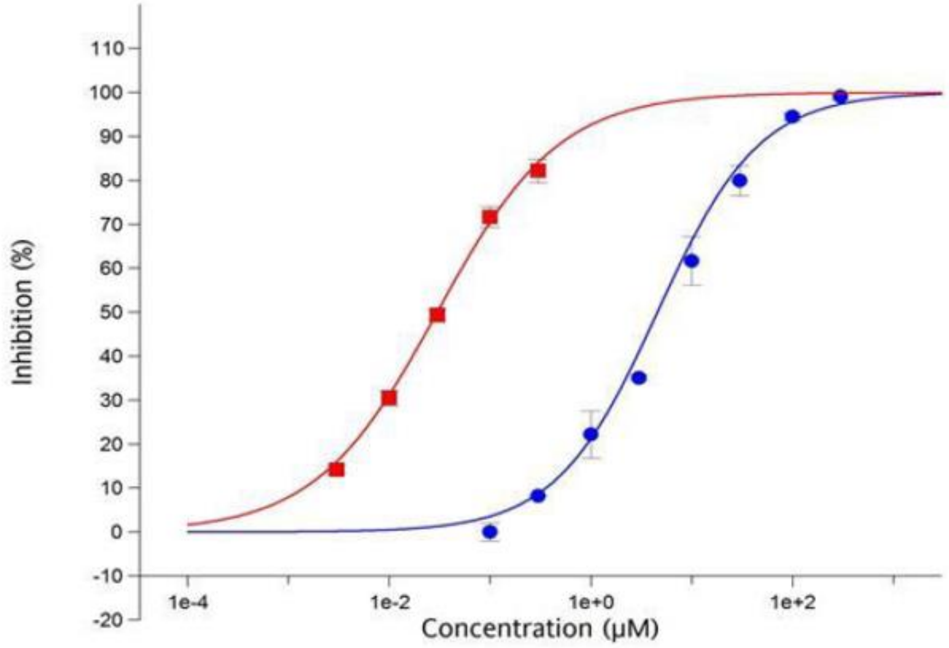

Compound Name	IC_{50}	K_i	n_H
(*3R*,*6R*)-NML	5.71 ± 1.15 µM	2.32 ± 0.47 µM	0.86 ± 0.001
DAMGO	0.031 ± 0.001 µM	12.7 ± 0.58 nM	0.74 ± 0.015

N-methyl-D-aspartate receptor (NMDAR)

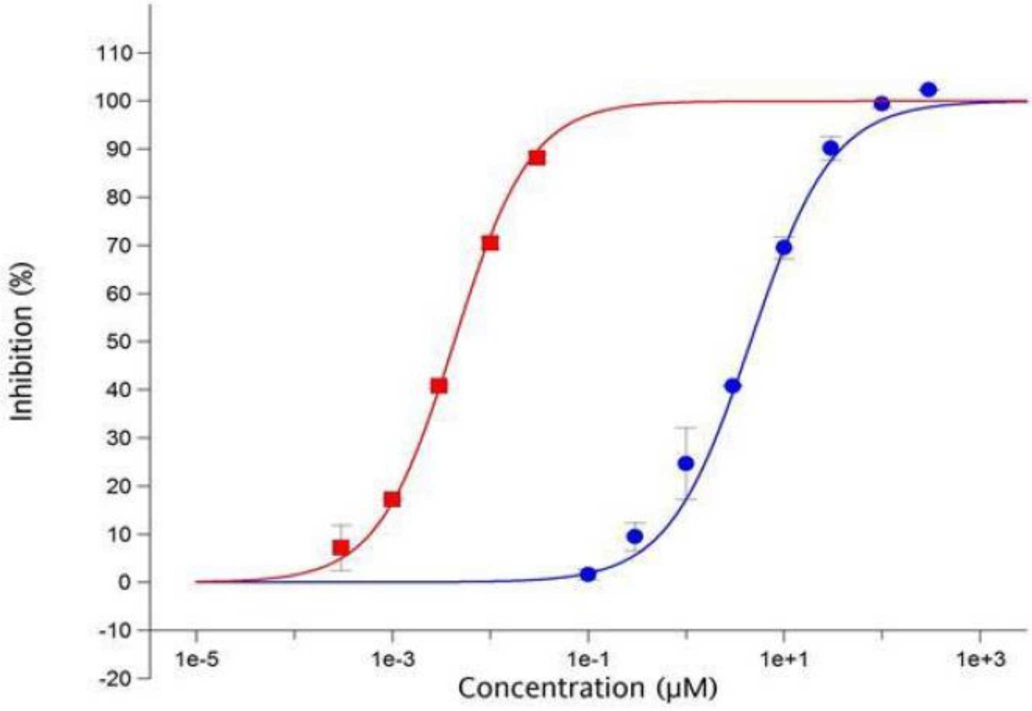

Compound Name	IC_{50}	K_i	n_H
Dextromethadone hydrochloride (1275146)	3.90 ± 0.64 µM	2.64 ± 0.43 µM	0.97 ± 0.064
Dizocilpine ((+)-MK-801)	4.32 ± 0.040 nM	2.93 ± 0.027 nM	1.03 ± 0.076

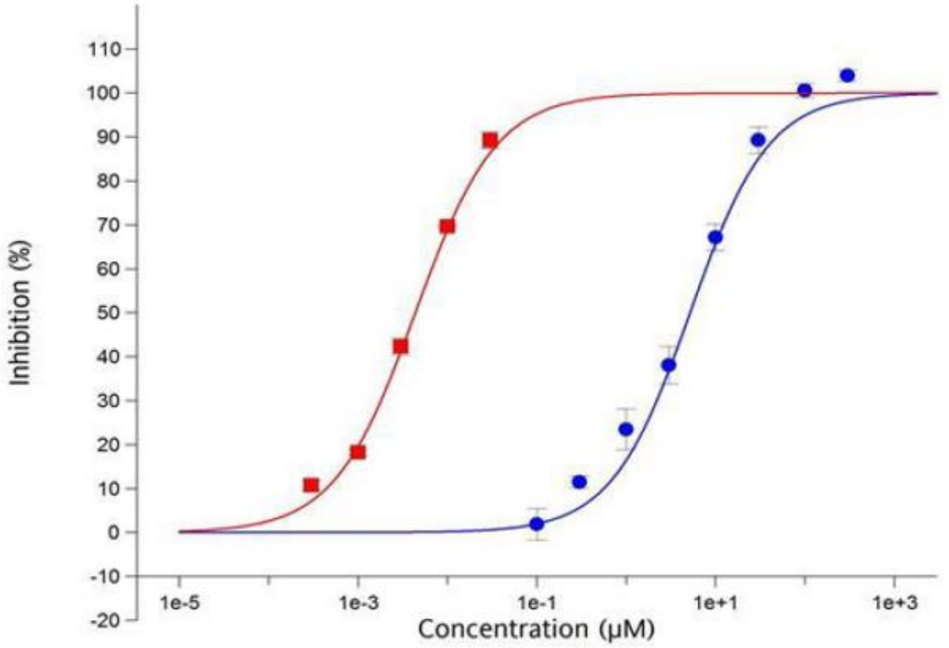

Compound Name	IC_{50}	K_i	n_H
● (*3S*,*6R*)-NML	4.32 ± 0.86 μM	2.92 ± 0.58 μM	0.97 ± 0.019
■ Dizocilpine ((+)-MK-801)	4.13 ± 0.23 nM	2.80 ± 0.15 nM	0.98 ± 0.023

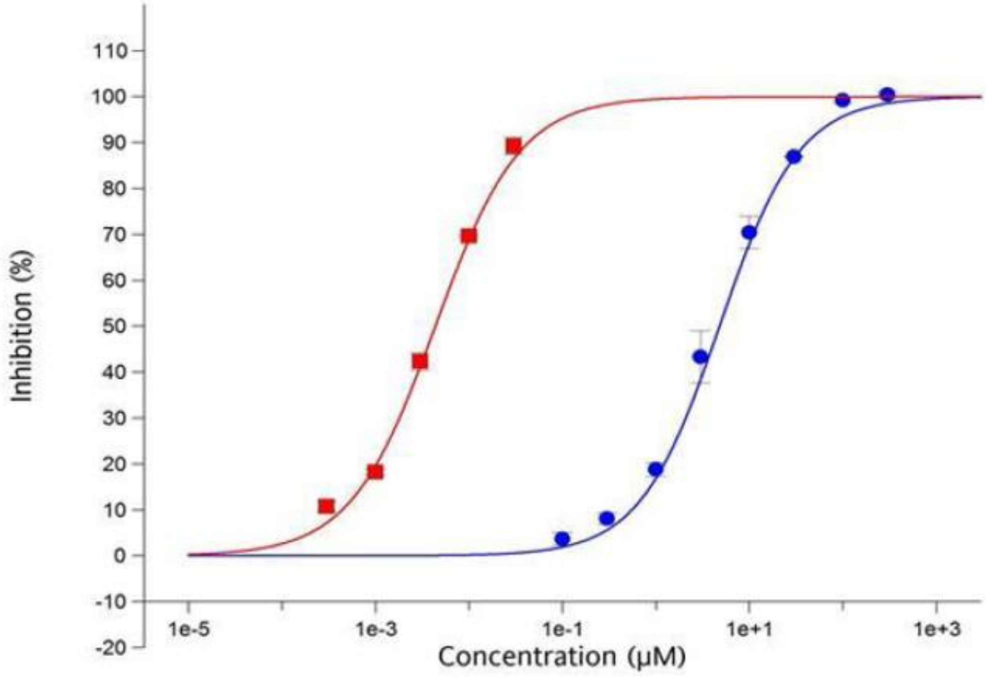

Compound Name	IC_{50}	K_i	n_H
● (*3R*,*6R*)-NML	4.11 ± 0.68 μM	2.78 ± 0.46 μM	1.01 ± 0.011
■ Dizocilpine ((+)-MK-801)	4.13 ± 0.23 nM	2.80 ± 0.15 nM	0.98 ± 0.023
